# From atoms to a data bank: optimizing transferability of electron-density symmetry

**DOI:** 10.1107/S2053273326004651

**Published:** 2026-06-12

**Authors:** Paulina Maria Rybicka, Marta Kulik, Vladislav Ignat’ev, Paulina Maria Dominiak

**Affiliations:** a University of Warsaw Faculty of Chemistry, Biological and Chemical Research Centre Żwirki i Wigury 101 Warsaw 02-089 Poland; Ernst Ruska-Centre for Microscopy and Spectroscopy with Electrons, Forschungszentrum Jülich, Jülich, 52428, Germany

**Keywords:** quantum crystallography, electron density, multipolar atom types from theory and statistical clustering, MATTS, transferable aspherical atom model, TAAM

## Abstract

The multipole model provides a significantly improved description of electron density compared with the spherical atom approximation and is widely applied in the crystallographic refinement of X-ray diffraction data. The multipolar atom types from theory and statistical clustering (MATTS) data bank collects multipole model parameters for atom types. These parameters are derived from quantum chemical calculations performed on experimental geometries of model molecules and rely on the concept of transferability between chemically similar atoms. An essential component of each atom type is the definition of the local coordinate system (LCS) and the symmetry of the electron density, which so far have been selected individually using expert judgment during data bank construction. In this work, we focus on the electron densities of atoms from model molecules and atom types from the MATTS data bank. We introduce a systematic procedure in which symmetry constraints are removed during multipole model refinement of model molecules and multiple types of LCS are tested using chemically meaningful directions. We examine how different LCS choices influence the pseudosymmetry, the apparent symmetry of electron density, identified empirically based on the refined multipole model parameters and their statistical significance. Our results show that refinement without symmetry constraints improves the representation of pseudosymmetry and, in some cases, leads to changes in both the values of the multipole model parameters and the assigned symmetry of the electron density. We propose an optimal LCS for each topological kind of atom type and provide clear criteria for symmetry assignment. This work contributes to the future development of the MATTS data bank with improved descriptions of LCSs and transferability of electron-density symmetry.

## Abstract 2

This work focuses on the electron density of atoms modeled with a multipolar approach. We investigate how the refinement of the multipole models with/without symmetry constraints and optimization of the orientation of the local coordinate system and pseudosymmetry assignment improves the accuracy of electron-density representations for atoms and atom types in a pseudoatom data bank.

## 1. Introduction

A physically valid and accurate model of electron density is essential for crystal structure determination. The earliest approach, the independent atom model (IAM) (Compton, 1915 ▸), treats atoms as spherical and neutral (or with integer charges). While simple and widely used, the IAM neglects deviations from the spherical shape of the valence shell of atoms in crystals, mostly caused by the valence electrons in lone electron pairs and covalent or non-covalent bonds. For X-ray diffraction, this usually leads to positive residual density in bonding regions, underestimated *X*–H bond lengths and overestimated atomic displacement parameters (Bąk *et al.*, 2011 ▸; Woińska *et al.*, 2016 ▸; Jha *et al.*, 2020 ▸; Sanjuan-Szklarz *et al.*, 2020 ▸). The IAM is therefore a reasonable approximation only for heavier atoms where core electrons dominate, but inadequate for lighter atoms where valence electrons shape the density.

To address the limitations of the IAM, quantum crystallography has introduced aspherical approaches to electron-density modeling (Genoni *et al.*, 2018 ▸; Kulik & Dominiak, 2022 ▸; Krawczuk & Genoni, 2024 ▸). These include methods either focusing on molecular wavefunctions (Jayatilaka & Dittrich, 2008 ▸; Sironi *et al.*, 2007 ▸) or on electron density (Hansen & Coppens, 1978 ▸; Stewart, 1976 ▸). Models more accurate than the IAM are mainly used in X-ray crystallography but can be applied to electron diffraction (3D-ED, micro-ED) and cryo-electron microscopy (cryo-EM) (Yonekura & Maki-Yonekura, 2016 ▸; Gruza *et al.*, 2020 ▸; Kulik *et al.*, 2022 ▸; Kumar *et al.*, 2024 ▸; Olech *et al.*, 2024 ▸; Pacoste *et al.*, 2024 ▸).

In this work, we focus on the Hansen–Coppens multipole model which represents the electron density of a molecule as a sum of contributions from individual pseudoatoms (Hansen & Coppens, 1978 ▸). This allows the electron density around each nucleus to be modeled in different directions, capturing chemical features such as bonds and lone electron pairs. The pseudoatom electron density [equation (1)] is described in spherical coordinates (*r*, θ, φ) as the sum of three components: frozen spherical core electron density, spherical valence, and aspherical deformation valence described by the sum of atom-centered real spherical harmonics (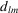) and radial terms (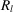). 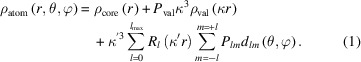
The parameters of electron density responsible for spherical and aspherical deformations are known as the multipole model parameters. These include parameters of contraction–expansion for spherical (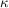) and deformation (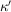) valence electrons, and populations of the valence electrons (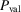) and the deformation functions (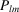). The 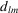 and thus 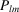 are defined in the atom-centered Cartesian local coordinate system (LCS).

A full multipole model refinement requires high-quality, complete and high-resolution data with minimal disorder. When such data are unavailable, the transferable aspherical atom model (TAAM) provides a practical alternative to full multipole model refinement. The central concept of TAAM lies in the transferability of multipole parameters for atoms in similar chemical environments (Brock *et al.*, 1991 ▸); thus, each atom can be represented by an atom type (Pichon-Pesme *et al.*, 1995 ▸). The electron density of an atom type should be as similar as possible to the electron densities of the atoms it is meant to represent. Atom types are stored in data banks, such as the MATTS (multipolar atom types from theory and statistical clustering) data bank (Jha *et al.*, 2022 ▸; Rybicka *et al.*, 2022 ▸) and its previous version UBDB (Koritsanszky *et al.*, 2002 ▸; Volkov *et al.*, 2004 ▸; Kumar *et al.*, 2019 ▸), ELMAM2 (Pichon-Pesme *et al.*, 1995 ▸; Jelsch *et al.*, 1998 ▸; Domagała & Jelsch, 2008 ▸, Domagała *et al.*, 2012 ▸) and GID (Dittrich *et al.*, 2004 ▸; Dittrich *et al.*, 2013 ▸). Pre-calculated multipole parameters are transferred from a data bank to the refined atomic model and during a TAAM refinement (Brock *et al.*, 1991 ▸; Bąk *et al.*, 2009 ▸) the same set of parameters is refined as in standard IAM refinement.

In this work, we focus specifically on the transferability of electron-density symmetry between individual atoms and their corresponding atom types in the MATTS data bank (Jha *et al.*, 2022 ▸; Rybicka *et al.*, 2022 ▸). The MATTS data bank is built with a set of model molecules with good-quality experimental geometry for which electron densities are obtained using quantum mechanical calculations and represented in the multipole model formalism. Atoms from these model molecules that share similar chemical environments (*i.e.* have similar local topologies) and simultaneously share similar values of multipole model parameters are grouped together and, for each group, an atom type is defined. In newer versions of the data bank, machine learning methods are increasingly applied to aid the clustering and classification of atoms represented by multipole model parameters only into groups used to define atom types (Rybicka *et al.*, 2022 ▸). The multipole parameters of an atom type are obtained by averaging the multipole parameters of individual atoms coming from model molecules and assigned to that type. The MATTS2021 data bank includes 651 atom types for H, C, N, O, P, S, F, Cl and Br.

The definition of an atom type in the MATTS data bank includes three components that are connected with one another: the local topology, the LCS in which the 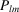 parameters are expressed, and the symmetry that these parameters fulfill. The symmetry refers to the local symmetry of the electron density of an atom or atom type as represented by the multipole expansion, not the global symmetry of the molecule or crystal. Correct representation of atoms using the averaged description provided by an atom type and, thus, correct transfer of multipole parameters, depends critically on how well these three components are defined and if they are consistent with each other.

Topology is the characterization of a central atom and its nearest neighbors, taking into account their connections through chemical bonds and their spatial arrangement. In particular, the topology definition takes into account the type of chemical element, the number of bonded atoms, the planarity of the group (of the analyzed atom and atoms directly bonded with it, *i.e.* its first neighbors), membership in a planar or non-planar ring and the ring size (from three to eight atoms). Nearest neighbors are atoms bonded stepwise to the central atom: first are directly bonded, while second, third and fourth extend outwards through successive bonds. Hybridization, lone electron pairs and bond orders are not directly specified but can be inferred from topology. Atoms can be divided into topological groups according to the number of first neighbors and planarity. For each group the symmetry and LCSs will be considered separately in this work.

The LCS is defined by specifying two neighboring atoms, towards which two selected axes of the LCS are oriented. Alternatively, instead of a single atom, an axis can also be placed in the average direction towards two or three atoms (see the supporting information S1). The third axis is defined to maintain a right-handed (R) LCS. This definition limits the possible LCS orientations in 3D space, though multiple possibilities still exist. It is important to note that the 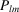 values depend on the LCS orientation, *i.e.* different LCS orientations yield different 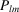 values for the same electron density. Currently, the LCS orientation specified in the atom-type definition is selected individually using expert judgment to satisfy three criteria: (*a*) the LCS is optimal with respect to the symmetry inferred from the atom type’s topology, (*b*) it is consistent with the symmetry observed for the electron density represented by the averaged 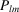 values specified in the atom type, and (*c*) it aligns with the symmetry of the electron densities of the atoms used to parameterize the atom type. For consistency, the same LCS orientation is applied to all atoms within a given atom type before multipole model parameters are averaged. If a different LCS orientation was used during the refinement of model molecules, the LCS is rotated to the orientation defined in the atom type. This increases the likelihood that averaged 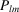 values reliably approximate the aspherical deformation of the electron density of atoms. Misaligned LCS axes in the averaging procedure may cause certain averaged 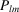 components to vanish (*i.e.* to average to zero). Consequently, the asphericity may be progressively suppressed and, in the limit of extensive averaging over randomly oriented LCS frames, may disappear entirely.

There may be two different perspectives on how symmetry of an atom type is determined: from topology and from electron density. The determination of an atom-type symmetry from its topology relies on the number of first neighbors, group planarity and equivalency of the first neighbors. The first neighbors are equivalent if they have the same topology specified in the atom-type definition. Sometimes, the number of second neighbors and its equivalency has to be taken into account. The symmetry inferred from topology provides an initial expectation of the symmetry of the electron density, but it is the symmetry of electron density that has a decisive role when the final symmetry is assigned to atom type.

To directly determine the symmetry of an atom type from its electron density represented by multipolar expansion, the LCS should be oriented so that its axes lie appropriately with the symmetry elements. In this context, ‘symmetry’ denotes the symmetry group, usually a point group. In some cases, cylindrical (axial) symmetry is present, which may be described by a continuous rotational symmetry rather than a crystallographic point group. For most symmetry groups, especially of a higher order, there is a single optimal LCS orientation, *e.g.* with the *Z* axis placed along the highest-order (principal) rotation axis, whereas for lower symmetries, multiple, equally valid LCS orientations may exist. Each symmetry, together with its associated LCS orientation, leads to a specific set of multipolar functions that should vanish, *i.e.* their populations (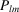 values) should be equal to zero (Kurki-Suonio, 1977 ▸). Thus, determining the symmetry reduces to checking which 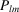 values are zero. In the case of atom type, 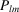 values represent the average (mean) values across all atoms belonging to the atom type. The following criteria were used in the construction of the MATTS2021 data bank, to decide if values can be considered statistically and genuinely equal to zero: 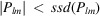 and 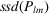 ≤ 0.05, where *ssd* is sample standard deviation (Jha *et al.*, 2022 ▸). The 0.05 threshold value in the MATTS2021 data bank was chosen arbitrarily (Jha *et al.*, 2022 ▸). Other arbitrarily chosen *ssd* thresholds were used to control the acceptable variability of the remaining multipole model parameters: 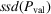 ≤ 0.1, 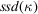 ≤ 0.01 and 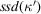 ≤ 0.1.

Until recently, the symmetry of electron density for atoms used to build atom types in the MATTS data bank was known *a priori*, since symmetry constraints were imposed during multipole refinement of model molecules. Therefore, it was easy to define symmetry of atom types if the type collected atoms refined with the same symmetry constraints. However, it could happen that a given definition of topology selected atoms refined with different symmetry constraints (the algorithm defining symmetries in refinement is much simpler and more general than the atom-type definitions in the MATTS data bank). Then, the consistency between topology, LCS, atom-type symmetry, averaged 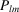 values and symmetry of contributing atoms was achieved iteratively.

As the MATTS data bank expands to include more complex chemical topologies, defining appropriate symmetry constraints during multipole refinement becomes increasingly difficult. In many cases, the symmetry of the electron density cannot be reliably predicted *a priori* from topology. As a result, an increasing number of model molecules have been refined with reduced or no symmetry constraints. Consequently, non-automated monitoring on a case-by-case basis to verify whether the symmetry of the topology and electron density of an atom type truly represents that of its constituent atoms has become progressively less feasible and more prone to errors.

To address this, here we propose: (*a*) refinement of all model molecules without symmetry constraints, allowing the true symmetry of an atom’s electron density to emerge naturally from 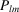 values, (*b*) acceptance of the fact that for most atoms the true symmetry is 1, and that a search for pseudosymmetry (cases where electron density nearly, but not perfectly, fulfills the symmetry) should instead be undertaken, and (*c*) introduction of automatic *post-hoc* assignment of electron-density pseudosymmetry based on (*d*) probabilistic thresholds that define when 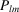 can be considered zero (‘zero-value thresholds’).

A further difficulty arises from the dependence of the 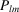 values on the LCS orientation. In theory, one could search the full 3D rotational space to find the LCS orientation that maximizes the pseudosymmetry of the electron density. This might be feasible for atoms themselves but not for atom types and data bank construction. Such a search would result in LCS orientations that differ from one atom to another. However, data bank construction requires equivalent LCS orientations for all atoms representing a given atom type to enable proper clustering/classification and averaging of the 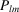 values. In addition, existing software implementations of the multipole model further restrict the definition of LCSs to a limited set of conventions tied to the bonding topology.

Despite these limitations, many LCS orientations remain available. In this work, we assigned the pseudosymmetry of atom and atom-type electron densities, as they appear from the 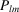 values, by simultaneous analysis of several carefully selected LCS orientations. LCS orientations included in this work differ slightly from, and represent an expanded set compared with, those implemented in our previous study (Rybicka *et al.*, 2022 ▸). In that work, the main focus was on comparing different topological groups of atom types with one another. Here, we included various types of LCS orientations with the focus on that they: (*a*) are needed to capture the symmetries of most atom types in MATTS, (*b*) involve in their definitions a varying number of neighboring atoms, and (*c*) either cover the widest possible range of symmetries or specifically target a given symmetry. Simultaneous analysis ensured that the symmetry assignment was not biased by the choice of LCS orientation.

With the pseudosymmetry established, it finally allowed us to select the LCS orientation *optimal* for a given topological group of atoms, to be used in future data bank construction. Depending on the symmetry, one or several LCS orientations can be optimal. The *optimal* LCS orientation should have a few characteristics: (*a*) it should allow the true symmetry of the electron density to be directly observable and revealed in the 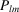 values, (*b*) it should match the topology, with the possibility that symmetry of electron density can be lower than symmetry inferred from the topology, (*c*) it should take into account topology information that is not explicitly defined in the atom type but can have a major influence on the symmetry of the electron density, such as the presence of lone electron pairs, difference in bond orders and intramolecular through-space polarization effects, (*d*) it should minimize artificial symmetry increase during averaging of atoms, maximize the transferability of symmetry from atoms to atom types, (*e*) it should not favor one symmetry over another, allowing unbiased representation of all possible symmetries, (*f*) it should enable observation of as many distinct symmetries as possible and thus help to find atoms having similar electron densities with machine learning models used for the construction and expansion of the data bank, (*g*) it should minimize the number of dominating 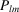, maximize the number of 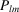 averaging to 0 to potentially speed up calculations necessary for TAAM refinement and to avoid overfitting in applications going beyond TAAM refinement (Zarychta *et al.*, 2011 ▸; Krause *et al.*, 2017 ▸), (*h*) it should not require an excessive use of dummy atoms necessary to orient the LCS to avoid limitations of existing software implementations of the multipole model.

This work contributes to developing an improved version of the MATTS data bank with optimized selection of LCS orientations and symmetry of the electron density for atom types. We investigate how to optimize the transferability of symmetry between atoms and atom types, how symmetry can be reliably determined, how suitable zero-value thresholds can be established, and how the LCS choice affects the visibility of the true symmetry of the electron density and its transferability. The work also explores whether releasing symmetry constraints during refinement provides a better foundation for data bank construction and more accurate symmetry representation.

## 2. Methods

### 2.1. Refinement of model molecules with no symmetry constraints

The set of crystal structures from the Cambridge Structural Database (Groom *et al.*, 2016 ▸) used in this work is the same as the one employed to construct the MATTS2021 data bank with the previously established refinement procedure (Jha *et al.*, 2022 ▸). Alterations were made at the stage of refining the multipole model for model molecules, in particular when applying symmetry constraints to refined 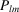 parameters. Two distinct datasets of refined multipolar models were thus created: one resulting from the refinements with the original symmetry constraints that were used for the construction of the MATTS2021 data bank (ref-SC), and another from refinements with no symmetry constraints (ref-NSC). The removal of the symmetry constraints enabled the refinement of all multipoles, increasing the number of refined parameters. The multipolar functions up to the quadrupoles (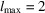) were used for hydrogen atoms and up to hexadecapoles (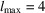) for non-hydrogen atoms. For hydrogen atoms only 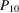 and 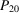 were refined as they describe the electron density along the *X*–H bond for the type of LCS used in the refinement. In the ref-SC dataset, the symmetry constraints were applied for all atoms except in instances where the symmetry was 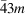 or 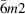. No symmetry constraints were used in the case of the symmetry 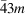. For the 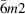 symmetry constraint 3*m* was applied.

### 2.2. Topological groups and subgroups of atoms and atom types

Each of the 651 atom types in the MATTS2021 data bank (available at https://github.com/discamb-project), as well as the atoms in model molecules used to define them, were classified according to the number of first neighbors and the planarity of the group formed by the central atom and its neighbors (‘group planarity’). This yielded eight groups: 6n (six neighbors, non-planar), 4n (four neighbors, non-planar), 3n (three neighbors, non-planar), 3p (three neighbors, planar), 2p (two neighbors, non-collinear), 2x (two neighbors, collinear), 1p (one neighbor, with first and second neighbors non-collinear) and 1x (one neighbor, with first and second neighbors collinear). Planarity was determined using the ‘planarity e.s.d.’ value calculated for each atom and its first neighbors, with a group planarity threshold of 0.1 Å used to distinguish between planar (≤ 0.1 Å) and non-planar (> 0.1 Å) atoms (for further details on the planarity definition see the supporting information S1). These groups were further divided into subgroups based on the central atom chemical element (Table S1.1). The 1p group was additionally divided based on the number of second neighbors (two or three), with the suffixes ‘-2’ or ‘-3’ added to the name, respectively. In total, 14 out of 25 subgroups with 602 of the 651 atom types (92.5%) from the MATTS2021 data bank were included in this work. For details on excluded subgroups see the supporting information S1.

### 2.3. Electron-density symmetries inferred from topology

**Figure 1:**
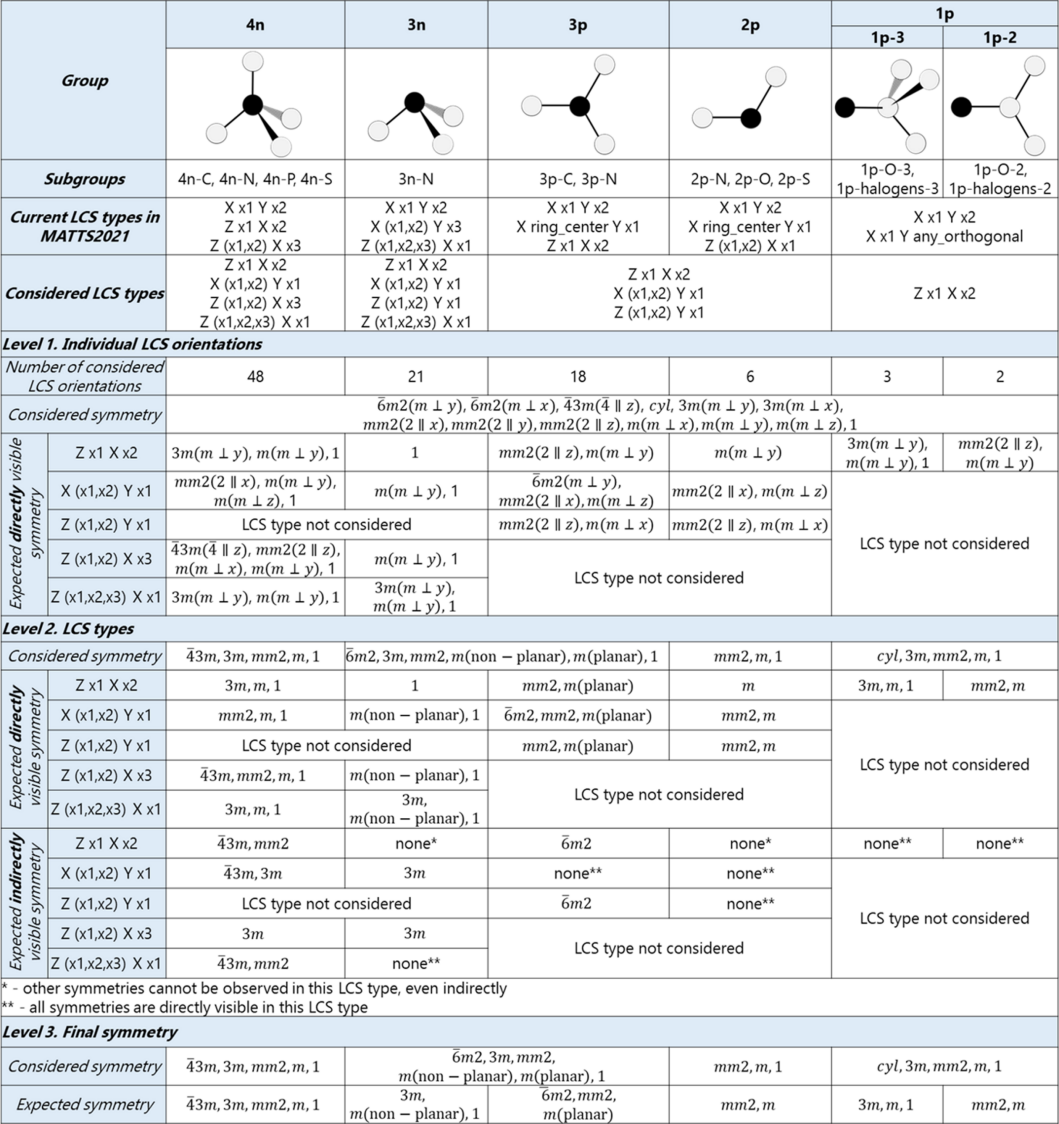
Representation of groups and subgroups of atoms and atom types included in this work. The table presents current and considered LCS types, number of considered LCS orientations, and considered and expected symmetry groups at each level of the analysis. The graphical overview of different LCS types used in MATTS2021 and statistics per subgroup are provided in the supporting information (Tables S1.2–S1.3).

For each topological group, the possible symmetry point groups are limited (Fig. 1 ▸) and depend on the equivalency of electron-density fragments pointing towards the first neighbors (supporting information S1). For the 1p group, the highest symmetry depends on the number of second neighbors.

In this work, we used two different notations for the *m* symmetry for atoms and atom types with three first neighbors, depending on the placement of the mirror plane in relation to the central atom and said neighbors. We called these two options *m*(planar) and *m*(non-planar). The *m*(planar) describes a situation where the central atom and all its three first neighbors lie on one plane (within the planarity e.s.d. threshold) and the mirror plane coincides with the atom plane. The *m*(non-planar) describes a situation when the mirror plane is perpendicular to the average plane of the central atom and its three first neighbors. The explicit distinction between *m*(planar) and *m*(non-planar) was essential for analyzing the 3p-N and 3n-N subgroups.

Lone electron pairs, differences in the bond orders, or through-space intramolecular polarization by the non-immediate neighbors may further reduce observable symmetry. Thus, other chemically reasonable symmetries, even if unexpected for a given topological group, were in addition included in the symmetry recognition algorithm (Fig. 1 ▸).

### 2.4. Selection of LCS types and individual LCS orientations within the LCS type

The LCS types selected for this work were defined using the following options: (*a*) the two axes of the LCS were chosen as either *X* and *Y*, *Z* and *X*, or *Z* and *Y*, (*b*) the first axis was directed from the central atom either towards a single first neighboring atom (x1), or, when possible, along the average direction towards two (x1, x2) or three (x1, x2, x3) first neighbors, and (*c*) the second axis was oriented from the central atom towards either one neighbor already involved in defining the first axis or one of the remaining first (if needed second) neighbors. From these options five different LCS types were combined: (*a*) Z x1 X x2, (*b*) X (x1,x2) Y x1, (*c*) Z (x1,x2) Y x1, (*d*) Z (x1,x2) X x3 and (*e*) Z (x1,x2,x3) X x1 (see Table S1.4). A single LCS type corresponds to multiple LCS orientations, which are realized by assigning the abstract directions defined using x1, x2 and x3 to specific atoms from the list of possible first (*a*, *b*, *c*, *d*) or second (*e*, *f*, *g*) neighbors. Based on the selected LCS types, a defined number of possible individual LCS orientations was considered for each topological group: 48 for 4n, 21 for 3n, 18 for 3p, six for 2p, and three or two for 1p, depending on the number of second neighbors (Figs. S1.1–S1.5).

The main changes in the considered LCS types and orientations compared with our previous work (Rybicka *et al.*, 2022 ▸) are as follows: (i) including additional orientations of the Z (x1,x2,x3) X x1 LCS type to ensure more accurate and reliable assignment of 3*m* and 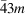 symmetries for the 4n and 3n topological group, and for the correct observation of the lone electron pair in the 3n topological group, (ii) adding the Z (x1,x2) Y x1 LCS type for both the 3n and 3p topological groups to enable comparison with the X (x1,x2) Y x1 LCS type and decide which is more optimal, and simultaneously removing the Z (x1,x2) X x3 LCS type for the 3n group so as to not have an excessive number of LCS types, (iii) for the 1p group, including LCS orientations that also consider second neighboring atoms.

### 2.5. Calculation of multipole model parameters in various LCS orientations

Multipole parameters for all considered LCS orientations across all considered LCS types for the given subgroups (Fig. 1 ▸, Table S1.5) were generated using the *bankMaker* utility program from the *DiSCaMB* library (Chodkiewicz *et al.*, 2018 ▸), with a modified rotation procedure that enabled consideration of all symmetry-allowed neighbor permutations. Two analogous procedures were applied to calculate multipole model parameters for different LCS orientations: one for individual atoms, and the other for atom types. For individual atoms, 14 generalized (universal) atom-type definitions were created for each subgroup (*e.g.* 4n-C, 3p-N), with only planarity of the central atom explicitly defined. Within each universal atom type, the LCS orientation was varied over all considered orientations (Fig. S1.6). These definitions were intentionally broad, ensuring inclusion of all atoms within a given subgroup, regardless of whether a corresponding atom type exists in the MATTS2021 data bank. For atom types, an analogous approach was applied using original MATTS2021 definitions of atom types, edited when necessary to distinguish between neighbors (Fig. S1.7). Rotations were performed on atoms belonging to each atom type, and resulting parameters were averaged within each LCS orientation.

### 2.6. Finding which *P_lm_* can be approximated as zero

**Figure 2:**
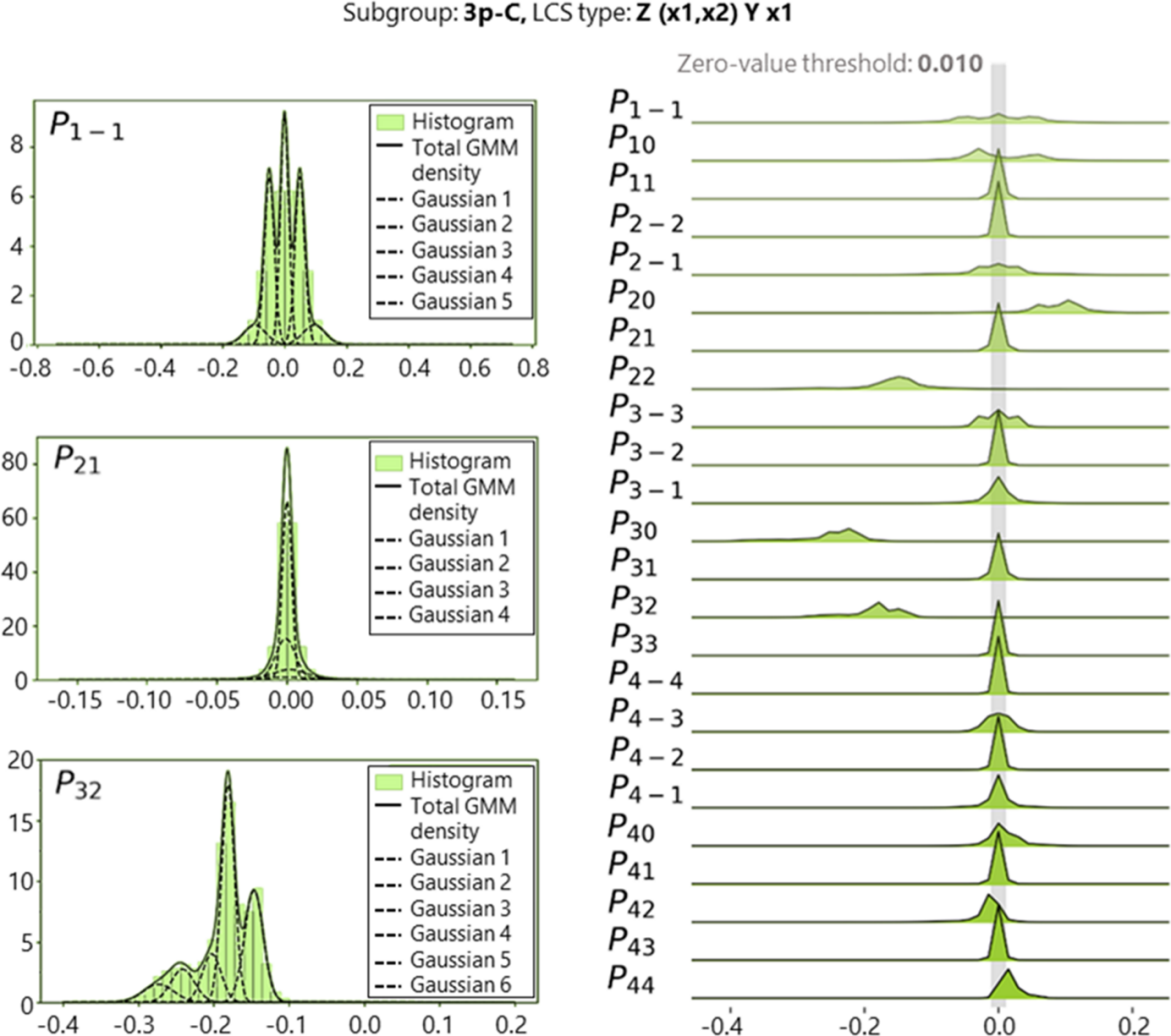
GMM analysis of 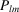 in the Z (x1,x2) Y x1 LCS type (20500 individual LCS orientations) for the 3p-C subgroup. Three exemplary 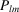 parameters are shown on the left: 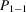 which deviates from a normal Gaussian shape, 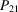 which follows a normal Gaussian shape, and 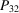 with a global maximum far from zero. The right side of the figure presents a ridgeplot of 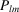 and the section of the data fulfilling the condition 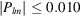 is marked in gray.

To automatically identify the zero-value thresholds, a Gaussian mixture model (GMM) approach was applied. GMM is a probabilistic, parametric clustering method that represents a data distribution as a weighted sum of Gaussian components (Zhuang *et al.*, 1996 ▸). It was chosen because 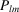 distributions often deviate from a normal Gaussian shape (Fig. 2 ▸, Figs. S2.1–S2.58).

The sets of 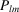 parameters resulting from the previous step of rotating the LCS for atoms for ref-NSC were used for the procedure of identifying the zero-value thresholds. A local Python script automatically determined the optimal number of Gaussian components (from one to six) and calculated their weights, representing contributions to the overall distribution. The procedure was performed separately for each 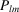 from each LCS type within a given subgroup.

In each LCS type, 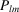 parameters with clearly shifted distributions (*i.e.* their global maximum was far from zero) were automatically excluded from further calculation of the zero-value threshold. To identify such dominant 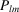 parameters, the Gaussian component with the largest weight was first selected. If the absolute value of its mean was at least three times greater than its standard deviation, that 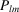 was considered significantly different from zero and excluded.

For all remaining 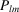 parameters in each LCS type, the Gaussian component with a local maximum closest to zero was identified. The median of standard deviations of these Gaussian components across all 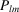 in the LCS type was then taken as a representative value. Finally, the median of these representative values across all LCS types was calculated, multiplied by three, and rounded to three decimal places to define the zero-value threshold for the subgroup (Fig. 2 ▸, Figs. S2.1–S2.58).

The median was used instead of the mean because it is a more reliable measure for skewed data and groups of unequal sizes (Wicox, 2013 ▸; Cao *et al.*, 2020 ▸; Rousselet & Wilcox, 2018 ▸). The selection of the zero-value threshold as three times larger than the median was guided by the empirical rule, also known as the 68-95-99.7 rule (Ross, 2009 ▸).

### 2.7. Electron-density symmetry assignment based on *P_lm_* values

**Figure 3:**
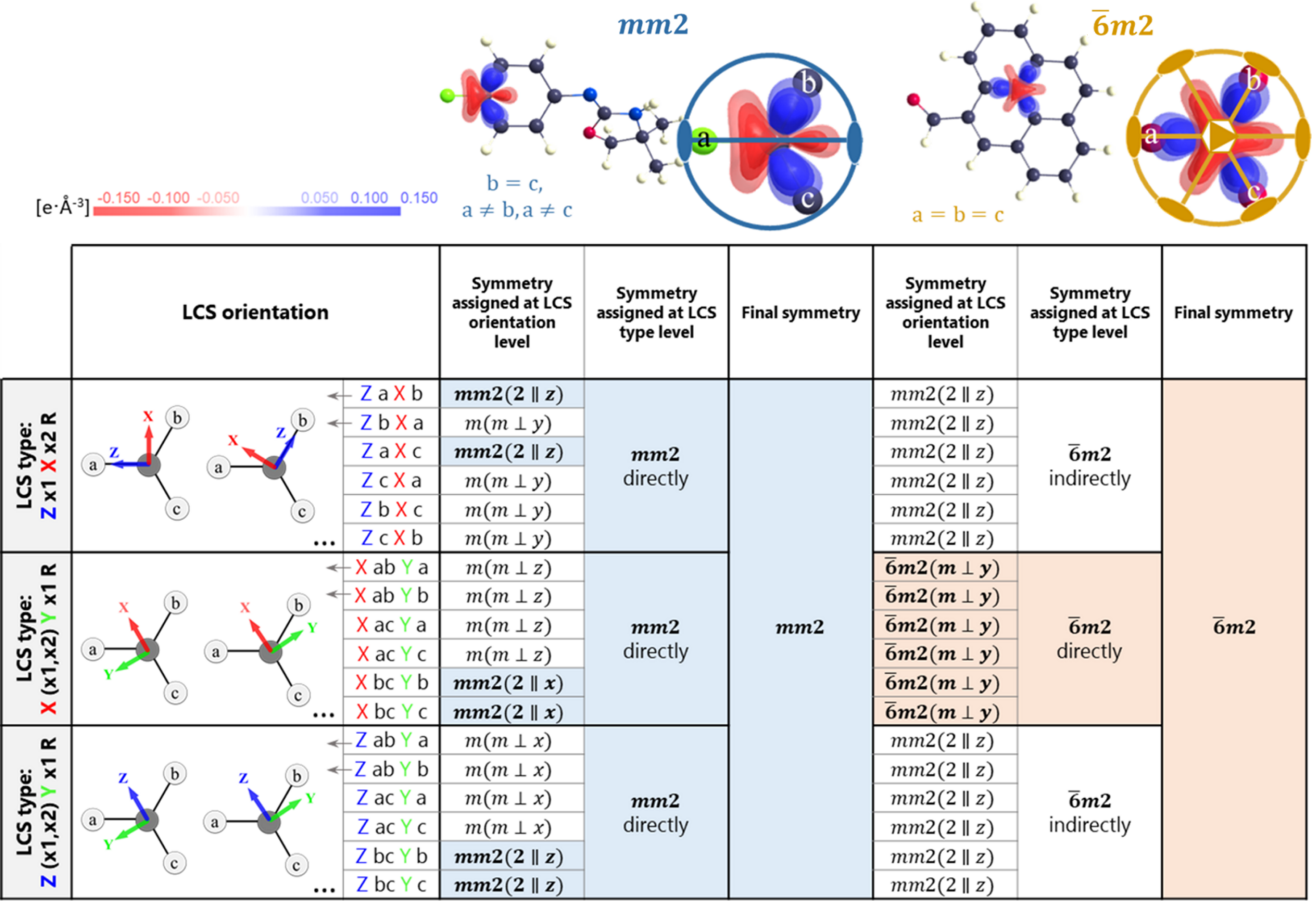
Illustration of the multilevel approach of electron-density symmetry assignment based on 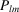 values. An example is given for the *mm*2 and 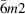 symmetry within the 3p topological group. The *mm*2 symmetry can be directly observed in all three LCS types but only for certain LCS orientations where the mutual arrangement of symmetry elements and LCS axes is optimal. The 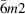 symmetry can be directly observed only in the X (x1,x2) Y x1 LCS type and indirectly in the other two LCS types.

The symmetry of the electron density was assigned through a multilevel hierarchical approach based solely on the values of the 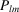 parameters (Fig. 3 ▸). At the first level, a symmetry group with information about the placement of symmetry elements with respect to the LCS axes is assigned independently for each LCS orientation based on the presence or absence of specific 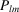 parameters. At the second, these symmetries are evaluated collectively within each LCS type to identify consistent patterns. At this stage, separate analysis paths are followed for direct and indirect symmetry assignment. At the third, results from all LCS types are compared to determine the final symmetry group. In cases of conflicting assignments between LCS types, the lowest consistent symmetry is selected. This multilevel approach is necessary because the observability of symmetry depends on the alignment between symmetry elements and LCS axes, and no single LCS orientation or type is sufficient to capture all symmetry features.

#### 2.7.1. Symmetry assignment at the level of individual LCS orientations

For each atom or atom type, symmetry for each individual LCS orientation was determined by comparing the calculated 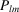 values with the Kurki-Suonio symmetry selection rules (Kurki-Suonio, 1977 ▸), which define the allowed and prohibited multipoles for each symmetry point group (Table S1.6). When the value corresponding to a prohibited multipole vanished, *i.e.* it was zero with the appropriate zero-value threshold, the presence of a specific symmetry was inferred. This approach allows symmetry groups to be recognized directly from the disappearance of selected multipoles (Table S1.7). The rules were applied systematically in the same way across all subgroups and all considered symmetries in the symmetry order as listed in Fig. 1 ▸ and Table S1.6. At this level, the symmetry label includes axis-specific notation to indicate the orientation of the symmetry elements with respect to the LCS axes [*e.g.mm*2(2∥*x*) or *m*(*m*⊥*z*)].

#### 2.7.2. Symmetry assignment at the level of LCS types

Next, all LCS orientations of the atom or atom type within a given LCS type were analyzed together. At this level, each topological group has its own set of topologically relevant symmetries that are considered, unlike the previous level of individual LCS orientations (see Fig. 1 ▸). This level was used to identify consistent symmetry patterns across multiple LCS orientations of the same LCS type for each topological group (Tables S1.8–S1.13). The analysis here addresses three possible situations, ensuring that all symmetry information accessible within a given LCS type is taken into account: (*a*) direct symmetry assignment that occurs when the LCS coordinate axes correctly align with the symmetry elements of a given point group, (*b*) indirect symmetry assignment occurs when the same lower symmetry repeatedly appears across several LCS orientations, oriented in respect to different first neighbors, implying the presence of a higher, underlying symmetry that cannot be directly seen in the given LCS type, and (*c*) symmetry that is not observable within a given LCS type, even if it is present in the electron density.

Not all symmetries can be directly observed in every LCS type, as visibility depends on how the coordinate axes relate to the symmetry elements. Kurki-Suonio’s selection rules for point groups 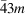, 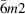 and 3*m* are defined assuming the highest-fold axis is aligned with the *Z* axis (*i.e.*4∥*z*, 6∥*z* or 3∥*z*, respectively). If the *Z* axis is instead aligned with a lower-order symmetry element or another axis (*e.g.X* or *Y*), no straightforward rules exist to determine which 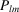 should take the values of zero and which 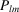 are related to one another by a linear combination, making it difficult to directly assign the full symmetry based on 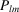 values alone. When the *Z* axis does not coincide with the principal rotation axis, these higher symmetries can still be detected indirectly by identifying repeating symmetry patterns across all orientations of the LCS type. For instance, if all six orientations of the Z x1 X x2 LCS type for the 3p group are consistently assigned *mm*2(2∥*z*), this pattern indicates the presence of the higher 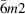 symmetry (Fig. 3 ▸).

In extreme cases, the symmetry cannot be observed either directly or indirectly. In the Z x1 X x2 LCS type, the 3*m* and *m*(non-planar) symmetries cannot be detected for the 3n group because the threefold axis is not parallel and the *m* plane is not perpendicular to any of the *X*, *Y* or *Z* axes. Similarly, for the 2p group, the *mm*2 symmetry cannot be detected in the Z x1 X x2 LCS type because the twofold axis is not parallel to any of the *X*, *Y* or *Z* axes (Fig. S1.8).

As a side note, an optimal LCS type is assumed to be the one in which the symmetry can be seen directly, rather than inferred indirectly, and, in the case of the 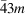, 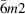 and 3*m* symmetry, where the *Z* axis aligns with the highest-fold symmetry axis (4∥*z*, 6∥*z* or 3∥*z*). Within that type, optimal LCS orientations would be those where all symmetry elements are correctly aligned with the coordinate axes and the equality between symmetry-related electron-density fragments pointing towards neighbors could be correctly observed (Figs. S1.9–S1.13).

#### 2.7.3. Final symmetry assignment

The final level was to compare the symmetries assigned across all LCS types available for a given atom or atom type. If the same symmetry was consistently assigned across all LCS types, that symmetry was accepted as the final symmetry. If different symmetries were obtained for different LCS types, the lowest symmetry was assigned. If the same symmetry point group was found in at least two LCS types but the equality between symmetry-related electron-density fragments pointing towards neighbors was contradictory, the final symmetry was also reduced.

### 2.8. Data analysis

For each subgroup, we computed the global distribution of pseudosymmetries assigned at three levels: (i) for individual LCS orientations but grouped by the LCS type, (ii) for each LCS type, and (iii) as the final pseudosymmetry. These distributions were generated separately for atoms and atom types, and for ref-SC and ref-NSC. In subgroups for which both ref-SC and ref-NSC results were available (4n-C, 3n-N, 3p-N), for every atom and atom type we performed a direct per-pair comparison of pseudosymmetry assignments for ref-SC and ref-NSC, again at the aforementioned three levels. The number and percentage of occurrences of each combination were calculated. The consistency of pseudosymmetry assignment between LCS types was evaluated.

For each atom type, we quantified how many (counts and percentages) atoms belonging to that type were assigned each pseudosymmetry. The pseudosymmetry assigned to each atom type was compared with the first and second most common pseudosymmetries assigned to its constituent atoms, to evaluate the agreement between them. Each case was classified according to whether the pseudosymmetry for atoms was the same, higher or lower than that observed for the atom type, and the number and percentage of each category were calculated.

The final pseudosymmetry assigned to each atom type was also compared with the symmetry specified in the MATTS2021 data bank. For each atom type, the result was classified as identical, higher or lower symmetry relative to the MATTS2021 one and summary statistics (counts and percentages) were produced.

### 2.9. Software

The *bankMaker* utility program from the *DiSCaMB* library (Chodkiewicz *et al.*, 2018 ▸) was used to calculate multipole parameters. All local scripts necessary for preparing, analyzing and visualizing the data and results were made with Python 3.9.13 (Python Software Foundation, 2010 ▸) and Bash 5.2.12 (Free Software Foundation, 2007 ▸). The program *Spyder 5.2.2* (Raybaut, 2009 ▸) with packages: *pandas 1.4.4* (Reback *et al.*, 2022 ▸), *matplotlib 3.5.2* (Caswell *et al.*, 2022 ▸), *seaborn 0.11.2* (Waskom, 2021 ▸), *numpy 1.21.5* (Harris *et al.*, 2020 ▸) and *scikit-learn 1.0.2* (Pedregosa *et al.*, 2011 ▸) was used for programming in Python. Programs *WinXD 1.05* (Volkov *et al.*, 2004 ▸) and *MoleCoolQt 4.8.6* (Hübschle & Dittrich, 2011 ▸) were used for generation and visualization of deformation electron-density maps. All local Python and Bash scripts are available in the repository RepOD (https://doi.org/10.18150/1MEFPJ) (Rybicka *et al.*, 2025 ▸).

## 3. Results and discussion

An analysis of pseudosymmetry assignments to electron densities of atoms and atom types was done separately for each subgroup (see the supporting information S3). Assignments were evaluated at three levels: individual LCS orientations, LCS types and final pseudosymmetry. Differences in the distributions of assigned pseudosymmetries were analyzed both at a global scale and per-pair for each atom and atom type (supporting information S3 and S4).

Across all topological groups, subgroups and levels of analysis, the distribution of assigned electron-density pseudosymmetries was found to depend on several factors: (*a*) the chemical element of the central atom of the atom type within the topological group (see Sections 3.2 and 3.5); (*b*) the level of the analysis (individual LCS orientations, LCS type, final pseudosymmetry) (see Section 3.5); (*c*) the choice of LCS type and orientation (see Sections 3.4 and 3.9); (*d*) whether the pseudosymmetry was assigned for atoms or atom types (see Sections 3.7 and 3.8); (*e*) whether symmetry constraints were applied during refinement or not (see Section 3.12).

Notably, the analysis revealed the need to introduce subgroup-specific zero-value thresholds to approximate 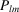 as zero instead of using a global threshold. This approach improved agreement between the pseudosymmetry of atoms and atom types. It also highlighted systematic differences relative to the description of atom types in MATTS2021. Furthermore, it revealed the necessity of introducing substantial changes in key methodological aspects of the MATTS data bank construction and atom-type definitions. These aspects are discussed in detail in the following sections.

### 3.1. Refinement without symmetry constraints improves, in some cases, the fit of the multipole model to the valence electron densities of model molecules altering the values of the multipole model parameters

**Figure 4:**
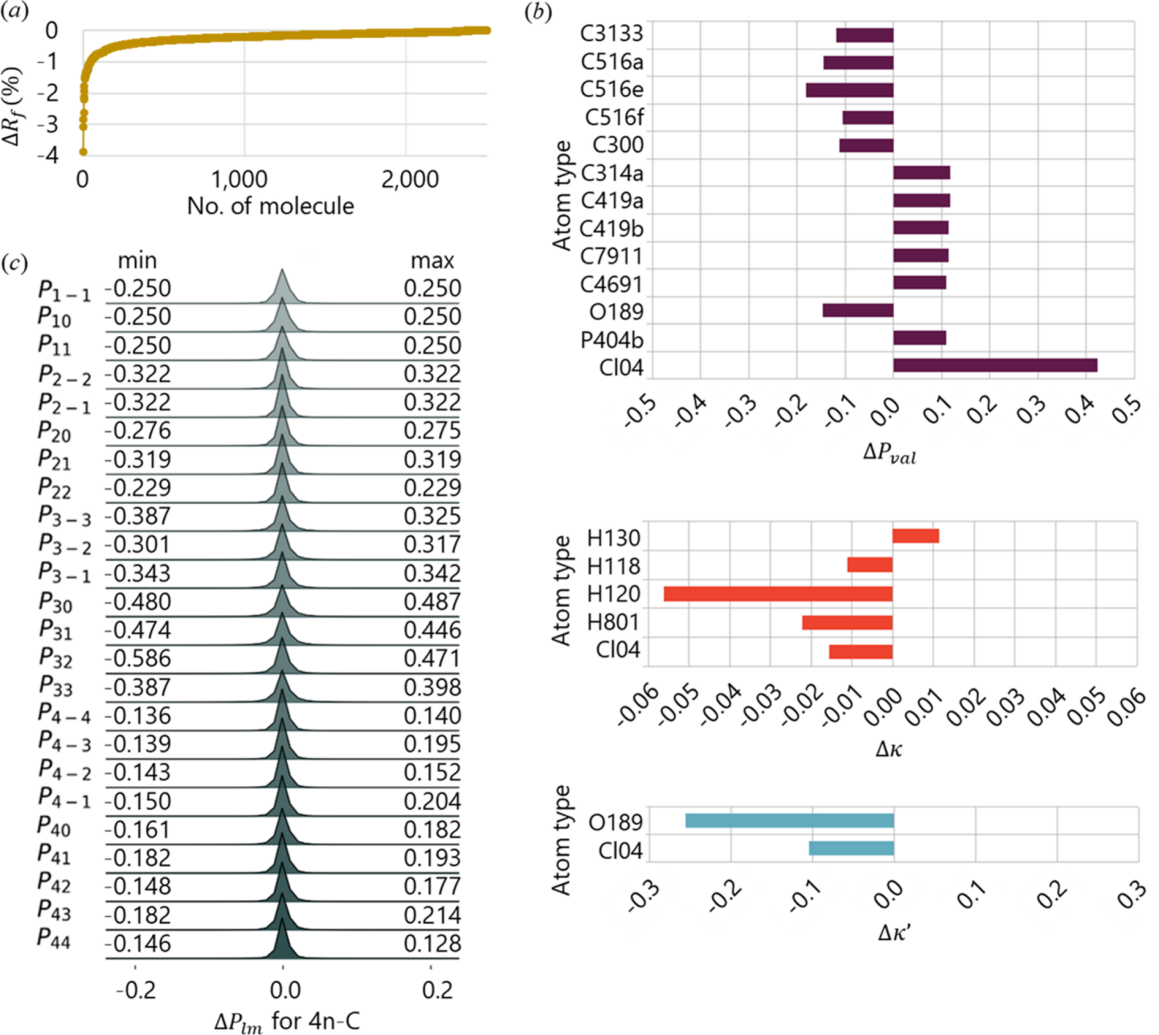
Changes in parameters obtained for ref-NSC and ref-SC: (*a*) 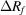 (%) for all 2512 model molecules, (*b*) 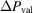, 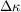 or 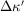 for atom types where the absolute change of at least one of the parameters exceeded the acceptable *ssd* thresholds used in MATTS, (*c*) 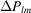 for all 472512 individual LCS orientations of 9844 atoms in the 4n-C subgroup. Differences were calculated as 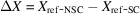, where *X* = 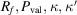 or 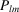.

*Changes in*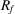 (%). Across the full set of 2512 model molecules, 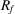 (%) values from ref-NSC were always lower than or equal to those from ref-SC [Fig. 4 ▸(*a*)]. The ten molecules with the largest improvements shared structural similarities such as fused or bridged rings, and frequently contained nitro­gen atoms (Figs. S1.14–S1.15). Although the maximum 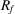 decrease was 3.9%, the median change was smaller (0.2%).

*Changes in*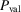, 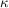
*and*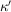. Differences between values of 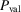, 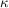 and 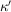 parameters for atom types from ref-NSC and ref-SC were generally small: median of 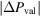 = 0.007, median of |Δκ| = 0.0005 and median of |Δκ′| = 0.0032. However, for 17 out of 651 atom types at least one of 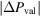 , |Δκ| or |Δκ′| exceeded the acceptable *ssd* thresholds used in MATTS [Fig. 4 ▸(*b*), Table S1.14, Fig. S1.16]. Thirteen atom types showed 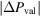 > 0.1: it increased for six atom types and decreased for seven. These atom types shared common features: (*a*) most belonged to the 3p-C and 4n-C subgroups, (*b*) many belonged to four-membered rings, and (*c*) most included less than 20 atoms (Table S1.15). Five atom types showed |Δκ| > 0.01, four of which corresponded to hydrogen types. One of these (H120) represents the hydrogen atom in the hydro­nium cation H_3_O^+^. Only two atom types, both associated with the perchlorate ion ClO_4_^−^, had |Δκ′| > 0.1.

The relationship between changes in 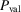, 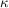 and 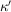 parameters varied across atom types. The most common pattern was an increase in 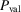 accompanied by decreases in κ and κ′, or the opposite trend (Fig. S1.17).

*Changes in*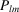. For 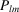, the ref-SC and ref-NSC datasets seem to be largely consistent with each other (Figs. S2.1–S2.8, S2.21–S2.28, S2.38–S2.34), with 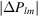 for individual LCS orientations for individual atoms generally close to zero [Fig. 4 ▸(*c*)]. Nevertheless, systematic shifts towards higher or lower 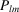 values are observed after releasing symmetry constraints, as reflected by the minimum and maximum 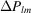 values. In the 4n-C subgroup, 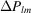 typically falls within the range of −0.05 to 0.05, exceeding these limits in less than 1% of cases. There was a strong positive correlation between the ref-SC and ref-NSC datasets, consistent for all 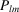 (Fig. S1.18). The correlation plots for the majority of 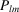 had the data points clustered along the trend line and many had the *R*^2^ value larger than 0.85.

### 3.2. It is possible to determine an objective threshold, using statistical tools, below which the *P_lm_* values can be considered effectively zero

Zero-value thresholds were determined separately for each subgroup using the ref-NSC dataset and their values differ between subgroups (Table 1 ▸). For most subgroups, the zero-value thresholds determined empirically directly from the data are smaller than the arbitrarily chosen 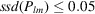 condition used in the MATTS2021 data bank (with the exception of the 4n-P subgroup, which requires a noticeably higher zero-value threshold), indicating that many 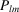 populations are significantly different from zero than previously assumed.

**Figure 5:**
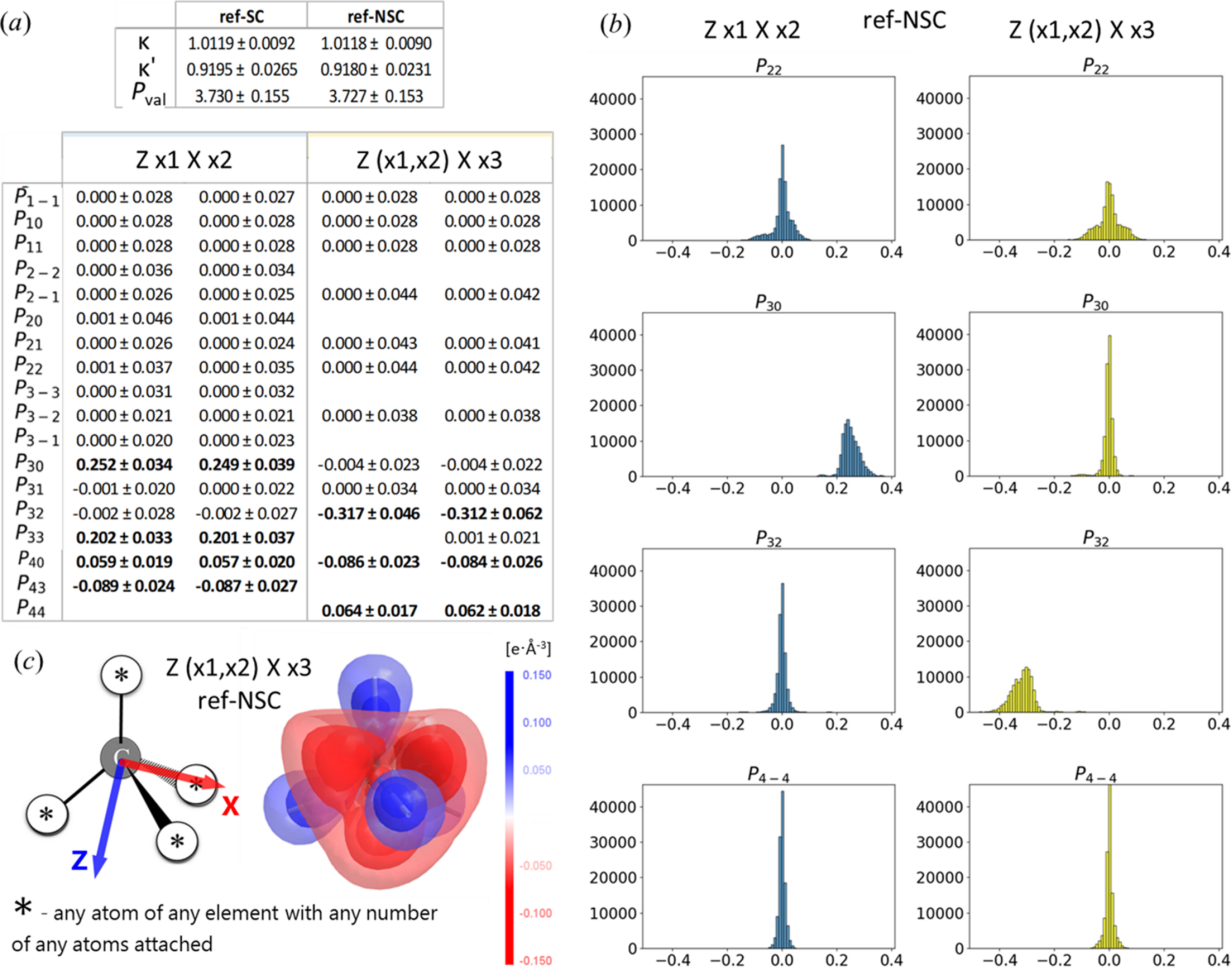
A universal representation of a 4n-C atom: (*a*) mean ± *ssd* of multipole model parameters obtained by averaging over all individual LCS orientations of Z x1 X x2 and Z (x1,x2) X x3 LCS types (118128 in each LCS type) for all 9844 4n-C atoms (ref-SC and ref-NSC, only 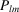 with |mean ± *ssd*| > 0.019 are shown); (*b*) histograms for the chosen 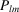 in Z x1 X x2 and Z (x1,x2) X x3 LCS types (ref-NSC); (*c*) a deformation electron-density map plotted using 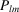 averaged over all 118128 individual LCS orientations of the Z (x1,x2) X x3 LCS type (ref-NSC).

Zero-value thresholds follow the distribution of 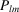 values across subgroups (Figs. S2.1–S2.58). A few 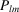 consistently dominated in the given LCS type [see Figs. 5 ▸(*a*)–5 ▸(*b*) for example, all data in Table S1.16], depending on the topological group. In general, heavier elements tend to have higher zero-value thresholds and larger absolute values of the dominant 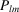 parameters (although not universally). A decrease in the number of first neighbors is followed by a decrease in 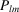 values and zero-value thresholds. In the 4n group, 4n-P and 4n-S have substantially larger 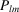 populations than 4n-C and 4n-N. Overall, the means of the most dominant 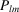 populations follow the trend 4n-P > 4n-S > 4n-N > 4n-C. In the 3p and 3n groups, 3p-C has higher 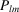 values than 3p-N and 3n-N. Within nitro­gen with three first neighbors, 3n-N has higher 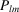 values than 3p-N. In the 2p group, the 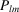 populations increase in the order: 2p-O, 2p-N, 2p-S. Finally, in the 1p group, the 1p-halogens subgroup shows higher 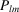 populations than 1p-O.

### 3.3. Averaging within overly universal atom types and across all LCS orientations inflates pseudosymmetry beyond what is supported by the standard deviations of the averaged *P_lm_* values

For each subgroup, the mean 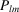 values for a universal atom type were calculated separately in each LCS type as the mean of 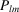 values across all corresponding individual LCS orientations within that LCS type. Many 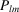 averaged to zero. The pseudosymmetry of the electron density represented by the mean 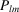 values appeared to be 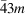 for the Z (x1,x2) X x3 LCS type [Fig. 5 ▸(*c*)]. However, only some 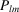 were genuinely zero (their mean values were zero and their *ssd* values were below the 0.019 zero-value threshold established for the 4n-C subgroup). Several 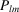 parameters had mean values of 0.000 but *ssd* values exceeding 0.019 and some had clearly non-unimodal distributions of individual values. This finding demonstrated that global averaging over the entire 4n-C subgroup and all LCS orientations within a given LCS type was too coarse, highlighting the need to analyze individual LCS orientations independently and to cluster atoms by atom types that are more specific than the broad universal 4n-C subgroup definition.

The same was observed in other subgroups, where averaging 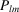 across all LCS orientations within LCS type for an overly general atom type (universal atom-type definitions) led to the assignment of the highest possible pseudosymmetry in each LCS type (Table S1.17). It is noteworthy that the pseudosymmetries assigned for such a universal 3n-N atom-type definition were: *m*(*m*⊥*y*) in Z x1 X x2, *mm*2(2∥*x*) in X (x1,x2) Y x1, *mm*2(2∥*z*) in Z (x1,x2) Y x1 and 3*m*(*m*⊥*y*) in Z (x1,x2,x3) X x1 LCS type (Table S1.17). Thus, the X (x1,x2) Y x1 and Z (x1,x2) Y x1 are clearly non-optimal for 3n – they show symmetry that is unexpected for this group.

### 3.4. An incorrect LCS type may artificially increase the assigned atom-type symmetry and cause the selective loss of important aspherical features of the electron density

**Figure 6:**
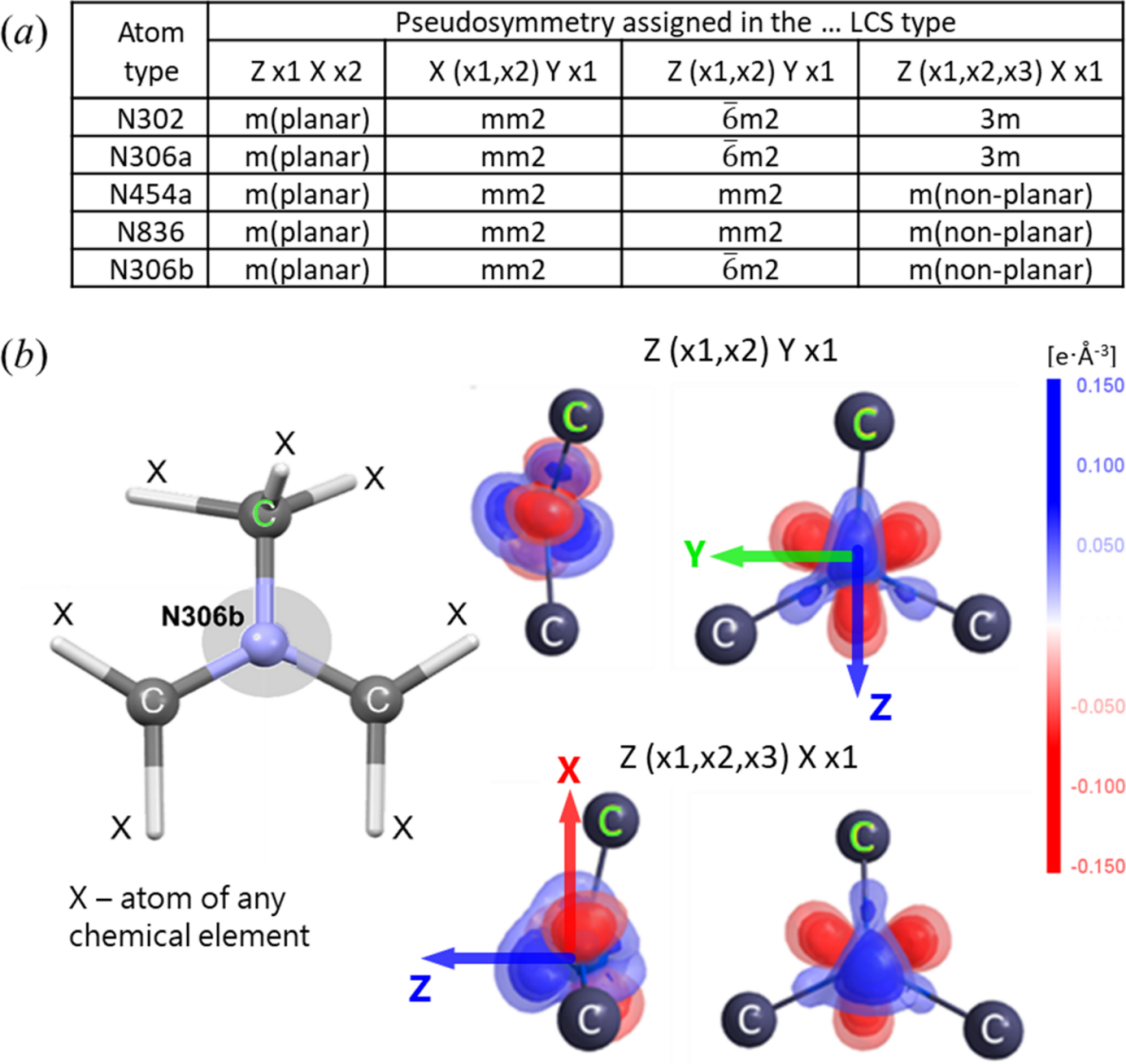
(*a*) Comparison of pseudosymmetry assigned for 3n-N atom types where any other LCS type than Z (x1,x2,x3) X x1 leads to an assignment of incorrect planar pseudosymmetry; (*b*) graphical visualization of the N306b atom type and its deformation electron-density maps in Z (x1,x2) Y x1 and Z (x1,x2,x3) X x1 LCS types.

This effect is evident for the 3n-N subgroup. In individual LCS orientations of the Z x1 X x2, X (x1,x2) Y x1 and Z (x1,x2) Y x1 LCS types, a significant fraction of 3n-N atom types were assigned pseudosymmetries [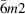, *mm*2, *m*(planar)], which are unexpected for this subgroup [Fig. 6 ▸(*a*), Table S4.5]. This situation happens because in these three LCS types there is no control over how the second axis of the LCS will be oriented with respect to the lone electron pair of the central nitro­gen atom and the third first neighbor. During the averaging of nitro­gen atoms belonging to the 3n-N subgroup expressed in these LCS types, the orientation of the lone electron pair was not preserved. Atoms with the lone pair oriented in the opposite directions were averaged, leading to the appearance of artificial planar pseudosymmetries mixing the electron density of the lone pair with the electron density directed to one of the neighboring atoms.

In contrast, when using the Z (x1,x2,x3) X x1 LCS type, where the *Z* axis is aligned along the lone electron pair and all three neighbors are involved, only pseudosymmetries expected for the 3n-N group appear for atom types [3*m*, *m*(non-planar), 1]. This LCS type is necessary to properly average multipole model parameters in the 3n group and assign the final pseudosymmetry for atom types. The other three LCS types are also suitable, but only in cases when x1, x2 and x3 are topologically distinguishable in the definition of the atom type, and the chiral option is applied. This option explicitly takes into account the handedness of the group, using the positions of the three first neighbors to determine the absolute configuration (*R* or *S*) for atom types with chiral centers. As a result, the averaging of multipole populations 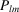 is performed in a chirality-consistent reference frame, preserving the handedness of the electron density and ensuring sensitivity to the spatial position of features such as lone electron pairs.

### 3.5. LCS-based *post-hoc* analysis reveals both expected and unexpected, but chemically meaningful, pseudosymmetry

The assignment of pseudosymmetry can be automated *post-hoc* by applying a zero-value threshold to the 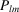 parameters across a series of different LCS orientations. This approach often effectively reconstructs symmetries of atom types that were previously imposed, demonstrating that the procedure is complete and consistent with expectations, without the need to enforce symmetry *a priori*. At the same time, the observed unexpected pseudosymmetries provide valuable information, either highlighting procedural limitations of the construction process of the MATTS data bank or revealing chemically meaningful effects that would not be known if only the topology was considered.

The distributions of pseudosymmetries assigned to electron densities depend on multiple factors, including the topological group, chemical element, LCS type and orientation, and level of analysis (individual orientations, LCS types, final assignment), as well as on whether atoms or atom types are considered. Differences between atoms and atom types, as well as between LCS types, are observed, including variations in the prevalence of higher versus lower pseudosymmetries, consistency of assignments and occurrence of unexpected pseudosymmetries. Detailed results for each subgroup of the ref-NSC dataset are provided in the supporting information S3. For a comparison between ref-SC and ref-NSC for 4n-C, 3n-N and 3p-N see Section 3.12.

One case worth highlighting is the 1p group (Fig. S3.18). The pseudosymmetries observed for 1p-2 atom types generally agree well with the expected ones. Unexpected pseudosymmetries are rare and limited to two cases among 1p-O-2 types, namely O122f (pseudosymmetry cyl) and O372 (pseudosymmetry 1). Contrastingly, for 1p-3 atom types the only unexpected observed symmetry is the cylindrical one, yet it occurs relatively frequently, accounting for 42.9% (six out of 14) of 1p-O-3 types and 60.0% (three out of five) of 1p-halogens-3 types. However, it should be remembered that oxygen atoms have two lone electron pairs, while halogens have three. Interestingly, their expected shapes are not clearly resolved in the deformation density maps (Fig. S3.18). Instead, the observed shapes appear to be effectively dominated by the arrangement of second neighbors, indicating that lone pairs play a secondary role in determining the apparent pseudosymmetry of the electron density. The occurrence of unexpected pseudosymmetries in the 1p group can likely be attributed to several factors, including inequality of lone electron pairs, alignment of lone electron pairs with second neighbors or lack thereof, mutual arrangement of lone electron pairs and the plane of the first neighbor, partial averaging of electron density which may lead to apparent cylindrical symmetry, and possible electron delocalization effects, for example in π systems. Given this complexity, the interpretation of pseudosymmetry in the 1p group should be approached with caution, and deviations from expected symmetry are best regarded as a natural consequence of structural and electronic factors.

### 3.6. Pseudosymmetries assigned solely from *P_lm_* values allow the establishment of a more objective and appropriate planarity threshold for atoms with three first neighbors

**Figure 7:**
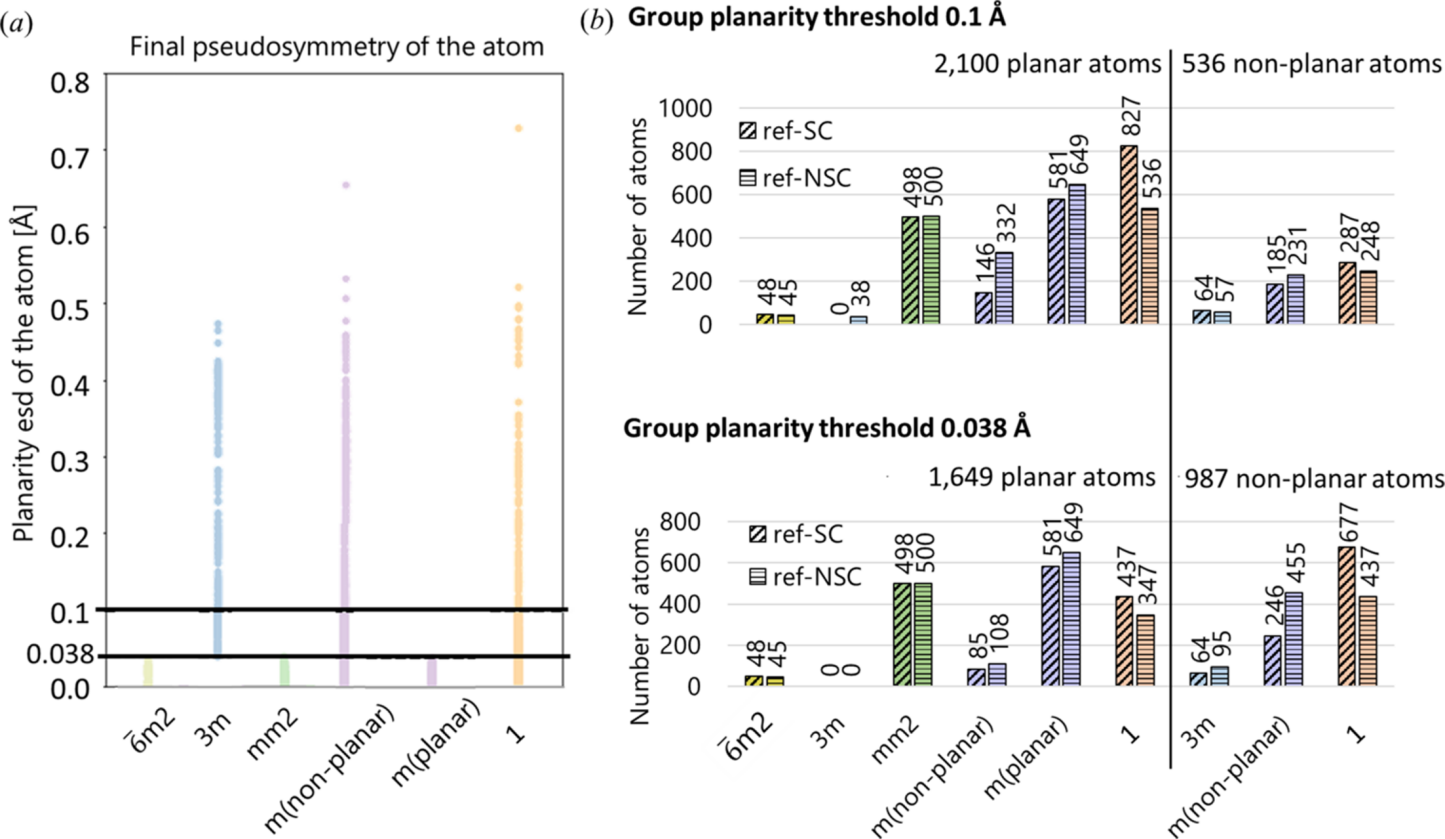
(*a*) Pseudosymmetry of electron density assigned for all 3n-N and 3p-N atoms versus their ‘planarity e.s.d.’ parameter (ref-NSC); (*b*) division of nitro­gen atoms into 3n-N and 3p-N subgroups and a comparison of pseudosymmetry of electron density assigned for them with group planarity thresholds 0.1 Å and 0.038 Å (ref-SC and ref-NSC).

One possible reason for the presence of unexpected pseudo­symmetries in the 3p group could be that the group planarity threshold was set too high, and atoms were therefore not sufficiently planar. This prompted a systematic analysis of the final assigned pseudosymmetries of the electron densities versus the ‘planarity e.s.d.’ for each 3n-N and 3p-N atom [Fig. 7 ▸(*a*)]. The analysis revealed that pseudosymmetries expected in the 3p group [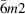, *mm*2, *m*(planar)] ceased well before the group planarity threshold of 0.1 Å. The highest ‘planarity e.s.d.’ values for 3p-N atoms with *mm*2 and 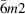 pseudosymmetries were 0.037 Å and 0.033 Å, respectively, while the lowest ‘planarity e.s.d.’ of an 3p-N atom with assigned 3*m* pseudosymmetry was 0.039 Å. This supports a revised group planarity threshold of 0.038 Å as a more accurate estimation for nitro­gen atoms with three neighbors.

Using 0.038 Å instead of 0.1 Å as the group planarity threshold, 451 atoms (17.1%) switched from 3p-N to 3n-N [Fig. 7 ▸(*b*)]. Following that, the percentage of unexpected *m*(non-planar) and 1 pseudosymmetries significantly decreased in 3p-N, and the unexpected 3*m* pseudosymmetry observed in ref-NSC disappeared completely in 3p-N.

Some 3p-N atoms retained 1 or *m*(non-planar) pseudosymmetries despite having a very low ‘planarity e.s.d.’ (Table S4.7). Examination of the molecules showed that many of these atoms were topologically planar but most probably they exhibited electron densities polarized through space, with asymmetry above and below the plane, explaining the elevated 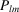 values.

The confusion between planar and non-planar character is also visible at the level of atom types. Two 3p-N atom types (N333b and N338) had the unexpected for this subgroup *m*(non-planar) pseudosymmetry assigned as the final one for the atom type and also as the most common one for their atoms (Table S4.7). Atom type N333b, with three bulky *sp*^3^ carbon neighbors, from the chemical point of view should be non-planar but was classified as planar within the applied planarity threshold 0.1 Å. Atom type N338, with two bulky *sp*^3^ carbon neighbors and one nitro­gen of any kind, again should be chemically non-planar. Their ‘planarity e.s.d.’, calculated as an average of the ‘planarity e.s.d.’ of atoms that belong to them, were 0.051 Å for N333b and 0.041 Å for N338. Applying a group planarity threshold of 0.038 Å instead of 0.1 Å would classify them as 3n-N, not 3p-N.

Similar analysis for 3p-C revealed that this subgroup also would benefit from adjusting the group planarity threshold (Fig. S1.19). The highest ‘planarity e.s.d.’ values for 3p-C atoms with each pseudosymmetry higher than 1 were: 0.012 Å for 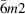, 0.014 Å for *mm*2 and 0.017 Å for *m*. This suggests the group planarity threshold of *circa* 0.017 Å to be a more accurate estimation for carbon atoms with three neighbors. The proposed group planarity threshold for carbon is different than for nitro­gen, which means that the planarity estimation depends on the chemical element.

### 3.7. The pseudosymmetry of an atom-type electron density does not always represent the actual symmetry of the atoms from which that atom type is defined

The comparison between the final pseudosymmetry assigned to each atom type and the most common final pseudosymmetries assigned to individual atoms that belong to said atom type (Table 2 ▸, Tables S4.1–S4.12) highlighted the challenges in transferring the electron-density distribution of atoms to the more generalized description presented by an atom type. The 1p group was skipped due to a common presence of unexpected pseudosymmetries on individual LCS orientations for atoms and difficulties in assigning the final pseudosymmetry for atoms (supporting information S3). In many cases, the most common pseudosymmetry among atoms did not match the pseudosymmetry assigned to the atom type (Tables S4.1–S4.12). In extreme situations, all atoms (100.0%) within a given atom type exhibited a different pseudosymmetry (typically lower) than the pseudosymmetry assigned to the type. Moreover, identifying the single dominant pseudosymmetry among individual atoms was not always straightforward, especially when two pseudosymmetries occurred with nearly identical frequency (*e.g.* for atom type C404a, pseudosymmetry 1 was assigned to 38.4% of atoms and *mm*2 to 38.0%). Thus the second most common final pseudosymmetry of individual atoms was included in the analysis.

In more than half of the subgroups, the most common pseudosymmetry assigned to the individual atoms of the atom type is often lower than the pseudosymmetry assigned to the atom type itself (Table 2 ▸, Tables S4.1–S4.12). This is reflected in the percentage ratio ‘the same: lower: higher’, where the dominance of ‘lower’ over ‘the same’ is most pronounced for 4n-C, 4n-N, 3n-N, 3p-C and 3p-N.

Overall, for 316 out of 548 (57.7%) atom types the first most common pseudosymmetry for atoms was lower than that assigned to the atom type, while for five (<1%) it is higher (Tables S4.1–S4.12). The latter includes atom types: P409c (atoms: 3*m*, atom type: 1), C352b and C387 (atoms: *m*, atom type: 1), C332c (atoms: *mm*2, atom type: *m*) and N3593 [atoms: *m*(non-planar), atom type: 1]. Each of these atom types includes less than ten atoms.

The situation improves when the second most common pseudosymmetry among atoms was taken into account, with the largest improvement observed for the 3p-C subgroup. After application of more appropriate planarity thresholds, the situation most probably would improve. The worst transferability of electron-density pseudosymmetry was observed for 4n-C and 4n-N. The 4n-N subgroup contains only nine atom types, and such low variability limits deeper understanding of possible reasons. We therefore focused further mostly on the 4n-C subgroup.

Averaging inevitably conceals details seen on the atomic level, and as a result a balance must be found between preserving the real distribution of the electron density of atoms and obtaining stable, transferable parameters for atom types. The above analysis demonstrated that averaging may not preserve the individual pseudosymmetry of electron density of each atom and tends to raise the pseudosymmetry for the atom type. If this were the case, it should have led to an increase in the *ssd* value for some 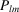 of the atom type. Until now, when deciding which 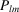 of a given atom type is effectively zero, only the value of that 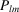 was considered. However, a second condition could be added in the case of atom types and the *ssd* of that 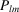 should also be required to be lower than the zero-value threshold. This would then change the pseudosymmetry assigned to the analyzed atom types and help achieve consistency between the pseudosymmetry of the atom type and the atoms it is meant to represent, particularly in subgroups where this problem is most apparent, *i.e.* 4n-C, 4n-N, 3n-N, 3p-C and 3p-N.

Applying this stricter rule to 4n-C shows that (*a*) 129 atom types (97.7%) would then have pseudosymmetry consistent with the first or second most common pseudosymmetry of atoms within that type, and (*b*) 117 atom types (88.6%) would end up with pseudosymmetry 1 as the final one (Table S4.1). However, such an outcome, where almost all atom types have pseudosymmetry 1, is unacceptable from the perspective of the data bank. This would require a change in the definition of atom types so that the symmetry resulting from the topology of the atom type and the pseudosymmetry of the electron density are consistent. To achieve the pseudosymmetry 1, the topology definition would have to be much more detailed, which is unrealistic.

Therefore, a compromise is necessary. Continuing with the example of the 4n-C subgroup, if we apply a less strict criterion for 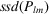 and assume that 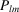 is effectively zero only when (*a*) 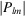 is smaller than the zero-value threshold 0.019 and (*b*) 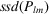 is smaller than 3× the zero-value threshold (3 × 0.019 = 0.056), we can observe that only 52 types have the final pseudosymmetry 1 and for all of them it matches the most common symmetry assigned to atoms (Table S4.1). The overall agreement between the atom-type symmetry and the first most common symmetry among the atoms increased to 53.0%.

For the rest of the problematic subgroups, applying the ‘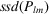 smaller than 1× zero-value threshold’ criterion (Tables S4.5–S4.7) was not enough to substantially improve agreement between the pseudosymmetry assigned to atom type and atoms, similarly as for 4n-C. However, increasing the criterion to 2× the zero-value threshold increased agreement to *circa* 70–85%, depending on the subgroup, while a 3× the zero-value threshold criterion further raised agreement to approximately 90–96%. This further confirms that using conditions addressing 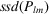 is necessary when deciding when 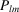 can effectively be zero.

### 3.8. Some atom types, as defined in this work, exhibit systematically higher electron-density symmetry than the atoms they represent, indicating the need to adjust the definition of type topology

As already established, the electron-density pseudosymmetry obtained for an atom type was often systematically higher than the most common symmetry observed for the individual atoms it represents, which is an effect arising from too excessive averaging.

Reintroducing distance-based sorting of neighbors and including this feature in LCS assignment provides a better definition of atom type and recovers atom-type symmetry that correctly represents the symmetries of atoms. For example, 3p-C atom types C008, C340a, C340e, C507, C331, C561 and C330 show that although their atom-type pseudosymmetry is *mm*2, which matches the topology, many atoms in these types have *m* pseudosymmetry assigned (47–71%, depending on the atom type) (Table S4.6).

Another situation was also observed: some atom types were assigned a higher pseudosymmetry than implied by the topology, while the atoms themselves retained the topology-consistent pseudosymmetry. For instance, several 4n-C types, such as C401 (topology: 3*m*, atoms: 3*m*, atom type: 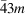), C431, C450b, C460, C7852 and C792 (topology: 1, atoms: 1, atom type: *m*), illustrate overestimation of symmetry introduced during averaging (Table S4.1). Similar effects were found for atom types in other subgroups, *e.g.* P404b in 4n-P (topology: *m*, atoms: *m*, atom type: *mm*2) (Table S4.3). There are two patterns that are most common for all the above cases: either the number of atoms belonging to the given atom type was relatively small or there were carbon and hydrogen atoms among the first neighbors.

A particularly interesting pattern was sometimes observed for atom types when their first neighbors included at least one carbon and one hydrogen atoms. For example, 16 out of 73 such atom types in the 4n-C subgroup had pseudosymmetries higher than expected from the topology that implied the similarity between the electron-density fragments pointing from the central carbon atom towards neighboring carbon and hydrogen atoms (Table S1.18). Examples include C403a (first neighbors: CCCH, atom type: 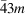, atoms: 1), C403b, C998 and C999 (first neighbors: CCCH, atom type: *mm*2, atoms: 1) and C405 (first neighbors: CCHH, atom type: 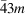, atoms: 1). Sporadically, there was an agreement between atoms and atom-type pseudosymmetry, *e.g.* for C838c (first neighbors: CCClH, atom type: 3*m*, atoms: 3*m*).

### 3.9. For each topological group (4n, 3p *etc*.) an optimal LCS type can be proposed which, after restoring the distance sorting, best reproduces the atom’s pseudosymmetry after averaging within the atom type

As established in the *Introduction*, the optimal LCS balances accurate representation of electron-density pseudosymmetry, chemical group topology, transferability and computational efficiency. For each topological group (4n, 3n, 3p, 2p, 1p), such an optimal LCS type can be proposed. In addition, for each symmetry, the selection of specific individual LCS orientations within the optimal LCS type is required to correctly reflect the symmetry and equivalence of electron-density fragments pointing towards neighbors (Figs. S1.9–S1.13).

(i) 4n – Z (x1,x2) X x3. The Z (x1,x2) X x3 LCS type is the most optimal as it (*a*) provides the most balanced distribution of pseudosymmetries, (*b*) has the fewest number of dominant 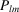 (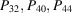), minimizing parameters for linear combination (Table S1.16), and (*c*) allows direct observation of the highest pseudosymmetry 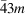. The 3*m* pseudosymmetry was favored in Z x1 X x2 and Z (x1,x2,x3) X x1, while *mm*2 appeared mainly in X (x1,x2) Y x1. Individual differences in pseudosymmetry ratios across LCS types were similar between 4n-C, 4n-N, 4n-P and 4n-S. The highest percentage of atoms with pseudosymmetry 1 occurred in the Z (x1,x2) X x3 LCS type. The Z x1 X x2 and Z (x1,x2,x3) X x1 LCS types had very similar pseudosymmetry distributions, whereas X (x1,x2) Y x1 consistently overestimated symmetry compared with other LCS types.

(ii) 3n – Z (x1,x2,x3) X x1. For reasons that have already been discussed, only the Z (x1,x2,x3) X x1 LCS type reproduces the pseudosymmetry adequately. Additionally, it has the lowest number of dominant 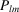 (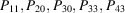) among LCS types for 3n (Table S1.16).

(iii) 3p – X (x1,x2) Y x1. For 3p-C, differences in the distribution of pseudosymmetries between LCS types were minimal. For 3p-N, the X (x1,x2) Y x1 and Z (x1,x2) Y x1 LCS types provide similar distributions, whereas Z x1 X x2 favors lower symmetries. The number of dominant 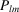 is the lowest for X (x1,x2) Y x1, only three 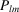 dominate: 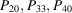 (Table S1.16).

(iv) 2p – X (x1,x2) Y x1 or Z (x1,x2) Y x1. The Z x1 X x2 LCS type is unsuitable because the *mm*2 pseudosymmetry cannot be observed either directly or indirectly. It also has many, at least ten, dominant 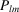, depending on the subgroup. The X (x1,x2) Y x1 and Z (x1,x2) Y x1 LCS types give nearly identical distributions and match the final atom pseudosymmetry well, with similar numbers of dominant 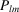 (Table S1.16).

(v) 1p – Z x1 X x2. It was the only considered LCS type.

Our work follows the concept of optimal LCS necessary for a data bank construction introduced by Domagała & Jelsch (2008 ▸), who proposed specific symmetry-aligned LCS types for atoms grouped by coordination and local geometry. In their scheme, for tetra-coordinated atoms (4n), a limited set of LCSs based on two or three neighbors [X x1 Y x2, X (x1,x2) Y x1, Z x1 X x2, Z (x1,x2) Y x1, or Z (x1,x2) Y x3] is selected according to local symmetry; for three neighbors, non-planar (3n) systems employ Z (x1,x2) X x1 or Z (x1,x2,x3) X x1 depending on the presence of threefold symmetry, while planar systems (3p) use X x1 Y x2 or X (x1,x2) Y x1 with the *Z* axis perpendicular to the plane; for two neighbors, Z x1 X x2 axes are applied to linear groups (2x) and X (x1,x2) Y x1 to non-linear ones (2p); and Z x1 X x2 axes are used for atoms with one neighbor (1p). Several of these optimal LCS types [Z x1 X x2, X (x1,x2) Y x1 and Z (x1,x2,x3) X x1] are directly equivalent to those analyzed in our work, confirming consistency with the principles underlying the transferable data bank development. Consistent with their examples, we likewise favor the Z (x1,x2,x3) X x1 LCS type for N *sp*^3^ atoms and the LCS type with the first axis defined between the two H atoms for oxygen in water representing the 2p group. However, we systematically explored multiple LCS types within each group while the LCS choice was predetermined based on assumed topological symmetry and number of neighbors.

### 3.10. Automation makes it possible to identify cases where current atom-type definitions require corrections to the symmetry defined in them and reduces the risk of human errors

The final pseudosymmetry of the electron density of atom types from the ref-NSC was compared with the symmetry from the MATTS2021 data bank. It should be noted that some atom types in MATTS were defined using a ‘safe’ symmetry assignment (*i.e.* the lowest possible for the topological group), when the appropriate symmetry could not be determined with sufficient confidence.

**Figure 8:**
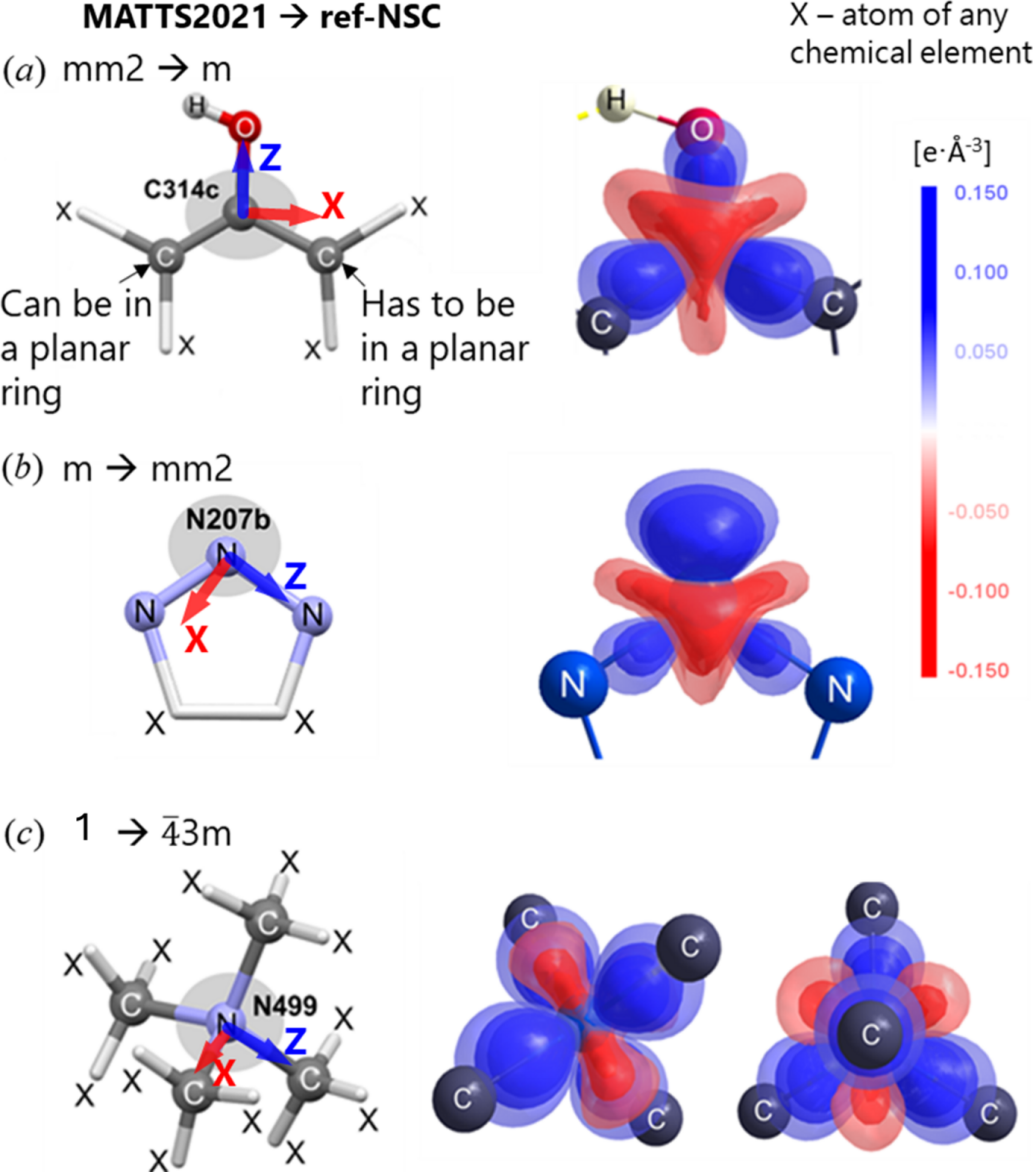
Graphical visualization of the C314c (*a*), N207b (*b*) and N499 (*c*) atom types and their deformation electron-density maps in the Z x1 X x2 LCS type.

Automated pseudosymmetry analysis allowed the identification of atom types whose symmetry definitions in the MATTS2021 data bank require revision. Changes were observed across all subgroups, with both increases and decreases in pseudosymmetry [Figs. 8 ▸(*a*)–8 ▸(*b*)]. Subgroups with mostly unchanged symmetry were 3p-C (70.5%), 2p-O (86.0%), 2p-N (77.8%) and 2p-S (78.6%). The 1p-O and 1p-halogen atom types showed substantial increases in symmetry (Tables S4.11–S4.12). In particular, atom types originally defined with symmetry *m* frequently changed, either increasing to higher symmetry (*e.g.mm*2) or decreasing to 1. For further details see the supporting information S3.

Automation of pseudosymmetry assignment also reduces the risk of human errors in atom-type definitions. For example, in the MATTS2021 data bank, two 3p-C atom types (C522 and C310) were originally assigned an incorrect 3*m* symmetry. Our automated analysis revealed that the pseudosymmetries *mm*2 and *m*, respectively, provide a more accurate description of their electron density. Similarly, two 4n-N atom types (N455 and N499), defined with symmetry 1 in MATTS2021, were found to have 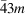 pseudosymmetry that aligns correctly with their topology [Fig. 8 ▸(*c*)].

Overall, these results highlighted that current definitions of atom types sometimes over- or underestimate symmetry, emphasizing the need for refined definitions and automated symmetry assignment in future data bank versions. Automated symmetry assignment can detect mistakes in existing data banks, ensuring that atom-type definitions better reflect the apparent symmetry of electron density.

### 3.11. The improved LCS definitions result in the same LCS type spanning a larger number of atom types, enabling the use of machine learning methods to optimize existing topology definitions and to introduce new ones based on classifications performed on *P_lm_*

The revised LCS definitions substantially increase the consistency of LCS types across atom types. In MATTS2021, nine different LCS types were used, with multiple LCS types appearing within the same topological group (Table S1.3). In contrast, the optimized LCS scheme proposed in this work establishes a single LCS type optimal for each group, producing a far more cohesive and uniform representation.

This consistency has two major consequences. Firstly, using the same LCS type across a larger number of atom types ensures that the corresponding 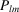 values are directly comparable, enabling more reliable averaging, symmetry assignment and transferability of parameters. Secondly, the unified LCS representation enables the use of machine learning methods to optimize existing atom-type definitions and to introduce new ones through clustering or classification performed on 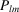, facilitating future extensions of the data bank.

### 3.12. Removing symmetry constraints enhances pseudosymmetries assigned *post-hoc*, makes them more consistent among various LCS types, and improves transferability of electron-density pseudosymmetry from atoms to atom types

Removing symmetry constraints during refinement had a clear, non-negligible impact on the pseudosymmetries assigned at each level for atoms and atom types in the three analyzed subgroups (4n-C, 3p-N, 3n-N). The effects were analyzed at each level of pseudosymmetry assignment (LCS orientations, LCS types, final) for both atoms and atom types.

*Influence on atoms*. Releasing symmetry constraints altered the per-pair pseudosymmetry assignment for 17–31% of individual LCS orientations, depending on the subgroup (21.8% for 4n-C, 16.9% for 3n-N and 30.5% for 3p-N). Changes occurred in both directions, towards lower pseudosymmetries (12.7% for 4n-C, 7.6% for 3n-N, 17.2% for 3p-N) and unexpectedly towards higher (7.9% for 4n-C, 9.3% for 3n-N, 12.6% for 3p-N) pseudosymmetries. The dominant trends differed by subgroup (Tables S1.19–S1.21). Most noticeably, unexpected cylindrical symmetry assigned to 45 individual LCS orientations of 4n-C atoms in ref-SC was no longer present in ref-NSC.

After all LCS orientations within given LCS types were considered together, many atoms changed their assigned pseudosymmetry depending on the subgroup and LCS type (35–44% in 4n-C, 32–36% in 3n-N, 65–75% in 3p-N, see Tables S1.22–S1.24). The largest percentage of changes was observed in the X (x1,x2) Y x1 LCS type for all three subgroups (4n-C 43.8%, 3n-N 35.6% and 3p-N 75.1%). Frequent shifts both towards lower and higher pseudosymmetries occurred (on average: in 4n-C 27.8% to lower and 11.6% to higher, in 3n-N 17.7% to lower and 15.7% to higher, in 3p-N 29.9% to lower and 37.3% to higher). For 3p-N, transitions between expected *m*(planar) and unexpected *m*(non-planar) occurred in both directions, although shifts to *m*(non-planar) were two times more frequent (∼4%) than to *m*(planar) (∼2%), which further corresponds to issues with the group planarity threshold presented in Section 3.6.

**Figure 9:**
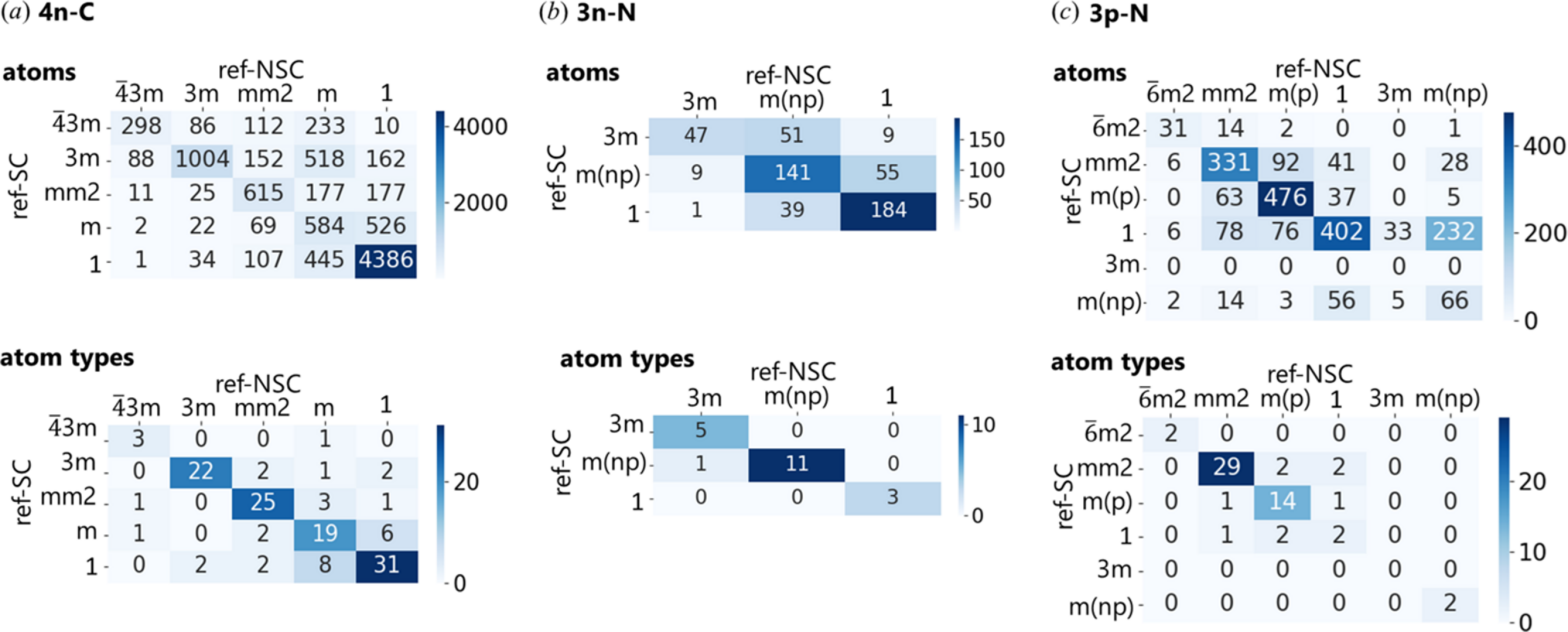
Heatmaps comparing final pseudosymmetry of electron density between ref-SC and ref-NSC assigned for atoms (top) and atom types (bottom) belonging to the 4n-C (*a*), 3n-N (*b*) and 3p-N (*c*) subgroups. Identical pseudosymmetries between ref-SC and ref-NSC datasets are shown on the diagonal. Off-diagonal numbers represent changes: the row indicates the pseudosymmetry in ref-SC, and the column indicates the pseudosymmetry in ref-NSC. Note that the matrix is not symmetric, since each off-diagonal entry represents a distinct comparison. Abbreviations: *m*(p) = *m*(planar), *m*(np) = *m*(non-planar).

Releasing symmetry constraints altered the assignment of final pseudosymmetry for 30.4% of 4n-C, 35.4% of 3n-N and 37.8% of 3p-N atoms [Figs. 9 ▸(*a*)–9 ▸(*c*), top]. The dominant transitions were from higher to lower pseudosymmetry, although some increases also occurred. For 4n-C atoms, the most frequent changes were *m* → 1, 3*m* → *m* and 1 → *m*. For 3n-N atoms, the most frequent changes were *m*(non-planar) → 1 or 3*m* → *m*(non-planar). For 3p-N atoms, the most frequent changes were 1 → *m*(non-planar) and many atoms switched between *m*(planar) and *m*(non-planar). Between ref-SC and ref-NSC, consistency of pseudosymmetry assignment across different LCS types increased from 46.8% to 49.6% for 4n-C (Fig. S3.1c left), from 53.0% to 53.4% for 3n-N (Fig. S3.7c left), and from 67.9% to 70.2% for 3p-N atoms (Fig. S3.11*c* left). This increase in consistency is desirable because inconsistent assignment between LCS types leads to a lowered final pseudosymmetry for such atoms.

The effects of removing symmetry constraints observed for pseudosymmetry assignment for atoms carry over to atom types. Changes for atoms are systematic enough that they also produce non-random shifts for atom types.

*Influence on atom types*. Across subgroups, releasing symmetry constraints altered the per-pair pseudosymmetry assignments for 12–18% of individual LCS orientations for atom types (11.8% for 4n-C, 18.3% for 3n-N, 16.9% for 3p-N). Although changes occurred in both directions (increase or decrease of pseudosymmetry), the dominant transitions were specific for a given subgroup (Tables S1.25–S1.27).

At the level of LCS types, 22–25% of atom types changed pseudosymmetry in 4n-C, 12–20% in 3p-N, and up to 65% in 3n-N, depending strongly on the LCS type (Tables S1.28–S1.30). In Z x1 X x2, 35% of 3n-N atom types changed their pseudosymmetry after releasing constraints, whereas in X (x1,x2) Y x1 and Z (x1,x2) Y x1 60–65% of 3n-N atom types changed, with frequent switches among all pseudosymmetries (Table S1.26). In contrast, in the Z (x1,x2,x3) X x1 LCS type only 5% of 3n-N atom types had a change of pseudosymmetry between ref-SC and ref-NSC. This means that the Z (x1,x2,x3) X x1 LCS type is the most stable choice for representing the pseudosymmetry for the 3n group.

The number of atom types with consistent pseudosymmetry assignments across all LCS types increased from 57 in ref-SC to 59 in ref-NSC for 4n-C (Fig. S3.1c, right) and decreased from 44 in ref-SC to 39 in ref-NSC for 3p-N (Fig. S3.11c, right). Because the proper final pseudosymmetry of 3n-N atom types can only be found in the Z (x1,x2,x3) X x1 LCS type, the consistency of pseudosymmetry assignments across all LCS types between ref-SC and ref-NSC for 3n-N cannot be evaluated.

5–24% of atom types changed their final assignment of pseudosymmetry (24.2% 4n-C, 5.0% 3n-N, 19.6% 3p-N) with shifts dominated by reductions from higher to lower symmetry [Figs. 9 ▸(*a*)–9 ▸(*c*), bottom, Tables S1.31–S1.33]. Releasing symmetry constraints reduced or eliminated unexpected pseudosymmetries (*e.g.* cylindrical symmetry in 4n-C) and revealed limitations related to the group planarity threshold in the 3p group.

Changes in pseudosymmetry assignment between ref-SC and ref-NSC are more pronounced for atoms than for atom types. Symmetry constraint removal reduces or eliminates unexpected pseudosymmetries, improves consistency of assignments across LCS types in most cases, and highlights issues related to group planarity for the 3p group. Overall, these results demonstrate that the refinement strategy has a direct impact on the reliability and transferability of electron-density pseudosymmetry, and refinement without symmetry constraints is recommended for further development of the MATTS data bank.

## 4. Related literature

The following references are cited in the supporting information: Akriche & Rzaigui (2001 ▸), Bentrude *et al.* (1986 ▸), Czapla *et al.* (1999 ▸), Hartmann *et al.* (1999 ▸), Haneef *et al.* (1985 ▸), Jones & Bubenitschek (2008 ▸), Kutter *et al.* (2018 ▸), Murthy *et al.* (2003 ▸), Pearlman & Kim (1985 ▸), Pinkerton & Schwarzenbach (1978 ▸), Tsuno *et al.* (2003 ▸), Urzhumtsev (1991 ▸).

## 5. Conclusions

In this work, we studied the interplay between atom or atom-type electron-density pseudosymmetry and the symmetry inferred from topology, the choice of LCS type and orientation, and the averaging of 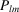 values of individual atoms to obtain atom-type electron density. We have shown that, using symmetry-unconstrained multipole model refinements, which generally improves consistency among all studied factors, it is possible to objectively assign pseudosymmetry to atoms and atom types by combining (i) a statistically determined zero threshold below which 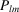 values are considered effectively zero, (ii) a carefully designed set of chemically meaningful LCS orientations, and (iii) Kurki-Suonio symmetry selection rules that identify symmetry-unexpected 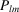. Chemical context, central-atom element, number and identity of first neighbors, and the planarity of the central-atom plus neighbor group strongly influence the distribution of 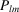 values, the zero-value thresholds and therefore the assigned pseudosymmetry. Pseudosymmetries derived solely from 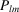 distributions also permit a more objective planarity threshold for atoms with three neighbors.

We have shown the importance of selecting an optimal LCS orientation in which 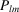 values are averaged to obtain atom-type electron density. An incorrect LCS type may artificially increase the assigned atom-type symmetry and cause the selective loss of important aspherical features of the electron density. We have shown the relation between the topological symmetry, the symmetry of atom-type electron density and the symmetry of the electron density of the atoms constituting those atom types. Averaging within overly universal atom types and across all LCS orientations inflates pseudosymmetry beyond what is supported by the standard deviations of the averaged 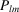 values. The pseudosymmetry of an atom type’s electron density does not always represent the actual symmetry of the atoms from which that atom type is defined. Some atom types, as defined in this work, exhibit systematically higher electron-density symmetry than the atoms they represent, indicating the need to adjust the definition of type topology. Automation makes it possible to identify cases where current atom-type definitions require corrections to the symmetry defined in them and reduces the risk of human error.

The observation that different LCS types may give different pseudosymmetry assignments for the same atoms or atom types, sometimes leading to incorrect results, highlights the complexity of the process and the importance of choosing the most appropriate LCS. For each topological group (4n, 3p *etc*.) an optimal LCS type can be proposed that best reproduces the atom’s pseudosymmetry after averaging within the atom type. The improved LCS definitions result in the same LCS type spanning a larger number of atom types, enabling the use of machine learning methods to optimize existing topology definitions and to introduce new ones based on classifications performed on 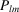.

Comparisons with the MATTS2021 data bank showed that several atom types require updated symmetry and LCS definitions, as well as improvements in topology definitions. Although the MATTS2021 data bank already provides accurate and efficient electron-density modeling, the present findings establish a foundation for restructuring atom-type information in future versions. The next version of the MATTS data bank will incorporate refinements without symmetry constraints along with an expanded set of model molecules. Optimized LCS orientations and automated procedures for assigning symmetry will be incorporated, and information about LCS and symmetry included in atom-type definitions will be updated when necessary in accordance with our findings. The obtained information about the pseudosymmetry of atoms and atom types can be used as a descriptor for defining atom types through machine learning methods.

## Supplementary Material

S1: Figures S1.1-S1.19, Tables S1.1-S1.33, and additional information for the Methods section. DOI: 10.1107/S2053273326004651/tw5016sup1.pdf

S2: Figures S2.1-S2.58 showing ridgeplots and tables with statistical data describing the distributions of multipole model parameters (Î°,P_val,Î°',Plm) for different subgroups of atoms. Statistical metrics include absolute minimum, maximum, mean, median, and sample standard deviation (ssd). Grey rectangles indicate parts of Plm approximated as zero using zero-value thresholds that vary by subgroup. DOI: 10.1107/S2053273326004651/tw5016sup2.pdf

S3: Figures S3.1-S3.18. Pseudosymmetry analysis for atoms and atom types in each subgroup at the level of individual LCS orientations, LCS types, final assignments, and comparisons of atoms versus atom types, and types versus MATTS. DOI: 10.1107/S2053273326004651/tw5016sup3.pdf

S4: Tables S4.1-S4.12. Tables of pseudosymmetry assignments for atom types per LCS type and final, including comparison with the first and second most common pseudosymmetry per atom and comparison with MATTS symmetry, and additional information about atom types (planarity esd, topology, etc.). DOI: 10.1107/S2053273326004651/tw5016sup4.zip

The repository provides additional datasets, analyses and scripts: https://doi.org/10.18150/1MEFPJ

## Figures and Tables

**Table 1:** Zero-value thresholds for each subgroup 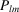
 is considered to be zero when its absolute value is smaller than the given zero-value threshold.

Subgroup	Zero-value threshold
4n-C	0.019
4n-N	0.021
4n-P	0.087
4n-S	0.036
3p-C	0.010
3n-N and 3p-N	0.013
2p-N	0.008
2p-O	0.007
2p-S	0.011
1p-O	0.006
1p-halogens	0.006

**Table 2:** Comparison between the final pseudosymmetry assigned to atom types and the most common final pseudosymmetries assigned to individual atoms that belong to said atom types in each subgroup *Exception: N319 *m*(planar) for atom type, *m*(non-planar) for atoms.

Subgroup	% of atom types where the *first* most common pseudosymmetry for atoms is **the same: lower: higher** than for the atom type	% of atom types where the *first* or *second* most common pseudosymmetry for atoms is **the same** as for the atom type
		
4n-C	43.9%: 56.1%: 0	61.6%
4n-N	11.1%: 88.9%: 0	22.2%
4n-P	63.6%: 27.3%: 9.1%	90.9%
4n-S	45.4%: 54.6%: 0	72.7%
3n-N	40.0%: 60.0%: 0	70.0%
3p-C	28.2%: 70.5%: 1.2%	74.2%
3p-N	44.6%*: 53.6%: 1.8%	71.4%
2p-N	61.1%: 38.9%: 0	88.9%
2p-O	97.2%: 2.8%: 0	100.0%
2p-S	64.3%: 35.7%: 0	85.7%
		
Total	41.4%: 57.7%: 0.9%	72.6%

## Data Availability

The data supporting the results can be accessed in the supporting information files S1–S4 and from the Repository for Open Data (RepOD, Inter­disciplinary Centre for Mathematical and Computational Modelling, University of Warsaw, Warsaw, Poland, https://repod.icm.edu.pl/), https://doi.org/10.18150/1MEFPJ (Rybicka *et al.*, 2025 ▸). The repository provides additional datasets, analyses and scripts. It includes: (i) atom and atom-type (*.ods) datasets for each subgroup with multipole parameters and pseudosymmetry assignments for all LCS orientations; (ii) Gaussian mixture model analyses for determining zero-value thresholds, with histograms (*.png) and statistics (*.txt) per subgroup; (iii) universal atom-type definitions (*txt) with all options of considered LCS orientations; (iv) Bash and Python scripts for preparing definitions, generating 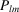 values for given LCS orientations, assigning pseudosymmetry, computing statistics, checking consistency and comparing pseudosymmetries.
