# Supplementary material for: From atoms to a data bank: optimizing transferability of electron-density symmetry
Source: Acta Crystallogr A Found Adv. 2026 Jun 12;82(Pt 4):256–75. doi: 10.1107/S2053273326004651 (PMC13325189; doi:10.1107/S2053273326004651)
Supplement: Supplementary file 1 [file a-82-00256-sup1.pdf]

FOUNDATIONS
ADVANCES

Volume 82 (2026)

Supporting information for article:

From atoms to a data bank: optimizing transferability of electron-density symmetry

Paulina Maria Rybicka, Marta Kulik, Vladislav Ignat'ev and Paulina Maria Dominiak

From atoms to a data bank: optimizing transferability of electron density symmetry

Authors

Paulina Maria Rybicka[a], Marta Kulik[a], Vladislav Ignat'ev[a] and Paulina Maria Dominiak[a]*

[a]University of Warsaw, Faculty of Chemistry, Biological and Chemical Research Centre, Żwirki i Wigury 101, Warsaw, 02-089, Poland

Correspondence email: pdomin@uw.edu.pl

Funding information Narodowe Centrum Nauki (grant No. UMO-2020/39/I/ST4/02904).

Supporting information S1

This file includes Figures S1.1-S1.19, Tables S1.1-S1.33, and additional information for the Methods section

Note on nomenclature: In the main text, the term “no symmetry” has been replaced with “1” for consistency with standard notation. However, in this Supporting Information, the original phrasing “no symmetry” has been retained for better visibility. Both notations refer to the same concept.

IMPORTANT: this document contains embedded data - to preserve data integrity, please ensure where possible that the IUCr Word tools (available from http://journals.iucr.org/services/docxtemplate/) are installed when editing this document.

S1.1 Figures

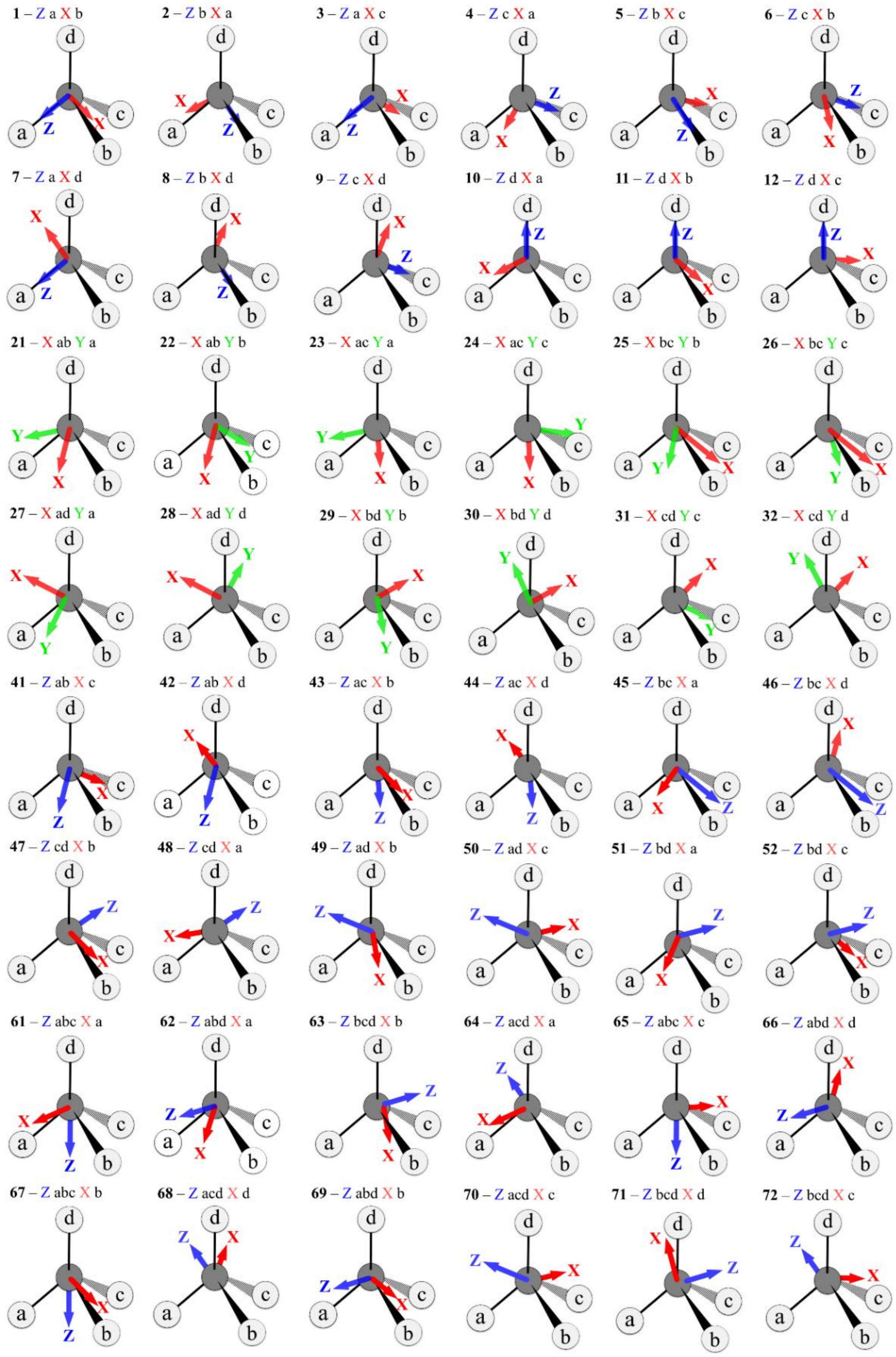

Figure S1.1 All considered LCS orientations for a non-planar atom or atom type with four first neighbors (the 4n group). A total of 48 LCS orientations were generated, 12 for each of the following four LCS types: Z x1 X x2 R, X (x1,x2) Y x1 R, Z (x1,x2) X x3 R, and Z (x1,x2,x3) X x1 R.

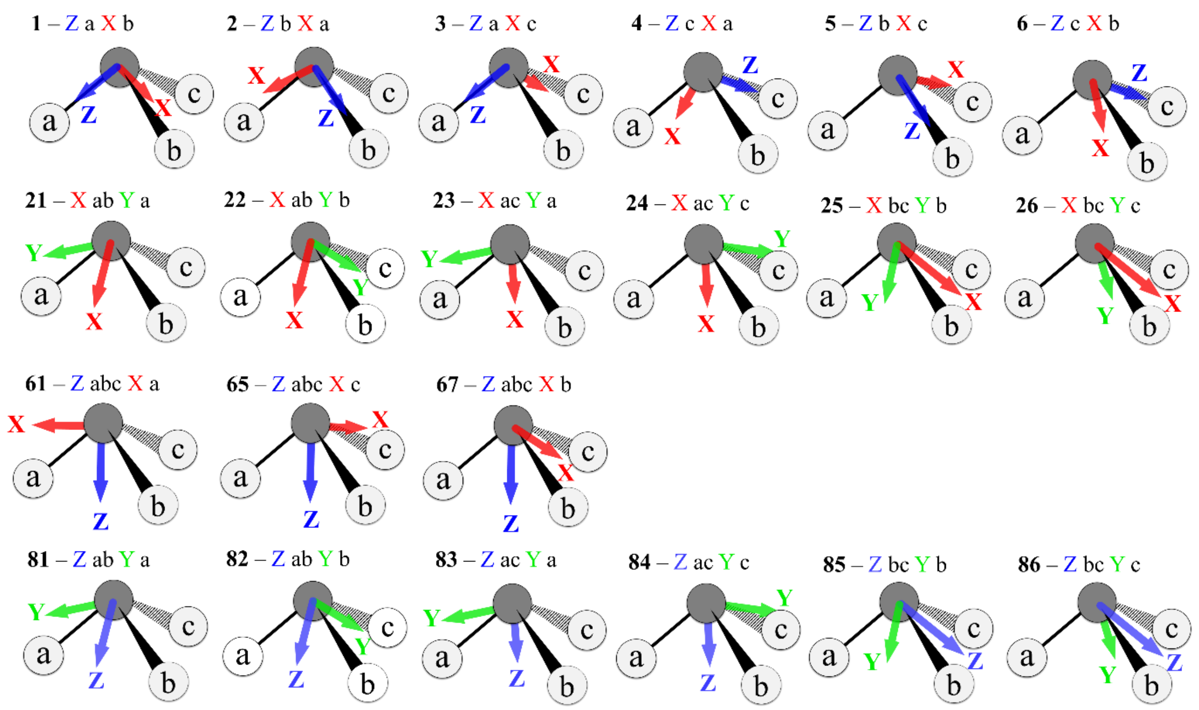

Figure S1.2 All considered LCS orientations for a non-planar atom or atom type with three first neighbors (the 3n group). A total of 21 orientations were considered, six in each of the following three LCS types: Z x1 X x2 R, X (x1,x2) Y x1 R, and Z (x1,x2) Y x1 R, and three orientations in the Z (x1,x2,x3) X x1 R LCS type.

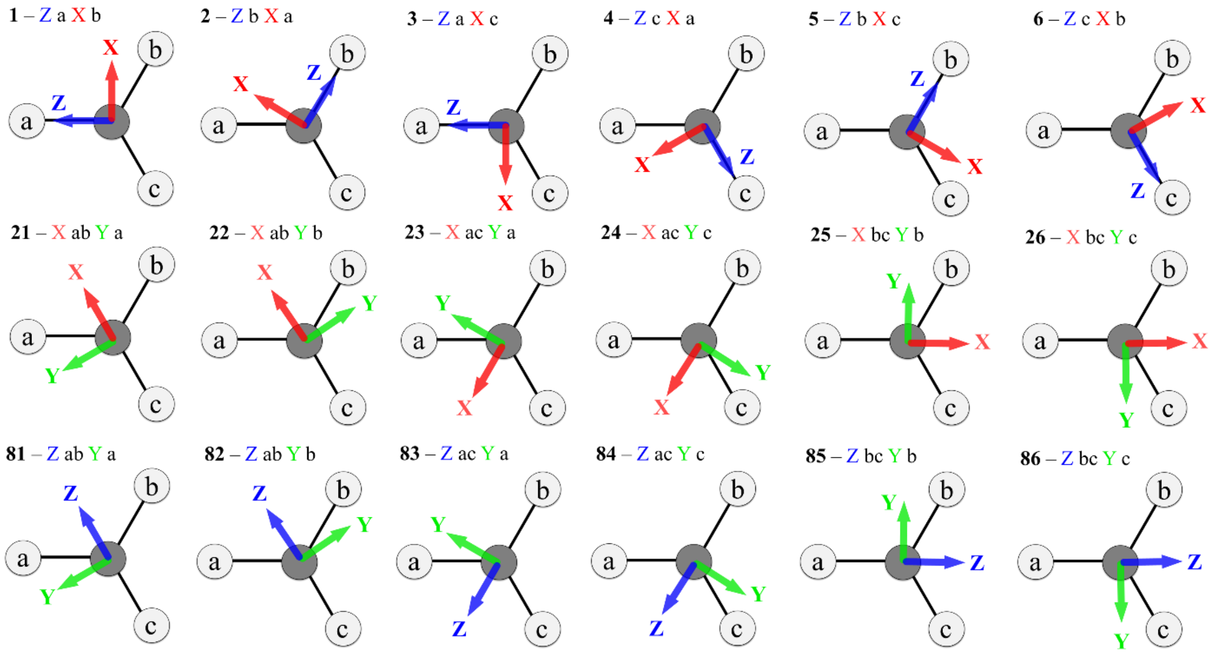

Figure S1.3 All considered LCS orientations for a planar atom or atom type with three first neighbors (the 3p group). A total of 18 orientations were considered, six in each of the following three LCS types: Z x1 X x2 R, X (x1,x2) Y x1 R, and Z (x1,x2) Y x1 R.

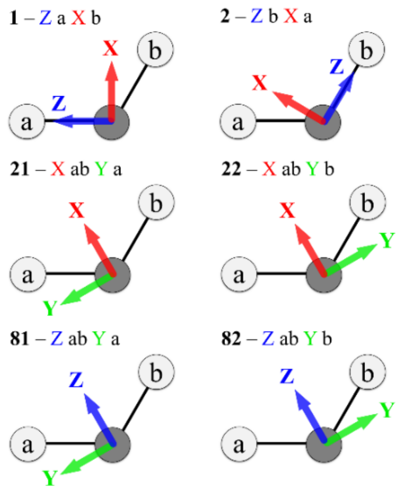

Figure S1.4 All considered LCS orientations for a planar atom or atom type with two first neighbors (the 2p group). A total of six orientations were considered, two in each of the following three LCS types: Z x1 X x2 R, X (x1,x2) Y x1 R, and Z (x1,x2) Y x1 R.

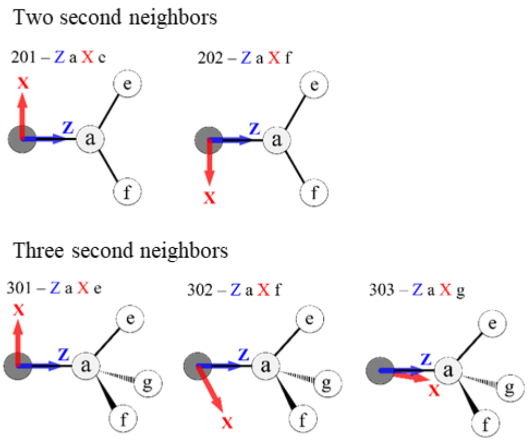

Figure S1.5 All considered LCS orientations for a planar atom or atom type with one first neighbor (the 1p group). The number of orientations of the Z x1 X x2 R LCS type depends on the number and arrangement of second neighbors (the number of LCS orientations is equal to the number of second neighbors). Thus, there are two LCS orientations of the Z x1 X x2 R type for 1p-2 (two second neighbors) group and three for the 1p-3 (three second neighbors) group.

```
ENTRY
 ID
  4n-C
NOI
  9,844
 ATOM DESCRIPTORS
# central atom
  C1   CONNECTED_TO X2,X3,X4,X5     PLANARITY - PLANAR_RING_WITH_PLANAR_ATOMS -     IN_3_MEMBER_RING - IN_4_MEMBER_RING -
# 1-st neighbors
  X2   CONNECTED_TO C1,*        PLANARITY * PLANAR_RING_WITH_PLANAR_ATOMS *     IN_3_MEMBER_RING * IN_4_MEMBER_RING *
  X3   CONNECTED_TO C1,*        PLANARITY * PLANAR_RING_WITH_PLANAR_ATOMS *     IN_3_MEMBER_RING * IN_4_MEMBER_RING *
  X4   CONNECTED_TO C1,*        PLANARITY * PLANAR_RING_WITH_PLANAR_ATOMS *     IN_3_MEMBER_RING * IN_4_MEMBER_RING *
  X5   CONNECTED_TO C1,*        PLANARITY * PLANAR_RING_WITH_PLANAR_ATOMS *     IN_3_MEMBER_RING * IN_4_MEMBER_RING *
 LOCAL COORDINATE SYSTEM
  Z X2 X X3 R
 SYMMETRY
  no
 CHIRALITY
  X2 X3 X4
```

Figure S1.6 An universal atom type definition for the 4n-C subgroup. X – any atom of any chemical element, * – any number of any atoms of any chemical element (including zero); any planarity; any ring size.

```
ENTRY
 ID
  N310
NOI
  204
 ATOM DESCRIPTORS
# central atom
  N1   CONNECTED_TO C2,H3,H4     PLANARITY + PLANAR_RING_WITH_PLANAR_ATOMS -     IN_3_MEMBER_RING - IN_4_MEMBER_RING -
# 1-st neighbors
  C2   CONNECTED_TO N1,X,X       PLANARITY + PLANAR_RING_WITH_PLANAR_ATOMS *     IN_3_MEMBER_RING * IN_4_MEMBER_RING *
  H3   CONNECTED_TO N1,*         PLANARITY * PLANAR_RING_WITH_PLANAR_ATOMS *     IN_3_MEMBER_RING * IN_4_MEMBER_RING *
  H4   CONNECTED_TO N1,*         PLANARITY * PLANAR_RING_WITH_PLANAR_ATOMS *     IN_3_MEMBER_RING * IN_4_MEMBER_RING *
 LOCAL COORDINATE SYSTEM
  Z C2 X H3 R
 SYMMETRY
  no
```

Figure S1.7 Example of an atom type definition for the 3p-N subgroup where information about first neighbors had to be added to properly define the LCS orientation. Added parts are written in magenta font. X – any atom of any chemical element, * – any number of any atoms of any chemical element (including zero); any planarity; any ring size.

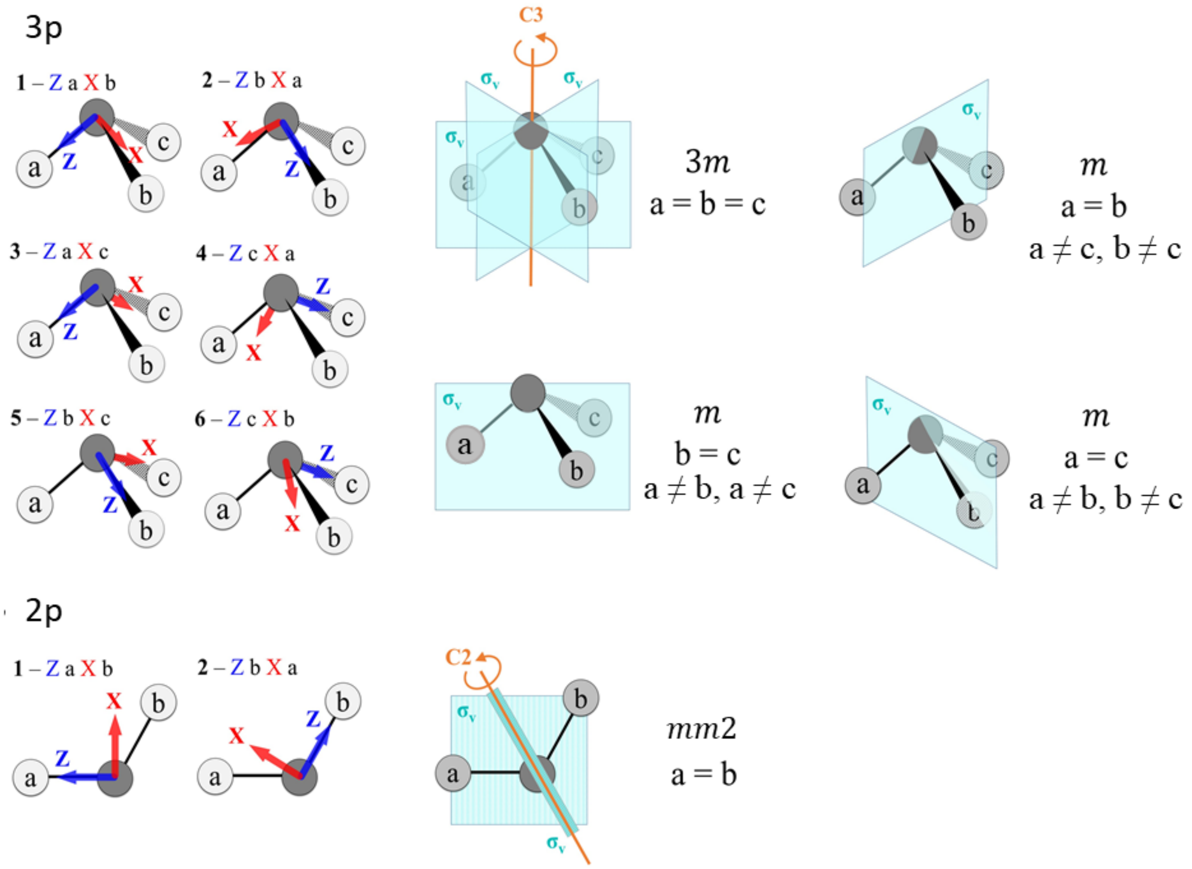

Figure S1.8A graphical representation of symmetry that cannot be seen in the Z x1 X x2 R LCS type for the 3n and 2p topological groups.

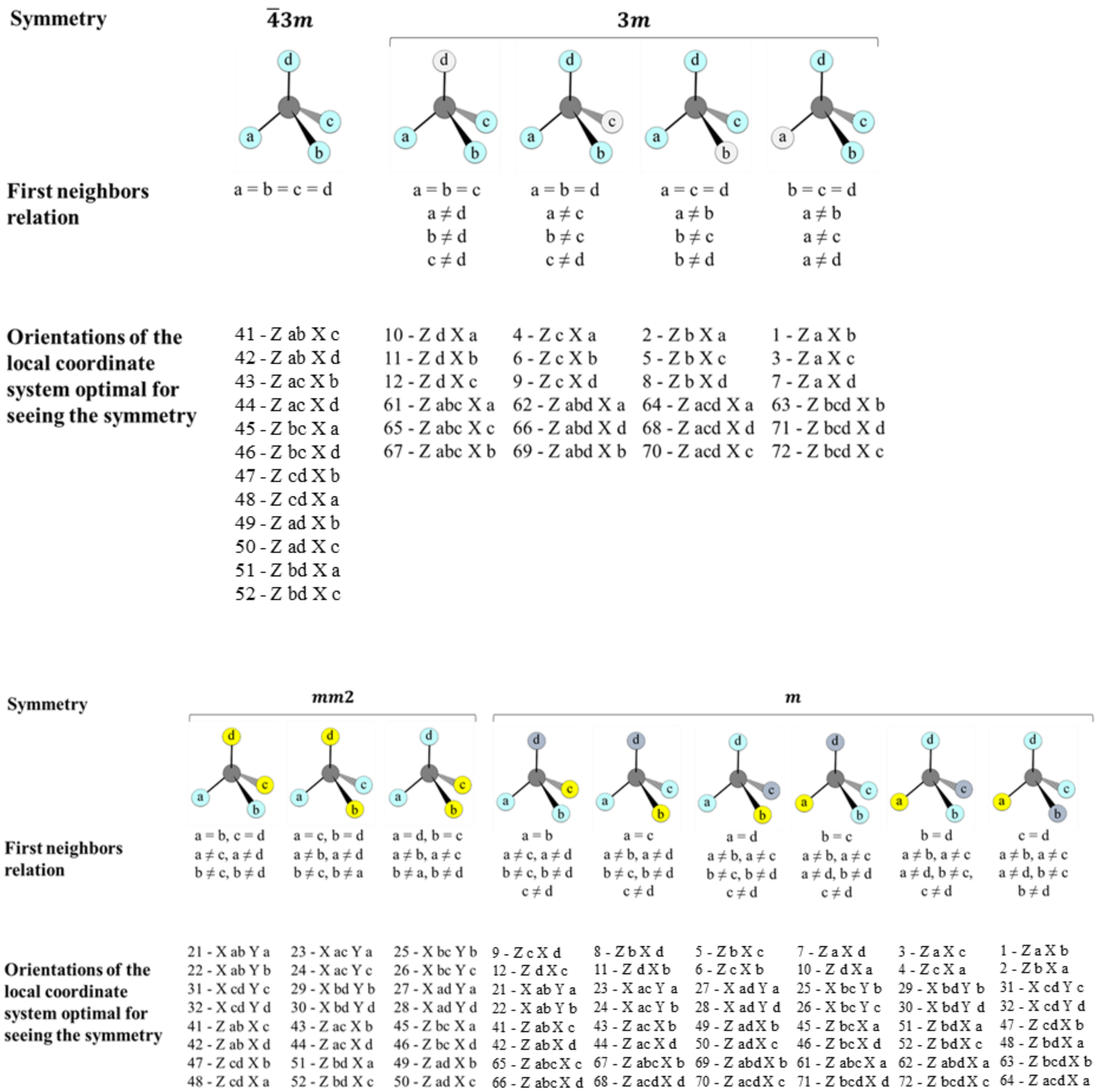

Figure S1.9LCS orientations optimal for seeing symmetry higher than *no* for the 4n topological group.

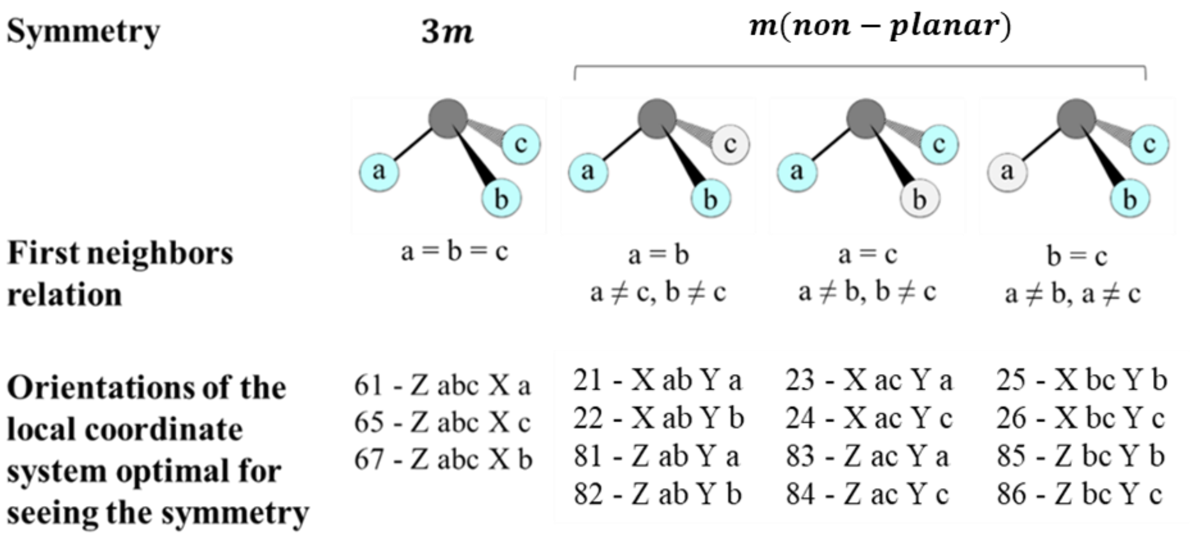

Figure S1.10 LCS orientations optimal for seeing symmetry higher than no for the 3n topological group.

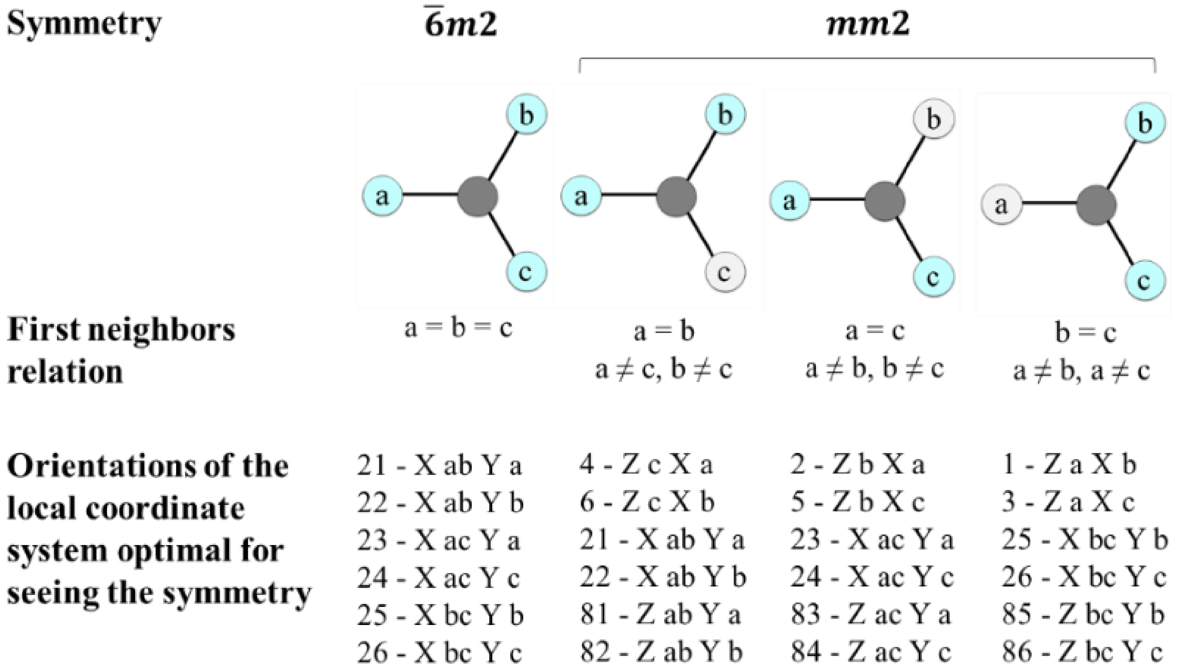

Figure S1.11 LCS orientations optimal for seeing symmetry higher than $m(planar)$ for the 3p topological group.

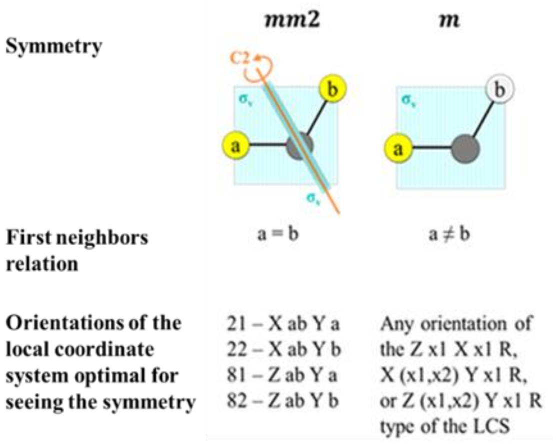

Figure S1.12 LCS orientations optimal for seeing symmetry for the 2p topological group.

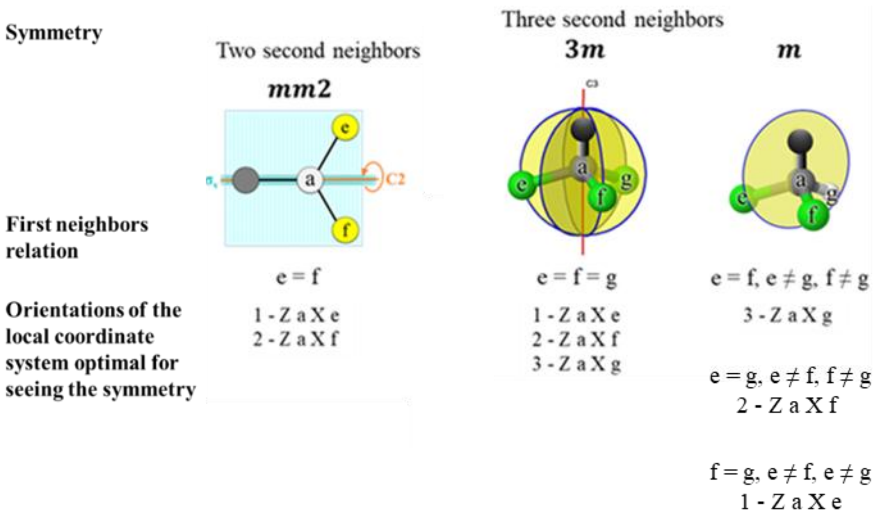

Figure S1.13 LCS orientations optimal for seeing symmetry for the 1p topological group.

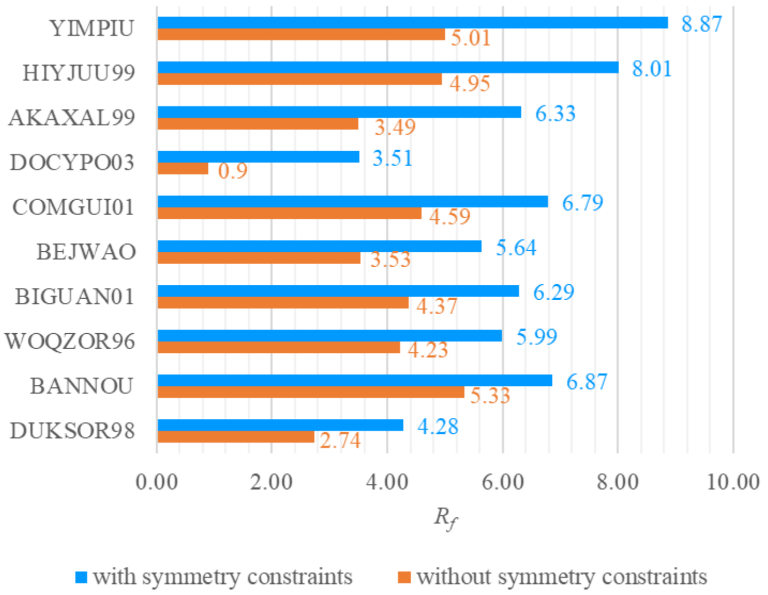

Figure S1.14 The values of R_f (%) for 10 model molecules with the highest change of R_f between refinement with (ref-SC, blue) and without (ref-NSC, orange) symmetry constraints. Refcodes from the CSD for the molecules are listed in the figure (YIMPIU: Jones *et al*, 2008; HIYJUU: Hartmann *et al.*, 1999; AKAXAL: Murthy *et al.*, 2003; DOCYPO03: Pearlman *et al.*, 1985; COMGUI: Kutter *et al.*, 2018; BEJWAO: Czapla *et al.*, 1999; BIGUAN: Pinkerton *et al.*, 1978; WOQZOR: Akriche *et al.*, 2001; BANNOU: Tsuno *et al.*, 2003; DUKSOR: Bentrude *et al.*, 1986). The number over 90 at the end of the refcode (96, 98, 99, etc.) are our additions to distinguish individual molecules if there was more than one molecule in the asymmetric unit.

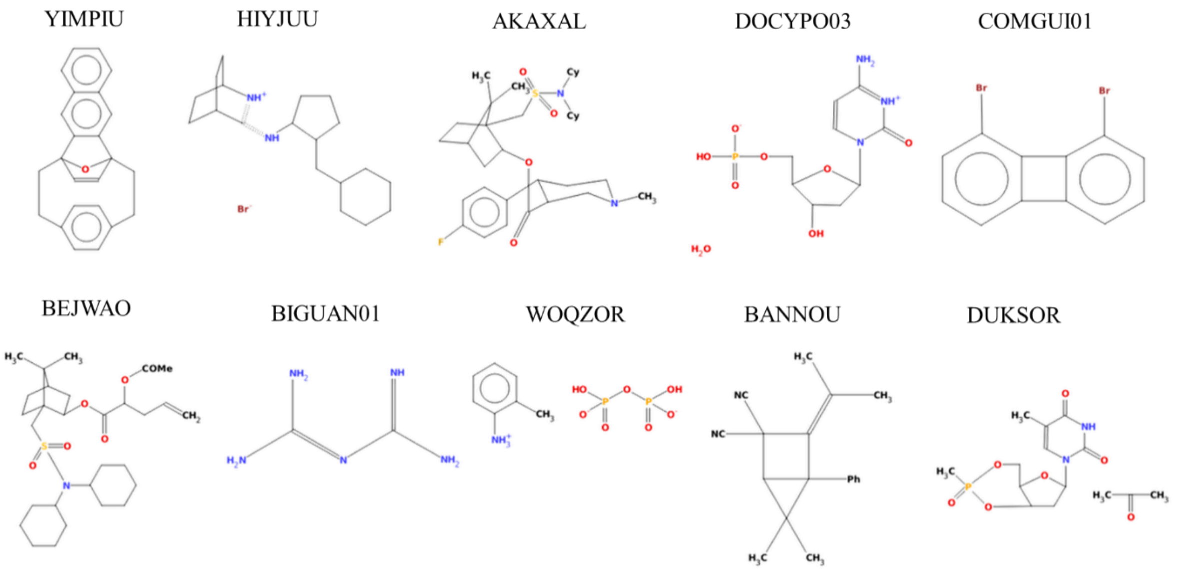

Figure S1.15 CSD diagrams of 10 molecules for which the R_f (%) change between ref-SC and ref-NSC was the highest.

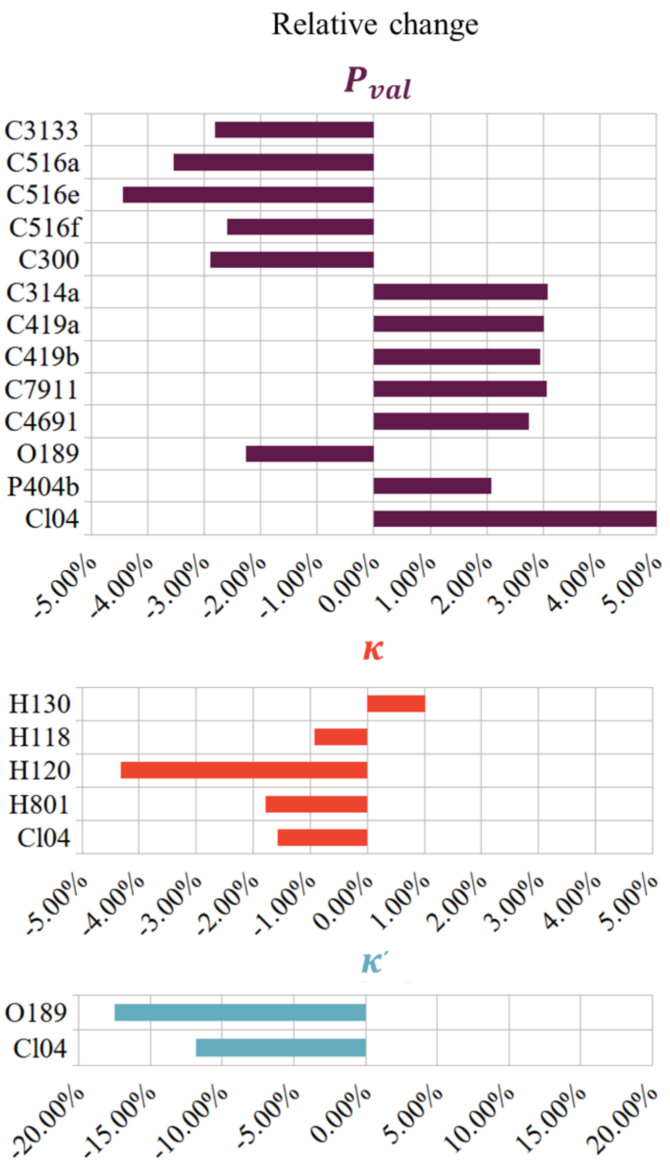

Figure S1.16 The relative changes (%) in the P_{val}, κ, and κ' parameters between atom types from the ref-NSC and the ref-SC datasets, where the absolute value of the change was either higher than 0.1 for P_{val} and κ', or 0.01 for κ. The relative changes were calculated as $\frac{Y_{ref-NSC}-Y_{ref-SC}}{Y_{ref-SC}} \cdot 100$ % where $Y -\ \kappa, \kappa'$, or P_{val}. A positive change indicates that the P_{val}, κ or κ' parameter from ref-NSC is higher than from ref-SC. The atom types are ordered according to their appearance in the MATTS2021 data bank.

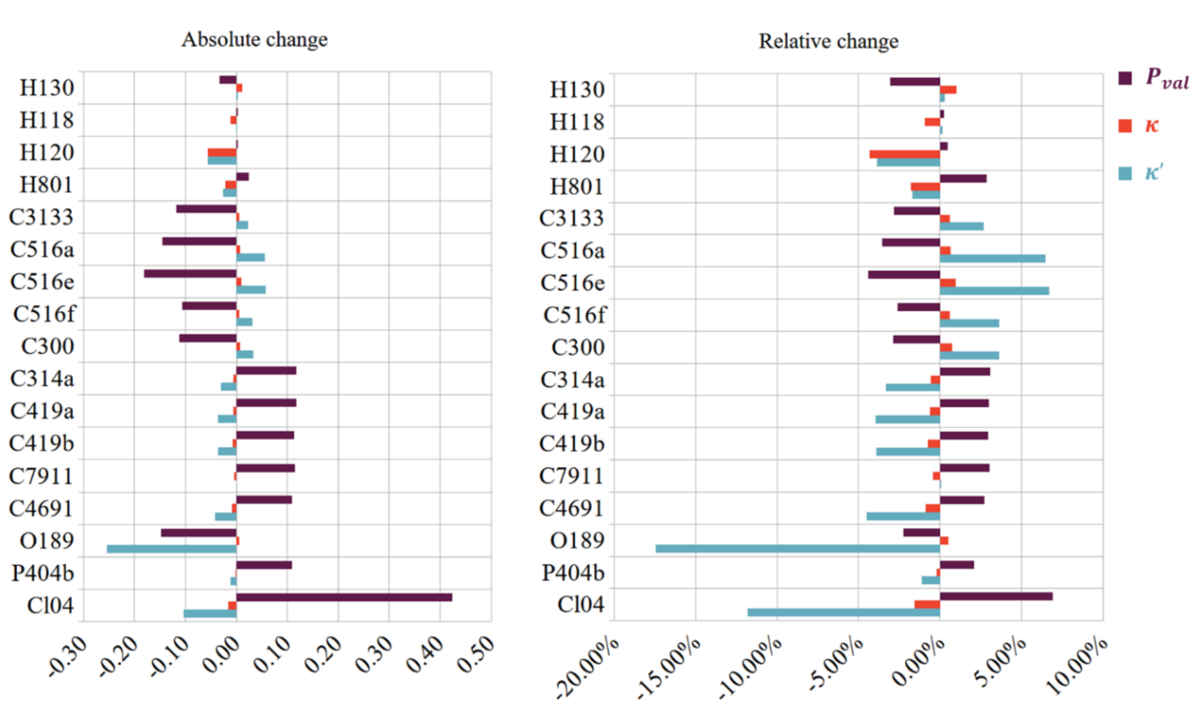

Figure S1.17 The absolute (left) and relative (right) changes of P_{val}, κ, and κ' parameters for atom types where the absolute value of the absolute change for at least one of P_{val}, κ, and κ' parameters was either higher than 0.1 for P_{val} and κ', or 0.01 for κ. Atom types in the figure are shown in the order of appearance in the MATTS2021 data bank.

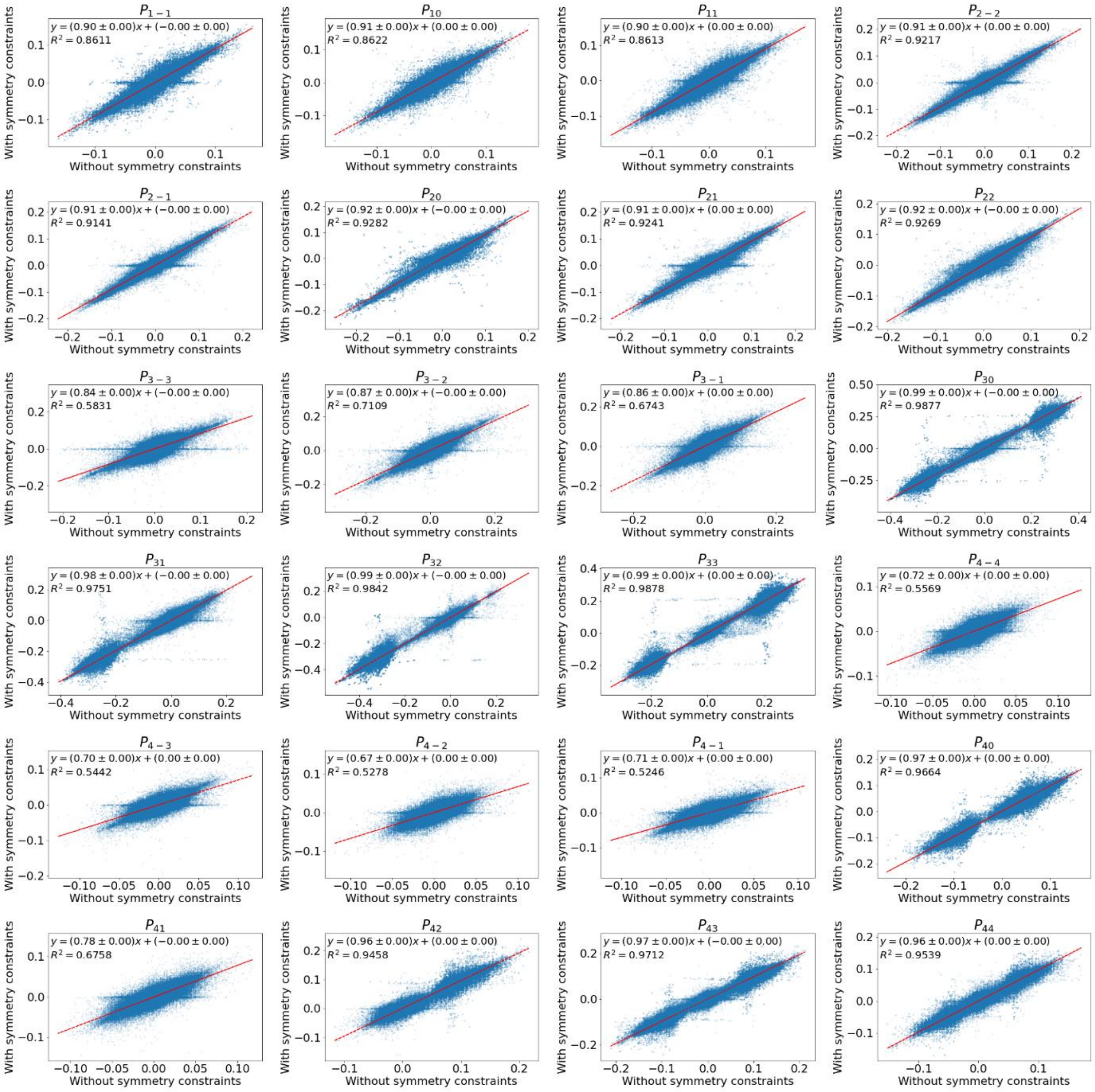

Figure S1.18 Correlation plots for the P_{lm} parameters for 9,844 non-planar carbon atoms with four first neighbors (the 4n-C subgroup) with a trend line (red) for 472,512 LCS orientations for atoms from the refinement of model molecules without symmetry constraints (ref-NSC, x-axis) and with symmetry constraints (ref-SC, y-axis).

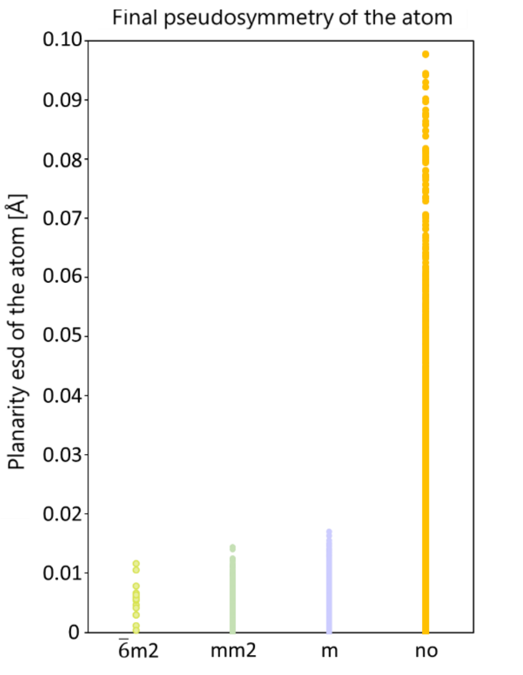

Figure S1.19 Pseudosymmetry of electron density assigned for all 3p-C atoms versus their "planarity *esd*" parameter (ref-NSC).

S1.2 Tables

Table S1.1 Classification of atom types in the MATTS2021 data bank into subgroups. Subgroups omitted in this work are written in a *red cursive* font.

Group	Chemical element of the central atom	Subgroup	Number of atom types	Number of atoms
6n	*P*	*6n-P*	*1*	*15*
4n	C	4n-C	132	9,844
4n	N	4n-N	9	192
4n	P	4n-P	11	168
4n	S	4n-S	11	344
4n	*Cl*	*4n-Cl*	*1*	*10*
3n	*C*	*3n-C*	*1*	*11*
3n	N	3n-N	20	536
3n	*O*	*3n-O*	*2*	*25*
3n	*S*	*3n-S*	*4*	*38*
3p	C	3p-C	241	20,500
3p	N	3p-N	56	2,100
2p	N	2p-N	18	1,144
2p	O	2p-O	36	2,490
2p	S	2p-S	14	493
2x	*H*	*2x-H*	*1*	*7*
2x	*C*	*2x-C*	*4*	*319*
1p	*H*	*1p-H*	*27*	*34,095*
1p	O	1p-O	44	3,721
1p	*S*	*1p-S*	*6*	*110*
1p	F	1p-F	3	485
1p	Cl	1p-Cl	5	352
1p	Br	1p-Br	2	39
1x	*H*	*1x-H*	*1*	*34*
1x	*N*	*1x-N*	*1*	*221*

Table S1.2 Overview of different LCS types used in MATTS2021 with graphical examples. (two pages)

X x1 Y x2 R,<br>where the X axis is oriented from the central atom exactly towards one of the first neighboring atoms, and the Y axis is oriented from the central atom in a general direction of another neighboring atom, first or second	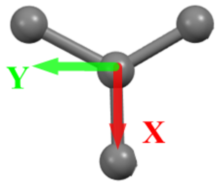
X x1 Y any_orthogonal R,<br>where the X axis is oriented from the central atom exactly towards one of the first neighboring atoms, and the Y axis is oriented from the central atom in a general orthogonal direction of any neighboring atom	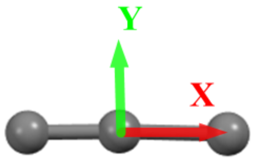
X ring_center Y x1 R,<br>where the X axis is oriented from the central atom towards a center of the ring that the central atom belongs to, and the Y axis is oriented from the central atom in a general direction of one of the neighboring atoms	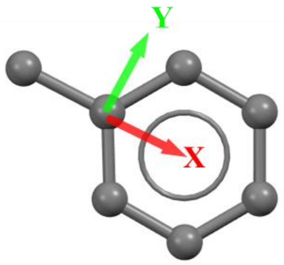
X (x1,x2) Y x3 R,<br>where the X axis is oriented from the central atom exactly in the average direction of two first neighboring atoms, and the Y axis is oriented from the central atom in a general direction of the first neighboring atom different from the ones used to define the X axis	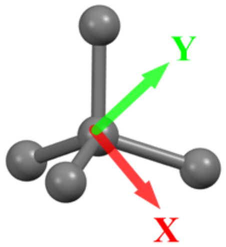
Z x1 X x2 R,<br>where the Z axis is oriented from the central atom exactly towards one of the first neighboring atoms, and the X axis is oriented from the central atom in a general direction of another neighboring atom, first or second	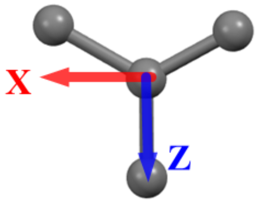
Z x1 X any_orthogonal R,<br>where the Z axis is oriented from the central atom exactly towards one of the first neighboring atoms, and the X axis is oriented from the central atom in a general orthogonal direction of any neighboring atom	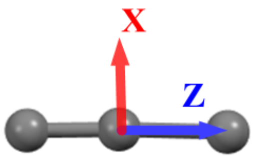

Z (x1,x2) X x1 R,
where the Z axis is oriented from the central atom exactly in the average direction of two first neighboring atoms, and the X axis is oriented from the central atom in a general direction of one of the neighboring atom used to define Z axis

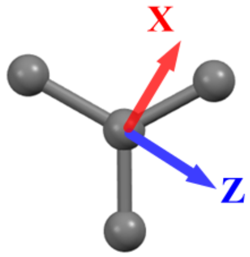

Z (x1,x2) X x3 R,
where the Z axis is oriented from the central atom exactly in the average direction of two first neighboring atoms, and the X axis is oriented from the central atom in a general direction of the first neighboring atom different from the one used to define the Z axis

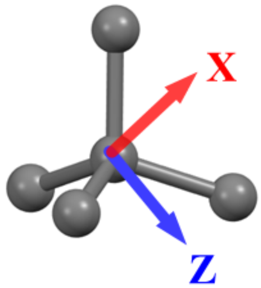

Z (x1,x2,x3) X x1 R,
where the Z axis is oriented from the central atom exactly in the average direction of three the first neighboring atoms, and the X axis is oriented from the central atom in a general direction of one of the neighboring atoms used to define the Z axis

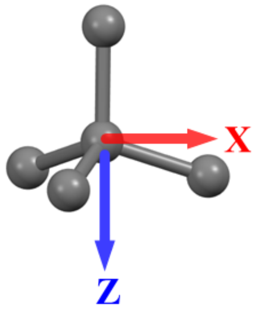

Table S1.3 Overview of different LCS types used per subgroup in MATTS2021 data bank.

Subgroup	LCS type	Number of atom types	Subgroup	LCS type	Number of atom types
6n-P	X x1 Y x2 R	1	3p-N	X x1 Y x2 R	18
4n-C	X x1 Y x2 R	94		X ring_center Y x1 R	26
	Z x1 X x2 R	25		Z x1 X x2 R	12
	Z (x1,x2) X x3 R	13	2p-N	X x1 Y x2 R	10
4n-N	X x1 Y x2 R	2		X ring_center Y x1 R	8
	Z x1 X x2 R	4	2p-O	X x1 Y x2 R	24
	Z (x1,x2) X x3 R	1		X ring_center Y x1 R	6
4n-P	X x1 Y x2 R	5		Z (x1,x2) X x1 R	6
	Z x1 X x2 R	5	2p-S	X x1 Y x2 R	7
	Z (x1,x2) X x3 R	1		X ring_center Y x1 R	4
4n-S	X x1 Y x2 R	4		Z (x1,x2) X x1 R	3
	Z x1 X x2 R	3	2x-H	Z x1 X any_orthogonal R	1
	Z (x1,x2) X x3 R	4	2x-C	Z x1 X any_orthogonal R	4
4n-Cl	Z (x1,x2) X x3 R	1	1p-H	Z x1 X x2 R	21
3n-C	X x1 Y x2 R	1		Z x1 X any_orthogonal R	5
3n-N	X x1 Y x2 R	8	1p-O	X x1 Y x2 R	44
	X (x1,x2) Y x3 R	11	1p-S	X x1 Y x2 R	6
	Z (x1,x2,x3) X x1 R	1	1p-F	X x1 Y x2 R	1
3n-O	X (x1,x2) Y x3 R	1		X x1 Y any_orthogonal R	2
	Z (x1,x2,x3) X x1 R	1	1p-Cl	X x1 Y x2 R	5
3n-S	X x1 Y x2 R	1	1p-Br	X x1 Y x2 R	2
	X (x1,x2) Y x3 R	3	1x-H	Z x1 X any_orthogonal R	2
3p-C	X x1 Y x2 R	73	1x-N	Z x1 X any_orthogonal R	1
	X ring_center Y x1 R	141			
	Z x1 X x2 R	27			

Table S1.4 All considered LCS types and orientations. The a, b, c, and d symbols denote the first neighbors. The e, f, and g symbols denote the second neighbors. (two pages)

LCS type	Orientation of the axes	Index number	Exact LCS orientation
Z x1 X x2 R	Z axis from the central atom exactly towards one of the first neighboring atoms, X axis from the central atom in a general direction of another neighboring atom, first or second	1	Z a X b
		2	Z b X a
		3	Z a X c
		4	Z c X a
		5	Z b X c
		6	Z c X b
		7	Z a X d
		8	Z b X d
		9	Z c X d
		10	Z d X a
		11	Z d X b
		12	Z d X c
		201	Z a X e
		202	Z a X f
		301	Z a X e
		302	Z a X f
		303	Z a X g
X (x1,x2) Y x1 R	X axis from the central atom exactly in the average direction of two the first neighboring atoms (x1 and x2), Y axis from the central atom in a general direction of one of the neighboring atom used to define X axis (x1 or x2)	21	X ab Y a
		22	X ab Y b
		23	X ac Y a
		24	X ac Y c
		25	X bc Y b
		26	X bc Y c
		27	X ad Y a
		28	X ad Y d
		29	X bd Y b
		30	X bd Y d
		31	X cd Y c
		32	X cd Y d
Z (x1,x2) X x3 R	Z axis from the central atom exactly in the average direction of two first neighboring atoms (x1 and x2), X axis from the central atom in a general direction of the first neighboring atom different from the one used to define the Z axis (x3 ≠ x1, x3 ≠ x2)	41	Z ab X c
		42	Z ab X d
		43	Z ac X b
		44	Z ac X d
		45	Z bc X a
		46	Z bc X d
		47	Z cd X b
		48	Z cd X a
		49	Z ad X b
		50	Z ad X c
		51	Z bd X a
		52	Z bd X c
**Z (x1,x2,x3) X x1 R**	Z axis from the central atom exactly in the average direction of three the first neighboring atoms (x1, x2, and x3), X axis from the central atom in a general direction of one of the neighboring atoms used to define the Z axis	61	Z abc X a
		62	Z abd X a
		63	Z bcd X b
		64	Z acd X a
		65	Z abc X c
		66	Z abd X d
		67	Z abc X b
		68	Z acd X d
		69	Z abd X b
		70	Z acd X c
		71	Z bcd X d
		72	Z bcd X c

**Z (x1,x2) Y x1 R**	Z axis from the central atom exactly in the average direction of two the first neighboring atoms (x1 and x2), Y axis from the central atom in a general direction of one of the neighboring atom used to define the Z axis (x1 or x2)	81<br>82<br>83<br>84<br>85<br>86	Z ab Y a<br>Z ab Y b<br>Z ac Y a<br>Z ac Y c<br>Z bc Y b<br>Z bc Y c

Table S1.5 Number of sets of P_{lm} parameters generated per subgroup for ref-SC and ref-NSC.

Subgroup	Number of LCS orientations	Sets of P_{lm} expressed in individual LCS orientations for atoms	Sets of P_{lm} expressed in individual LCS orientations for atom types
**ref-SC**			
4n-C	48	472,512	6,336
3n-N	21	11,256	420
3p-N	18	37,800	1,008
**ref-NSC**			
4n-C	48	472,512	6,336
4n-N	48	9,216	432
4n-P	48	8,064	528
4n-S	48	16,512	528
3n-N	21	11,256	420
3p-C	18	369,000	4,338
3p-N	18	37,800	1,008
2p-N	6	6,864	108
2p-O	6	14,940	216
2p-S	6	2,958	84
1p-O	2 or 3	8,521	102
1p-halogens	2 or 3	2,111	27

Table S1.6 The Kurki-Suonio rules for P_{lm} allowed in each considered symmetry point group.

Symmetry	Our notation	Coordinate axes	P_{lm} that are allowed (values do not have to be 0)
$\bar{6}m2$	$\bar{6}m2(m \perp y)$	$\bar{6} \parallel z, m \perp y$	P_{20}, P_{33}, P_{40}
$\bar{6}m2$	$\bar{6}m2(m \perp x)$	$\bar{6} \parallel z, m \perp x$	P_{20}, P_{3-3}, P_{40}
$\bar{4}3m$	$\bar{4}3m(\bar{4} \parallel z)$	$\bar{4} \parallel z$	$P_{20}, P_{32}, P_{40}, P_{44}$
cyl	cyl	$\infty \parallel z$	$P_{10}, P_{20}, P_{30}, P_{40}$
$3m$	$3m(m \perp y)$	$3 \parallel z, m \perp y$	$P_{10}, P_{20}, P_{30}, P_{33}, P_{40}, P_{43}$
$3m$	$3m(m \perp x)$	$3 \parallel z, m \perp x$	$P_{10}, P_{20}, P_{3-3}, P_{30}, P_{4-3}, P_{40}$
$mm2$	$mm2(2 \parallel z)$	$2 \parallel z$	$P_{10}, P_{20}, P_{22}, P_{30}, P_{32}, P_{40}, P_{42}, P_{44}$
$mm2$	$mm2(2 \parallel y)$	$2 \parallel y$	$P_{1-1}, P_{20}, P_{22}, P_{3-3}, P_{3-1}, P_{40}, P_{42}, P_{44}$
$mm2$	$mm2(2 \parallel x)$	$2 \parallel x$	$P_{11}, P_{20}, P_{22}, P_{31}, P_{33}, P_{40}, P_{42}, P_{44}$
m	$m(m \perp z)$	$m \perp z$	$P_{1-1}, P_{11}, P_{2-2}, P_{20}, P_{22}, P_{3-3}, P_{3-1}, P_{31}, P_{33}, P_{4-4}, P_{4-2}, P_{40}, P_{42}, P_{44}$
m	$m(m \perp y)$	$m \perp y$	$P_{10}, P_{11}, P_{20}, P_{21}, P_{22}, P_{30}, P_{31}, P_{32}, P_{33}, P_{40}, P_{41}, P_{42}, P_{43}, P_{44}$
m	$m(m \perp x)$	$m \perp x$	$P_{1-1}, P_{10}, P_{2-1}, P_{20}, P_{22}, P_{3-3}, P_{3-1}, P_{30}, P_{32}, P_{4-3}, P_{4-1}, P_{40}, P_{42}, P_{44}$
no	no	any	all

Table S1.7 Symmetries higher than no that can be directly observed for individual LCS orientations of each LCS type. If the LCS type was not used for the group, a symbol "-" is written

	Group				
LCS type	4n	3n	3p	2p	1p
Z x1 X x2 R	$3m(m \perp y)$<br>$m(m \perp y)$	none	$mm2(2 \parallel z)$<br>$m(m \perp y)$	$m(m \perp y)$	cyl<br>$3m(m \perp y)$<br>$mm2(2 \parallel z)$<br>$m(m \perp y)$
X (x1,x2) Y x1 R	$mm2(2 \parallel x)$<br>$m(m \perp z)$	$m(m \perp y)$	$\bar{6}m2(m \perp y)$<br>$mm2(2 \parallel x)$<br>$m(m \perp z)$	$mm2(2 \parallel x)$<br>$m(m \perp z)$	-
Z (x1,x2) X x3 R	$\bar{4}3m(\bar{4} \parallel z)$<br>$mm2(2 \parallel z)$<br>$m(m \perp x)$<br>$m(m \perp y)$	-	-	-	-
Z (x1,x2,x3) X x1 R	$3m(m \perp y)$<br>$m(m \perp y)$	$3m(m \perp y)$<br>$m(m \perp y)$	-	-	-
Z (x1,x2) Y x1 R	-	$m(m \perp y)$	$mm2(2 \parallel z)$<br>$m(m \perp x)$	$mm2(2 \parallel z)$<br>$m(m \perp x)$	-

Table S1.8 Symmetries higher than no that can be directly observed in each LCS type. If the LCS type was not used for the group, a symbol "-" is written

	Group				
LCS type	4n	3n	3p	2p	1p
Z x1 X x2 R	$3m$<br>m	none	$mm2$<br>m (planar)	m	cyl<br>$3m$<br>$mm2$<br>m
X (x1,x2) Y x1 R	$mm2$<br>m	m (non-planar)	$\bar{6}m2$<br>$mm2$<br>m (planar)	$mm2$<br>m	-
Z (x1,x2) X x3 R	$\bar{4}3m$<br>$mm2$<br>m	-	-	-	-
Z (x1,x2,x3) X x1 R	$3m$<br>m	$3m$<br>m (non-planar)	-	-	-
Z (x1,x2) Y x1 R	-	m (non-planar)	$mm2$<br>m (planar)	$mm2$<br>m	-

Table S1.9 Symmetry patterns across multiple LCS orientations of the same LCS type for direct and indirect assignment of symmetry higher than *no* for the 4n topological group.

Symmetry		-43m	3m				mm2			m					
Symmetry subgroup			3m-3a	3m-3b	3m-3c	3m-3d	mm2-ab-cd	mm2-ac-bd	mm2-ad-bc	m-ab	m-ac	m-ad	m-bc	m-bd	m-cd
First neighbors relation		a=b=c=d	b=c=d, different a	a=c=d, different b	a=b=d, different c	a=b=c, different d	a=b, c=d	a=c, b=d	a=d, b=c	a=b	a=c	a=d	b=c	b=d	c=d
**Orientations of the LCS: Z x1 X x2 R**															
1	Z a X b	3m(m⊥y)	3m(m⊥y)				m(m⊥y)								m(m⊥y)
2	Z b X a	3m(m⊥y)		3m(m⊥y)			m(m⊥y)								m(m⊥y)
3	Z a X c	3m(m⊥y)	3m(m⊥y)					m(m⊥y)						m(m⊥y)	
4	Z c X a	3m(m⊥y)			3m(m⊥y)			m(m⊥y)						m(m⊥y)	
5	Z b X c	3m(m⊥y)		3m(m⊥y)					m(m⊥y)			m(m⊥y)			
6	Z c X b	3m(m⊥y)			3m(m⊥y)				m(m⊥y)			m(m⊥y)			
7	Z a X d	3m(m⊥y)	3m(m⊥y)						m(m⊥y)				m(m⊥y)		
8	Z b X d	3m(m⊥y)		3m(m⊥y)				m(m⊥y)			m(m⊥y)				
9	Z c X d	3m(m⊥y)			3m(m⊥y)		m(m⊥y)			m(m⊥y)					
10	Z d X a	3m(m⊥y)				3m(m⊥y)			m(m⊥y)				m(m⊥y)		
11	Z d X b	3m(m⊥y)				3m(m⊥y)		m(m⊥y)			m(m⊥y)				
12	Z d X c	3m(m⊥y)				3m(m⊥y)	m(m⊥y)			m(m⊥y)					
**X (x1,x2) Y x1 R**															
21	X ab Y a	mm2(2∥x)	m(m⊥z)	m(m⊥z)			mm2(2∥x)			m(m⊥z)					
22	X ab Y b	mm2(2∥x)	m(m⊥z)	m(m⊥z)			mm2(2∥x)			m(m⊥z)					
23	X ac Y a	mm2(2∥x)	m(m⊥z)		m(m⊥z)			mm2(2∥x)			m(m⊥z)				
24	X ac Y c	mm2(2∥x)	m(m⊥z)		m(m⊥z)			mm2(2∥x)			m(m⊥z)				
25	X bc Y b	mm2(2∥x)		m(m⊥z)	m(m⊥z)				mm2(2∥x)				m(m⊥z)		
26	X bc Y c	mm2(2∥x)		m(m⊥z)	m(m⊥z)				mm2(2∥x)				m(m⊥z)		
27	X ad Y a	mm2(2∥x)	m(m⊥z)			m(m⊥z)			mm2(2∥x)			m(m⊥z)			
28	X ad Y d	mm2(2∥x)	m(m⊥z)			m(m⊥z)			mm2(2∥x)			m(m⊥z)			
29	X bd Y b	mm2(2∥x)		m(m⊥z)		m(m⊥z)		mm2(2∥x)						m(m⊥z)	
30	X bd Y d	mm2(2∥x)		m(m⊥z)		m(m⊥z)		mm2(2∥x)						m(m⊥z)	
31	X cd Y c	mm2(2∥x)			m(m⊥z)	m(m⊥z)	mm2(2∥x)								m(m⊥z)
32	X cd Y d	mm2(2∥x)			m(m⊥z)	m(m⊥z)	mm2(2∥x)								m(m⊥z)
**Z (x1,x2) X x3 R**															
41	Z ab X c	-43m(-4∥z)			m(m⊥y)	m(m⊥y)	mm2(2∥z)			m(m⊥x)					
42	Z ab X d	-43m(-4∥z)			m(m⊥y)	m(m⊥y)	mm2(2∥z)			m(m⊥x)					
43	Z ac X b	-43m(-4∥z)		m(m⊥y)		m(m⊥y)		mm2(2∥z)			m(m⊥x)				
44	Z ac X d	-43m(-4∥z)		m(m⊥y)		m(m⊥y)		mm2(2∥z)			m(m⊥x)				
45	Z bc X a	-43m(-4∥z)	m(m⊥y)			m(m⊥y)			mm2(2∥z)				m(m⊥x)		
46	Z bc X d	-43m(-4∥z)	m(m⊥y)			m(m⊥y)			mm2(2∥z)				m(m⊥x)		
47	Z cd X b	-43m(-4∥z)	m(m⊥y)	m(m⊥y)			mm2(2∥z)								m(m⊥x)
48	Z cd X a	-43m(-4∥z)	m(m⊥y)	m(m⊥y)			mm2(2∥z)								m(m⊥x)
49	Z ad X b	-43m(-4∥z)		m(m⊥y)	m(m⊥y)				mm2(2∥z)			m(m⊥x)			
50	Z ad X c	-43m(-4∥z)		m(m⊥y)	m(m⊥y)				mm2(2∥z)			m(m⊥x)			
51	Z bd X a	-43m(-4∥z)	m(m⊥y)		m(m⊥y)			mm2(2∥z)						m(m⊥x)	
52	Z bd X c	-43m(-4∥z)	m(m⊥y)		m(m⊥y)			mm2(2∥z)						m(m⊥x)	
**Z (x1,x2,x3) X x1 R**															
61	Z abc X a	3m(m⊥y)				3m(m⊥y)			m(m⊥y)				m(m⊥y)		
62	Z abd X a	3m(m⊥y)			3m(m⊥y)			m(m⊥y)						m(m⊥y)	
63	Z bcd X b	3m(m⊥y)	3m(m⊥y)				m(m⊥y)								m(m⊥y)
64	Z acd X a	3m(m⊥y)		3m(m⊥y)			m(m⊥y)								m(m⊥y)
65	Z abc X c	3m(m⊥y)				3m(m⊥y)	m(m⊥y)			m(m⊥y)					
66	Z abd X d	3m(m⊥y)			3m(m⊥y)		m(m⊥y)			m(m⊥y)					
67	Z abc X b	3m(m⊥y)				3m(m⊥y)		m(m⊥y)			m(m⊥y)				
68	Z acd X d	3m(m⊥y)		3m(m⊥y)				m(m⊥y)			m(m⊥y)				
69	Z abd X b	3m(m⊥y)			3m(m⊥y)				m(m⊥y)			m(m⊥y)			
70	Z acd X c	3m(m⊥y)		3m(m⊥y)					m(m⊥y)			m(m⊥y)			
71	Z bcd X d	3m(m⊥y)	3m(m⊥y)						m(m⊥y)				m(m⊥y)		
72	Z bcd X c	3m(m⊥y)	3m(m⊥y)					m(m⊥y)						m(m⊥y)	

Table S1.10 Symmetry patterns across multiple LCS orientations of the same LCS type for direct and indirect assignment of symmetry higher than *no* for the 3n topological group.

		Symmetry	***3m***	***m***		
		Symmetry subgroup		***m-ab***	***m-ac***	***m-bc***
		First neighbors relation	***a=b=c***	***a=b*** ***a≠c b≠c***	***a=c*** ***a≠b b≠c***	***b=c*** ***a≠b a≠c***
Orientations of the LCS	**Z x1 X x2 R**					
	1	Z a X b	Cannot see the symmetry, none of the coordinate axes align with the symmetry elements.			
	2	Z b X a				
	3	Z a X c				
	4	Z c X a				
	5	Z b X c				
	6	Z c X b				
	**X (x1,x2) Y x2 R**					
	21	X ab Y a	*m(m⊥y)*	*m(m⊥y)*		
	22	X ab Y b	*m(m⊥y)*	*m(m⊥y)*		
	23	X ac Y a	*m(m⊥y)*		*m(m⊥y)*	
	24	X ac Y c	*m(m⊥y)*		*m(m⊥y)*	
	25	X bc Y b	*m(m⊥y)*			*m(m⊥y)*
	26	X bc Y c	*m(m⊥y)*			*m(m⊥y)*
	**Z (x1,x2,x3) X x1 R**					
	61	Z abc X a	*3m(m⊥y)*	*m(m⊥y)*		
	65	Z abc X c	*3m(m⊥y)*		*m(m⊥y)*	
	67	Z abc X b	*3m(m⊥y)*			*m(m⊥y)*
	**Z (x1,x2,) Y x1 R**					
	81	Z ab Y a	*m(m⊥y)*	*m(m⊥y)*		
	82	Z ab Y b	*m(m⊥y)*	*m(m⊥y)*		
	83	Z ac Y a	*m(m⊥y)*		*m(m⊥y)*	
	84	Z ac Y c	*m(m⊥y)*		*m(m⊥y)*	
	85	Z bc Y b	*m(m⊥y)*			*m(m⊥y)*
	86	Z bc Y c	*m(m⊥y)*			*m(m⊥y)*

Table S1.11 Symmetry patterns across multiple LCS orientations of the same LCS type for direct and indirect assignment of symmetry higher than *no* for the 3p topological group.

		Symmetry	***-6m2***	***mm2***			***m***
		Symmetry subgroup		***mm2-ab***	***mm2-ac***	***mm2-bc***	
		First neighbors relation	***a=b=c***	***a=b*** ***a≠c*** ***b≠c***	***a=c*** ***a≠b*** ***b≠c***	***b=c*** ***a≠b*** ***a≠c***	***a≠b≠c***
Orientations of the LCS	**Z x1 X x2 R**						
	1	Z a X b	*mm2(2‖z)*	*m(m⊥y)*	*m(m⊥y)*	*mm2(2‖z)*	*m(m⊥y)*
	2	Z b X a	*mm2(2‖z)*	*m(m⊥y)*	*mm2(2‖z)*	*m(m⊥y)*	*m(m⊥y)*
	3	Z a X c	*mm2(2‖z)*	*m(m⊥y)*	*m(m⊥y)*	*mm2(2‖z)*	*m(m⊥y)*
	4	Z c X a	*mm2(2‖z)*	*mm2(2‖z)*	*m(m⊥y)*	*m(m⊥y)*	*m(m⊥y)*
	5	Z b X c	*mm2(2‖z)*	*m(m⊥y)*	*mm2(2‖z)*	*m(m⊥y)*	*m(m⊥y)*
	6	Z c X b	*mm2(2‖z)*	*mm2(2‖z)*	*m(m⊥y)*	*m(m⊥y)*	*m(m⊥y)*
	**X (x1,x2) Y x1 R**						
	21	X ab Y a	*-6m2(m⊥y)*	*mm2(2‖x)*	*m(m⊥z)*	*m(m⊥z)*	*m(m⊥z)*
	22	X ab Y b	*-6m2(m⊥y)*	*mm2(2‖x)*	*m(m⊥z)*	*m(m⊥z)*	*m(m⊥z)*
	23	X ac Y a	*-6m2(m⊥y)*	*m(m⊥z)*	*mm2(2‖x)*	*m(m⊥z)*	*m(m⊥z)*
	24	X ac Y c	*-6m2(m⊥y)*	*m(m⊥z)*	*mm2(2‖x)*	*m(m⊥z)*	*m(m⊥z)*
	25	X bc Y b	*-6m2(m⊥y)*	*m(m⊥z)*	*m(m⊥z)*	*mm2(2‖x)*	*m(m⊥z)*
	26	X bc Y c	*-6m2(m⊥y)*	*m(m⊥z)*	*m(m⊥z)*	*mm2(2‖x)*	*m(m⊥z)*
	**Z (x1,x2) Y x1 R**						
	81	Z ab Y a	*mm2(2‖z)*	*mm2(2‖z)*	*m(m⊥x)*	*m(m⊥x)*	*m(m⊥x)*
	82	Z ab Y b	*mm2(2‖z)*	*mm2(2‖z)*	*m(m⊥x)*	*m(m⊥x)*	*m(m⊥x)*
	83	Z ac Y a	*mm2(2‖z)*	*m(m⊥x)*	*mm2(2‖z)*	*m(m⊥x)*	*m(m⊥x)*
	84	Z ac Y c	*mm2(2‖z)*	*m(m⊥x)*	*mm2(2‖z)*	*m(m⊥x)*	*m(m⊥x)*
	85	Z bc Y b	*mm2(2‖z)*	*m(m⊥x)*	*m(m⊥x)*	*mm2(2‖z)*	*m(m⊥x)*
	86	Z bc Y c	*mm2(2‖z)*	*m(m⊥x)*	*m(m⊥x)*	*mm2(2‖z)*	*m(m⊥x)*

Table S1.12 Symmetry patterns across multiple LCS orientations of the same LCS type for direct assignment of symmetry higher than *no* for the 2p topological group.

Orientations of the LCS	Symmetry	***mm2***	***m***
	First neighbors relation	***a=b***	***a≠b***
	**Z x1 X x2 R**		
	1 Z a X b	■	*m(m⊥y)*
	2 Z b X a	■	*m(m⊥y)*
	**X (x1,x2) Y x1 R**		
	21 X ab Y a	*mm2(2 ∥x)*	*m(m⊥z)*
	22 X ab Y b	*mm2(2 ∥x)*	*m(m⊥z)*
	**Z (x1,x2) Y x1 R**		
	81 Z ab Y a	*mm2(2 ∥z)*	*m(m⊥x)*
	82 Z ab Y b	*mm2(2 ∥z)*	*m(m⊥x)*

■ Cannot see the symmetry, none of the coordinate axes align with the symmetry elements.

Table S1.13 Symmetry patterns across multiple LCS orientations of the same LCS type for direct assignment of symmetry higher than *no* for the 1p topological group.

Orientations of the LCS		Two 2nd neighbors	
	Symmetry	***mm2***	***m***
	**Z x1 X x2 R**		
	201 Z a X e	*mm2(2 ∥ z)*	*m(m⊥y)*
	202 Z a X f	*mm2(2 ∥ z)*	*m(m⊥y)*

Orientations of the LCS		Three 2nd neighbors				
	Symmetry	***cyl***	***3m***	***m***		
	**Z x1 X x2 R**			***f=g***	***e=g***	***e=f***
	301 Z a X e	*cyl*	*3m(m⊥y)*	*m(m⊥y)*		
	302 Z a X f	*cyl*	*3m(m⊥y)*		*m(m⊥y)*	
	303 Z a X g	*cyl*	*3m(m⊥y)*			*m(m⊥y)*

Table S1.14 Values of the P_{val}, κ, and κ' parameters for atom types where the absolute change between ref-SC and ref-NSC is either greater than 0.1 for P_{val} or κ', or greater than 0.01 for κ. Numbers are presented in the format mean(*ssd*).

	ref-SC (original MATTS2021 values)			ref-NSC		
	P_{val}	κ	κ'	P_{val}	κ	κ'
**H130**	1.098(92)	1.132(42)	1.162(30)	1.064(72)	1.143(47)	1.165(27)
**H118**	0.985(43)	1.209(45)	1.328(51)	0.988(38)	1.198(43)	1.330(51)
**H120**	0.5888(99)	1.299(21)	1.467(16)	0.5915(40)	1.2428(30)	1.4105(20)
**H801**	0.851(54)	1.237(47)	1.520(50)	0.875(24)	1.215(31)	1.494(34)
**C3133**	4.19(12)	0.9915(90)	0.864(35)	4.072(48)	0.9975(36)	0.8870(89)
**C516a**	4.12(24)	0.9924(15)	0.857(13)	3.9744(83)	0.9989(19)	0.912(13)
**C516e**	4.076(12)	0.99162(66)	0.8581(44)	3.8954(46)	1.00107(76)	0.91571(34)
**C516f**	4.084(73)	0.9907(43)	0.857(28)	3.9779(87)	0.9968(12)	0.8881(42)
**C300**	3.87(16)	1.003(10)	0.896(43)	3.758(47)	1.0103(33)	0.929(14)
**C314a**	3.84(11)	1.01(60)	0.935(38)	3.958(89)	1.0044(60)	0.904(34)
**C419a**	3.882(83)	1.1946(45)	0.931(26)	3.999(29)	0.9986(20)	0.894(14)
**C419b**	3.863(72)	1.1976(52)	0.925(31)	3.977(17)	1.002(15)	0.8888(80)
**C7911**	3.744(30)	1.01313(12)	0.90615(50)	3.858(46)	1.0087(28)	0.9066(37)
**C4691**	3.996(70)	0.9956(38)	0.926(51)	4.105(80)	0.9867(47)	0.884(43)
**O189**	6.5098(40)	0.97504(58)	1.459(30)	6.3624(78)	0.98021(73)	1.204(14)
**P404b**	5.27(10)	0.9451(55)	1.043(11)	5.379(34)	0.9431(46)	1.0315(35)
**Cl04**	6.1273(33)	0.99046(71)	0.8777(17)	6.5505(58)	0.97494(79)	0.77393(50)

Table S1.15 Information about atom types where the absolute change between ref-SC and ref-NSC is either greater than 0.1 for P_{val} or κ', or greater than 0.01 for κ.

	Subgroup	NOI	Chemical elements of first neighbors	Planar rings with planar atoms	In 3-member ring	In 4-member rings
**H130**	1x-H	34	C	-	-	-
**H118**	1p-H	23	N	-	-	-
**H120**	1p-H	27	O	-	-	-
**H801**	1p-H	5	P	-	-	-
**C3133**	3p-C	8	CCC	-	-	+
**C516a**	3p-C	3	CCC	-	-	-
**C516e**	3p-C	3	CCC	-	-	-
**C516f**	3p-C	3	CCC	-	-	-
**C300**	3p-C	78	CHH	-	-	-
**C314a**	3p-C	16	OCC	-	-	-
**C419a**	4n-C	8	NCCC	-	*	+
**C419b**	4n-C	5	NCCC	-	*	+
**C7911**	4n-C	2	NCCH	-	-	+
**C4691**	4n-C	3	BrCCC	-	-	-
**O189**	1p-O	40	Cl	-	-	-
**P404b**	4n-P	4	OCCC	-	-	-
**Cl04**	4n-Cl	10	OOOO	-	-	-

Table S1.16 P_{lm} dominant in each LCS type for each subgroup.

Subgroup	LCS type				
	Z x1 X x2 R	X (x1,x2) Y x1 R	Z (x1,x2) X x3 R	Z (x1,x2,x3) X x1 R	Z (x1,x2) Y x1 R
4n-C	P_{30},P_{33},P_{40},P_{43}	P_{31},P_{33},P_{40},P_{42},P_{44}	P_{32},P_{40},P_{44}	P_{30},P_{33},P_{40},P_{43}	–
4n-N	P_{30},P_{33},P_{40},P_{43}	P_{31},P_{33},P_{40},P_{42},P_{44}	P_{32},P_{40},P_{44}	P_{30},P_{33},P_{40},P_{43}	–
4n-P	P_{30},P_{33},P_{40},P_{43}	P_{31},P_{33},P_{42},P_{44}	P_{32},P_{40},P_{44}	P_{30},P_{33},P_{40},P_{43}	–
4n-S	P_{30},P_{33},P_{40},P_{43}	P_{31},P_{33},P_{40},P_{42},P_{44}	P_{32},P_{40},P_{44}	P_{30},P_{33},P_{40},P_{43}	–
3p-C	P_{20},P_{22},P_{30},P_{32}, P_{40},P_{42},P_{44}	P_{20},P_{33},P_{40}	–	–	P_{20},P_{22},P_{30},P_{32}, P_{42},P_{44}
3p-N	P_{22},P_{30},P_{32}	P_{20},P_{33},P_{40}	–	–	P_{20},P_{22},P_{30},P_{32}
3n-N	P_{10},P_{11},P_{20},P_{21}, P_{22},P_{30},P_{32},P_{33}, P_{40},P_{43}	P_{11},P_{20},P_{22},P_{31}, P_{33},P_{42},P_{44}	–	P_{11},P_{20},P_{30},P_{33},P_{43}	P_{11},P_{20},P_{22},P_{30}, P_{32},P_{40},P_{44}
2p-N	P_{10},P_{11},P_{20},P_{21}, P_{22},P_{30},P_{31},P_{32}, P_{33},P_{40},P_{41},P_{42}	P_{10},P_{20},P_{22},P_{31}, P_{33},P_{44}	–	–	P_{10},P_{20},P_{22},P_{30}, P_{32},P_{40},P_{42}
2p-O	P_{10},P_{11},P_{20},P_{21}, P_{22},P_{30},P_{32}, P_{33},P_{40},P_{41}	P_{10},P_{20},P_{22},P_{31}, P_{33},P_{40},P_{44}	–	–	P_{10},P_{20},P_{22},P_{30}, P_{32},P_{40},P_{42}
2p-S	P_{10},P_{11},P_{20},P_{21}, P_{22},P_{30},P_{31},P_{32}, P_{33},P_{40},P_{41},P_{42} P_{43},P_{44}	P_{10},P_{20},P_{22},P_{31}, P_{33},P_{40},P_{42},P_{44}	–	–	P_{10},P_{20},P_{22},P_{30}, P_{32},P_{40},P_{42},P_{44}
1p-O-2	P_{10},P_{20},P_{22},P_{30},P_{42}	–	–	–	–
1p-O-3	P_{10},P_{20},P_{30},P_{42}				
1p-halogens-2	P_{10},P_{20},P_{22},P_{30},P_{40}	–	–	–	–
1p-halogens-3	P_{10},P_{20},P_{30},P_{42}				

Table S1.17 The pseudosymmetry assigned for the electron density of a universal atom type definition for each subgroup in each LCS type, using P_{lm} averaged across all LCS orientations for atoms within a given LCS type (Figures S2.1-S2.58) and the zero-value thresholds. Symbol "–" means that the LCS type was not used for the subgroup.

	**LCS type**				
**Subgroup**	Z x1 X x2 R	X (x1,x2) Y x1 R	Z (x1,x2) X x3 R	Z (x1,x2,x3) X x1 R	Z (x1,x2) Y x1 R
4n-C ref-SC	$3m(m \perp y)$	$mm2(2 \parallel x)$	$\bar{4}3m(\bar{4} \parallel z)$	$3m(m \perp y)$	–
4n-C ref-NSC	$3m(m \perp y)$	$mm2(2 \parallel x)$	$\bar{4}3m(\bar{4} \parallel z)$	$3m(m \perp y)$	–
4n-N	$3m(m \perp y)$	$mm2(2 \parallel x)$	$\bar{4}3m(\bar{4} \parallel z)$	$3m(m \perp y)$	–
4n-P	$3m(m \perp y)$	$mm2(2 \parallel x)$	$\bar{4}3m(\bar{4} \parallel z)$	$3m(m \perp y)$	–
4n-S	$3m(m \perp y)$	$mm2(2 \parallel x)$	$\bar{4}3m(\bar{4} \parallel z)$	$3m(m \perp y)$	–
3n-N ref-SC	$m(m \perp y)$	$mm2(2 \parallel x)$	–	$3m(m \perp y)$	$mm2(2 \parallel z)$
3n-N ref-NSC	$m(m \perp y)$	$mm2(2 \parallel x)$	–	$3m(m \perp y)$	$mm2(2 \parallel z)$
3p-C	$mm2(2 \parallel z)$	$\bar{6}m2(m \perp y)$	–	–	$mm2(2 \parallel z)$
3p-N ref-SC	$mm2(2 \parallel z)$	$\bar{6}m2(m \perp y)$	–	–	$mm2(2 \parallel z)$
3p-N ref-NSC	$mm2(2 \parallel z)$	$\bar{6}m2(m \perp y)$	–	–	$mm2(2 \parallel z)$
2p-N	$m(m \perp y)$	$mm2(2 \parallel x)$	–	–	$mm2(2 \parallel z)$
2p-O	$m(m \perp y)$	$mm2(2 \parallel x)$	–	–	$mm2(2 \parallel z)$
2p-S	$m(m \perp y)$	$mm2(2 \parallel x)$	–	–	$mm2(2 \parallel z)$
1p-O	$mm2(2 \parallel z)$	–	–	–	–
1p-O-2	$mm2(2 \parallel z)$	–	–	–	–
1p-O-3	cyl	–	–	–	–
1p-halogens	cyl	–	–	–	–
1p-halogens-2	$mm2(2 \parallel z)$	–	–	–	–
1p-halogens-3	cyl	–	–	–	–

Table S1.18 Atom types with an assigned pseudosymmetry that implies the similarity between the electron density fragments pointing from the central carbon atom towards neighboring carbon and hydrogen atoms. Results are presented for the ref-NSC, subgroup 4n-C.

**Simplified 1st neighbors**	**Atom type**	**Pseudosymmetry assigned for the atom type**	**Most common pseudosymmetry assigned for atoms**
CCCH	C403a	$\bar{4}3m$	no
	C403b	$mm2$	no
	C998	$mm2$	no
	C999	$mm2$	no
CCClH	C838c	$3m$	$3m$
CCHH	C405	$\bar{4}3m$	no
CHHH	C401	$\bar{4}3m$	$3m$
CHHN	C418	$3m$	no
	C423	$3m$	no
CHHO	C429	$3m$	no
CHHS	C434	$3m$	no
CHOO	C440	$mm2$	no
CHSS	C782	$mm2$	no
CCHS	C459	$3m$	no
CCHO	C430	$3m$	no
CCHF	C855	$3m$	m

Table S1.19 Comparison of pseudosymmetries assigned to individual LCS orientations of atoms from the 4n-C subgroup for ref-SC and ref-NSC.

ref-SC	ref-NSC	Number of LCS orientations	Percentage of LCS orientations
No change between ref-SC and ref-NSC			
no	no	228111	48.28
$m(m\perp y)$	$m(m\perp y)$	59184	12.53
$3m(m\perp y)$	$3m(m\perp y)$	32005	6.77
$m(m\perp z)$	$m(m\perp z)$	13976	2.96
$mm2(2\parallel x)$	$mm2(2\parallel x)$	13254	2.81
$m(m\perp x)$	$m(m\perp x)$	11598	2.45
$\bar{4}3m(\bar{4}\parallel z)$	$\bar{4}3m(\bar{4}\parallel z)$	8025	1.70
$mm2(2\parallel z)$	$mm2(2\parallel z)$	3464	0.73
	total	***369617***	***78.22***
Change between ref-SC and ref-NSC			
$m(m\perp y)$	no	19849	4.20
no	$m(m\perp y)$	18964	4.01
$3m(m\perp y)$	no	10034	2.12
$m(m\perp z)$	no	6983	1.48
no	$3m(m\perp y)$	6371	1.35
$3m(m\perp y)$	$m(m\perp y)$	5011	1.06
$m(m\perp x)$	no	3394	0.72
$mm2(2\parallel x)$	$m(m\perp y)$	3108	0.66
$m(m\perp y)$	$3m(m\perp y)$	2854	0.60
$mm2(2\parallel x)$	$m(m\perp z)$	2532	0.54
no	$m(m\perp z)$	2384	0.50
$\bar{4}3m(\bar{4}\parallel z)$	$m(m\perp y)$	2038	0.43
no	$m(m\perp x)$	1940	0.41
$m(m\perp z)$	$mm2(2\parallel x)$	1928	0.41
$m(m\perp y)$	$mm2(2\parallel x)$	1816	0.38
$\bar{4}3m(\bar{4}\parallel z)$	$m(m\perp x)$	1801	0.38
$mm2(2\parallel x)$	no	1453	0.31
no	$mm2(2\parallel x)$	1258	0.27
$m(m\perp y)$	$\bar{4}3m(\bar{4}\parallel z)$	1141	0.24
$\bar{4}3m(\bar{4}\parallel z)$	no	1043	0.22
$\bar{4}3m(\bar{4}\parallel z)$	$mm2(2\parallel z)$	1031	0.22
$m(m\perp x)$	$\bar{4}3m(\bar{4}\parallel z)$	990	0.21
$mm2(2\parallel z)$	$m(m\perp y)$	720	0.15
no	$\bar{4}3m(\bar{4}\parallel z)$	593	0.13
$m(m\perp y)$	$mm2(2\parallel z)$	521	0.11
$mm2(2\parallel z)$	$m(m\perp x)$	514	0.11
$m(m\perp x)$	$mm2(2\parallel z)$	507	0.11
$mm2(2\parallel z)$	no	469	0.10
no	$mm2(2\parallel z)$	406	0.09
$m(m\perp z)$	$m(m\perp y)$	390	0.08
$mm2(2\parallel z)$	$\bar{4}3m(\bar{4}\parallel z)$	257	0.05
$m(m\perp y)$	$m(m\perp z)$	210	0.04
$m(m\perp x)$	$m(m\perp y)$	184	0.04
$m(m\perp y)$	$m(m\perp x)$	129	0.03
cyl	no	43	0.01
$3m(m\perp x)$	no	26	0.01
cyl	$m(m\perp y)$	2	0.00
$3m(m\perp x)$	$m(m\perp y)$	1	0.00
	total	***102895***	***21.78***

Table S1.20 Comparison of pseudosymmetries assigned to individual LCS orientations of atoms from the 3n-N subgroup for ref-SC and ref-NSC.

ref-SC	ref-NSC	Number of LCS orientations	Percentage of LCS orientations
No change between ref-SC and ref-NSC			
no	no	6849	60.85
$m(m\perp y)$	$m(m\perp y)$	2303	20.46
$3m(m\perp y)$	$3m(m\perp y)$	199	1.77
	total	***9351***	***83.08***
Change between ref-SC and ref-NSC			
no	$m(m\_y)$	1029	9.14
$m(m\perp y)$	no	420	3.73
$m(m\perp x)$	no	124	1.10
$m(m\perp z)$	no	89	0.79
$3m(m\perp y)$	no	85	0.76
$3m(m\perp y)$	$m(m\perp y)$	48	0.43
$mm2(2\parallel z)$	$m(m\perp y)$	38	0.34
$mm2(2\parallel x)$	$m(m\perp y)$	24	0.21
$\bar{6}m2(m\perp y)$	$m(m\perp y)$	16	0.14
no	$3m(m\perp y)$	12	0.11
$mm2(2\parallel z)$	no	6	0.05
$m(m\perp y)$	$3m(m\perp y)$	4	0.04
$m(m\perp x)$	$m(m\perp y)$	4	0.04
$mm2(2\parallel x)$	no	2	0.02
$m(m\perp z)$	$m(m\perp y)$	2	0.02
$3m(m\perp x)$	no	2	0.02
	total	***1905***	***16.92***

Table S1.21 Comparison of pseudosymmetries assigned to individual LCS orientations of atoms from the 3p-N subgroup for ref-SC and ref-NSC.

**ref-SC**	**ref-NSC**	**Number of LCS**	**Percentage of LCS**
No change between ref-SC and ref-NSC			
$m(m \perp y)$	$m(m \perp y)$	5943	15.72
$m(m \perp x)$	$m(m \perp x)$	5288	13.99
$m(m \perp z)$	$m(m \perp z)$	5164	13.66
no	no	4872	12.89
$mm2(2 \parallel z)$	$mm2(2 \parallel z)$	3309	8.75
$mm2(2 \parallel x)$	$mm2(2 \parallel x)$	970	2.57
$\bar{6}m2(m \perp y)$	$\bar{6}m2(m \perp y)$	720	1.90
	total	***26266***	***69.49***
Change between ref-SC and ref-NSC			
no	$m(m \perp y)$	1959	5.18
$m(m \perp x)$	no	1287	3.40
$m(m \perp y)$	no	1157	3.06
$m(m \perp z)$	no	1092	2.89
$mm2(2 \parallel z)$	$m(m \perp y)$	978	2.59
$m(m \perp x)$	$mm2(2 \parallel z)$	585	1.55
no	$m(m \perp z)$	570	1.51
$mm2(2 \parallel z)$	no	550	1.46
$mm2(2 \parallel z)$	$m(m \perp x)$	468	1.24
$m(m \perp y)$	$mm2(2 \parallel z)$	333	0.88
no	$m(m \perp x)$	325	0.86
$m(m \perp z)$	$mm2(2 \parallel x)$	320	0.85
no	$mm2(2 \parallel z)$	265	0.70
$mm2(2 \parallel x)$	$m(m \perp z)$	210	0.56
$\bar{6}m2(m \perp y)$	$m(m \perp z)$	174	0.46
$mm2(2 \parallel x)$	$m(m \perp y)$	166	0.44
$\bar{6}m2(m \perp y)$	$m(m \perp y)$	160	0.42
$\bar{6}m2(m \perp y)$	$mm2(2 \parallel x)$	122	0.32
$m(m \perp z)$	$\bar{6}m2(m \perp y)$	106	0.28
no	$mm2(2 \parallel x)$	92	0.24
$m(m \perp y)$	$mm2(2 \parallel x)$	92	0.24
$m(m \perp x)$	$m(m \perp y)$	92	0.24
$mm2(2 \parallel x)$	no	72	0.19
$\bar{6}m2(m \perp y)$	no	62	0.16
$m(m \perp z)$	$m(m \perp y)$	60	0.16
$m(m \perp y)$	$m(m \perp z)$	58	0.15
$mm2(2 \parallel x)$	$\bar{6}m2(m \perp y)$	50	0.13
$m(m \perp y)$	$\bar{6}m2(m \perp y)$	48	0.13
$m(m \perp y)$	$m(m \perp x)$	35	0.09
no	$\bar{6}m2(m \perp y)$	28	0.07
$3m(m \perp y)$	$m(m \perp y)$	6	0.02
no	$3m(m \perp y)$	4	0.01
$m(m \perp y)$	$3m(m \perp y)$	4	0.01
$3m(m \perp y)$	no	2	0.01
$\bar{6}m2(m \perp y)$	$3m(m \perp y)$	2	0.01
	total	***11534***	***30.51***

Table S1.22 Comparison of pseudosymmetries assigned for ref-SC and ref-NSC in each LCS type for atoms from the 4n-C subgroup. (two pages)

LCS type	ref-SC	ref-NSC	Number of atoms	Percentage of atoms
**Z x1 X x2 R**	*No change between ref-SC and ref-NSC*			
	$3m$	$3m$	1635	16.61
	m	m	1531	15.55
	no	no	1495	15.19
	$mm2$	$mm2$	587	5.96
	$\bar{4}3m$	$\bar{4}3m$	384	3.90
		total	***5632***	***57.21***
	Change between ref-SC and ref-NSC			
	m	no	1330	13.51
	no	m	647	6.57
	$3m$	m	560	5.69
	$\bar{4}3m$	$3m$	432	4.39
	$mm2$	m	315	3.20
	no	$3m$	163	1.66
	$3m$	$\bar{4}3m$	127	1.29
	$3m$	no	124	1.26
	$mm2$	$3m$	115	1.17
	m	$mm2$	102	1.04
	m	$3m$	95	0.97
	$mm2$	no	59	0.60
	no	$mm2$	46	0.47
	$\bar{4}3m$	m	26	0.26
	$3m$	$mm2$	26	0.26
	$\bar{4}3m$	$mm2$	24	0.24
	$\bar{4}3m$	no	8	0.08
	$mm2$	$\bar{4}3m$	7	0.07
	no	$\bar{4}3m$	4	0.04
	m	$\bar{4}3m$	2	0.02
		total	***4212***	***42.79***
**X (x1,x2) Y x1 R**	*No change between ref-SC and ref-NSC*			
	m	m	2175	22.09
	$3m$	$3m$	1290	13.10
	$mm2$	$mm2$	932	9.47
	no	no	706	7.17
	$\bar{4}3m$	$\bar{4}3m$	429	4.36
		total	***5532***	***56.20***
	Change between ref-SC and ref-NSC			
	m	no	1830	18.59
	$\bar{4}3m$	$mm2$	481	4.89
	$3m$	m	464	4.71
	no	m	311	3.16
	$3m$	$mm2$	280	2.84
	$mm2$	m	243	2.47
	m	$mm2$	161	1.64
	no	$mm2$	111	1.13
	m	$3m$	75	0.76
	$3m$	$\bar{4}3m$	67	0.68
	$\bar{4}3m$	$3m$	65	0.66
	$3m$	no	64	0.65
	$mm2$	no	49	0.50
	$\bar{4}3m$	m	31	0.31
	$mm2$	$\bar{4}3m$	29	0.29
	$mm2$	$3m$	23	0.23
	no	$3m$	15	0.15
	m	$\bar{4}3m$	5	0.05
	$\bar{4}3m$	no	4	0.04

	no	$\bar{4}3m$	4	0.04
		total	***4312***	***43.80***
**Z (x1,x2) X x3 R**	*No change between ref-SC and ref-NSC*			
	no	*no*	2130	21.64
	m	m	1873	19.03
	$3m$	$3m$	1191	12.10
	$mm2$	$mm2$	780	7.92
	$\bar{4}3m$	$\bar{4}3m$	416	4.23
		total	***6390***	***64.91***
	Change between ref-SC and ref-NSC			
	m	*no*	977	9.92
	no	m	636	6.46
	$3m$	m	476	4.84
	$\bar{4}3m$	m	280	2.84
	$mm2$	m	229	2.33
	$\bar{4}3m$	$mm2$	172	1.75
	$3m$	$mm2$	147	1.49
	no	$mm2$	106	1.08
	m	$3m$	70	0.71
	m	$mm2$	69	0.70
	$3m$	$\bar{4}3m$	68	0.69
	$\bar{4}3m$	$3m$	65	0.66
	$mm2$	*no*	64	0.65
	$3m$	*no*	24	0.24
	m	$\bar{4}3m$	16	0.16
	$mm2$	$\bar{4}3m$	16	0.16
	$mm2$	$3m$	16	0.16
	no	$3m$	13	0.13
	no	$\bar{4}3m$	6	0.06
	$\bar{4}3m$	*no*	4	0.04
		total	***3454***	***35.09***
**Z (x1,x2,x3) X x1 R**	*No change between ref-SC and ref-NSC*			
	no	*no*	2044	20.76
	$3m$	$3m$	1680	17.07
	m	m	1576	16.01
	$mm2$	$mm2$	636	6.46
	$\bar{4}3m$	$\bar{4}3m$	372	3.78
		total	***6308***	***64.08***
	Change between ref-SC and ref-NSC			
	m	*no*	734	7.46
	no	m	701	7.12
	$3m$	m	518	5.26
	$\bar{4}3m$	$3m$	423	4.30
	m	$3m$	222	2.26
	$mm2$	m	178	1.81
	$mm2$	$3m$	132	1.34
	$3m$	$\bar{4}3m$	118	1.20
	$3m$	*no*	118	1.20
	no	$3m$	108	1.10
	m	$mm2$	97	0.99
	$3m$	$mm2$	57	0.58
	no	$mm2$	40	0.41
	$mm2$	*no*	34	0.35
	$\bar{4}3m$	m	24	0.24
	$\bar{4}3m$	$mm2$	18	0.18
	$\bar{4}3m$	*no*	8	0.08
	$mm2$	$\bar{4}3m$	3	0.03
	m	$\bar{4}3m$	2	0.02
	no	$\bar{4}3m$	1	0.01
		total	***3536***	***35.92***

Table S1.23 Comparison of pseudosymmetries assigned for ref-SC and ref-NSC in each LCS type for atoms from the 3n-N subgroup.

LCS type	ref-SC	ref-NSC	Number of atoms	Percentage of atoms
**Z x1 X x2 R**	*No change between ref-SC and ref-NSC*			
	no	no	536	100.00
		total	***536***	***100.00***
**X (x1,x2) Y x1 R**	*No change between ref-SC and ref-NSC*			
	$m(non-planar)$	$m(non-planar)$	157	29.29
	no	no	99	18.47
	$3m$	$3m$	89	16.60
		total	***345***	***64.37***
	Change between ref-SC and ref-NSC			
	no	$m(non-planar)$	95	17.72
	$m(non-planar)$	$3m$	43	8.02
	no	$3m$	24	4.48
	$3m$	$m(non-planar)$	19	3.54
	$m(non-planar)$	no	8	1.49
	$3m$	no	2	0.37
		total	***191***	***35.63***
**Z (x1,x2) Y x1 R**	*No change between ref-SC and ref-NSC*			
	$m(non-planar)$	$m(non-planar)$	189	35.26
	no	no	127	23.69
	$3m$	$3m$	50	9.33
		total	***366***	***68.28***
	Change between ref-SC and ref-NSC			
	$m(non-planar)$	no	94	17.54
	$3m$	$m(non-planar)$	38	7.09
	no	$m(non-planar)$	26	4.85
	$m(non-planar)$	$3m$	8	1.49
	$3m$	no	3	0.56
	no	$3m$	1	0.19
		total	***170***	***31.72***
**Z (x1,x2,x3) X x1 R**	*No change between ref-SC and ref-NSC*			
	no	no	180	33.58
	$m(non-planar)$	$m(non-planar)$	137	25.56
	$3m$	$3m$	44	8.21
		total	***361***	***67.35***
	Change between ref-SC and ref-NSC			
	$m(non-planar)$	no	57	10.63
	$3m$	$m(non-planar)$	52	9.70
	no	$m(non-planar)$	42	7.84
	$3m$	no	11	2.05
	$m(non-planar)$	$3m$	11	2.05
	no	$3m$	2	0.37
		total	***175***	***32.65***

Table S1.24 Comparison of pseudosymmetries assigned for ref-SC and ref-NSC in each LCS type for atoms from the 3p-N subgroup. (two pages)

LCS type	ref-SC	ref-NSC	Number of atoms	Percentage of atoms
**Z x1 X x2 R**	*No change between ref-SC and ref-NSC*			
	no	no	352	16.76
	$mm2$	$mm2$	219	10.43
	$m(planar)$	$m(planar)$	143	6.81
	$\bar{6}m2$	$\bar{6}m2$	2	0.10
		total	***716***	***34.10***
	Change between ref-SC and ref-NSC			
	no	$m(planar)$	274	13.05
	no	$mm2$	246	11.71
	$mm2$	$m(planar)$	228	10.86
	$mm2$	no	218	10.38
	$m(planar)$	no	178	8.48
	$m(planar)$	$mm2$	128	6.10
	no	$\bar{6}m2$	24	1.14
	$\bar{6}m2$	$m(planar)$	23	1.10
	$\bar{6}m2$	no	22	1.05
	$mm2$	$\bar{6}m2$	17	0.81
	$\bar{6}m2$	$mm2$	13	0.62
	$m(planar)$	$\bar{6}m2$	13	0.62
		total	***1384***	***65.90***
**X (x1,x2) Y x1 R**	*No change between ref-SC and ref-NSC*			
	$mm2$	$mm2$	218	10.38
	no	no	158	7.52
	$m(planar)$	$m(planar)$	118	5.62
	$m(non-planar)$	$m(non-planar)$	27	1.29
	$\bar{6}m2$	$\bar{6}m2$	2	0.10
		total	***523***	***24.90***
	Change between ref-SC and ref-NSC			
	no	$mm2$	239	11.38
	no	$m(planar)$	177	8.43
	$m(planar)$	$mm2$	156	7.43
	no	$m(non-planar)$	133	6.33
	$mm2$	no	130	6.19
	$mm2$	$m(planar)$	125	5.95
	$m(planar)$	no	111	5.29
	$m(planar)$	$m(non-planar)$	87	4.14
	$mm2$	$m(non-planar)$	84	4.00
	$m(non-planar)$	$mm2$	45	2.14
	$m(non-planar)$	$m(planar)$	42	2.00
	$m(non-planar)$	no	39	1.86
	$\bar{6}m2$	$m(planar)$	31	1.48
	$mm2$	$\bar{6}m2$	30	1.43
	no	$\bar{6}m2$	23	1.10
	$\bar{6}m2$	$mm2$	19	0.90
	$\bar{6}m2$	no	18	0.86
	no	$3m$	18	0.86
	$m(planar)$	$\bar{6}m2$	16	0.76
	$mm2$	$3m$	14	0.67
	$\bar{6}m2$	$m(non-planar)$	13	0.62
	$m(non-planar)$	$\bar{6}m2$	11	0.52
	$m(planar)$	$3m$	11	0.52
	$m(non-planar)$	$3m$	5	0.24
		total	***1577***	***75.10***
**Z (x1,x2) Y x1 R**	*No change between ref-SC and ref-NSC*			
	$mm2$	$mm2$	274	13.05
	$m(planar)$	$m(planar)$	148	7.05
	no	no	120	5.71

	$m(non-planar)$	$m(non-planar)$	20	0.95
	$\bar{6}m2$	$\bar{6}m2$	5	0.24
		total	***567***	***27.00***
	Change between ref-SC and ref-NSC			
	$m(planar)$	$mm2$	179	8.52
	no	$mm2$	172	8.19
	$mm2$	$m(planar)$	151	7.19
	no	$m(planar)$	130	6.19
	$mm2$	no	123	5.86
	$mm2$	$m(non-planar)$	117	5.57
	$m(planar)$	no	110	5.24
	no	$m(non-planar)$	102	4.86
	$m(planar)$	$m(non-planar)$	99	4.71
	$mm2$	$\bar{6}m2$	47	2.24
	$m(non-planar)$	$m(planar)$	35	1.67
	$\bar{6}m2$	$m(planar)$	33	1.57
	$m(non-planar)$	no	31	1.48
	$m(non-planar)$	$mm2$	29	1.38
	$\bar{6}m2$	$mm2$	26	1.24
	$m(planar)$	$\bar{6}m2$	26	1.24
	no	$\bar{6}m2$	26	1.24
	$\bar{6}m2$	no	25	1.19
	no	$3m$	19	0.90
	$mm2$	$3m$	14	0.67
	$\bar{6}m2$	$m(non-planar)$	13	0.62
	$m(planar)$	$3m$	12	0.57
	$m(non-planar)$	$\bar{6}m2$	8	0.38
	$m(non-planar)$	$3m$	5	0.24
	$\bar{6}m2$	$3m$	1	0.05
		total	***1533***	***73.00***

Table S1.25 Comparison of pseudosymmetries assigned to individual LCS orientations of atom types from the 4n-C subgroup for ref-SC and ref-NSC.

ref-SC	ref-NSC	Number of LCS	Percentage of LCS
No change between ref-SC and ref-NSC			
$m(m\perp y)$	$m(m\perp y)$	1937	30.57
no	no	1891	29.85
$3m(m\perp y)$	$3m(m\perp y)$	518	8.18
$m(m\perp z)$	$m(m\perp z)$	502	7.92
$mm2(2\parallel x)$	$mm2(2\parallel x)$	310	4.89
$mm2(2\parallel z)$	$mm2(2\parallel z)$	181	2.86
$\bar{4}3m(\bar{4}\parallel z)$	$\bar{4}3m(\bar{4}\parallel z)$	94	1.48
$m(m\perp x)$	$m(m\perp x)$	158	2.49
	total	***5591***	***88.24***
Change between ref-SC and ref-NSC			
$m(m\perp y)$	no	163	2.57
no	$m(m\perp y)$	155	2.45
$3m(m\perp y)$	$m(m\perp y)$	63	0.99
no	$m(m\_z)$	52	0.82
$m(m\perp z)$	no	50	0.79
$m(m\perp x)$	no	27	0.43
$m(m\perp y)$	$3m(m\perp y)$	25	0.39
$mm2(2\parallel z)$	$m(m\perp y)$	22	0.35
$mm2(2\parallel x)$	$m(m\perp z)$	22	0.35
$mm2(2\parallel x)$	$m(m\perp y)$	22	0.35
$m(m\perp z)$	$mm2(2\|\|x)$	22	0.35
no	$3m(m\_y)$	20	0.32
$3m(m\perp y)$	no	19	0.30
no	$m(m\perp x)$	12	0.19
$\bar{4}3m(\bar{4}\parallel z)$	$mm2(2\parallel z)$	11	0.17
$m(m\perp y)$	$mm2(2\parallel z)$	9	0.14
$m(m\perp y)$	$mm2(2\parallel x)$	8	0.13
$m(m\perp y)$	$\bar{4}3m(\bar{4}\parallel z)$	8	0.13
$\bar{4}3m(\bar{4}\parallel z)$	$m(m\perp y)$	8	0.13
$mm2(2\parallel z)$	$m(m\perp x)$	5	0.08
no	$mm2(2\|\|x)$	4	0.06
$mm2(2\parallel z)$	no	4	0.06
$mm2(2\parallel z)$	$\bar{4}3m(\bar{4}\parallel z)$	4	0.06
no	$mm2(2\parallel z)$	3	0.05
$m(m\perp x)$	$mm2(2\parallel z)$	3	0.05
$mm2(2\parallel x)$	no	2	0.03
no	$\bar{4}3m(\bar{4}\parallel z)$	1	0.02
cyl	$m(m\perp y)$	1	0.02
	total	***745***	***11.76***

Table S1.26 Comparison of pseudosymmetries assigned to individual LCS orientations of atom types from the 3n-N subgroup for ref-SC and ref-NSC.

**ref-SC**	**ref-NSC**	**Number of LCS**	**Percentage of LCS**
No change between ref-SC and ref-NSC			
no	no	187	44.52
$m(m\_y)$	$m(m\_y)$	90	21.43
$mm2(2 \parallel z)$	$mm2(2 \parallel z)$	26	6.19
$mm2(2 \parallel x)$	$mm2(2 \parallel x)$	24	5.71
$m(m \perp z)$	$m(m \perp z)$	8	1.90
$m(m \perp x)$	$m(m \perp x)$	8	1.90
	total	***343***	***81.67***
Change between ref-SC and ref-NSC			
no	$m(m\_y)$	19	4.52
$m(m\_y)$	no	17	4.05
$mm2(2 \parallel x)$	$3m(m \perp y)$	10	2.38
$m(m\_y)$	$3m(m \perp y)$	8	1.90
$mm2(2 \parallel z)$	$m(m\_y)$	6	1.43
$mm2(2 \parallel x)$	$m(m\_y)$	4	0.95
$m(m \perp z)$	no	3	0.71
$mm2(2 \parallel x)$	no	2	0.48
$m(m \perp z)$	$m(m\_y)$	2	0.48
$m(m \perp x)$	no	2	0.48
$\bar{6}m2(m \perp y)$	$mm2(2 \parallel x)$	2	0.48
$\bar{6}m2(m \perp y)$	no	1	0.24
$\bar{6}m2(m \perp y)$	$3m(m \perp y)$	1	0.24
	total	***77***	***18.33***

Table S1.27 Comparison of pseudosymmetries assigned to individual LCS orientations of atom types from the 3p-N subgroup for ref-SC and ref-NSC.

ref-SC	ref-NSC	Number of LCS	Percentage of LCS
No change between ref-SC and ref-NSC			
$m(m \perp y)$	$m(m \perp y)$	222	22.02
$m(m \perp z)$	$m(m \perp z)$	202	20.04
$mm2(2 \parallel z)$	$mm2(2 \parallel z)$	199	19.74
$m(m \perp x)$	$m(m \perp x)$	192	19.05
$mm2(2 \parallel x)$	$mm2(2 \parallel x)$	68	6.75
$\bar{6}m2(m \perp y)$	$\bar{6}m2(m \perp y)$	26	2.58
no	no	14	1.39
	total	***923***	***91.57***
Change between ref-SC and ref-NSC			
$mm2(2 \parallel z)$	$m(m \perp x)$	12	1.19
no	$m(m \perp z)$	10	0.99
no	$m(m \perp y)$	9	0.89
$\bar{6}m2(m \perp y)$	$m(m \perp z)$	8	0.79
$mm2(2 \parallel z)$	$m(m \perp y)$	7	0.69
no	$m(m \perp x)$	6	0.60
$m(m \perp y)$	no	6	0.60
$mm2(2 \parallel x)$	$m(m \perp z)$	4	0.40
$m(m \perp z)$	no	4	0.40
$m(m \perp x)$	no	4	0.40
$m(m \perp x)$	$mm2(2 \parallel z)$	4	0.40
no	$mm2(2 \parallel z)$	2	0.20
$mm2(2 \parallel x)$	$\bar{6}m2(m \perp y)$	2	0.20
$m(m \perp z)$	$mm2(2 \parallel x)$	2	0.20
$m(m \perp z)$	$\bar{6}m2(m \perp y)$	2	0.20
$m(m \perp y)$	$mm2(2 \parallel z)$	2	0.20
$mm2(2 \parallel z)$	no	1	0.10
	total	***85***	***8.43***

Table S1.28 Comparison of pseudosymmetries assigned for ref-SC and ref-NSC in each LCS type for atom types from the 4n-C subgroup. (two pages)

LCS type	ref-SC	ref-NSC	Number of atom types	Percentage of atom types
**Z x1 X x2 R**	*No change between ref-SC and ref-NSC*			
	$3m$	$3m$	39	29.55
	$mm2$	$mm2$	31	23.48
	m	m	17	12.88
	no	no	10	7.58
	$\bar{4}3m$	$\bar{4}3m$	3	2.27
		total	***100***	***75.76***
	Change between ref-SC and ref-NSC			
	m	no	6	4.55
	$\bar{4}3m$	$3m$	4	3.03
	m	$mm2$	4	3.03
	no	$3m$	3	2.27
	no	m	3	2.27
	$3m$	m	2	1.52
	$mm2$	m	2	1.52
	$\bar{4}3m$	$mm2$	1	0.76
	$3m$	$\bar{4}3m$	1	0.76
	$3m$	$mm2$	1	0.76
	$3m$	no	1	0.76
	m	$3m$	1	0.76
	$mm2$	$3m$	1	0.76
	$mm2$	no	1	0.76
	no	$mm2$	1	0.76
		total	***32***	***24.24***
**X (x1,x2) Y x1 R**	*No change between ref-SC and ref-NSC*			
	$3m$	$3m$	47	35.61
	m	m	26	19.70
	$mm2$	$mm2$	16	12.12
	$\bar{4}3m$	$\bar{4}3m$	8	6.06
	no	no	6	4.55
		total	***103***	***78.03***
	Change between ref-SC and ref-NSC			
	m	no	5	3.79
	$\bar{4}3m$	$mm2$	2	1.52
	$3m$	$mm2$	2	1.52
	m	$mm2$	2	1.52
	$3m$	m	5	3.79
	$mm2$	m	2	1.52
	no	m	2	1.52
	m	$3m$	8	6.06
	$mm2$	$3m$	1	0.76
		total	***29***	***21.97***
**Z (x1,x2) X x3 R**	*No change between ref-SC and ref-NSC*			
	$3m$	$3m$	33	25.00
	$mm2$	$mm2$	29	21.97
	m	m	22	16.67
	no	no	14	10.61
	$\bar{4}3m$	$\bar{4}3m$	3	2.27
		total	***101***	***76.52***
	Change between ref-SC and ref-NSC			
	m	$mm2$	7	5.30
	$3m$	m	6	4.55
	m	no	4	3.03
	$mm2$	m	4	3.03
	no	m	4	3.03
	$\bar{4}3m$	$3m$	1	0.76
	$\bar{4}3m$	$mm2$	1	0.76

	$3m$	$\bar{4}3m$	1	0.76
	m	$3m$	1	0.76
	$mm2$	$3m$	1	0.76
	no	$3m$	1	0.76
		total	***31***	***23.48***
**Z (x1,x2,x3) X x1 R**	*No change between ref-SC and ref-NSC*			
	no	no	2044	20.76
	$mm2$	$mm2$	1680	17.07
	$3m$	$3m$	1576	16.01
	m	m	636	6.46
	$\bar{4}3m$	$\bar{4}3m$	372	3.78
		total	***100***	***75.76***
	Change between ref-SC and ref-NSC			
	no	m	9	6.82
	m	no	6	4.55
	$mm2$	m	3	2.27
	$3m$	$mm2$	2	1.52
	$3m$	no	2	1.52
	m	$mm2$	2	1.52
	no	$3m$	2	1.52
	no	$mm2$	2	1.52
	$\bar{4}3m$	m	1	0.76
	$3m$	m	1	0.76
	$mm2$	$\bar{4}3m$	1	0.76
	$mm2$	no	1	0.76
		total	***32***	***24.24***

Table S1.29 Comparison of pseudosymmetries assigned for ref-SC and ref-NSC in each LCS type for atom types from the 3n-N subgroup.

LCS type	ref-SC	ref-NSC	Number of atom types	Percentage of atom types
**Z x1 X x2 R**	*No change between ref-SC and ref-NSC*			
	no	no	11	55.00
	$m(planar)$	$m(planar)$	2	10.00
		total	***13***	***65.00***
	Change between ref-SC and ref-NSC			
	no	$m(planar)$	4	20.00
	$m(planar)$	no	2	10.00
	$mm2$	no	1	5.00
		total	***7***	***35.00***
**X (x1,x2) Y x1 R**	*No change between ref-SC and ref-NSC*			
	$m(non-planar)$	$m(non-planar)$	7	35.00
	$mm2$	$mm2$	1	5.00
		total	***8***	***40.00***
	Change between ref-SC and ref-NSC			
	$mm2$	$m(non-planar)$	4	20.00
	$m(non-planar)$	$mm2$	2	10.00
	$3m$	$mm2$	1	5.00
	no	$mm2$	1	5.00
	no	$m(non-planar)$	1	5.00
	$mm2$	$3m$	1	5.00
	no	$3m$	1	5.00
	$3m$	no	1	5.00
		total	***12***	***60.00***
**Z (x1,x2) Y x1 R**	*No change between ref-SC and ref-NSC*			
	$m(non-planar)$	$m(non-planar)$	6	30.00
	$\bar{6}m2$	$\bar{6}m2$	1	5.00
		total	***7***	***35.00***
	Change between ref-SC and ref-NSC			
	$\bar{6}m2$	$m(non-planar)$	2	10.00
	$mm2$	$m(non-planar)$	2	10.00
	$3m$	$\bar{6}m2$	1	5.00
	$3m$	no	1	5.00
	$m(non-planar)$	$\bar{6}m2$	1	5.00
	$m(non-planar)$	$mm2$	1	5.00
	$m(non-planar)$	no	1	5.00
	$\bar{6}m2$	$3m$	1	5.00
	no	$3m$	1	5.00
	no	$m(non-planar)$	1	5.00
	no	$mm2$	1	5.00
		total	***13***	***65.00***
**Z (x1,x2,x3) X x1 R**	*No change between ref-SC and ref-NSC*			
	$m(non-planar)$	$m(non-planar)$	11	55.00
	$3m$	$3m$	5	25.00
	no	no	3	15.00
		total	***19***	***95.00***
	Change between ref-SC and ref-NSC			
	$m(non-planar)$	$3m$	1	5.00
		total	***1***	***5.00***

Table S1.30 Comparison of pseudosymmetries assigned for ref-SC and ref-NSC in each LCS type for atom types from the 3p-N subgroup.

LCS type	ref-SC	ref-NSC	Number of atom types	Percentage of atom types
**Z x1 X x2 R**	*No change between ref-SC and ref-NSC*			
	$mm2$	$mm2$	27	48.21
	$m(planar)$	$m(planar)$	12	21.43
	$\bar{6}m2$	$\bar{6}m2$	2	3.57
	no	no	4	7.14
		total	***45***	***80.36***
	Change between ref-SC and ref-NSC			
	$mm2$	$m(planar)$	4	7.14
	$mm2$	no	2	3.57
	no	$m(planar)$	2	3.57
	$\bar{6}m2$	$mm2$	1	1.79
	$m(planar)$	$mm2$	1	1.79
	$m(planar)$	no	1	1.79
		total	***11***	***19.64***
**X (x1,x2) Y x1 R**	*No change between ref-SC and ref-NSC*			
	$mm2$	$mm2$	32	57.14
	$m(planar)$	$m(planar)$	9	16.07
	$\bar{6}m2$	$\bar{6}m2$	3	5.36
	$m(non-planar)$	$m(non-planar)$	2	3.57
	no	no	2	3.57
		total	***48***	***85.71***
	Change between ref-SC and ref-NSC			
	$mm2$	$m(planar)$	2	3.57
	no	$m(planar)$	2	3.57
	$m(planar)$	$mm2$	1	1.79
	no	$mm2$	1	1.79
	$mm2$	$\bar{6}m2$	1	1.79
	$m(planar)$	no	1	1.79
		total	***8***	***14.29***
**Z (x1,x2) Y x1 R**	*No change between ref-SC and ref-NSC*			
	$mm2$	$mm2$	31	55.36
	$m(planar)$	$m(planar)$	9	16.07
	$\bar{6}m2$	$\bar{6}m2$	5	8.93
	$m(non-planar)$	$m(non-planar)$	2	3.57
	no	no	2	3.57
		total	***49***	***87.50***
	Change between ref-SC and ref-NSC			
	$\bar{6}m2$	$mm2$	3	5.36
	$mm2$	$m(planar)$	1	1.79
	$m(planar)$	$mm2$	1	1.79
	$m(planar)$	no	1	1.79
	no	$m(planar)$	1	1.79
		total	***7***	***12.50***

Table S1.31 Comparison of pseudosymmetries assigned to atom types from the 4n-C subgroup for ref-SC and ref-NSC. Only atom types where the pseudosymmetry changes are presented.

Atom type	ref-SC	ref-NSC	Simplified 1st neighbors	Planar rings	In 3-member rings	In 4-member rings
C402	$3m$	m	CCCH	-	-	-
C400a	$3m$	$mm2$	CCCC	-	-	-
C403b	$3m$	$mm2$	CCCH	-	-	-
C417b	$3m$	no	CCCN	-	-	-
C837b	$3m$	no	BrCCH	-	-	-
C400b	$\bar{4}3m$	m	CCCC	-	-	-
C7901	m	$mm2$	CCHH	-	-	+
C799	m	$mm2$	CCCC	-	+	-
C111	m	no	CCCO	-	-	-
C408	m	no	CCCC	-	-	-
C411	m	no	CCCN	-	-	-
C4751	m	no	CCCC	-	-	-
C952	m	no	CCNO	-	-	-
C958	m	no	CCCH	-	-	-
C405	$mm2$	$\bar{4}3m$	CCHH	-	-	-
C449	$mm2$	m	CCNO	-	-	-
C779	$mm2$	m	COPP	-	-	-
C798	$mm2$	m	CCCC	-	-	+
C420b	$mm2$	no	CCHN	-	-	-
C838c	no	$3m$	CClHH	-	-	-
C889	no	$3m$	CFFF	-	-	-
C403c	no	m	CCCH	-	-	-
C419b	no	m	CCCN	-	*	+
C445	no	m	CCHO	-	-	-
C454	no	m	CCHH	-	-	-
C456	no	m	CCFF	-	-	-
C4753	no	m	CCCN	-	-	-
C785	no	m	CCCN	-	-	-
C792	no	m	CCHN	-	-	+
C793	no	m	CCHO	-	-	+
C464c	no	$mm2$	CCCO	-	-	-
C799b	no	$mm2$	CCCC	-	+	-

Table S1.32 Comparison of pseudosymmetries assigned to atom types from the 3n-N subgroup for ref-SC and ref-NSC. Only atom types where the pseudosymmetry changes are presented.

Atom type	ref-SC	ref-NSC	Simplified 1st neighbors	Planarity esd	Planar rings	In 3-member rings	In 4-member rings
N304s	$m(non-planar)$	$3m$	SCC	0.228322	-	-	-

Table S1.33 Comparison of pseudosymmetries assigned to atom types from the 3p-N subgroup for ref-SC and ref-NSC. Only atom types where the pseudosymmetry changes are presented.

Atom type	ref-SC	ref-NSC	Simplified 1st neighbors	Planarity esd	Planar rings	In 3-member rings	In 4-member rings
N3162	$m(planar)$	$mm2$	NCH	0.047418	-	-	-
N318	no	$mm2$	NNH	0.017736	5A	-	-
N319	$mm2$	$m(planar)$	CCS	0.054727	-	-	-
N325a	$mm2$	no	CCC	0.015455	6A	-	-
N334	$mm2$	no	SSH	0.036273	-	-	-
N3592	no	$m(planar)$	OCC	0.025504	-	-	-
N3593	$m(planar)$	no	OCC	0.030766	-	-	-
N447	no	$m(planar)$	NCH	0.026947	5A	-	-
N458	$mm2$	$m(planar)$	NCH	0.035166	-	-	-

S1.3 Additional information for the Methods section

Local coordinate system (text copied from Supporting Information of Jha et. al, 2022)

"Pseudoatom electron density functions are expressed using the local Cartesian coordinate system. The Cartesian system is centred on the central atom of the atom type and oriented according to neighbouring atoms. The local coordinate system is defined in the XD style7 "ax1 p1 ax2 p2 R/L", where ax1 and ax2 defines the type of axis (X or Y or Z) and p1 and p2 defines directions. The first axis (**ax1**) is oriented along the direction vector defined by **p1**. The second axis **ax2** lays on a plane defined by **p1** and **p2** directions and is as close as to **p2**, i.e. **ax2** is directed along (**p1** x **p2**) x **p1**. The third axis (**ax3**) is perpendicular to the two previous axes **ax3** = h (**ax1** x **ax2**), where h is 1 or -1 and it is chosen in such a way that the required handedness of the coordinate system (R/L) is achieved. Each direction (p1 and p2) can be specified in the MATTS2021 databank by the following ways:

(1) unique atom label (e.g. C1) - direction from the central atom to point located at atom (C1),

(2) chemical element symbol (atom label) (e.g. N(C1)) - direction from the central atom to point located at an atom of element given by atom symbol bonded to atom specified in brackets (in the example any nitrogen atom connected to atom C1),

(3) r(ring label) (e.g. r(6A)) - direction from the central atom to point in the centre of ring (6A),

(4) average_direction(atom_label_1,..,atom_label_n) (e.g. average_direction(C1,C2)) - average direction from the central atom to atoms on the list (also element symbols instead of atom label can be used e.g. average direction (N,N) will be an average direction of vectors from central atom to two nitrogen atoms which are connected to the central atom). For *n* neighbours of the central atoms the average direction is defined as follows:

$$\boldsymbol{r} = \frac{1}{n}\sum_{k=1}^{k=n} \frac{\boldsymbol{r}_k - \boldsymbol{r}_c}{\|\boldsymbol{r}_k - \boldsymbol{r}_c\|}$$

where $\boldsymbol{r}_k$ is a position of the k-th neighbor, $\boldsymbol{r}_c$ is a central atom position and $\|\boldsymbol{v}\|$ denotes length

of a vector $\boldsymbol{v}$.
(5) any_atom(atom_label) (e.g. any_atom(C1)) - direction from the central atom to any atom bonded to atom specified with atom label,
(6) !chemical element symbol(atom label) - direction from the central atom to any atom of different chemical element than given by chemical element symbol bonded to atom specified with atom label (e.g. !H(C1) - any non-hydrogen bonded to C1),
(7) any_orthogonal - any_orthogonal - any direction orthogonal to the other direction used in the definition of the local coordinate system. For example in the following definition of local coordinate system:
Z P2 Y any_orthogonal R
the Z direction is defined by a vector vz from the central atom to the atom P2 and the Y direction is chosen as a direction orthogonal to vz. In practice it is calculated in the following way:
1. Cross products of $\mathbf{v}_z$ with $\mathbf{x}$=[1,0,0] and $\mathbf{y}$=[0,1,0] are calculated:

$$\mathbf{c}_x = \mathbf{v}_z \times \mathbf{x}$$

$$\mathbf{c}_y = \mathbf{v}_z \times \mathbf{y}$$

2. The one of the two vectors ($\mathbf{c}_x$ and $\mathbf{c}_y$) with higher norm (i.e. more orthogonal to $\mathbf{v}_z$) is chosen as the Y direction.
In the case when both directions in the local coordinate system definition are defined as any_orthogonal then the local coordinate system corresponds to the global coordinate system (i.e. **x**=[1,0,0], **y**=[0,1,0], **z**= [0,0,1], where the **x**-axis is collinear with **a**-axis and **z**-axis is parallel to **c***-axis.).
For the following cases: average_direction (element symbol, element symbol), atom symbol (atom label), any_atom(atom_label) and !chemical element symbol(atom label) it is possible that the atom to be used in the definition has to be chosen from a group of atoms fulfilling requirements given by specification – e.g. for !H(C1) - any non-hydrogen bonded to C1 – there may be many non-hydrogen atoms bonded to C1. In such a situation an atom with the highest atomic number is chosen, if there is more than one then the atom with the lowest valence is chosen and if still there is no unique choice than the atom with shortest bond to the named atom is chosen (to the central atom in the case of average_direction). No dummy atoms are explicitly defined in local coordinate system definitions in the MATTS2021 databank, instead average directions from explicitly specified neighbouring atoms are computed on demand. The right-handed (R) coordinate system is always defined in the atom type entry. In case of chiral atom types, the databank creation program and the databank application programs takes care to inverse multipole model parameters or to inverse coordinate system for atoms which are recognised as the other enantiomer."

Planarity (text copied from Supporting Information of Jha et. al, 2022)

"Group "PLANARITY" which is defined for atoms having at least 3 first neighbours and is

specified with ‘+’, ‘-‘ or ‘*’ sign indicating whether the atom and its first neighbours are planar, non-planar or have any/undefined planarity, respectively. The planarity is evaluated on the basis of planarity indexed p computed for the set of n atomic positions and defined as: $p = \frac{1}{n-3}\sqrt{\sum_k d_k^2}$, where d_k is a distance of k-th atom from the plane which minimizes the planarity index. The applied procedure is based on methods described by Haneef et al. and Urzhumtsev. Atom planarity threshold is associated with this descriptor, which was set to 0.1 Å while constructing the MATTS2021 databank.

Local scripts

Universal atom type definitions with every considered LCS orientation listed in Table S1.4 were generated using a group-specific local bash script 1_make-definitions-{group}.bash. The *bankMaker* utility program from the *DiSCaMB* library (Chodkiewicz *et al*., 2018) and a local bash script ROTATE.bash were used to calculate multipole parameters for every considered LCS orientation for each atom. For atom types, the same procedure was applied but using the original MATTS2021 data bank definitions of atom types in an unchanged order, provided in files generated for subgroup-specific local bash script 2_make-little-banks-{subgroup}.bash, instead of the universal atom type ones. The resulting sets of P_{lm} parameters were generated for all LCS orientations in all subgroups for the ref-NSC set, and in the 4n-C, 3p-N, and 3n-N subgroups for ref-SC (Table S1.5) and combined into subgroup-specific files implemented in further analysis by using local bash and Python scripts 3_get-data.bash and 4_make-csv.py.

The local Python script gmm_threshold_analysis.py automatically determined the optimal number of Gaussian components (from one to six) and calculated their weights, representing contributions to the overall distribution.

A local bash script 1_pseudosymmetry-LCS-orientations.bash compared the calculated P_{lm} values with the Kurki-Suonio symmetry selection rules (Kurki-Suonio, 1977), which define the allowed and prohibited multipoles for each symmetry point group (Table S1.6). Next, all LCS orientations of the atom or atom type within a given LCS type were analyzed together with group-specific local bash script 2_pseudosymmetry-LCS-type-{group}.bash. The final step was to compare the symmetries assigned across all LCS types available for a given atom or atom type. This was done using a local group-specific Python script 5_pseudosymmetry-final-{group}.py. For each subgroup, we computed the global distribution of pseudosymmetries assigned at three levels: (1) for individual LCS orientations but grouped by the LCS type, (2) for each LCS type, and (3) as the final pseudosymmetry, using local bash and Python scripts3_get-data.bash-{group}.bash (group-specifc) and 4_calculate-stats-LCS.py. The consistency of pseudosymmetry assignment between LCS types was evaluated using a local group-specific Python script 6_consistency-LCS-{group}.py. The

pseudosymmetry assigned to each atom type was compared with the first and second most common pseudosymmetries assigned to its constituent atoms, to evaluate the agreement between them. Each case was classified according to whether the pseudosymmetry for atoms was the same, higher, or lower than that observed for the atom type, and the number and percentage of each category were calculated, using a local Python script 7_atoms-vs-types.py. The final pseudosymmetry assigned to each atom type was also compared with the symmetry specified in the MATTS2021 data bank. For each atom type, the result was classified as identical, higher, or lower symmetry relative to the MATTS2021 one and summary statistics (counts and percentages) were produced using a local Python script 8_matts-vs-types.

Topological groups and subgroups of atoms and atom types

The subgroups 6n-P (phosphorus with six first neighbors, non-planar), 4n-Cl (chlorine with four first neighbors, non-planar), 3n-C (carbon with three first neighbors, non-planar), 3n-O (oxygen with three first neighbors, non-planar), 3n-S (sulfur with three first neighbors, non-planar), 2x-H (hydrogen with two first neighbors, where the first neighbors and the central hydrogen atom are collinear with one another, e.g., middle H in $H_5O_2^+$), 2x-C (carbon with two first neighbors, where the first neighbors and the central hydrogen atom are collinear with one another), 1p-S (sulfur with one first neighbor, where the first neighbor, second neighbors, and the central hydrogen atom are not collinear with one another), 1x-H (hydrogen with one first neighbor, where the first neighbor, second neighbors, and the central hydrogen atom are collinear with one another), and 1x-N (nitrogen with one first neighbor, where the first neighbor, second neighbors, and the central hydrogen atom are not collinear with one another) were excluded because their occurrence in the MATTS2021 data bank was too limited to permit meaningful statistical analysis (Table S1.1). The 1p-H subgroup was also excluded, as hydrogen electron density along the X–H bond was assumed to be cylindrically symmetric and refined with cylindrical constraints. Although the 1p F, 1p Cl, and 1p Br subgroups were small, they were retained and analyzed together as “1p halogens” due to chemical similarity. The 1p-O and 1p-halogens were further split by the number of second neighbors: 1p-O-2 and 1p-halogen-2 (two second neighbors), 1p-O-3 and 1p-halogen-3 (three second neighbors). Atom types with only one second neighbor were excluded due to low occurrence. The 3n-N and 3p-N subgroups were partially analyzed together (see Section 3.6).

Electron density symmetries inferred from topology

For the 4n group, five symmetry point groups are possible: $\bar{4}3m$, 3m, mm2, m or no symmetry (point group 1, chiral). The $\bar{4}3m$ symmetry occurs when electron density fragments pointing toward all four neighbours are equal; 3m when three are equal and related by a threefold axis; mm2 when two equivalent pairs are present; and m when only two directions are equivalent (Figure 1). For the 3n group, three symmetries are possible: 3m, m, or no. The 3m symmetry arises

when all three fragments are equal and related by a threefold axis, m when two are equivalent and the third differs. For the 3p group, three symmetries are also possible. At minimum, m is always present. If two fragments are equal, mm2 symmetry appears; if all three are equal, the highest symmetry, $\bar{6}$m2, is obtained. In this work, we used two different notations for the m symmetry for atoms and atom types with three first neighbors, depending on the placement of the mirror plane in relation to the central atom and said neighbors, We called these two options m(planar) and m(non-planar). The m(planar) describes a situation where the central atom and all its three first neighbors lay on one plane (within the planarity *esd* threshold) and the mirror plane coincides with atom plane. The m(non-planar) describes a situation when the mirror plane is perpendicular to the average plane of the central atom and its three first neighbors. The explicit distinction between m(planar) and m(non-planar) was essential for analyzing the 3p-N and 3n-N subgroups. These subgroups were partially analyzed together to test whether differences in planarity are reflected in multipole parameters and assigned symmetries. For the 2p group, only mm2 or m are possible. Due to topology (three non-collinear atoms always lay on the same plane), m is the lowest symmetry, while mm2 appears when the two fragments are equivalent. For the 1p group, the highest symmetry depends on the number of second neighbors. With three second neighbors, the highest symmetry is 3m, while with two it is mm2. For three second neighbors, the symmetry reduces to m when only two fragments are equivalent, or to no symmetry when none are equivalent. For two second neighbors, the symmetry reduces to m when the fragments are not equivalent.

Calculation of multipole model parameters in various LCS orientations

For individual atoms, fourteen universal atom type definitions were created, representing the subgroups (4n-C, 3p-N, 2p-O, etc.). These definitions were generalized with respect to neighbors, ring presence, and ring size, only planarity of the central atom was explicitly defined (Figure S1.6, repository). This approach ensured that all atoms fitting a given subgroup, including both recognized (i.e., those with corresponding atom types in MATTS2021) and unrecognized (i.e., those without such atom types) ones, were identified in model molecules. Universal atom type definitions with every considered LCS orientation listed in Table S1.4 were generated using a group-specific local bash script 1_make-definitions-{group}.bash. The *bankMaker* utility program from the *DiSCaMB* library (Chodkiewicz *et al*., 2018) and a local bash script ROTATE.bash were used to calculate multipole parameters for every considered LCS orientation for each atom. The program was modified to ensure correct rotation of multipole functions exactly as specified in the universal atom type definition and to make the procedure independent of the atom order in *xd.res* and *xd.mas* files. Distance-based sorting of neighboring atoms during LCS assignment was removed, allowing all LCS-type-allowed neighbor permutations to be considered. Each LCS orientation represents the same electron density but expressed using different combinations of LCS and values of P_{lm} parameters.

For atom types, the same procedure was applied but using the original MATTS2021 data bank definitions of atom types in an unchanged order, provided in files generated for subgroup-specific local bash script 2_make-little-banks-{subgroup}.bash, instead of the universal atom type ones. When necessary, additional information about first neighbors was included to properly distinguish between them and define the LCS orientation (Figure S1.7). Because atom types themselves do not have explicit neighbor coordinates, the rotations were performed for the atoms belonging to each atom type, and the resulting multipole parameters for atoms were then averaged within given LCS orientation to get parameters for the atom type.

In both cases (individual atoms and atom types), all symmetries in atom type definitions were set to no, preventing the enforcement of any symmetry higher than 1 and enabling all multipolar functions to be populated in the atom type. The resulting sets of P_{lm} parameters were generated for all LCS orientations in all subgroups for the ref-NSC set, and in the 4n-C, 3p-N, and 3n-N subgroups for ref-SC (Table S1.5) and combined into subgroup-specific files implemented in further analysis by using local bash and Python scripts 3_get-data.bash and 4_make-csv.py. The *DiSCaMB* library calculates the values of the multipole model parameters up to the sixth decimal place. For this work we approximate values of κ and κ' to four decimal places, and P_{val} and P_{lm} parameters to three decimal places.

References

Akriche, S. & Rzaigui, M. (2001). CCDC 140842: Experimental Crystal Structure Determination.

Bentrude, W. G., Sopchik, A. E., Bajwa, G.S., Setzer, W.N. & Sheldrick, W.S. (1986). Acta Crystallogr. Sect. C 42, 1027.

Chodkiewicz, M. L., Migacz, S., Rudnicki, W., Makal, A., Kalinowski, J. A., Moriarty, N. W., Grosse-Kunstleve, R. W., Afonine, P. V., Adams, P. D. & Dominiak, P. M. (2018). J. Appl. Crystallogr. **51**, 193–199.

Czapla, A., Chajewski, A., Kiegiel, K., Bauer, T., Wielogorski, Z., Urbanczyk-Lipkowska, Z. & Jurczak, J. (1999). CCDC 114321: Experimental Crystal Structure Determination.

Hartmann, S., Weckert, E. & Frahm, A. W. (1999). CCDC 131269: Experimental Crystal Structure Determination.

Haneef, I.; Moss, D. S.; Stanford, M. J.; Borkakoti, N. Restrained Structure-factor Least-squares Refinement of Protein Structures Using a Vector Processing Computer. Acta Crystallogr. Sect. A 1985, 41 (5), 426–433. https://doi.org/10.1107/S0108767385000915.

Jha, K. K., Gruza, B., Sypko, A., Kumar, P., Chodkiewicz, M. L. & Dominiak, P. M. (2022). J. Chem. Inf. Model. **62**, 3752–3765.

Jones, P. G. & Bubenitschek, P. (2008). CCDC 663120: Experimental Crystal Structure Determination.

Kurki-Suonio, K. (1977). Isr. J. Chem. **16**, 115–123.

Kutter F., Denhof, A., Lork, E., Mebs, S. & Beckmann, J. (2018). CCDC 1587127: Experimental Crystal Structure Determination.

Murthy, K. S. K., Rey, A. W. & Tjepkema, M. (2003). CCDC 196924: Experimental Crystal Structure Determination.

Pearlman, D. A. & Kim, S-H. (1985). Biopolymers **24**, 327.

Pinkerton, A. A. & Schwarzenbach D. (1978). Journal of the Chemical Society, Dalton Transactions, 989.

Tsuno, T., Hoshino, H., Okuda, R. & Sugiyama, K. (2003). CCDC 147455: Experimental Crystal Structure Determination.

Urzhumtsev, A. G. How to Calculate Planarity Restraints. Acta Crystallogr. Sect. A 1991, 47 (6), 723–727. https://doi.org/10.1107/S0108767391006268
