# Supplementary material for: From atoms to a data bank: optimizing transferability of electron-density symmetry
Source: Acta Crystallogr A Found Adv. 2026 Jun 12;82(Pt 4):256–75. doi: 10.1107/S2053273326004651 (PMC13325189; doi:10.1107/S2053273326004651)
Supplement: Supplementary file 2 [file a-82-00256-sup2.pdf]

FOUNDATIONS
ADVANCES

Volume 82 (2026)

Supporting information for article:

From atoms to a data bank: optimizing transferability of electron-density symmetry

Paulina Maria Rybicka, Marta Kulik, Vladislav Ignat'ev and Paulina Maria Dominiak

Supporting information S2

The file contains Figures S2.1-S2.58 showing ridgeplots and tables with statistical data describing the distributions of multipole model parameters ($\kappa, P_{val}, \kappa', P_{lm}$) for different subgroups of atoms. Statistical metrics include absolute minimum, maximum, mean, median, and sample standard deviation (*ssd)*. Grey rectangles indicate parts of P_{lm} approximated as zero using zero-value thresholds that vary by subgroup.

IMPORTANT: this document contains embedded data - to preserve data integrity, please ensure where possible that the IUCr Word tools (available from http://journals.iucr.org/services/docxtemplate/) are installed when editing this document.

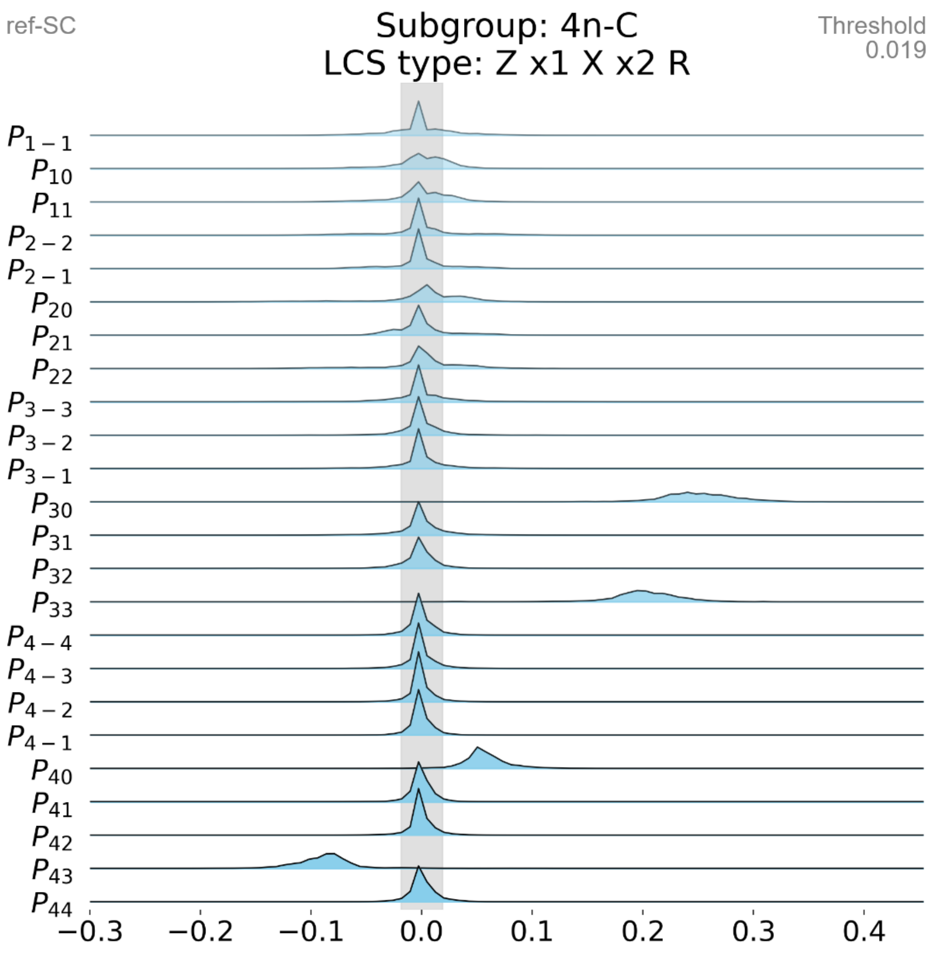

	min	max	median	mean	ssd
κ	0.9812	1.0513	1.0114	1.0119	0.0092
κ'	0.7901	1.1554	0.9200	0.9195	0.0265
P_{val}	3.025	4.333	3.733	3.730	0.155
P_{1-1}	-0.156	0.156	0.000	0.000	0.027
P_{10}	-0.177	0.158	0.002	0.000	0.028
P_{11}	-0.170	0.154	0.000	0.000	0.028
P_{2-2}	-0.175	0.175	0.000	0.000	0.034
P_{2-1}	-0.132	0.130	0.000	0.000	0.025
P_{20}	-0.250	0.181	0.005	0.001	0.044
P_{21}	-0.121	0.130	0.000	0.000	0.024
P_{22}	-0.190	0.143	0.001	0.001	0.035
P_{3-3}	-0.239	0.318	0.000	0.000	0.032
P_{3-2}	-0.190	0.288	0.000	0.000	0.021
P_{3-1}	-0.303	0.233	0.000	0.000	0.023
P_{30}	-0.226	0.457	0.249	0.249	0.039
P_{31}	-0.203	0.307	0.000	-0.000	0.022
P_{32}	-0.281	0.275	0.000	-0.002	0.027
P_{33}	-0.198	0.371	0.202	0.201	0.037
P_{4-4}	-0.111	0.098	0.000	0.000	0.012
P_{4-3}	-0.154	0.138	0.000	0.000	0.014
P_{4-2}	-0.119	0.146	0.000	0.000	0.010
P_{4-1}	-0.134	0.116	0.000	0.000	0.011
P_{40}	-0.070	0.212	0.055	0.057	0.020
P_{41}	-0.133	0.102	0.000	-0.000	0.010
P_{42}	-0.157	0.123	0.000	0.000	0.011
P_{43}	-0.245	0.199	-0.087	-0.087	0.027
P_{44}	-0.108	0.121	0.000	0.001	0.012

Figure S2.1 Ridgeplots for P_{lm} parameters (top) and statistical data for κ, κ', P_{val}, and P_{lm} parameters (bottom) for non-planar carbon atoms with four first neighbors (the 4n-C subgroup) for the refinement with symmetry constraints (ref-SC) in the Z x1 X x2 R LCS type. The grey rectangle shows parts of P_{lm} approximated as zero with threshold 0.019.

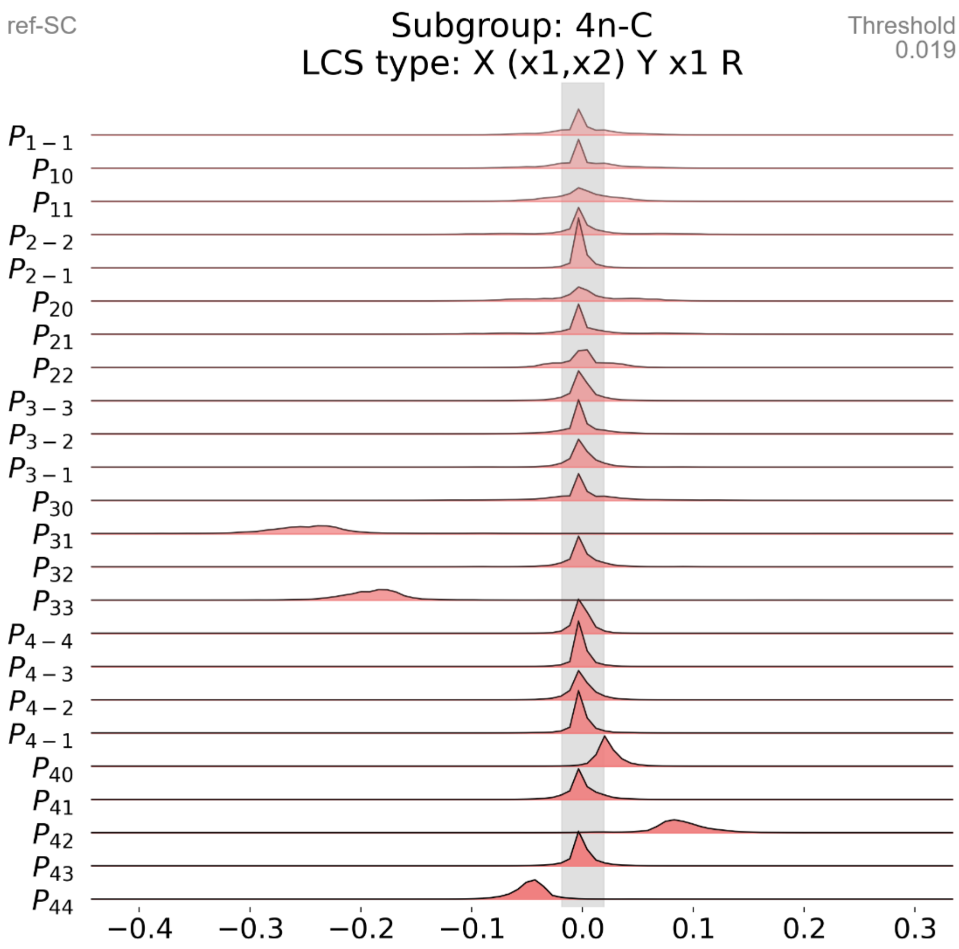

	min	max	median	mean	ssd
κ	0.9812	1.0513	1.0114	1.0119	0.0092
κ'	0.7901	1.1554	0.9200	0.9195	0.0265
P_{val}	3.025	4.333	3.733	3.730	0.155
P_{1-1}	-0.155	0.155	0.000	0.000	0.028
P_{10}	-0.156	0.156	0.000	0.000	0.027
P_{11}	-0.160	0.160	0.000	0.000	0.028
P_{2-2}	-0.218	0.218	0.000	0.000	0.042
P_{2-1}	-0.087	0.093	0.000	0.000	0.008
P_{20}	-0.180	0.180	-0.001	-0.001	0.039
P_{21}	-0.218	0.218	0.000	0.000	0.041
P_{22}	-0.115	0.117	0.000	0.000	0.021
P_{3-3}	-0.253	0.253	0.000	0.000	0.016
P_{3-2}	-0.207	0.245	0.000	0.000	0.021
P_{3-1}	-0.229	0.229	0.000	0.000	0.020
P_{30}	-0.274	0.274	0.000	0.000	0.036
P_{31}	-0.447	0.338	-0.246	-0.243	0.051
P_{32}	-0.243	0.243	0.000	0.000	0.021
P_{33}	-0.349	0.260	-0.188	-0.188	0.035
P_{4-4}	-0.130	0.130	0.000	0.000	0.009
P_{4-3}	-0.099	0.098	0.000	0.000	0.008
P_{4-2}	-0.127	0.127	0.000	0.000	0.012
P_{4-1}	-0.113	0.120	0.000	0.000	0.011
P_{40}	-0.092	0.147	0.021	0.023	0.012
P_{41}	-0.098	0.098	0.000	0.000	0.014
P_{42}	-0.082	0.240	0.087	0.088	0.025
P_{43}	-0.149	0.149	0.000	0.000	0.013
P_{44}	-0.168	0.121	-0.046	-0.048	0.016

Figure S2.2 Ridgeplots for P_{lm} parameters (top) and statistical data for κ, κ', P_{val}, and P_{lm} parameters (bottom) for non-planar carbon atoms with four first neighbors (the 4n-C subgroup) for the refinement with symmetry constraints (ref-SC) in the X (x1,x2) Y x1 LCS type. The grey rectangle shows parts of P_{lm} approximated as zero with threshold 0.019.

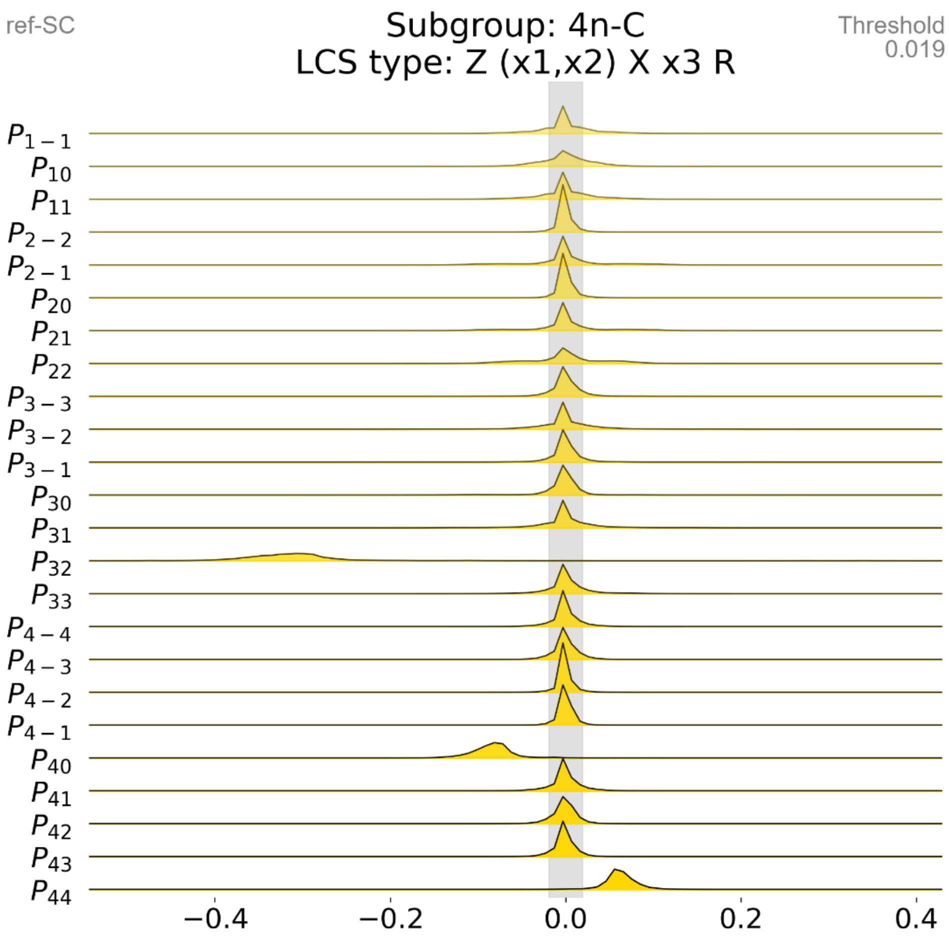

	min	max	median	mean	ssd
κ	0.9812	1.0513	1.0114	1.0119	0.0092
κ'	0.7901	1.1554	0.9200	0.9195	0.0265
P_{val}	3.025	4.333	3.733	3.730	0.155
P_{1-1}	-0.154	0.155	0.000	0.000	0.028
P_{10}	-0.160	0.160	0.000	0.000	0.028
P_{11}	-0.156	0.156	0.000	0.000	0.028
P_{2-2}	-0.094	0.096	0.000	0.000	0.009
P_{2-1}	-0.218	0.218	0.000	0.000	0.042
P_{20}	-0.093	0.091	0.000	0.001	0.009
P_{21}	-0.218	0.218	0.000	-0.000	0.041
P_{22}	-0.188	0.188	0.000	-0.000	0.042
P_{3-3}	-0.234	0.210	0.000	0.000	0.018
P_{3-2}	-0.328	0.319	0.000	0.000	0.038
P_{3-1}	-0.234	0.234	0.000	0.000	0.016
P_{30}	-0.307	0.261	-0.001	-0.004	0.022
P_{31}	-0.238	0.234	0.000	0.000	0.034
P_{32}	-0.548	0.434	-0.315	-0.312	0.062
P_{33}	-0.221	0.241	0.000	0.001	0.021
P_{4-4}	-0.120	0.122	0.000	0.000	0.014
P_{4-3}	-0.123	0.109	0.000	0.000	0.013
P_{4-2}	-0.106	0.091	0.000	0.000	0.008
P_{4-1}	-0.170	0.161	0.000	0.000	0.008
P_{40}	-0.233	0.193	-0.083	-0.084	0.026
P_{41}	-0.144	0.144	0.000	0.000	0.016
P_{42}	-0.113	0.105	-0.001	-0.001	0.014
P_{43}	-0.119	0.111	0.000	-0.001	0.011
P_{44}	-0.077	0.193	0.061	0.062	0.018

Figure S2.3 Ridgeplots for P_{lm} parameters (top) and statistical data for κ, κ', P_{val}, and P_{lm} parameters (bottom) for non-planar carbon atoms with four first neighbors (the 4n-C subgroup) for the refinement with symmetry constraints (ref-SC) in the Z (x1,x2) X x3 R LCS type. The grey rectangle shows parts of P_{lm} approximated as zero with threshold 0.019.

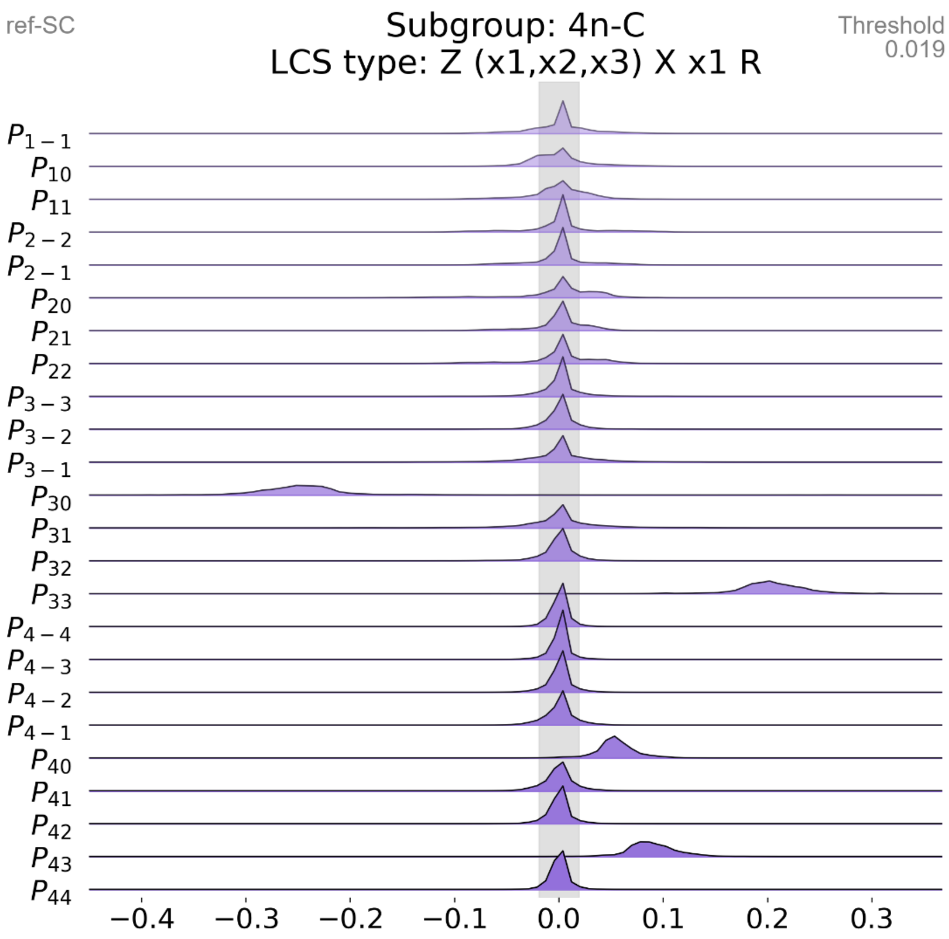

	min	max	median	mean	ssd
κ	0.9812	1.0513	1.0114	1.0119	0.0092
κ'	0.7901	1.1554	0.9200	0.9195	0.0265
P_{val}	3.025	4.333	3.733	3.730	0.155
P_{1-1}	-0.154	0.155	0.000	0.000	0.028
P_{10}	-0.157	0.177	-0.002	0.000	0.028
P_{11}	-0.164	0.151	0.000	0.000	0.028
P_{2-2}	-0.194	0.194	0.000	0.000	0.034
P_{2-1}	-0.145	0.145	0.000	0.000	0.026
P_{20}	-0.250	0.197	0.005	-0.000	0.043
P_{21}	-0.167	0.119	0.001	0.000	0.026
P_{22}	-0.181	0.167	0.001	0.000	0.034
P_{3-3}	-0.312	0.174	0.000	0.000	0.021
P_{3-2}	-0.226	0.354	0.000	0.000	0.018
P_{3-1}	-0.255	0.283	0.000	0.000	0.035
P_{30}	-0.454	0.343	-0.246	-0.244	0.050
P_{31}	-0.280	0.301	0.000	0.001	0.037
P_{32}	-0.288	0.339	0.000	-0.001	0.017
P_{33}	-0.187	0.371	0.203	0.204	0.032
P_{4-4}	-0.085	0.121	0.000	0.000	0.009
P_{4-3}	-0.190	0.135	0.000	0.000	0.009
P_{4-2}	-0.154	0.124	0.000	0.000	0.012
P_{4-1}	-0.158	0.119	0.000	0.000	0.015
P_{40}	-0.137	0.212	0.054	0.055	0.020
P_{41}	-0.136	0.139	0.000	-0.000	0.015
P_{42}	-0.161	0.165	0.000	-0.000	0.012
P_{43}	-0.116	0.263	0.088	0.089	0.024
P_{44}	-0.111	0.113	0.000	-0.000	0.009

Figure S2.4 Ridgeplots for P_{lm} parameters (top) and statistical data for κ, κ', P_{val}, and P_{lm} parameters (bottom) for non-planar carbon atoms with four first neighbors (the 4n-C subgroup) for the refinement with symmetry constraints (ref-SC) in the Z (x1,x2,x3) X x1 R LCS type. The grey rectangle shows parts of P_{lm} approximated as zero with threshold 0.019.

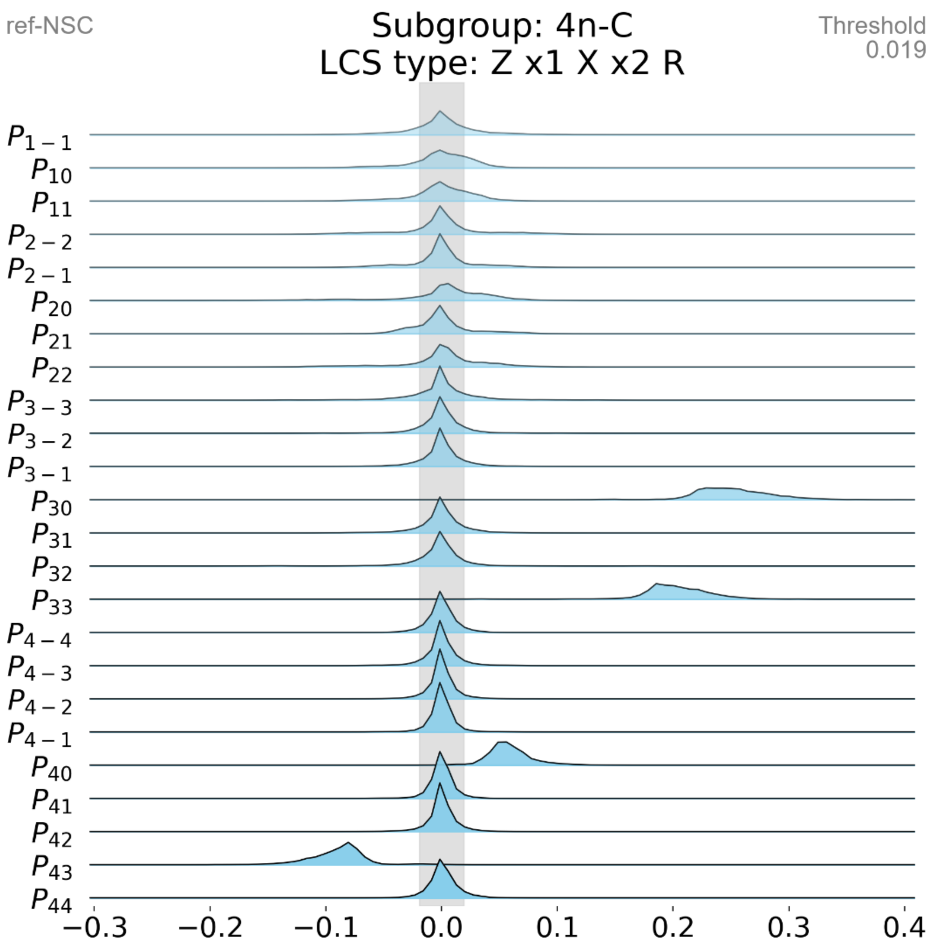

	min	max	median	mean	ssd
κ	0.9819	1.0349	1.0115	1.0118	0.0090
κ'	0.8142	1.0712	0.9193	0.9180	0.0231
P_{val}	3.370	4.198	3.724	3.727	0.153
P_{1-1}	-0.161	0.161	0.000	0.000	0.028
P_{10}	-0.177	0.143	0.002	-0.000	0.028
P_{11}	-0.172	0.156	0.001	-0.000	0.028
P_{2-2}	-0.179	0.178	0.000	-0.000	0.036
P_{2-1}	-0.132	0.133	0.000	0.000	0.026
P_{20}	-0.251	0.183	0.006	0.001	0.046
P_{21}	-0.122	0.131	-0.002	0.000	0.026
P_{22}	-0.190	0.153	0.003	0.001	0.037
P_{3-3}	-0.210	0.212	0.000	0.000	0.031
P_{3-2}	-0.273	0.297	0.000	-0.000	0.021
P_{3-1}	-0.235	0.219	0.000	-0.000	0.020
P_{30}	-0.229	0.412	0.249	0.252	0.034
P_{31}	-0.237	0.218	0.000	-0.001	0.020
P_{32}	-0.307	0.288	-0.001	-0.002	0.028
P_{33}	-0.204	0.342	0.201	0.202	0.033
P_{4-4}	-0.071	0.080	0.000	-0.000	0.012
P_{4-3}	-0.127	0.118	0.000	0.000	0.015
P_{4-2}	-0.093	0.095	0.000	0.000	0.012
P_{4-1}	-0.112	0.103	0.000	0.000	0.011
P_{40}	-0.083	0.166	0.056	0.059	0.019
P_{41}	-0.097	0.094	0.000	-0.000	0.011
P_{42}	-0.115	0.088	0.000	0.000	0.012
P_{43}	-0.211	0.070	-0.087	-0.089	0.024
P_{44}	-0.074	0.100	0.001	0.001	0.013

Figure S2.5 Ridgeplots for P_{lm} parameters (top) and statistical data for κ, κ', P_{val}, and P_{lm} parameters (bottom) for non-planar carbon atoms with four first neighbors (the 4n-C subgroup) for the refinement without symmetry constraints (ref-NSC) in the Z x1 X x2 R LCS type. The grey rectangle shows parts of P_{lm} approximated as zero with threshold 0.019.

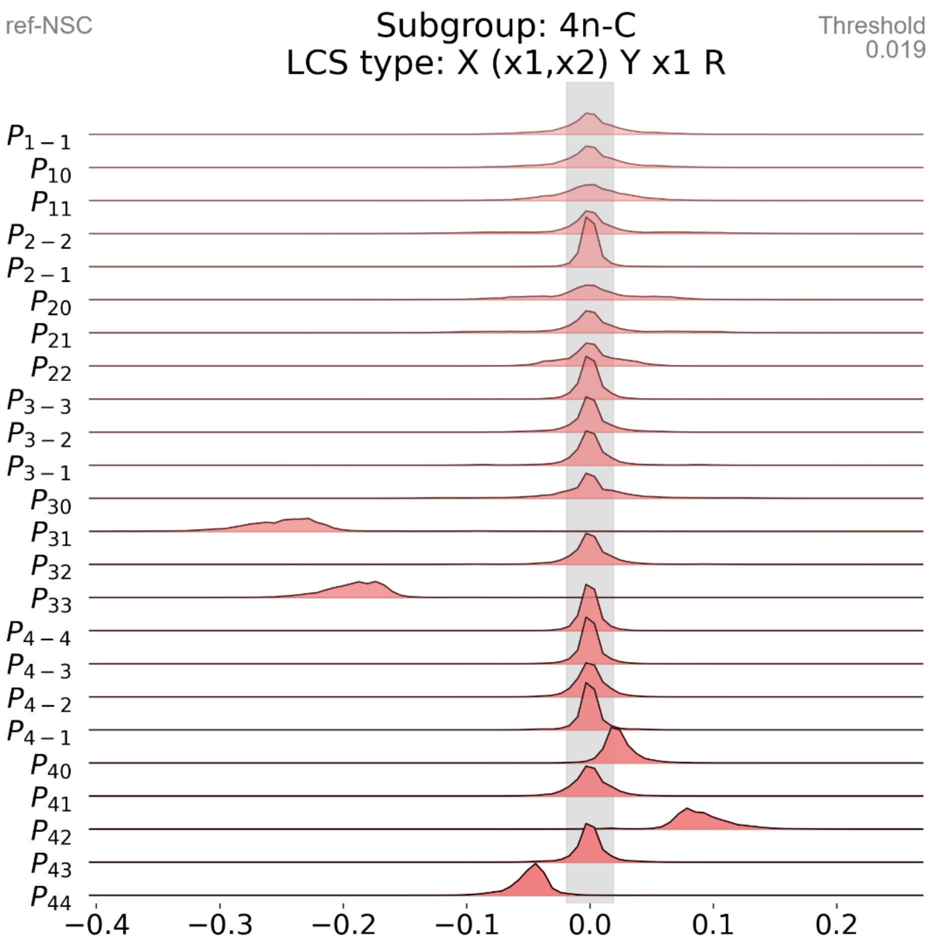

	min	max	median	mean	ssd
κ	0.9819	1.0349	1.0115	1.0118	0.0090
κ'	0.8142	1.0712	0.9193	0.9180	0.0231
P_{val}	3.370	4.198	3.724	3.727	0.153
P_{1-1}	-0.161	0.161	0.000	0.000	0.028
P_{10}	-0.161	0.161	0.000	0.000	0.028
P_{11}	-0.169	0.169	0.000	-0.000	0.028
P_{2-2}	-0.222	0.222	0.000	0.000	0.044
P_{2-1}	-0.077	0.076	0.000	-0.000	0.008
P_{20}	-0.187	0.188	-0.001	-0.001	0.041
P_{21}	-0.222	0.222	0.000	0.000	0.044
P_{22}	-0.126	0.114	0.000	0.000	0.023
P_{3-3}	-0.184	0.184	0.000	0.000	0.011
P_{3-2}	-0.280	0.252	0.000	-0.000	0.018
P_{3-1}	-0.236	0.236	0.000	0.000	0.019
P_{30}	-0.239	0.239	0.000	0.000	0.035
P_{31}	-0.409	0.196	-0.246	-0.247	0.039
P_{32}	-0.273	0.273	0.000	0.000	0.022
P_{33}	-0.340	0.188	-0.188	-0.191	0.025
P_{4-4}	-0.087	0.087	0.000	0.000	0.009
P_{4-3}	-0.098	0.091	0.000	-0.000	0.010
P_{4-2}	-0.102	0.102	0.000	0.000	0.012
P_{4-1}	-0.088	0.088	0.000	0.000	0.011
P_{40}	-0.096	0.144	0.022	0.023	0.013
P_{41}	-0.088	0.088	0.000	0.000	0.015
P_{42}	-0.069	0.216	0.087	0.090	0.024
P_{43}	-0.103	0.103	0.000	0.000	0.013
P_{44}	-0.153	0.059	-0.047	-0.049	0.015

Figure S2.6 Ridgeplots for P_{lm} parameters (top) and statistical data for κ, κ', P_{val}, and P_{lm} parameters (bottom) for non-planar carbon atoms with four first neighbors (the 4n-C subgroup) for the refinement without symmetry constraints (ref-NSC) in the X (x1,x2) Y x1 LCS type. The grey rectangle shows parts of P_{lm} approximated as zero with threshold 0.019.

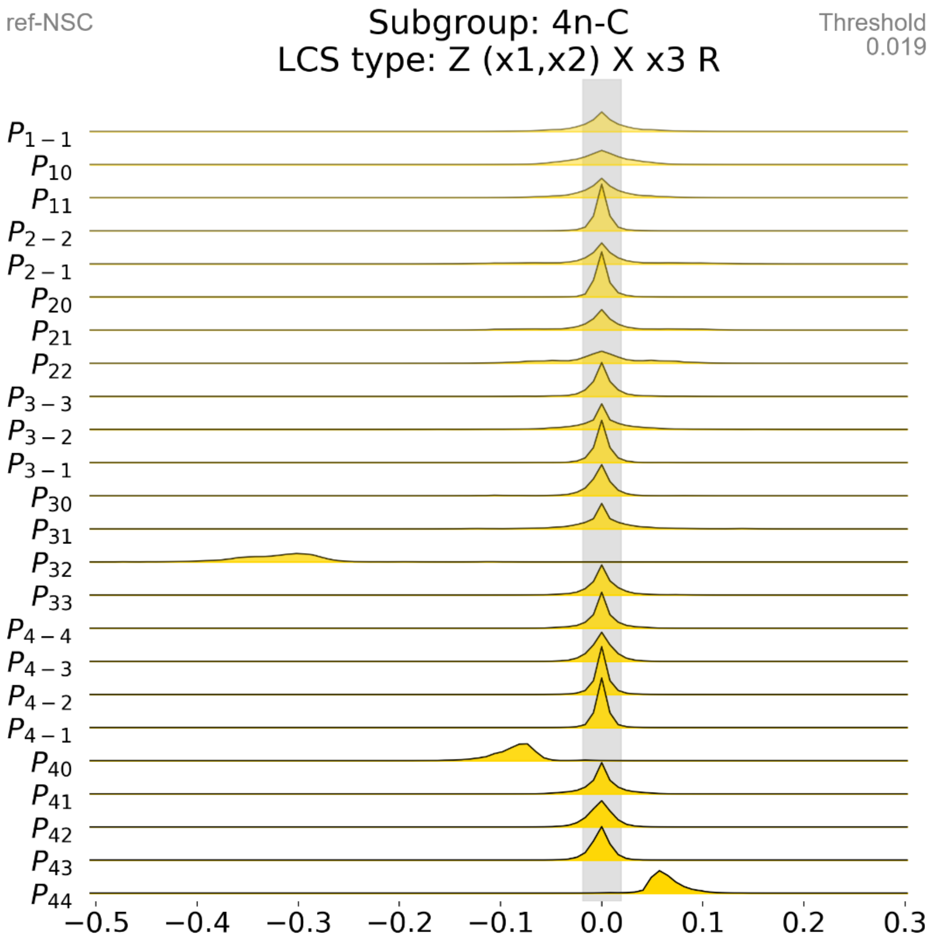

	min	max	median	mean	ssd
κ	0.9819	1.0349	1.0115	1.0118	0.0090
κ'	0.8142	1.0712	0.9193	0.9180	0.0231
P_{val}	3.370	4.198	3.724	3.727	0.153
P_{1-1}	-0.160	0.161	0.000	0.000	0.028
P_{10}	-0.169	0.169	0.000	-0.000	0.028
P_{11}	-0.161	0.161	0.000	-0.000	0.028
P_{2-2}	-0.100	0.105	0.000	-0.000	0.010
P_{2-1}	-0.222	0.222	0.000	0.000	0.044
P_{20}	-0.094	0.089	0.000	0.001	0.009
P_{21}	-0.222	0.222	0.000	-0.000	0.043
P_{22}	-0.201	0.204	0.000	-0.000	0.044
P_{3-3}	-0.202	0.202	0.000	-0.000	0.016
P_{3-2}	-0.297	0.306	0.000	0.000	0.038
P_{3-1}	-0.206	0.206	0.000	0.000	0.012
P_{30}	-0.340	0.239	-0.001	-0.004	0.023
P_{31}	-0.210	0.213	0.000	0.000	0.034
P_{32}	-0.510	0.183	-0.315	-0.317	0.046
P_{33}	-0.202	0.202	0.001	0.002	0.019
P_{4-4}	-0.104	0.128	0.000	-0.000	0.015
P_{4-3}	-0.085	0.084	0.000	-0.000	0.013
P_{4-2}	-0.101	0.101	0.000	0.000	0.009
P_{4-1}	-0.073	0.081	0.000	-0.000	0.008
P_{40}	-0.239	0.119	-0.083	-0.086	0.023
P_{41}	-0.115	0.115	0.000	0.000	0.017
P_{42}	-0.085	0.081	-0.001	-0.002	0.014
P_{43}	-0.089	0.088	-0.001	-0.001	0.011
P_{44}	-0.067	0.172	0.061	0.064	0.017

Figure S2.7 Ridgeplots for P_{lm} parameters (top) and statistical data for κ, κ', P_{val}, and P_{lm} parameters (bottom) for non-planar carbon atoms with four first neighbors (the 4n-C subgroup) for the refinement without symmetry constraints (ref-NSC) in the Z (x1,x2) X x3 R LCS type. The grey rectangle shows parts of P_{lm} approximated as zero with threshold 0.019.

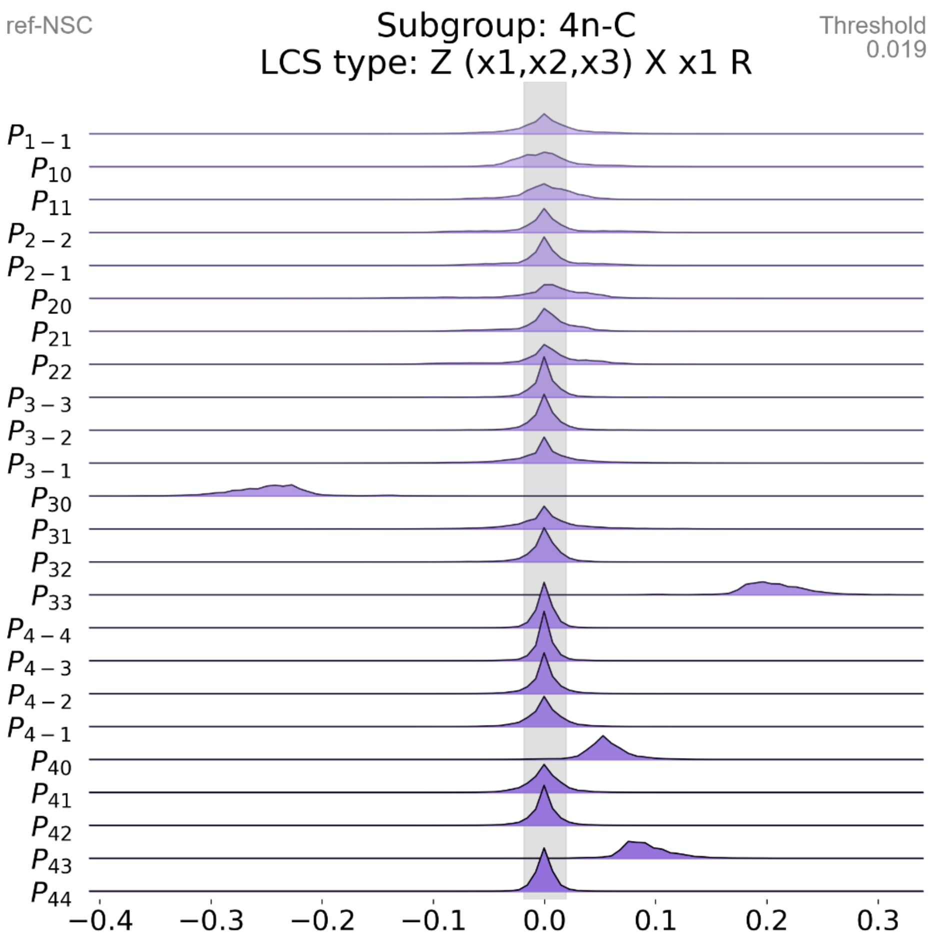

	min	max	median	mean	ssd
κ	0.9819	1.0349	1.0115	1.0118	0.0090
κ'	0.8142	1.0712	0.9193	0.9180	0.0231
P_{val}	3.370	4.198	3.724	3.727	0.153
P_{1-1}	-0.160	0.161	0.000	0.000	0.028
P_{10}	-0.149	0.177	-0.002	-0.000	0.028
P_{11}	-0.167	0.156	0.001	-0.000	0.028
P_{2-2}	-0.198	0.198	0.000	-0.000	0.036
P_{2-1}	-0.145	0.146	0.000	-0.000	0.027
P_{20}	-0.250	0.201	0.006	-0.001	0.046
P_{21}	-0.167	0.125	0.002	-0.000	0.027
P_{22}	-0.186	0.171	0.003	0.000	0.036
P_{3-3}	-0.196	0.199	0.000	-0.000	0.018
P_{3-2}	-0.280	0.296	0.000	0.000	0.017
P_{3-1}	-0.264	0.282	0.000	0.000	0.035
P_{30}	-0.413	0.237	-0.246	-0.247	0.038
P_{31}	-0.270	0.294	0.000	0.001	0.037
P_{32}	-0.294	0.344	0.000	-0.001	0.016
P_{33}	-0.206	0.342	0.204	0.206	0.028
P_{4-4}	-0.073	0.079	0.000	0.000	0.008
P_{4-3}	-0.096	0.089	0.000	-0.000	0.010
P_{4-2}	-0.119	0.113	0.000	-0.000	0.013
P_{4-1}	-0.106	0.108	0.000	-0.000	0.016
P_{40}	-0.087	0.158	0.054	0.055	0.019
P_{41}	-0.100	0.118	0.000	-0.000	0.016
P_{42}	-0.117	0.154	0.000	-0.000	0.013
P_{43}	-0.070	0.212	0.088	0.091	0.022
P_{44}	-0.077	0.073	0.000	-0.000	0.009

Figure S2.8 Ridgeplots for P_{lm} parameters (top) and statistical data for κ, κ', P_{val}, and P_{lm} parameters (bottom) for non-planar carbon atoms with four first neighbors (the 4n-C subgroup) for the refinement without symmetry constraints (ref-NSC) in the Z (x1,x2,x3) X x1 R LCS type. The grey rectangle shows parts of P_{lm} approximated as zero with threshold 0.019.

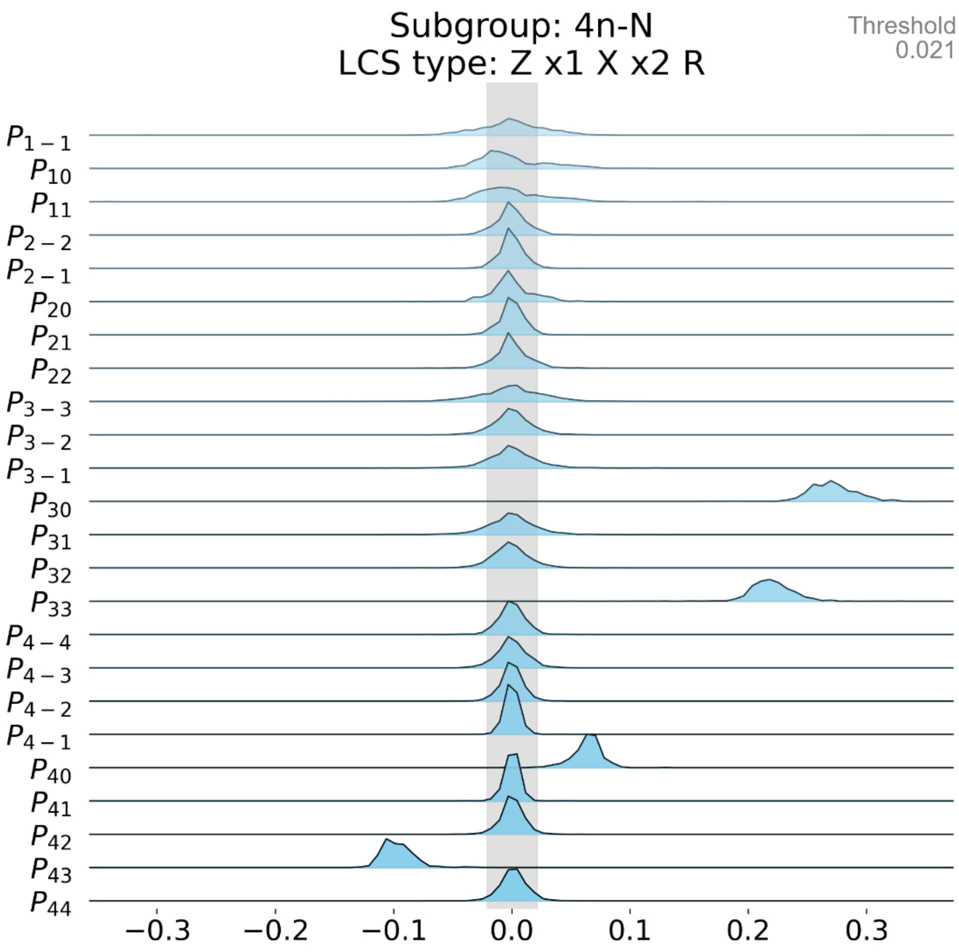

	min	max	median	mean	ssd
κ	0.9728	0.9968	0.9885	0.9864	0.0052
κ'	0.7583	0.8420	0.8056	0.8032	0.0190
P_{val}	5.012	5.352	5.146	5.158	0.071
P_{1-1}	-0.309	0.309	0.000	-0.000	0.031
P_{10}	-0.360	0.118	-0.007	-0.001	0.031
P_{11}	-0.347	0.173	-0.004	-0.000	0.031
P_{2-2}	-0.076	0.075	0.000	0.000	0.014
P_{2-1}	-0.045	0.054	-0.000	0.000	0.010
P_{20}	-0.089	0.085	-0.001	0.000	0.018
P_{21}	-0.050	0.055	0.000	0.000	0.010
P_{22}	-0.076	0.080	-0.000	0.000	0.014
P_{3-3}	-0.153	0.137	0.001	0.000	0.029
P_{3-2}	-0.065	0.065	-0.000	-0.000	0.016
P_{3-1}	-0.113	0.120	-0.000	-0.000	0.021
P_{30}	0.166	0.377	0.269	0.271	0.020
P_{31}	-0.103	0.137	-0.000	-0.001	0.021
P_{32}	-0.089	0.090	-0.001	-0.001	0.017
P_{33}	0.050	0.329	0.219	0.220	0.018
P_{4-4}	-0.069	0.062	0.000	0.000	0.011
P_{4-3}	-0.058	0.050	-0.000	0.000	0.013
P_{4-2}	-0.050	0.042	0.000	-0.000	0.009
P_{4-1}	-0.053	0.044	0.000	-0.000	0.006
P_{40}	-0.004	0.130	0.065	0.064	0.012
P_{41}	-0.042	0.052	0.000	0.000	0.006
P_{42}	-0.049	0.054	0.000	0.000	0.010
P_{43}	-0.126	-0.020	-0.098	-0.097	0.013
P_{44}	-0.071	0.066	0.001	0.002	0.011

Figure S2.9 Ridgeplots for P_{lm} parameters (top) and statistical data for κ, κ', P_{val}, and P_{lm} parameters (bottom) for non-planar nitrogen atoms with four first neighbors (the 4n-N subgroup) in the Z x1 X x2 R LCS type. The grey rectangle shows parts of P_{lm} approximated as zero with threshold 0.021.

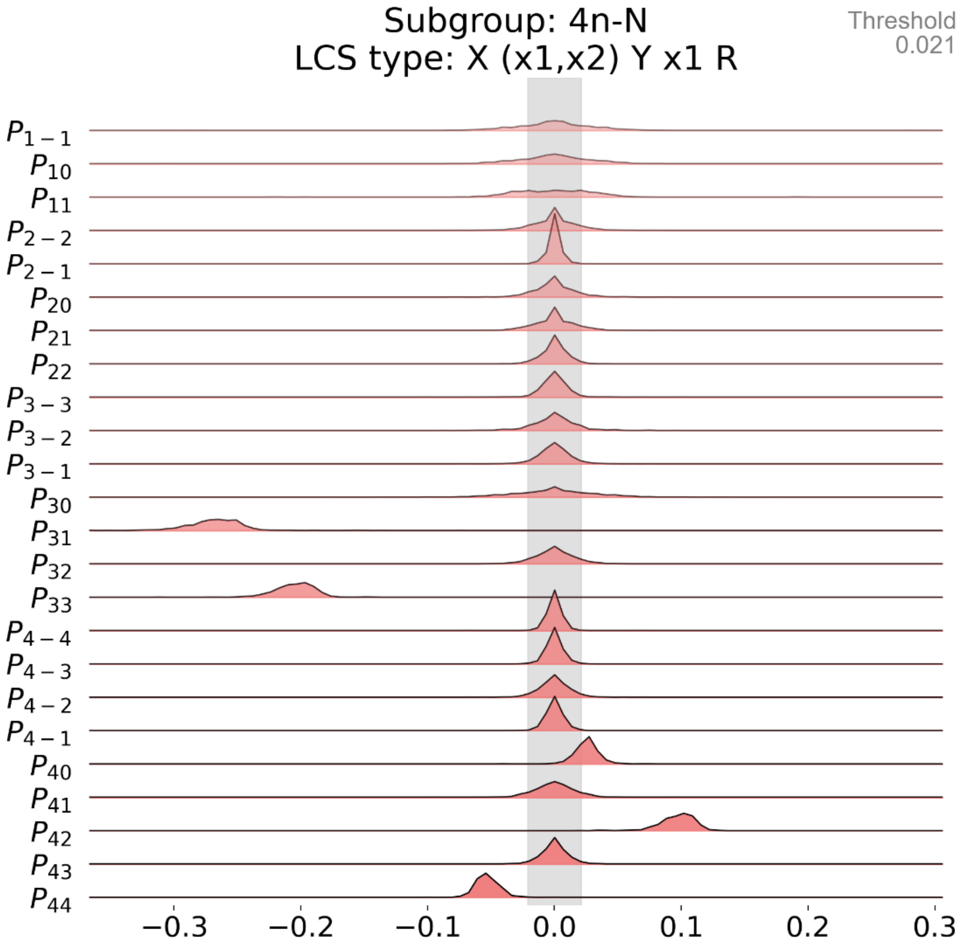

	min	max	median	mean	ssd
κ	0.9728	0.9968	0.9885	0.9864	0.0052
κ'	0.7583	0.8420	0.8056	0.8032	0.0190
P_{val}	5.012	5.352	5.146	5.158	0.071
P_{1-1}	-0.303	0.303	0.000	0.000	0.031
P_{10}	-0.309	0.309	0.000	0.000	0.031
P_{11}	-0.227	0.199	-0.001	-0.001	0.031
P_{2-2}	-0.090	0.090	0.000	0.000	0.017
P_{2-1}	-0.025	0.027	0.000	0.000	0.005
P_{20}	-0.079	0.087	-0.000	-0.000	0.016
P_{21}	-0.089	0.089	0.000	0.000	0.016
P_{22}	-0.040	0.041	0.000	0.000	0.009
P_{3-3}	-0.103	0.103	0.000	0.000	0.010
P_{3-2}	-0.087	0.092	0.000	0.000	0.017
P_{3-1}	-0.072	0.072	0.000	0.000	0.012
P_{30}	-0.174	0.174	0.000	0.000	0.035
P_{31}	-0.370	-0.077	-0.266	-0.267	0.022
P_{32}	-0.053	0.053	0.000	0.000	0.015
P_{33}	-0.310	-0.139	-0.203	-0.205	0.015
P_{4-4}	-0.070	0.070	0.000	0.000	0.006
P_{4-3}	-0.027	0.028	-0.000	0.000	0.006
P_{4-2}	-0.067	0.067	0.000	0.000	0.012
P_{4-1}	-0.034	0.022	-0.000	-0.000	0.007
P_{40}	-0.040	0.073	0.026	0.025	0.009
P_{41}	-0.093	0.093	0.000	0.000	0.015
P_{42}	0.019	0.127	0.099	0.097	0.013
P_{43}	-0.037	0.037	0.000	0.000	0.010
P_{44}	-0.102	0.002	-0.054	-0.053	0.009

Figure S2.10 Ridgeplots for P_{lm} parameters (top) and statistical data for κ, κ', P_{val}, and P_{lm} parameters (bottom) for non-planar nitrogen atoms with four first neighbors (the 4n-N subgroup) in the X (x1,x2) Y x1 R LCS type. The grey rectangle shows parts of P_{lm} approximated as zero with threshold 0.021.

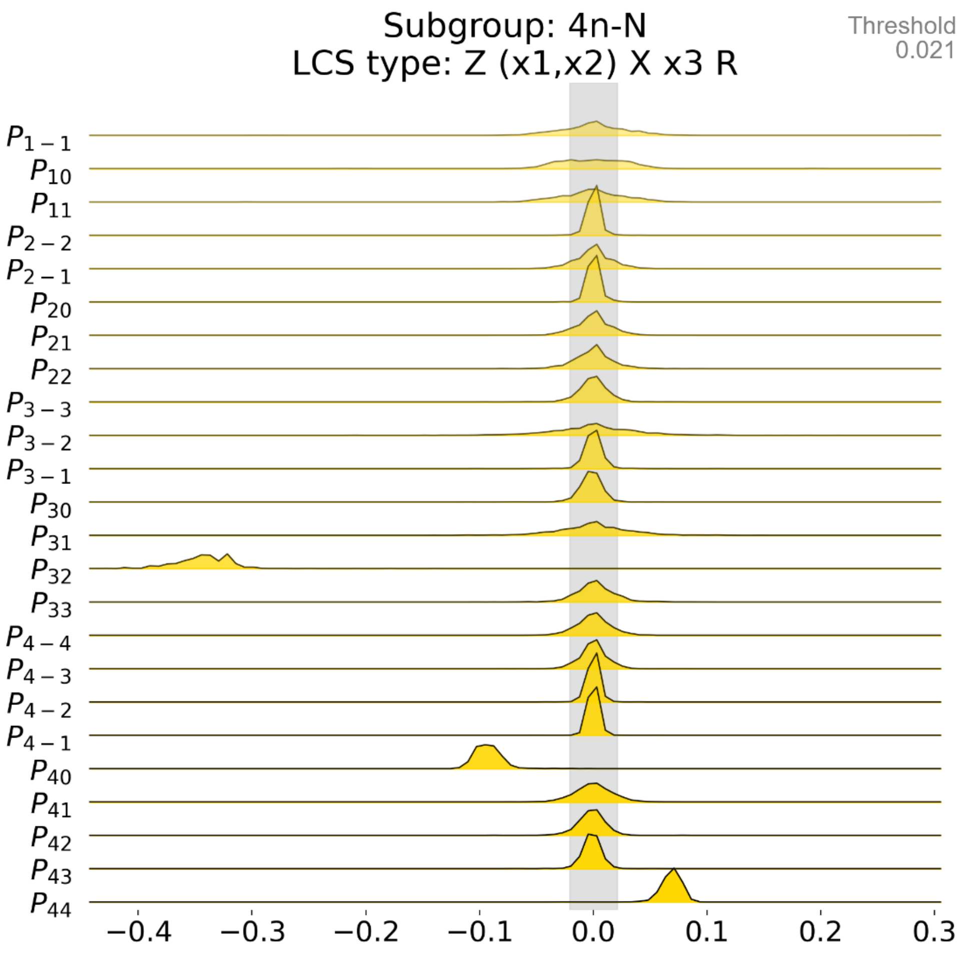

	min	max	median	mean	ssd
κ	0.9728	0.9968	0.9885	0.9864	0.0052
κ'	0.7583	0.8420	0.8056	0.8032	0.0190
P_{val}	5.012	5.352	5.146	5.158	0.071
P_{1-1}	-0.302	0.303	0.000	0.000	0.031
P_{10}	-0.227	0.199	-0.001	-0.001	0.031
P_{11}	-0.308	0.309	-0.001	-0.000	0.031
P_{2-2}	-0.031	0.043	-0.000	-0.000	0.005
P_{2-1}	-0.093	0.087	-0.000	0.000	0.017
P_{20}	-0.031	0.031	-0.000	0.000	0.006
P_{21}	-0.089	0.089	-0.000	-0.000	0.016
P_{22}	-0.083	0.090	-0.000	-0.000	0.017
P_{3-3}	-0.096	0.078	0.000	-0.000	0.013
P_{3-2}	-0.214	0.216	0.000	0.000	0.038
P_{3-1}	-0.106	0.105	-0.000	0.000	0.008
P_{30}	-0.107	0.060	-0.002	-0.002	0.011
P_{31}	-0.141	0.144	0.000	0.000	0.031
P_{32}	-0.446	-0.105	-0.341	-0.343	0.027
P_{33}	-0.099	0.100	0.001	0.002	0.017
P_{4-4}	-0.054	0.052	-0.000	0.000	0.014
P_{4-3}	-0.077	0.077	0.000	-0.000	0.012
P_{4-2}	-0.046	0.039	0.000	-0.000	0.005
P_{4-1}	-0.049	0.053	0.000	0.000	0.005
P_{40}	-0.118	-0.003	-0.094	-0.093	0.012
P_{41}	-0.072	0.071	-0.000	0.000	0.016
P_{42}	-0.086	0.081	-0.001	-0.002	0.012
P_{43}	-0.076	0.059	-0.001	-0.001	0.008
P_{44}	-0.031	0.090	0.069	0.068	0.010

Figure S2.11 Ridgeplots for P_{lm} parameters (top) and statistical data for κ, κ', P_{val}, and P_{lm} parameters (bottom) for non-planar nitrogen atoms with four first neighbors (the 4n-N subgroup) in the Z (x1,x2) X x3 R LCS type. The grey rectangle shows parts of P_{lm} approximated as zero with threshold 0.021.

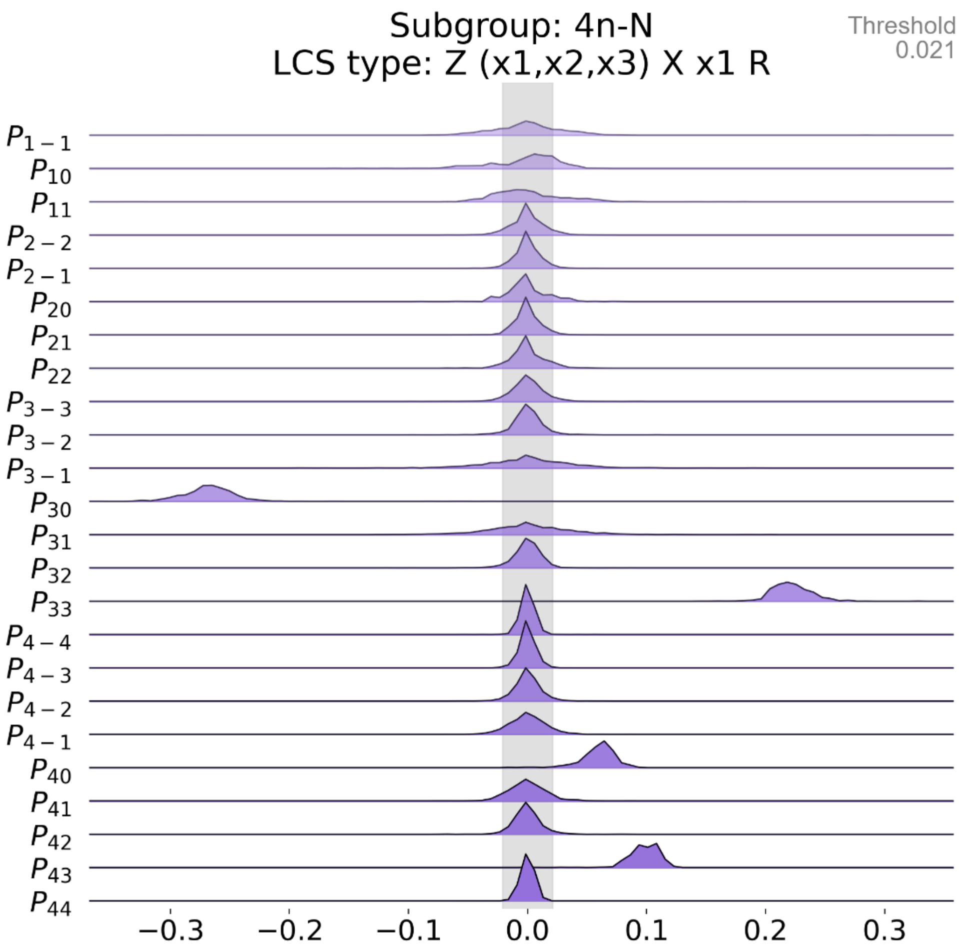

	min	max	median	mean	ssd
κ	0.9728	0.9968	0.9885	0.9864	0.0052
κ'	0.7583	0.8420	0.8056	0.8032	0.0190
P_{val}	5.012	5.352	5.146	5.158	0.071
P_{1-1}	-0.302	0.303	0.000	0.000	0.031
P_{10}	-0.163	0.361	0.004	-0.001	0.031
P_{11}	-0.342	0.182	-0.004	-0.000	0.031
P_{2-2}	-0.067	0.068	0.000	-0.000	0.014
P_{2-1}	-0.063	0.056	0.000	-0.000	0.011
P_{20}	-0.086	0.079	-0.001	-0.000	0.017
P_{21}	-0.063	0.059	-0.000	-0.000	0.011
P_{22}	-0.068	0.066	-0.000	0.000	0.014
P_{3-3}	-0.098	0.096	-0.000	-0.000	0.015
P_{3-2}	-0.096	0.111	0.000	0.000	0.013
P_{3-1}	-0.195	0.193	0.000	0.000	0.036
P_{30}	-0.371	-0.029	-0.267	-0.267	0.026
P_{31}	-0.127	0.206	-0.000	0.001	0.035
P_{32}	-0.117	0.084	0.000	-0.000	0.012
P_{33}	0.145	0.330	0.220	0.222	0.016
P_{4-4}	-0.053	0.057	-0.000	-0.000	0.006
P_{4-3}	-0.031	0.039	-0.000	0.000	0.007
P_{4-2}	-0.059	0.066	-0.000	0.000	0.011
P_{4-1}	-0.068	0.087	-0.000	-0.000	0.015
P_{40}	-0.017	0.097	0.061	0.059	0.014
P_{41}	-0.075	0.086	-0.001	-0.000	0.015
P_{42}	-0.071	0.087	-0.001	-0.000	0.012
P_{43}	-0.002	0.126	0.099	0.098	0.013
P_{44}	-0.050	0.052	0.000	-0.000	0.006

Figure S2.12 Ridgeplots for P_{lm} parameters (top) and statistical data for κ, κ', P_{val}, and P_{lm} parameters (bottom) for non-planar nitrogen atoms with four first neighbors (the 4n-N subgroup) in the Z (x1,x2,x3) X x1 R LCS type. The grey rectangle shows parts of P_{lm} approximated as zero with threshold 0.021.

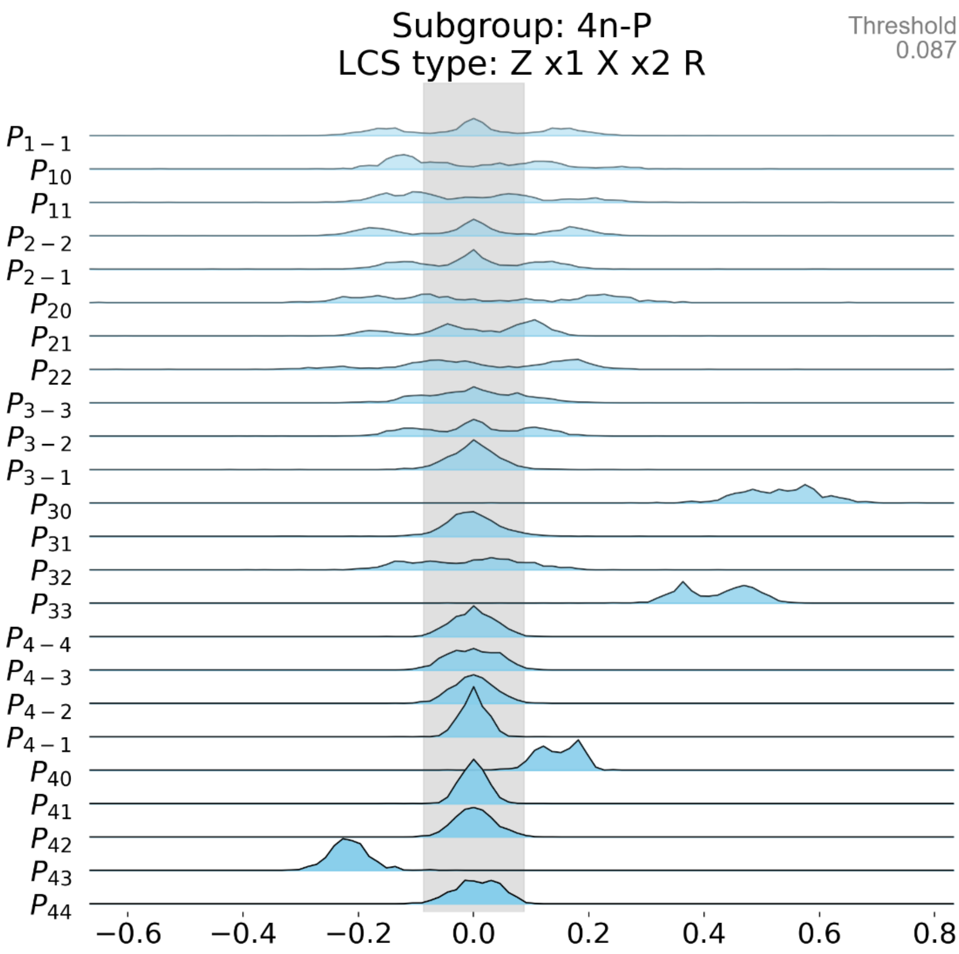

	min	max	median	mean	ssd
κ	0.9308	0.9677	0.9438	0.9438	0.0053
κ'	1.0000	1.1232	1.0358	1.0381	0.0159
P_{val}	5.014	5.542	5.284	5.287	0.081
P_{1-1}	-0.622	0.622	0.000	0.000	0.126
P_{10}	-0.541	0.511	-0.048	-0.009	0.131
P_{11}	-0.589	0.616	-0.015	-0.006	0.130
P_{2-2}	-0.570	0.820	0.000	-0.000	0.139
P_{2-1}	-0.673	0.598	-0.001	0.000	0.105
P_{20}	-0.645	0.647	-0.035	0.014	0.182
P_{21}	-0.599	0.692	0.016	0.005	0.109
P_{22}	-0.617	0.840	-0.005	0.014	0.142
P_{3-3}	-0.255	0.479	0.000	0.000	0.082
P_{3-2}	-0.403	0.321	0.001	0.000	0.093
P_{3-1}	-0.428	0.298	0.001	-0.000	0.045
P_{30}	0.025	0.771	0.536	0.532	0.070
P_{31}	-0.334	0.401	-0.004	-0.001	0.047
P_{32}	-0.396	0.421	-0.000	-0.010	0.092
P_{33}	-0.042	0.571	0.430	0.420	0.065
P_{4-4}	-0.153	0.124	0.000	0.000	0.036
P_{4-3}	-0.117	0.124	-0.000	-0.000	0.043
P_{4-2}	-0.136	0.125	-0.000	-0.000	0.036
P_{4-1}	-0.141	0.128	0.000	-0.000	0.023
P_{40}	-0.051	0.247	0.150	0.147	0.037
P_{41}	-0.149	0.126	0.000	0.000	0.023
P_{42}	-0.102	0.139	0.002	0.002	0.034
P_{43}	-0.320	-0.034	-0.217	-0.216	0.034
P_{44}	-0.128	0.113	0.008	0.007	0.039

Figure S2.13 Ridgeplots for P_{lm} parameters (top) and statistical data for κ, κ', P_{val}, and P_{lm} parameters (bottom) for non-planar phosphorus atoms with four first neighbors (the 4n-P subgroup) in the Z x1 X x2 R LCS type. The grey rectangle shows parts of P_{lm} approximated as zero with threshold 0.087.

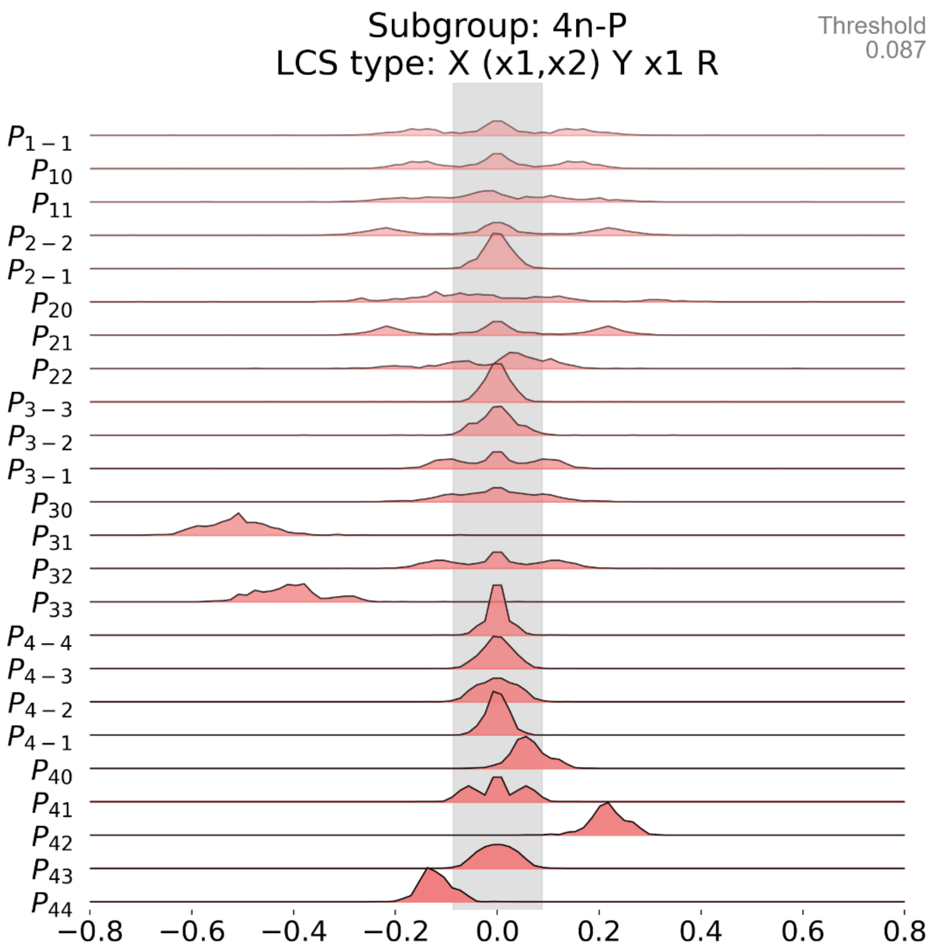

	min	max	median	mean	ssd
κ	0.9308	0.9677	0.9438	0.9438	0.0053
κ'	1.0000	1.1232	1.0358	1.0381	0.0159
P_{val}	5.014	5.542	5.284	5.287	0.081
P_{1-1}	-0.635	0.635	0.000	0.000	0.130
P_{10}	-0.622	0.622	0.000	0.000	0.126
P_{11}	-0.579	0.599	-0.017	-0.010	0.131
P_{2-2}	-0.726	0.726	0.000	0.000	0.174
P_{2-1}	-0.617	0.328	-0.000	-0.000	0.041
P_{20}	-0.697	0.624	-0.041	-0.020	0.156
P_{21}	-0.807	0.807	0.000	0.000	0.169
P_{22}	-0.470	0.583	0.024	0.003	0.098
P_{3-3}	-0.440	0.440	0.000	0.000	0.030
P_{3-2}	-0.383	0.340	0.000	0.000	0.042
P_{3-1}	-0.562	0.562	0.000	0.000	0.083
P_{30}	-0.403	0.403	0.000	0.000	0.087
P_{31}	-0.692	-0.076	-0.512	-0.513	0.062
P_{32}	-0.328	0.328	0.000	0.000	0.097
P_{33}	-0.571	0.034	-0.408	-0.406	0.067
P_{4-4}	-0.115	0.115	0.000	0.000	0.021
P_{4-3}	-0.113	0.131	0.000	0.000	0.030
P_{4-2}	-0.130	0.130	0.000	0.000	0.037
P_{4-1}	-0.068	0.113	-0.000	-0.000	0.022
P_{40}	-0.060	0.188	0.060	0.062	0.034
P_{41}	-0.155	0.155	0.000	0.000	0.049
P_{42}	0.064	0.314	0.216	0.218	0.035
P_{43}	-0.128	0.128	0.000	0.000	0.036
P_{44}	-0.235	0.028	-0.124	-0.120	0.031

Figure S2.14 Ridgeplots for P_{lm} parameters (top) and statistical data for κ, κ', P_{val}, and P_{lm} parameters (bottom) for non-planar phosphorus atoms with four first neighbors (the 4n-P subgroup) in the X (x1,x2) Y x1 R LCS type. The grey rectangle shows parts of P_{lm} approximated as zero with threshold 0.087.

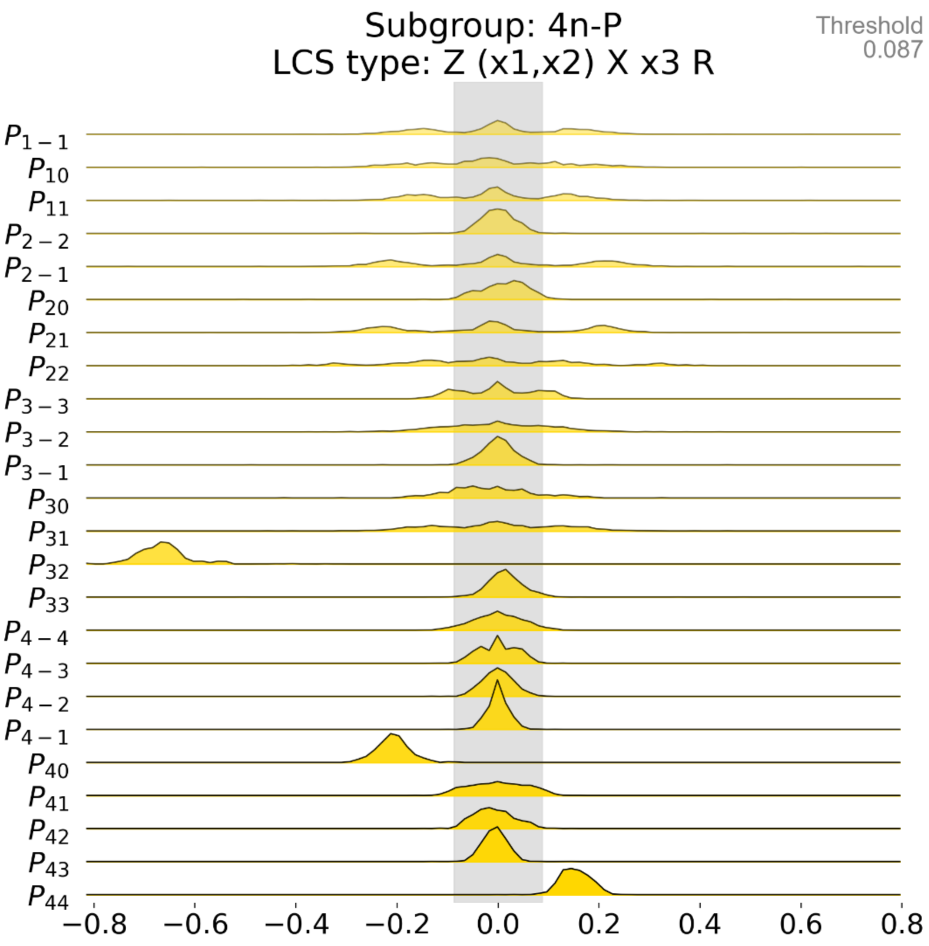

	min	max	median	mean	ssd
κ	0.9308	0.9677	0.9438	0.9438	0.0053
κ'	1.0000	1.1232	1.0358	1.0381	0.0159
P_{val}	5.014	5.542	5.284	5.287	0.081
P_{1-1}	-0.630	0.616	-0.000	-0.000	0.131
P_{10}	-0.579	0.599	-0.017	-0.010	0.131
P_{11}	-0.649	0.612	-0.007	-0.008	0.126
P_{2-2}	-0.333	0.651	-0.001	-0.000	0.045
P_{2-1}	-0.734	0.721	0.001	0.000	0.171
P_{20}	-0.506	0.564	0.018	0.013	0.054
P_{21}	-0.801	0.804	-0.010	-0.011	0.172
P_{22}	-0.702	0.689	-0.020	-0.013	0.170
P_{3-3}	-0.475	0.592	-0.000	0.000	0.075
P_{3-2}	-0.379	0.439	-0.000	-0.000	0.102
P_{3-1}	-0.483	0.452	-0.000	-0.000	0.037
P_{30}	-0.417	0.331	-0.024	-0.017	0.081
P_{31}	-0.461	0.457	-0.001	-0.001	0.121
P_{32}	-0.822	-0.305	-0.666	-0.663	0.054
P_{33}	-0.509	0.487	0.013	0.015	0.039
P_{4-4}	-0.143	0.143	-0.000	0.000	0.048
P_{4-3}	-0.124	0.136	0.000	0.000	0.038
P_{4-2}	-0.139	0.097	-0.000	-0.000	0.030
P_{4-1}	-0.106	0.108	0.000	0.000	0.020
P_{40}	-0.299	-0.075	-0.208	-0.208	0.032
P_{41}	-0.170	0.167	0.000	0.000	0.055
P_{42}	-0.151	0.139	-0.012	-0.008	0.039
P_{43}	-0.133	0.152	-0.006	-0.007	0.024
P_{44}	0.001	0.253	0.152	0.153	0.029

Figure S2.15 Ridgeplots for P_{lm} parameters (top) and statistical data for κ, κ', P_{val}, and P_{lm} parameters (bottom) for non-planar phosphorus atoms with four first neighbors (the 4n-P subgroup) in the Z (x1,x2) X x3 R LCS type. The grey rectangle shows parts of P_{lm} approximated as zero with threshold 0.087.

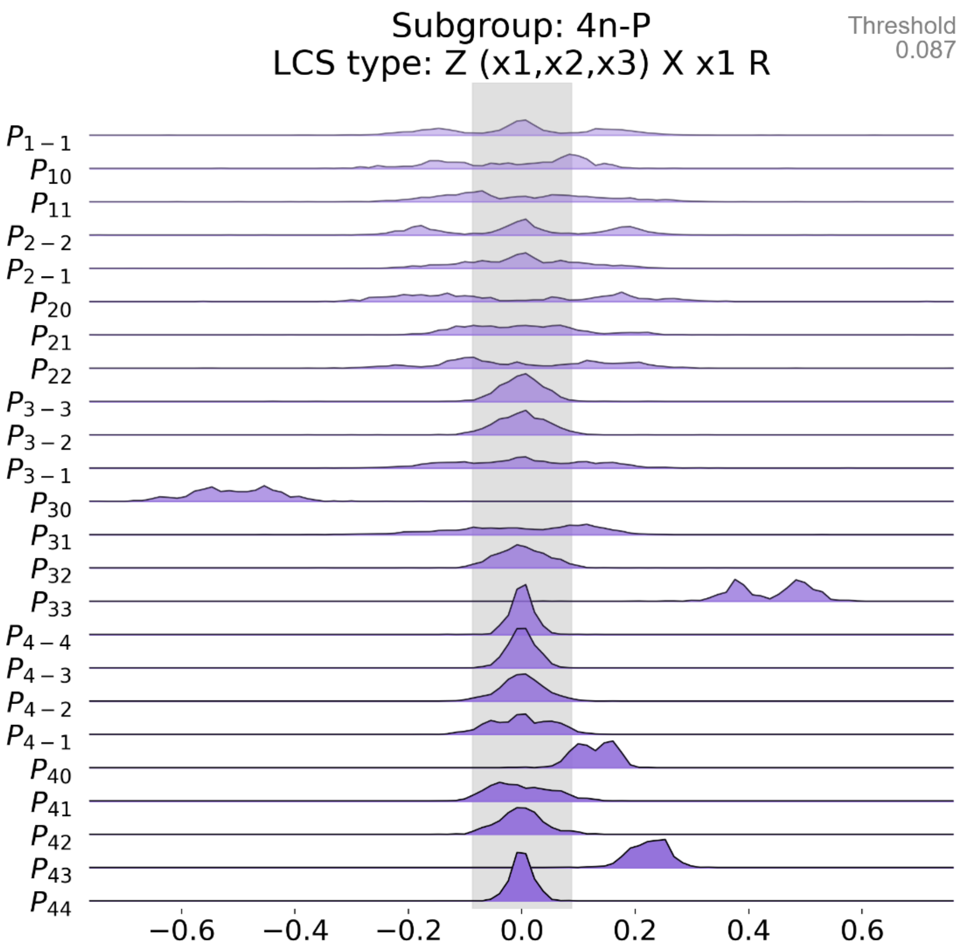

	min	max	median	mean	ssd
κ	0.9308	0.9677	0.9438	0.9438	0.0053
κ'	1.0000	1.1232	1.0358	1.0381	0.0159
P_{val}	5.014	5.542	5.284	5.287	0.081
P_{1-1}	-0.630	0.616	-0.000	-0.000	0.131
P_{10}	-0.531	0.475	0.012	-0.016	0.125
P_{11}	-0.603	0.589	-0.014	-0.002	0.132
P_{2-2}	-0.740	0.546	-0.000	-0.000	0.144
P_{2-1}	-0.682	0.663	-0.000	-0.000	0.102
P_{20}	-0.563	0.711	-0.077	-0.021	0.181
P_{21}	-0.756	0.681	-0.005	-0.001	0.102
P_{22}	-0.560	0.768	-0.015	0.005	0.144
P_{3-3}	-0.441	0.236	0.001	0.000	0.041
P_{3-2}	-0.379	0.335	0.001	0.000	0.047
P_{3-1}	-0.354	0.571	-0.001	0.000	0.116
P_{30}	-0.769	-0.095	-0.505	-0.506	0.076
P_{31}	-0.453	0.528	0.012	0.002	0.117
P_{32}	-0.359	0.368	-0.001	0.000	0.048
P_{33}	0.030	0.593	0.459	0.440	0.066
P_{4-4}	-0.126	0.128	0.000	-0.000	0.021
P_{4-3}	-0.081	0.082	-0.000	0.000	0.024
P_{4-2}	-0.128	0.162	-0.000	0.000	0.039
P_{4-1}	-0.181	0.150	-0.000	-0.000	0.053
P_{40}	-0.016	0.219	0.128	0.127	0.035
P_{41}	-0.140	0.180	-0.007	-0.001	0.054
P_{42}	-0.154	0.136	-0.002	-0.001	0.039
P_{43}	0.055	0.334	0.226	0.223	0.034
P_{44}	-0.141	0.086	-0.000	0.000	0.021

Figure S2.16 Ridgeplots for P_{lm} parameters (top) and statistical data for κ, κ', P_{val}, and P_{lm} parameters (bottom) for non-planar phosphorus atoms with four first neighbors (the 4n-P subgroup) in the Z (x1,x2,x3) X x1 R LCS type. The grey rectangle shows parts of P_{lm} approximated as zero with threshold 0.087.

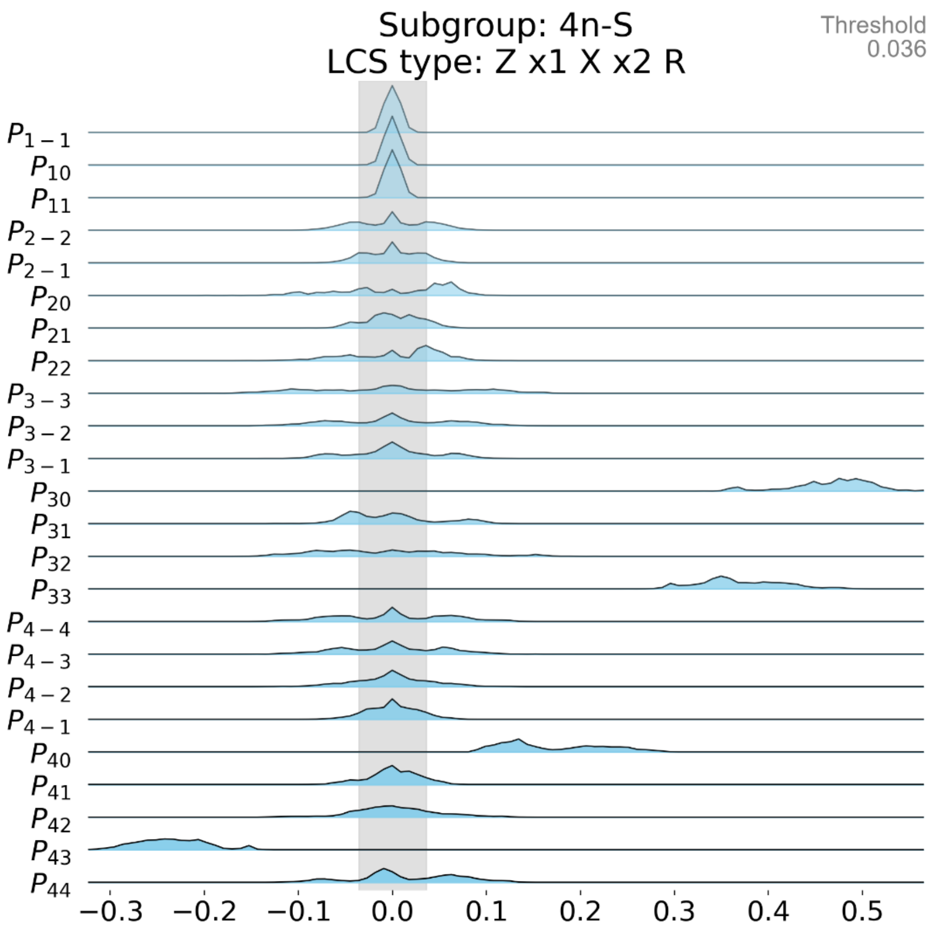

	min	max	median	mean	ssd
κ	0.9569	0.9732	0.9639	0.9644	0.0031
κ'	1.0024	1.1729	1.0428	1.0467	0.0279
P_{val}	5.779	6.108	5.968	5.965	0.058
P_{1-1}	-0.026	0.026	0.000	0.000	0.008
P_{10}	-0.025	0.023	-0.001	0.000	0.008
P_{11}	-0.025	0.023	-0.000	0.000	0.008
P_{2-2}	-0.112	0.109	-0.000	0.000	0.040
P_{2-1}	-0.086	0.074	0.000	-0.000	0.029
P_{20}	-0.198	0.118	0.023	0.005	0.058
P_{21}	-0.090	0.077	0.001	0.002	0.028
P_{22}	-0.160	0.124	0.021	0.006	0.047
P_{3-3}	-0.219	0.193	0.000	0.000	0.079
P_{3-2}	-0.147	0.161	-0.000	-0.000	0.060
P_{3-1}	-0.132	0.127	-0.000	-0.000	0.044
P_{30}	0.333	0.569	0.476	0.468	0.043
P_{31}	-0.129	0.124	-0.007	-0.002	0.046
P_{32}	-0.162	0.215	-0.010	-0.008	0.069
P_{33}	0.255	0.498	0.362	0.370	0.044
P_{4-4}	-0.143	0.146	-0.000	0.000	0.059
P_{4-3}	-0.157	0.189	0.000	-0.000	0.052
P_{4-2}	-0.142	0.134	-0.000	-0.000	0.041
P_{4-1}	-0.087	0.076	-0.000	-0.000	0.026
P_{40}	0.087	0.295	0.153	0.170	0.053
P_{41}	-0.093	0.072	0.001	0.001	0.027
P_{42}	-0.139	0.139	0.001	0.003	0.043
P_{43}	-0.328	-0.139	-0.237	-0.235	0.036
P_{44}	-0.123	0.146	0.005	0.016	0.054

Figure S2.17 Ridgeplots for P_{lm} parameters (top) and statistical data for κ, κ', P_{val}, and P_{lm} parameters (bottom) for non-planar sulfur atoms with four first neighbors (the 4n-S subgroup) in the Z x1 X x2 R LCS type. The grey rectangle shows parts of P_{lm} approximated as zero with threshold 0.036.

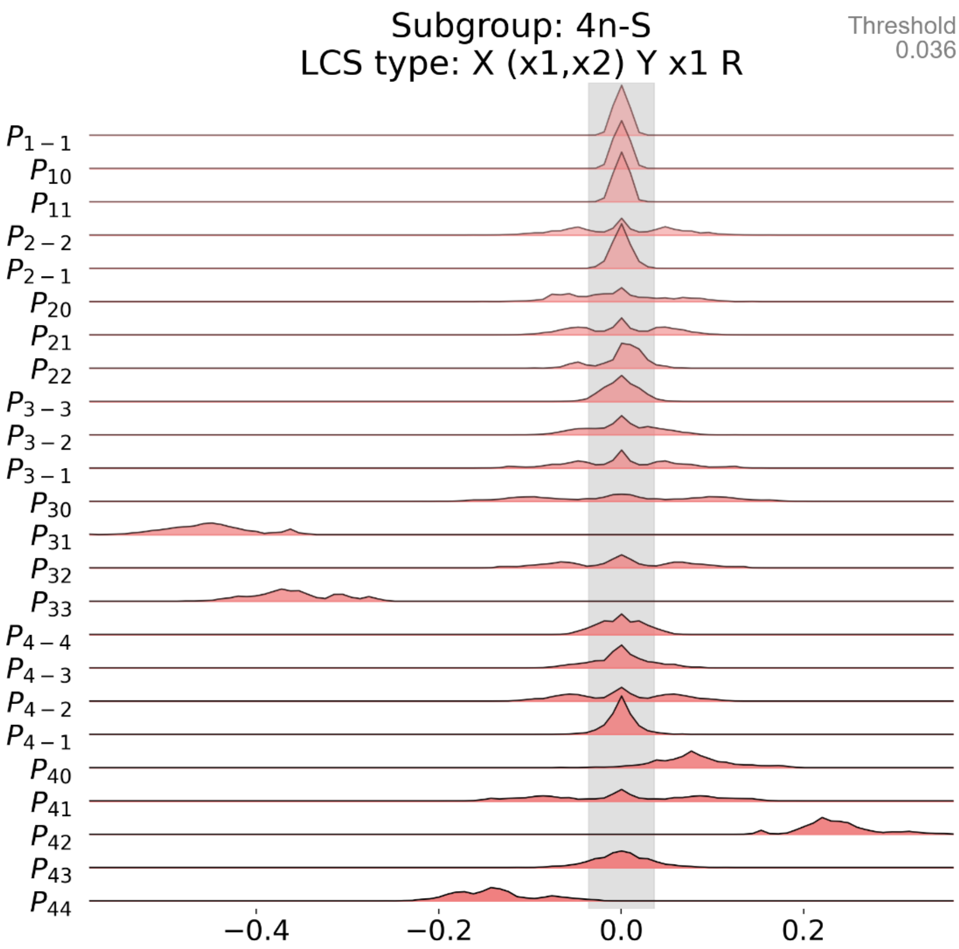

	min	max	median	mean	ssd
κ	0.9569	0.9732	0.9639	0.9644	0.0031
κ'	1.0024	1.1729	1.0428	1.0467	0.0279
P_{val}	5.779	6.108	5.968	5.965	0.058
P_{1-1}	-0.026	0.026	0.000	0.000	0.008
P_{10}	-0.026	0.026	0.000	0.000	0.008
P_{11}	-0.026	0.026	0.000	0.000	0.008
P_{2-2}	-0.166	0.166	0.000	0.000	0.054
P_{2-1}	-0.042	0.043	-0.000	0.000	0.010
P_{20}	-0.153	0.145	-0.009	-0.008	0.052
P_{21}	-0.132	0.132	0.000	0.000	0.048
P_{22}	-0.096	0.071	0.006	0.001	0.026
P_{3-3}	-0.065	0.065	0.000	0.000	0.018
P_{3-2}	-0.111	0.122	0.000	0.000	0.036
P_{3-1}	-0.152	0.152	0.000	0.000	0.055
P_{30}	-0.221	0.221	0.000	0.000	0.089
P_{31}	-0.587	-0.343	-0.453	-0.451	0.045
P_{32}	-0.152	0.152	0.000	0.000	0.063
P_{33}	-0.479	-0.253	-0.362	-0.358	0.045
P_{4-4}	-0.067	0.067	0.000	0.000	0.024
P_{4-3}	-0.094	0.096	-0.000	0.000	0.031
P_{4-2}	-0.129	0.129	0.000	0.000	0.054
P_{4-1}	-0.090	0.082	0.000	-0.000	0.017
P_{40}	-0.067	0.195	0.078	0.079	0.041
P_{41}	-0.192	0.192	0.000	0.000	0.082
P_{42}	0.138	0.368	0.231	0.237	0.039
P_{43}	-0.118	0.118	0.000	0.000	0.031
P_{44}	-0.253	-0.012	-0.142	-0.137	0.044

Figure S2.18 Ridgeplots for P_{lm} parameters (top) and statistical data for κ, κ', P_{val}, and P_{lm} parameters (bottom) for non-planar sulfur atoms with four first neighbors (the 4n-S subgroup) in the X (x1,x2) Y x1 R LCS type. The grey rectangle shows parts of P_{lm} approximated as zero with threshold 0.036.

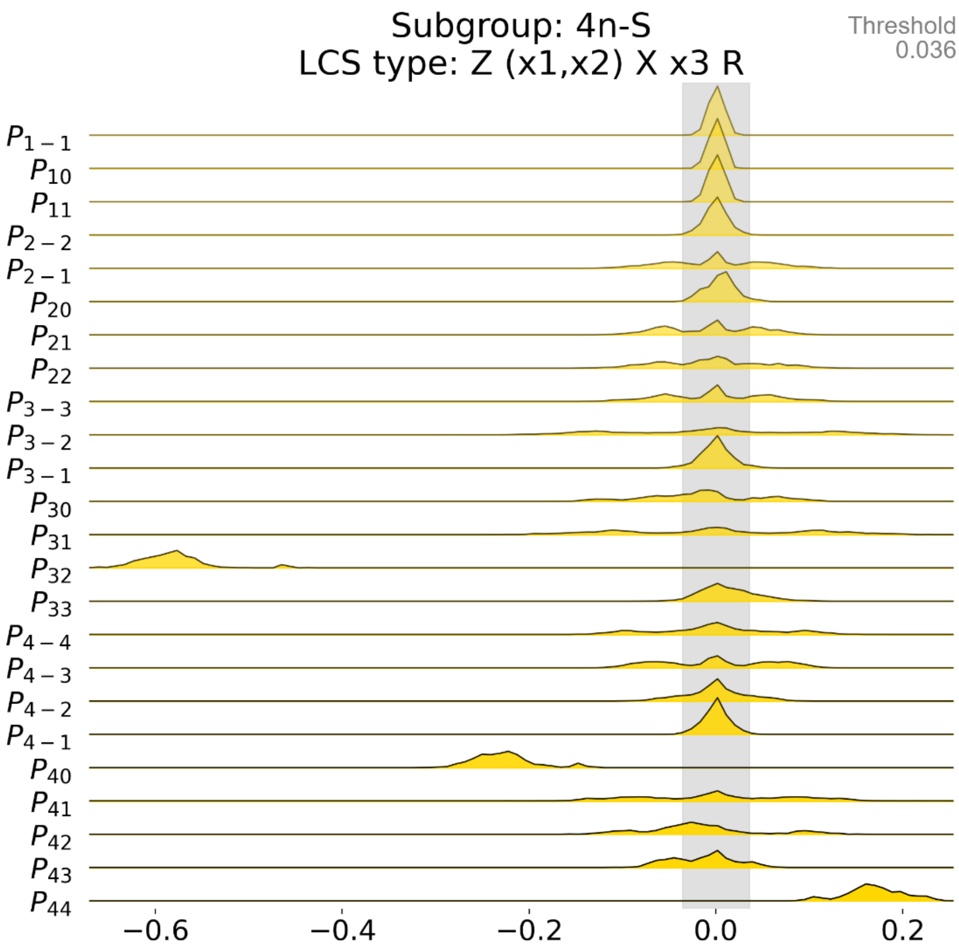

	min	max	median	mean	ssd
κ	0.9569	0.9732	0.9639	0.9644	0.0031
κ'	1.0024	1.1729	1.0428	1.0467	0.0279
P_{val}	5.779	6.108	5.968	5.965	0.058
P_{1-1}	-0.026	0.026	0.000	0.000	0.008
P_{10}	-0.026	0.026	0.000	0.000	0.008
P_{11}	-0.026	0.026	0.000	0.000	0.008
P_{2-2}	-0.048	0.044	-0.000	0.000	0.012
P_{2-1}	-0.165	0.163	0.000	-0.000	0.052
P_{20}	-0.035	0.057	0.006	0.005	0.016
P_{21}	-0.132	0.132	-0.001	-0.004	0.050
P_{22}	-0.146	0.146	-0.003	-0.005	0.053
P_{3-3}	-0.140	0.139	-0.000	0.000	0.054
P_{3-2}	-0.289	0.243	0.001	-0.000	0.100
P_{3-1}	-0.068	0.070	-0.000	-0.000	0.017
P_{30}	-0.169	0.126	-0.016	-0.015	0.058
P_{31}	-0.243	0.244	-0.001	-0.000	0.100
P_{32}	-0.675	-0.441	-0.584	-0.582	0.037
P_{33}	-0.082	0.115	0.009	0.013	0.029
P_{4-4}	-0.149	0.177	-0.000	-0.000	0.062
P_{4-3}	-0.137	0.138	-0.000	0.000	0.059
P_{4-2}	-0.102	0.097	0.000	-0.000	0.032
P_{4-1}	-0.049	0.049	0.000	0.000	0.014
P_{40}	-0.320	-0.125	-0.230	-0.227	0.032
P_{41}	-0.220	0.220	0.000	0.000	0.079
P_{42}	-0.157	0.156	-0.023	-0.017	0.059
P_{43}	-0.112	0.069	-0.010	-0.014	0.034
P_{44}	0.083	0.258	0.168	0.170	0.031

Figure S2.19 Ridgeplots for P_{lm} parameters (top) and statistical data for κ, κ', P_{val}, and P_{lm} parameters (bottom) for non-planar sulfur atoms with four first neighbors (the 4n-S subgroup) in the Z (x1,x2) X x3 R LCS type. The grey rectangle shows parts of P_{lm} approximated as zero with threshold 0.036.

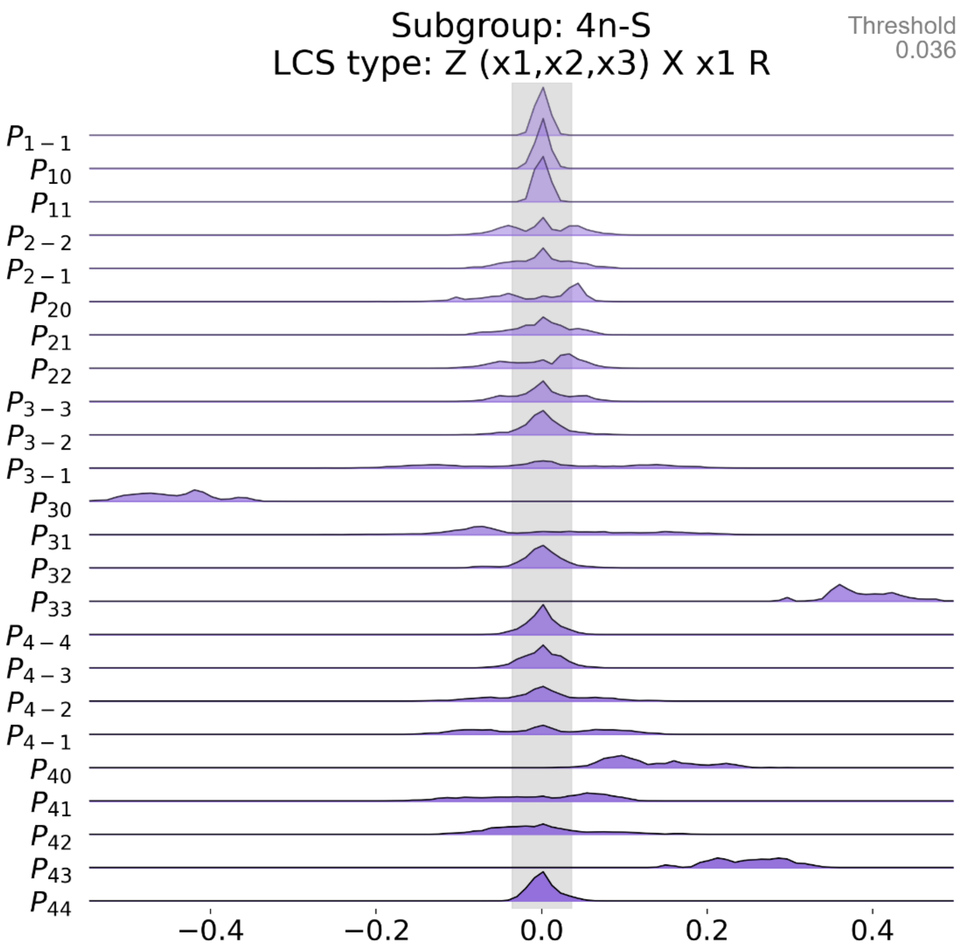

	min	max	median	mean	ssd
κ	0.9569	0.9732	0.9639	0.9644	0.0031
κ'	1.0024	1.1729	1.0428	1.0467	0.0279
P_{val}	5.779	6.108	5.968	5.965	0.058
P_{1-1}	-0.026	0.026	0.000	0.000	0.008
P_{10}	-0.023	0.025	0.001	0.000	0.008
P_{11}	-0.025	0.023	-0.000	0.000	0.008
P_{2-2}	-0.107	0.106	0.000	-0.000	0.040
P_{2-1}	-0.129	0.132	0.000	0.000	0.036
P_{20}	-0.199	0.082	0.004	-0.008	0.053
P_{21}	-0.148	0.095	0.001	-0.001	0.035
P_{22}	-0.128	0.106	0.011	0.002	0.042
P_{3-3}	-0.108	0.129	-0.000	0.000	0.035
P_{3-2}	-0.114	0.109	-0.000	0.000	0.025
P_{3-1}	-0.257	0.242	0.000	0.000	0.106
P_{30}	-0.551	-0.316	-0.444	-0.444	0.046
P_{31}	-0.263	0.252	-0.016	0.003	0.103
P_{32}	-0.098	0.109	0.000	0.000	0.026
P_{33}	0.282	0.502	0.380	0.387	0.040
P_{4-4}	-0.072	0.065	0.000	-0.000	0.019
P_{4-3}	-0.077	0.095	-0.000	-0.000	0.023
P_{4-2}	-0.153	0.155	0.000	0.000	0.052
P_{4-1}	-0.168	0.190	0.000	-0.000	0.073
P_{40}	0.014	0.299	0.113	0.129	0.053
P_{41}	-0.191	0.141	0.007	-0.002	0.071
P_{42}	-0.129	0.189	-0.009	-0.003	0.056
P_{43}	0.139	0.352	0.254	0.251	0.044
P_{44}	-0.056	0.073	-0.000	0.001	0.017

Figure S2.20 Ridgeplots for P_{lm} parameters (top) and statistical data for κ, κ', P_{val}, and P_{lm} parameters (bottom) for non-planar sulfur atoms with four first neighbors (the 4n-S subgroup) in the Z (x1,x2,x3) X x1 R LCS type. The grey rectangle shows parts of P_{lm} approximated as zero with threshold 0.036.

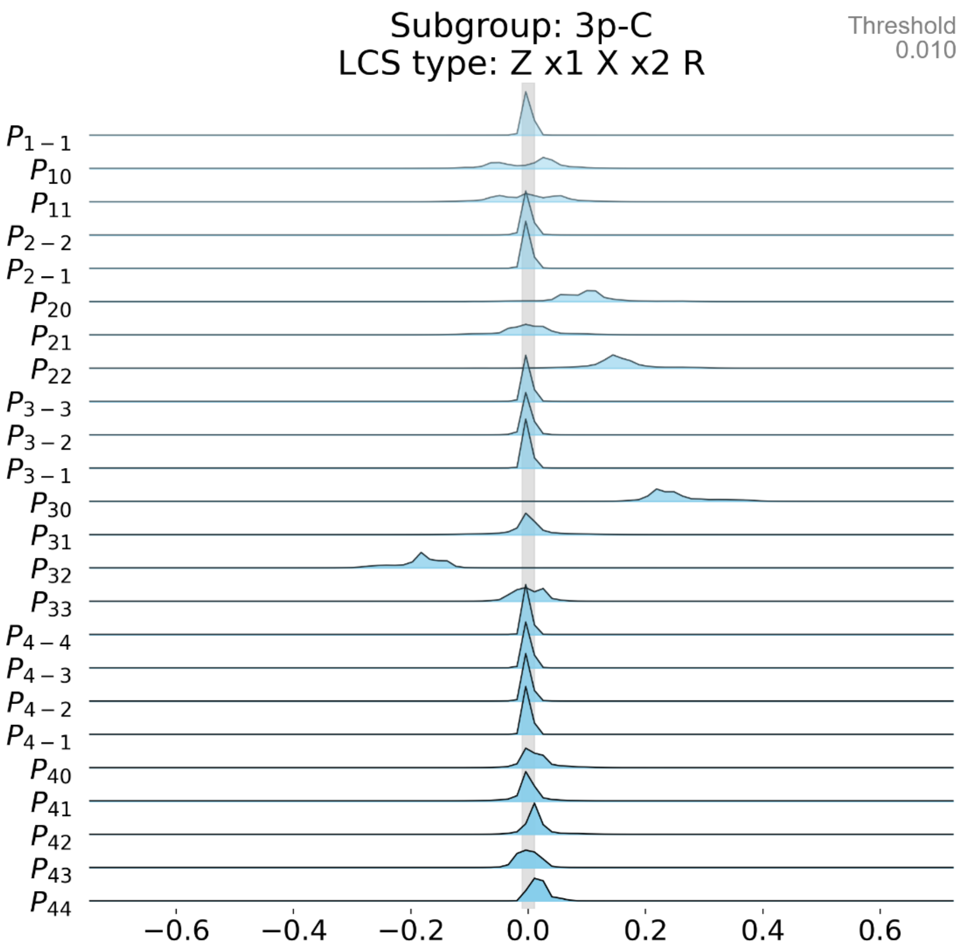

	min	max	median	mean	ssd
κ	0.9750	1.0572	1.0021	1.0019	0.0054
κ'	0.5841	1.1128	0.9000	0.8941	0.0304
P_{val}	3.272	4.376	3.944	3.967	0.098
P_{1-1}	-0.128	0.128	0.000	0.000	0.006
P_{10}	-0.754	0.535	0.013	-0.000	0.055
P_{11}	-0.731	0.731	0.000	0.000	0.054
P_{2-2}	-0.143	0.139	0.000	-0.000	0.008
P_{2-1}	-0.117	0.119	0.000	-0.000	0.006
P_{20}	-0.204	0.559	0.098	0.095	0.053
P_{21}	-0.467	0.467	-0.000	-0.000	0.049
P_{22}	-0.141	0.429	0.150	0.153	0.050
P_{3-3}	-0.103	0.103	0.000	-0.000	0.006
P_{3-2}	-0.138	0.150	-0.000	0.000	0.009
P_{3-1}	-0.124	0.122	0.000	0.000	0.005
P_{30}	-0.218	0.544	0.240	0.252	0.051
P_{31}	-0.352	0.352	-0.000	-0.000	0.040
P_{32}	-0.401	0.199	-0.180	-0.185	0.041
P_{33}	-0.285	0.285	0.000	0.000	0.026
P_{4-4}	-0.247	0.123	0.000	-0.000	0.004
P_{4-3}	-0.215	0.215	0.000	0.000	0.006
P_{4-2}	-0.072	0.117	-0.000	0.000	0.006
P_{4-1}	-0.202	0.202	0.000	0.000	0.005
P_{40}	-0.226	0.331	0.007	0.012	0.026
P_{41}	-0.391	0.391	0.000	0.000	0.023
P_{42}	-0.295	0.302	0.010	0.013	0.021
P_{43}	-0.190	0.189	-0.000	-0.000	0.018
P_{44}	-0.092	0.202	0.017	0.018	0.015

Figure S2.21 Ridgeplots for P_{lm} parameters (top) and statistical data for κ, κ', P_{val}, and P_{lm} parameters (bottom) for planar carbon atoms with three first neighbors (the 3p-C subgroup) in the Z x1 X x2 R LCS type. The grey rectangle shows parts of P_{lm} approximated as zero with threshold 0.010.

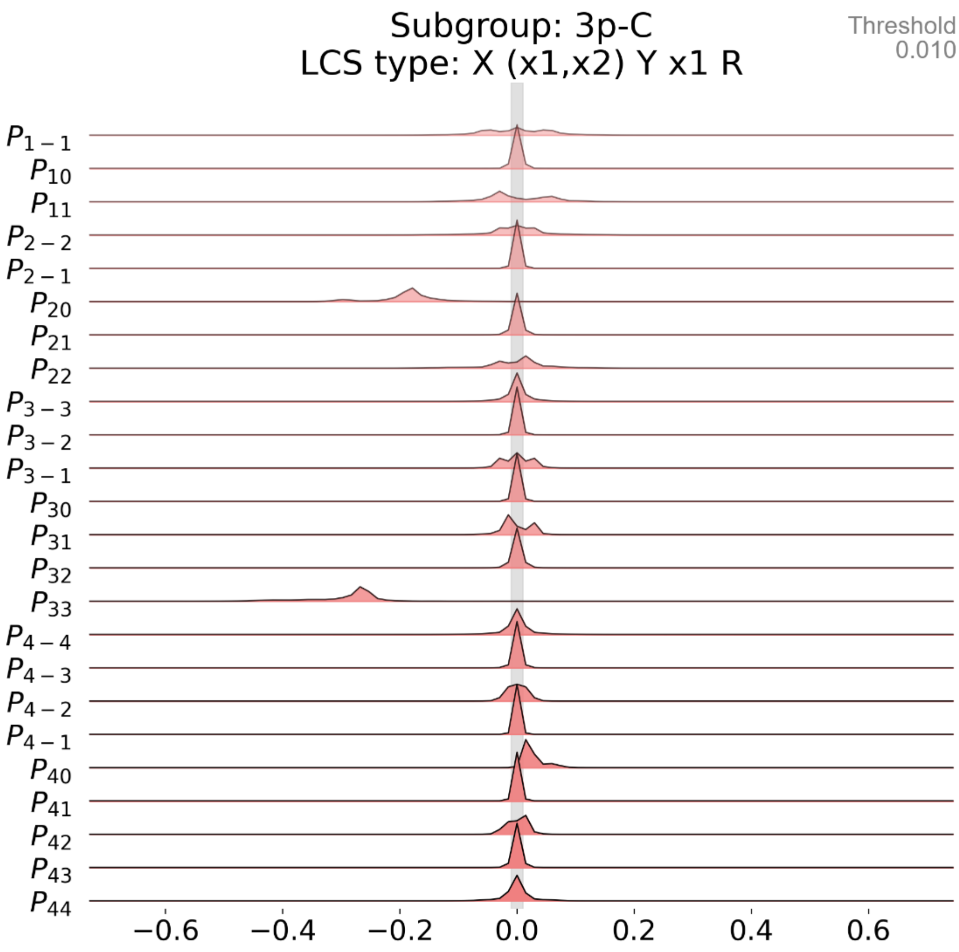

	min	max	median	mean	ssd
κ	0.9750	1.0572	1.0021	1.0019	0.0054
κ'	0.5841	1.1128	0.9000	0.8941	0.0304
P_{val}	3.272	4.376	3.944	3.967	0.098
P_{1-1}	-0.737	0.737	0.000	0.000	0.055
P_{10}	-0.128	0.128	0.000	0.000	0.006
P_{11}	-0.513	0.752	-0.014	-0.000	0.055
P_{2-2}	-0.435	0.435	0.000	0.000	0.049
P_{2-1}	-0.109	0.121	0.000	0.000	0.005
P_{20}	-0.433	0.114	-0.182	-0.186	0.053
P_{21}	-0.162	0.162	0.000	0.000	0.009
P_{22}	-0.320	0.504	0.006	-0.001	0.050
P_{3-3}	-0.200	0.200	0.000	0.000	0.022
P_{3-2}	-0.139	0.150	-0.000	-0.000	0.005
P_{3-1}	-0.285	0.285	0.000	0.000	0.024
P_{30}	-0.083	0.083	0.000	0.000	0.005
P_{31}	-0.281	0.273	-0.007	-0.000	0.024
P_{32}	-0.164	0.164	0.000	0.000	0.009
P_{33}	-0.595	0.044	-0.272	-0.293	0.057
P_{4-4}	-0.362	0.362	0.000	0.000	0.025
P_{4-3}	-0.085	0.069	0.000	-0.000	0.006
P_{4-2}	-0.162	0.162	0.000	0.000	0.016
P_{4-1}	-0.264	0.141	0.000	0.000	0.004
P_{40}	-0.086	0.264	0.021	0.027	0.017
P_{41}	-0.235	0.235	0.000	0.000	0.004
P_{42}	-0.190	0.154	0.004	0.000	0.016
P_{43}	-0.080	0.080	0.000	0.000	0.006
P_{44}	-0.310	0.413	-0.001	-0.001	0.024

Figure S2.22 Ridgeplots for P_{lm} parameters (top) and statistical data for κ, κ', P_{val}, and P_{lm} parameters (bottom) for planar carbon atoms with three first neighbors (the 3p-C subgroup) in the X (x1,x2) Y x1 R LCS type. The grey rectangle shows parts of P_{lm} approximated as zero with threshold 0.010.

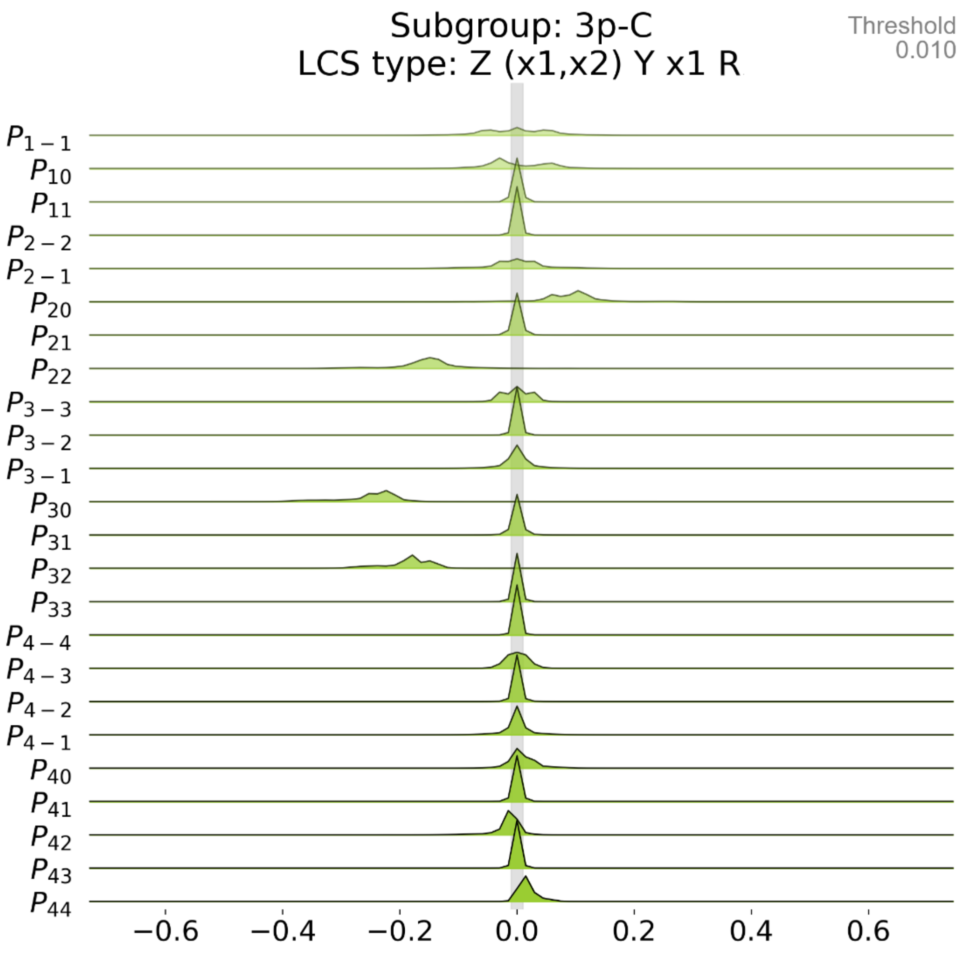

	min	max	median	mean	ssd
κ	0.9750	1.0572	1.0021	1.0019	0.0054
κ'	0.5841	1.1128	0.9000	0.8941	0.0304
P_{val}	3.272	4.376	3.944	3.967	0.098
P_{1-1}	-0.737	0.737	0.000	0.000	0.055
P_{10}	-0.513	0.752	-0.014	-0.000	0.055
P_{11}	-0.128	0.128	0.000	0.000	0.006
P_{2-2}	-0.121	0.109	-0.000	-0.000	0.005
P_{2-1}	-0.435	0.435	0.000	0.000	0.049
P_{20}	-0.204	0.559	0.096	0.092	0.052
P_{21}	-0.162	0.162	0.000	0.000	0.009
P_{22}	-0.430	0.142	-0.150	-0.155	0.051
P_{3-3}	-0.282	0.282	0.000	0.000	0.023
P_{3-2}	-0.150	0.139	0.000	0.000	0.005
P_{3-1}	-0.201	0.201	0.000	0.000	0.024
P_{30}	-0.534	0.221	-0.241	-0.253	0.052
P_{31}	-0.123	0.123	0.000	0.000	0.009
P_{32}	-0.399	0.200	-0.181	-0.187	0.041
P_{33}	-0.139	0.139	0.000	0.000	0.005
P_{4-4}	-0.111	0.253	0.000	-0.000	0.004
P_{4-3}	-0.180	0.180	0.000	0.000	0.018
P_{4-2}	-0.070	0.091	-0.000	0.000	0.006
P_{4-1}	-0.380	0.380	0.000	0.000	0.025
P_{40}	-0.232	0.335	0.005	0.009	0.023
P_{41}	-0.223	0.223	0.000	0.000	0.006
P_{42}	-0.305	0.296	-0.011	-0.015	0.021
P_{43}	-0.214	0.214	0.000	0.000	0.005
P_{44}	-0.102	0.202	0.017	0.018	0.015

Figure S2.23 Ridgeplots for P_{lm} parameters (top) and statistical data for κ, κ', P_{val}, and P_{lm} parameters (bottom) for planar carbon atoms with three first neighbors (the 3p-C subgroup) in the Z (x1,x2) Y x1 R LCS type. The grey rectangle shows parts of P_{lm} approximated as zero with threshold 0.010.

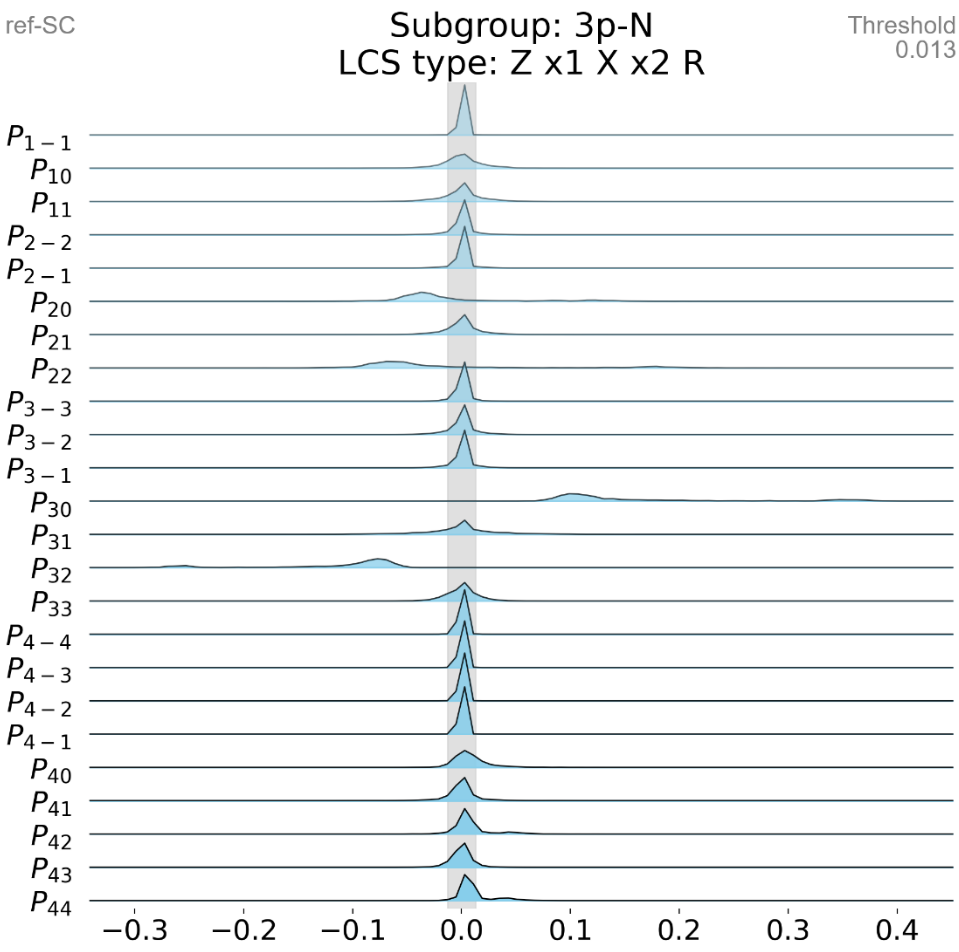

	min	max	median	mean	ssd
κ	0.9802	1.0193	0.9952	0.9950	0.0057
κ'	0.7100	1.2176	1.0145	0.9724	0.1190
P_{val}	4.667	5.320	5.012	5.008	0.080
P_{1-1}	-0.024	0.024	0.000	0.000	0.002
P_{10}	-0.113	0.126	0.000	0.000	0.019
P_{11}	-0.117	0.117	0.000	-0.000	0.019
P_{2-2}	-0.054	0.060	0.000	-0.000	0.009
P_{2-1}	-0.049	0.052	0.000	-0.000	0.007
P_{20}	-0.169	0.222	-0.030	-0.012	0.056
P_{21}	-0.178	0.178	0.000	0.000	0.019
P_{22}	-0.183	0.293	-0.053	-0.020	0.086
P_{3-3}	-0.041	0.050	0.000	-0.000	0.005
P_{3-2}	-0.067	0.073	0.000	0.000	0.011
P_{3-1}	-0.053	0.049	0.000	0.000	0.008
P_{30}	0.005	0.455	0.118	0.157	0.088
P_{31}	-0.254	0.254	0.000	-0.000	0.033
P_{32}	-0.345	0.004	-0.087	-0.115	0.065
P_{33}	-0.077	0.078	0.000	0.000	0.014
P_{4-4}	-0.023	0.026	0.000	0.000	0.002
P_{4-3}	-0.017	0.018	0.000	0.000	0.002
P_{4-2}	-0.017	0.015	0.000	-0.000	0.001
P_{4-1}	-0.018	0.015	0.000	0.000	0.002
P_{40}	-0.049	0.121	0.006	0.008	0.015
P_{41}	-0.112	0.112	0.000	-0.000	0.015
P_{42}	-0.066	0.121	0.005	0.008	0.016
P_{43}	-0.059	0.059	0.000	-0.000	0.009
P_{44}	-0.025	0.103	0.007	0.012	0.015

Figure S2.24 Ridgeplots for P_{lm} parameters (top) and statistical data for κ, κ', P_{val}, and P_{lm} parameters (bottom) for planar nitrogen atoms with three first neighbors (the 3p-N subgroup) for the refinement with symmetry constraints (ref-SC) in the Z x1 X x2 R LCS type. The grey rectangle shows parts of P_{lm} approximated as zero with threshold 0.013.

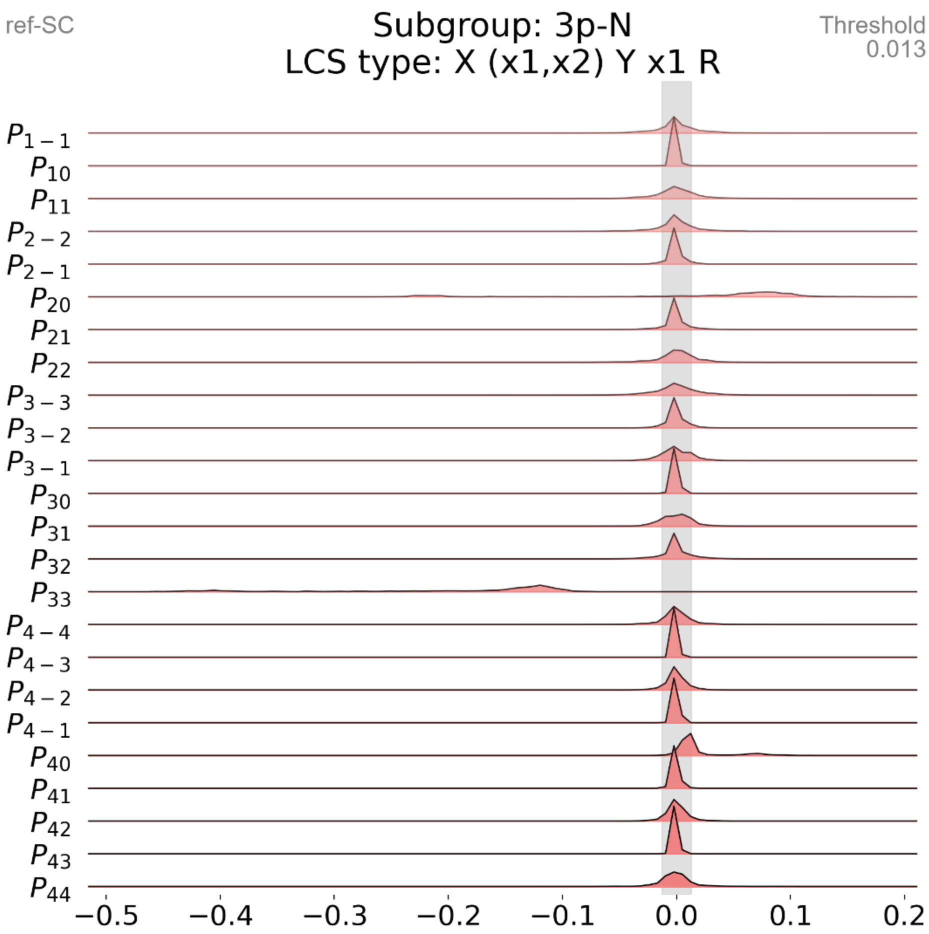

	min	max	median	mean	ssd
κ	0.9802	1.0193	0.9952	0.9950	0.0057
κ'	0.7100	1.2176	1.0145	0.9724	0.1190
P_{val}	4.667	5.320	5.012	5.008	0.080
P_{1-1}	-0.126	0.126	0.000	0.000	0.019
P_{10}	-0.024	0.024	0.000	0.000	0.002
P_{11}	-0.132	0.113	0.000	0.000	0.019
P_{2-2}	-0.214	0.214	0.000	0.000	0.020
P_{2-1}	-0.038	0.028	0.000	-0.000	0.006
P_{20}	-0.261	0.199	0.065	0.024	0.104
P_{21}	-0.057	0.057	0.000	0.000	0.010
P_{22}	-0.221	0.099	0.001	-0.000	0.019
P_{3-3}	-0.156	0.156	0.000	0.000	0.019
P_{3-2}	-0.050	0.043	0.000	-0.000	0.007
P_{3-1}	-0.073	0.073	0.000	0.000	0.013
P_{30}	-0.040	0.040	0.000	0.000	0.002
P_{31}	-0.066	0.058	0.000	-0.000	0.013
P_{32}	-0.068	0.068	0.000	0.000	0.013
P_{33}	-0.519	-0.053	-0.137	-0.183	0.101
P_{4-4}	-0.125	0.125	0.000	0.000	0.014
P_{4-3}	-0.013	0.014	0.000	0.000	0.001
P_{4-2}	-0.046	0.046	0.000	0.000	0.009
P_{4-1}	-0.014	0.013	0.000	0.000	0.002
P_{40}	-0.014	0.116	0.011	0.018	0.022
P_{41}	-0.024	0.024	0.000	0.000	0.003
P_{42}	-0.043	0.044	0.000	0.000	0.009
P_{43}	-0.018	0.018	0.000	0.000	0.002
P_{44}	-0.127	0.104	-0.001	-0.001	0.014

Figure S2.25 Ridgeplots for P_{lm} parameters (top) and statistical data for κ, κ', P_{val}, and P_{lm} parameters (bottom) for planar nitrogen atoms with three first neighbors (the 3p-N subgroup) for the refinement with symmetry constraints (ref-SC) in the X (x1,x2) Y x1 R LCS type. The grey rectangle shows parts of P_{lm} approximated as zero with threshold 0.013.

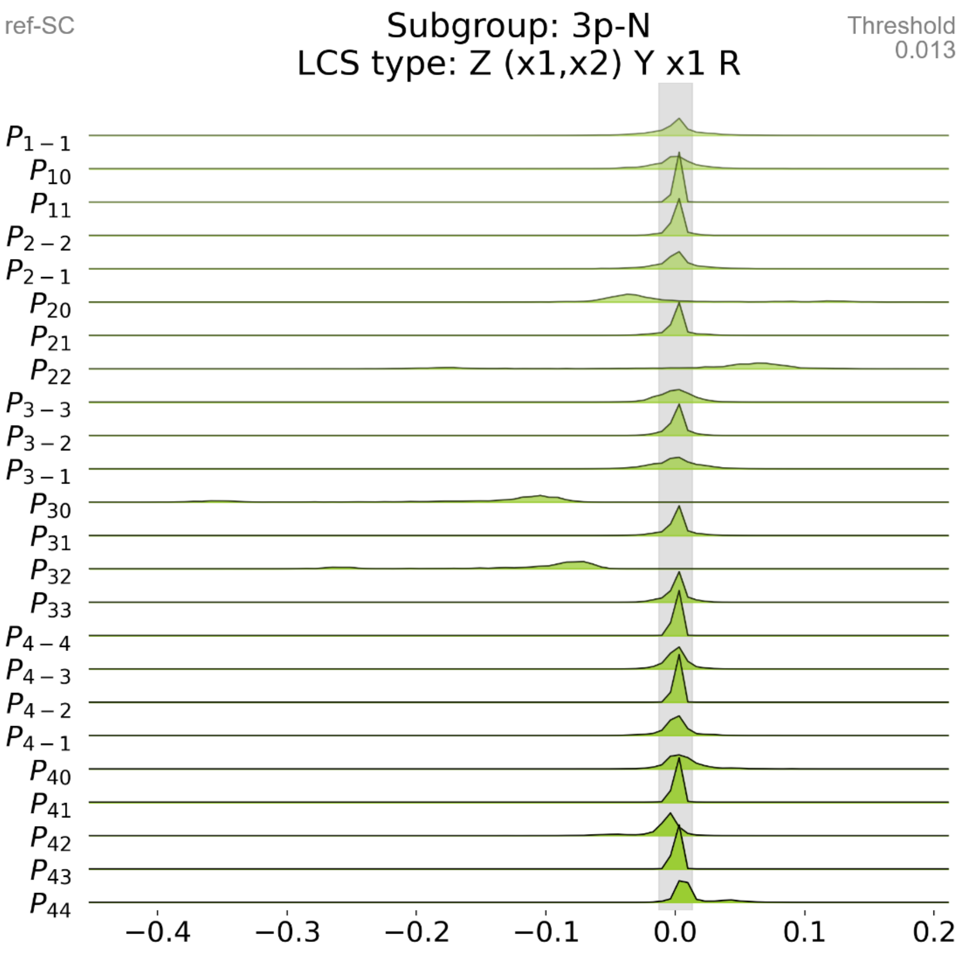

	min	max	median	mean	ssd
κ	0.9802	1.0193	0.9952	0.9950	0.0057
κ'	0.7100	1.2176	1.0145	0.9724	0.1190
P_{val}	4.667	5.320	5.012	5.008	0.080
P_{1-1}	-0.126	0.126	0.000	0.000	0.019
P_{10}	-0.132	0.113	0.000	0.000	0.019
P_{11}	-0.024	0.024	0.000	0.000	0.002
P_{2-2}	-0.028	0.038	0.000	0.000	0.006
P_{2-1}	-0.214	0.214	0.000	0.000	0.020
P_{20}	-0.167	0.192	-0.031	-0.012	0.054
P_{21}	-0.057	0.057	0.000	0.000	0.010
P_{22}	-0.292	0.183	0.053	0.020	0.087
P_{3-3}	-0.080	0.080	0.000	0.000	0.014
P_{3-2}	-0.043	0.050	0.000	0.000	0.007
P_{3-1}	-0.156	0.156	0.000	0.000	0.018
P_{30}	-0.456	-0.047	-0.119	-0.159	0.088
P_{31}	-0.057	0.057	0.000	0.000	0.010
P_{32}	-0.344	-0.032	-0.088	-0.117	0.065
P_{33}	-0.037	0.037	0.000	0.000	0.008
P_{4-4}	-0.013	0.010	0.000	-0.000	0.002
P_{4-3}	-0.051	0.051	0.000	0.000	0.009
P_{4-2}	-0.015	0.015	0.000	-0.000	0.001
P_{4-1}	-0.130	0.130	0.000	0.000	0.014
P_{40}	-0.068	0.103	0.004	0.006	0.015
P_{41}	-0.022	0.022	0.000	0.000	0.003
P_{42}	-0.132	0.054	-0.006	-0.010	0.016
P_{43}	-0.016	0.016	0.000	0.000	0.002
P_{44}	-0.025	0.103	0.007	0.012	0.015

Figure S2.26 Ridgeplots for P_{lm} parameters (top) and statistical data for κ, κ', P_{val}, and P_{lm} parameters (bottom) for planar nitrogen atoms with three first neighbors (the 3p-N subgroup) for the refinement with symmetry constraints (ref-SC) in the Z (x1,x2) Y x1 R LCS type. The grey rectangle shows parts of P_{lm} approximated as zero with threshold 0.013.

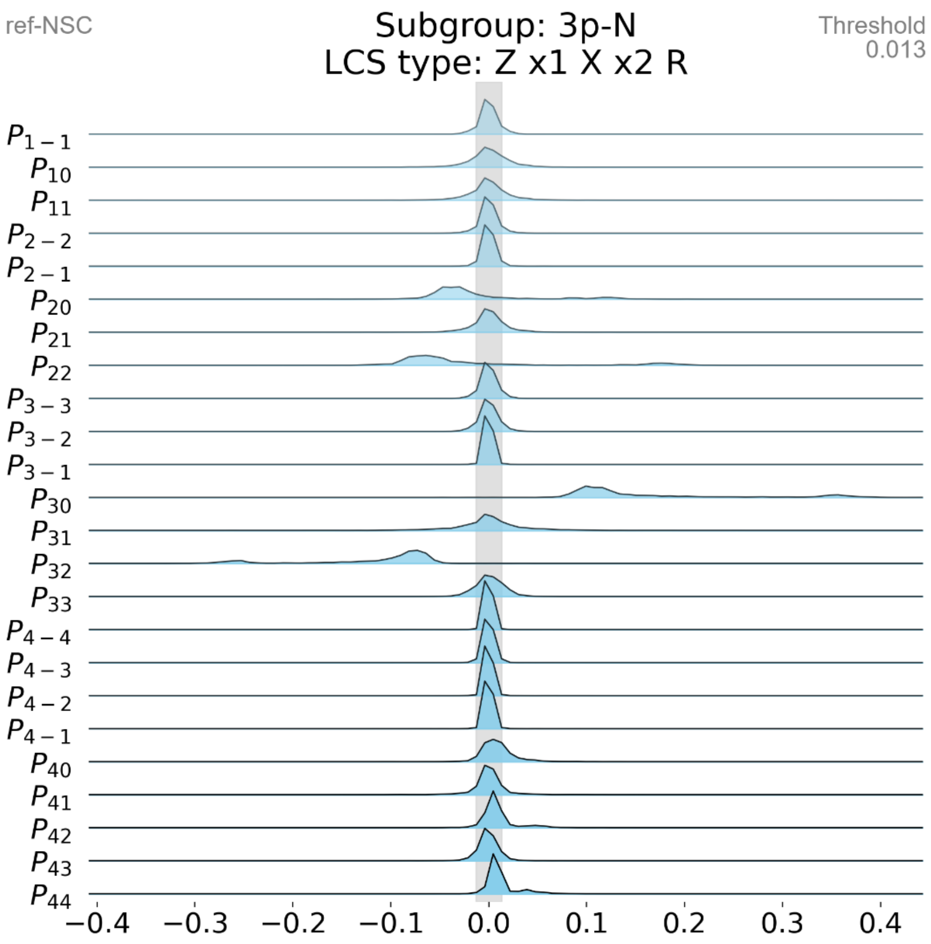

	min	max	median	mean	ssd
κ	0.9781	1.0217	0.9954	0.9950	0.0059
κ'	0.7088	1.8155	1.0152	0.9737	0.1224
P_{val}	4.663	5.372	5.005	5.002	0.081
P_{1-1}	-0.048	0.048	0.000	0.000	0.009
P_{10}	-0.268	0.436	0.000	0.000	0.021
P_{11}	-0.412	0.410	0.000	-0.000	0.021
P_{2-2}	-0.086	0.117	0.000	0.000	0.009
P_{2-1}	-0.085	0.059	0.000	-0.000	0.006
P_{20}	-0.241	0.241	-0.031	-0.012	0.056
P_{21}	-0.179	0.179	0.000	0.000	0.020
P_{22}	-0.236	0.292	-0.055	-0.022	0.086
P_{3-3}	-0.047	0.045	0.000	0.000	0.008
P_{3-2}	-0.049	0.052	0.000	-0.000	0.009
P_{3-1}	-0.054	0.052	0.000	-0.000	0.004
P_{30}	-0.048	0.447	0.119	0.158	0.088
P_{31}	-0.258	0.258	0.000	-0.000	0.032
P_{32}	-0.339	0.039	-0.087	-0.116	0.066
P_{33}	-0.086	0.090	0.001	0.001	0.015
P_{4-4}	-0.147	0.080	0.000	-0.000	0.004
P_{4-3}	-0.091	0.087	0.000	0.000	0.005
P_{4-2}	-0.046	0.097	0.000	0.000	0.004
P_{4-1}	-0.114	0.111	0.000	0.000	0.004
P_{40}	-0.109	0.130	0.006	0.008	0.016
P_{41}	-0.149	0.152	0.000	0.000	0.015
P_{42}	-0.110	0.140	0.006	0.009	0.016
P_{43}	-0.084	0.079	-0.001	-0.001	0.010
P_{44}	-0.079	0.161	0.008	0.012	0.015

Figure S2.27 Ridgeplots for P_{lm} parameters (top) and statistical data for κ, κ', P_{val}, and P_{lm} parameters (bottom) for planar nitrogen atoms with three first neighbors (the 3p-N subgroup) for the refinement without symmetry constraints (ref-NSC) in the Z x1 X x2 R LCS type. The grey rectangle shows parts of P_{lm} approximated as zero with threshold 0.013.

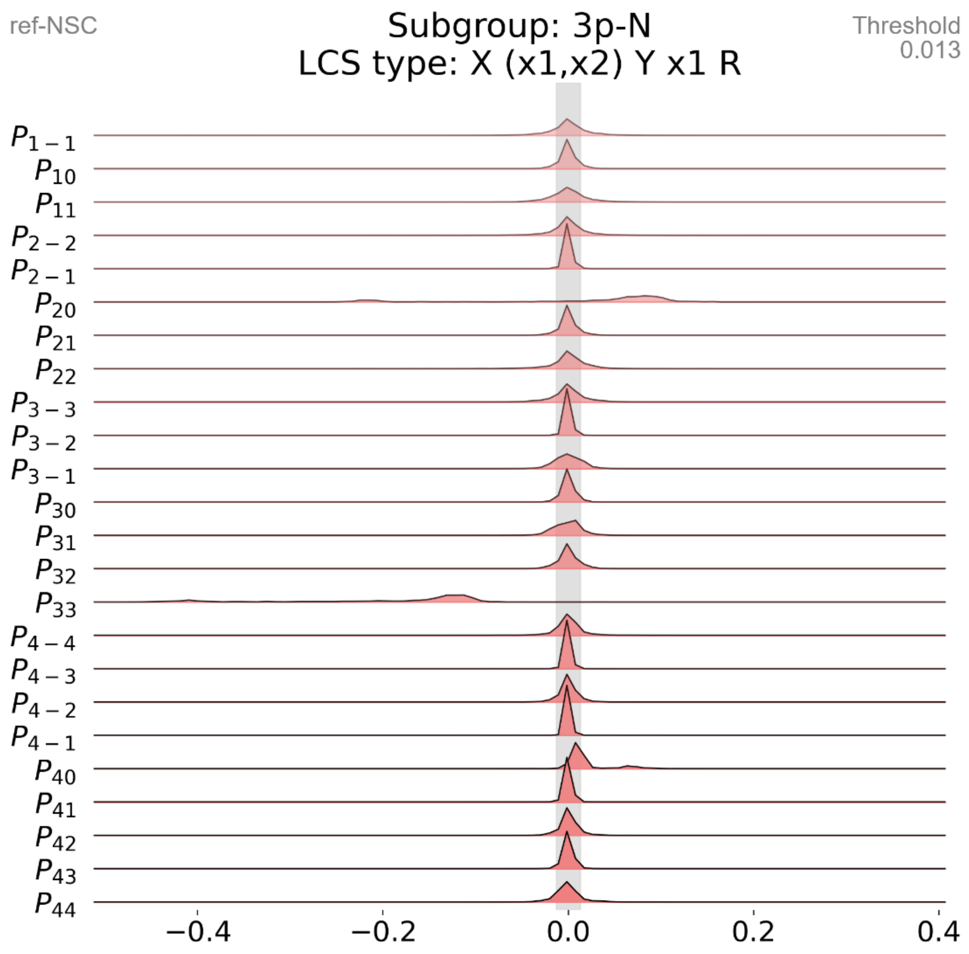

	min	max	median	mean	ssd
κ	0.9781	1.0217	0.9954	0.9950	0.0059
κ'	0.7088	1.8155	1.0152	0.9737	0.1224
P_{val}	4.663	5.372	5.005	5.002	0.081
P_{1-1}	-0.411	0.411	0.000	0.000	0.021
P_{10}	-0.048	0.048	0.000	0.000	0.009
P_{11}	-0.438	0.275	-0.001	-0.000	0.021
P_{2-2}	-0.214	0.214	0.000	0.000	0.021
P_{2-1}	-0.067	0.047	0.000	0.000	0.004
P_{20}	-0.266	0.200	0.066	0.026	0.104
P_{21}	-0.117	0.117	0.000	0.000	0.010
P_{22}	-0.222	0.146	0.001	-0.000	0.020
P_{3-3}	-0.154	0.154	0.000	0.000	0.017
P_{3-2}	-0.048	0.049	0.000	0.000	0.004
P_{3-1}	-0.087	0.087	0.000	0.000	0.014
P_{30}	-0.032	0.032	0.000	0.000	0.007
P_{31}	-0.093	0.061	0.000	-0.001	0.014
P_{32}	-0.048	0.048	0.000	0.000	0.011
P_{33}	-0.516	0.039	-0.136	-0.184	0.102
P_{4-4}	-0.157	0.157	0.000	0.000	0.014
P_{4-3}	-0.084	0.044	0.000	-0.000	0.004
P_{4-2}	-0.066	0.066	0.000	0.000	0.009
P_{4-1}	-0.113	0.088	0.000	0.000	0.004
P_{40}	-0.064	0.135	0.011	0.018	0.021
P_{41}	-0.106	0.106	0.000	0.000	0.004
P_{42}	-0.092	0.085	0.001	0.001	0.009
P_{43}	-0.057	0.057	0.000	0.000	0.006
P_{44}	-0.131	0.157	-0.001	-0.001	0.015

Figure S2.28 Ridgeplots for P_{lm} parameters (top) and statistical data for κ, κ', P_{val}, and P_{lm} parameters (bottom) for planar nitrogen atoms with three first neighbors (the 3p-N subgroup) for the refinement without symmetry constraints (ref-NSC) in the X (x1,x2) Y x1 R LCS type. The grey rectangle shows parts of P_{lm} approximated as zero with threshold 0.013.

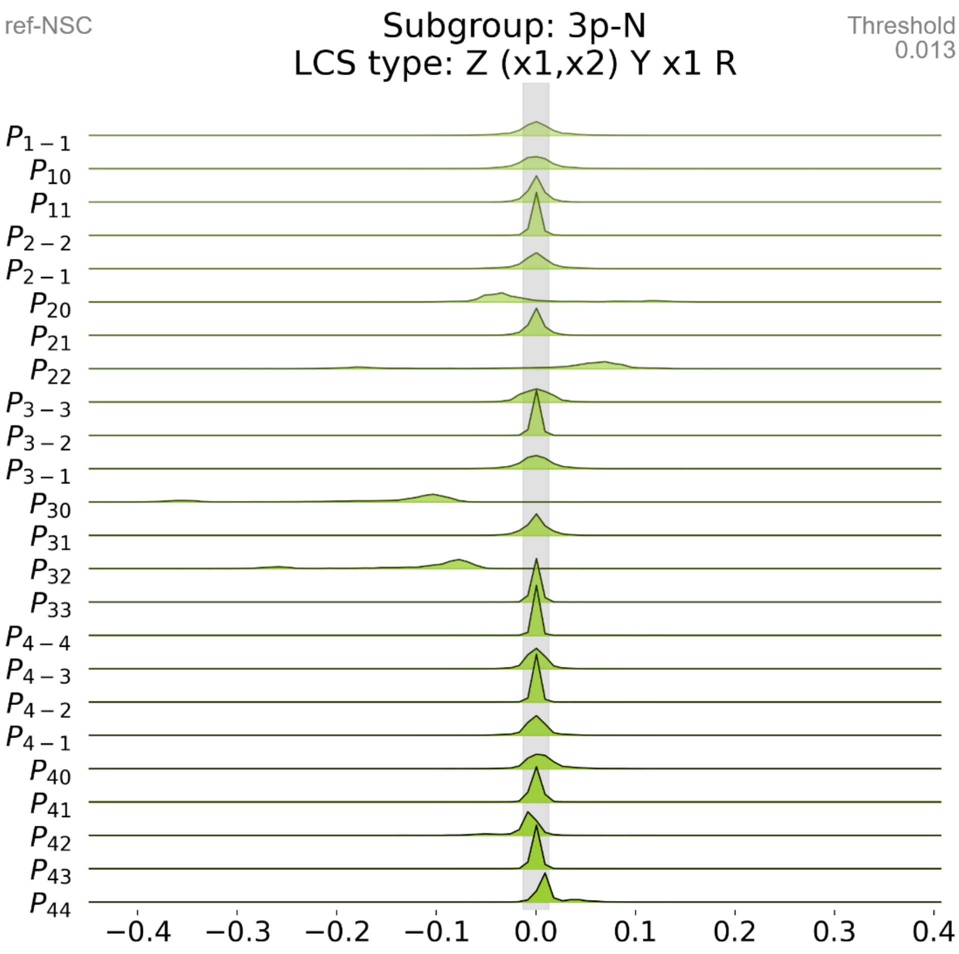

	min	max	median	mean	ssd
κ	0.9781	1.0217	0.9954	0.9950	0.0059
κ'	0.7088	1.8155	1.0152	0.9737	0.1224
P_{val}	4.663	5.372	5.005	5.002	0.081
P_{1-1}	-0.411	0.411	0.000	0.000	0.021
P_{10}	-0.438	0.275	-0.001	-0.000	0.021
P_{11}	-0.048	0.048	0.000	0.000	0.009
P_{2-2}	-0.047	0.067	0.000	-0.000	0.004
P_{2-1}	-0.214	0.214	0.000	0.000	0.021
P_{20}	-0.222	0.215	-0.031	-0.013	0.055
P_{21}	-0.117	0.117	0.000	0.000	0.010
P_{22}	-0.292	0.229	0.054	0.021	0.087
P_{3-3}	-0.089	0.089	0.000	0.000	0.015
P_{3-2}	-0.049	0.048	0.000	-0.000	0.004
P_{3-1}	-0.154	0.154	0.000	0.000	0.017
P_{30}	-0.453	0.057	-0.119	-0.159	0.089
P_{31}	-0.052	0.052	0.000	0.000	0.012
P_{32}	-0.341	0.036	-0.090	-0.118	0.065
P_{33}	-0.045	0.045	0.000	0.000	0.004
P_{4-4}	-0.076	0.119	0.000	0.000	0.003
P_{4-3}	-0.081	0.081	0.000	0.000	0.010
P_{4-2}	-0.037	0.110	0.000	0.000	0.004
P_{4-1}	-0.146	0.146	0.000	0.000	0.015
P_{40}	-0.129	0.136	0.004	0.005	0.016
P_{41}	-0.093	0.093	0.000	0.000	0.006
P_{42}	-0.132	0.116	-0.007	-0.010	0.016
P_{43}	-0.077	0.077	0.000	0.000	0.004
P_{44}	-0.066	0.130	0.008	0.012	0.015

Figure S2.29 Ridgeplots for P_{lm} parameters (top) and statistical data for κ, κ', P_{val}, and P_{lm} parameters (bottom) for planar nitrogen atoms with three first neighbors (the 3p-N subgroup) for the refinement without symmetry constraints (ref-NSC) in the Z (x1,x2) Y x1 R LCS type. The grey rectangle shows parts of P_{lm} approximated as zero with threshold 0.013.

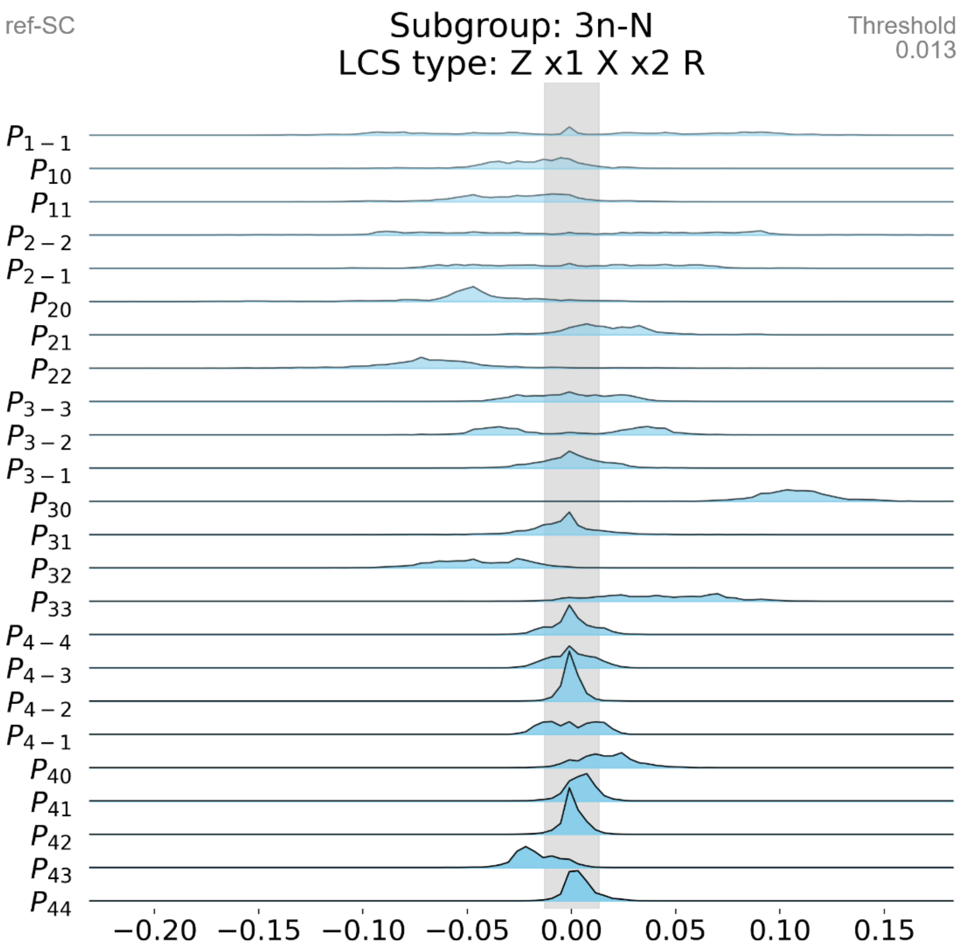

	min	max	median	mean	ssd
κ	0.9793	1.0149	0.9934	0.9931	0.0055
κ'	0.8899	1.1451	1.0307	1.0318	0.0368
P_{val}	4.764	5.265	5.031	5.033	0.078
P_{1-1}	-0.149	0.149	0.000	0.000	0.068
P_{10}	-0.113	0.074	-0.015	-0.018	0.023
P_{11}	-0.132	0.060	-0.024	-0.027	0.027
P_{2-2}	-0.176	0.174	0.000	0.000	0.067
P_{2-1}	-0.116	0.117	0.000	-0.000	0.046
P_{20}	-0.233	0.129	-0.047	-0.047	0.033
P_{21}	-0.080	0.126	0.015	0.018	0.022
P_{22}	-0.201	0.119	-0.067	-0.065	0.030
P_{3-3}	-0.090	0.064	0.000	-0.000	0.022
P_{3-2}	-0.086	0.097	0.000	-0.000	0.036
P_{3-1}	-0.062	0.061	0.000	0.000	0.015
P_{30}	-0.006	0.185	0.107	0.107	0.019
P_{31}	-0.102	0.095	-0.003	-0.004	0.017
P_{32}	-0.122	0.058	-0.045	-0.044	0.022
P_{33}	-0.054	0.116	0.040	0.041	0.028
P_{4-4}	-0.036	0.033	0.000	0.000	0.010
P_{4-3}	-0.033	0.031	0.000	0.000	0.010
P_{4-2}	-0.021	0.024	0.000	-0.000	0.005
P_{4-1}	-0.029	0.034	0.000	0.000	0.012
P_{40}	-0.025	0.066	0.016	0.016	0.013
P_{41}	-0.025	0.028	0.005	0.005	0.007
P_{42}	-0.024	0.025	0.000	0.001	0.005
P_{43}	-0.061	0.015	-0.018	-0.016	0.010
P_{44}	-0.020	0.041	0.003	0.004	0.007

Figure S2.30 Ridgeplots for P_{lm} parameters (top) and statistical data for κ, κ', P_{val}, and P_{lm} parameters (bottom) for non-planar nitrogen atoms with three first neighbors (the 3n-N subgroup) for the refinement with symmetry constraints (ref-SC) in the Z x1 X x2 R LCS type. The grey rectangle shows parts of P_{lm} approximated as zero with threshold 0.013.

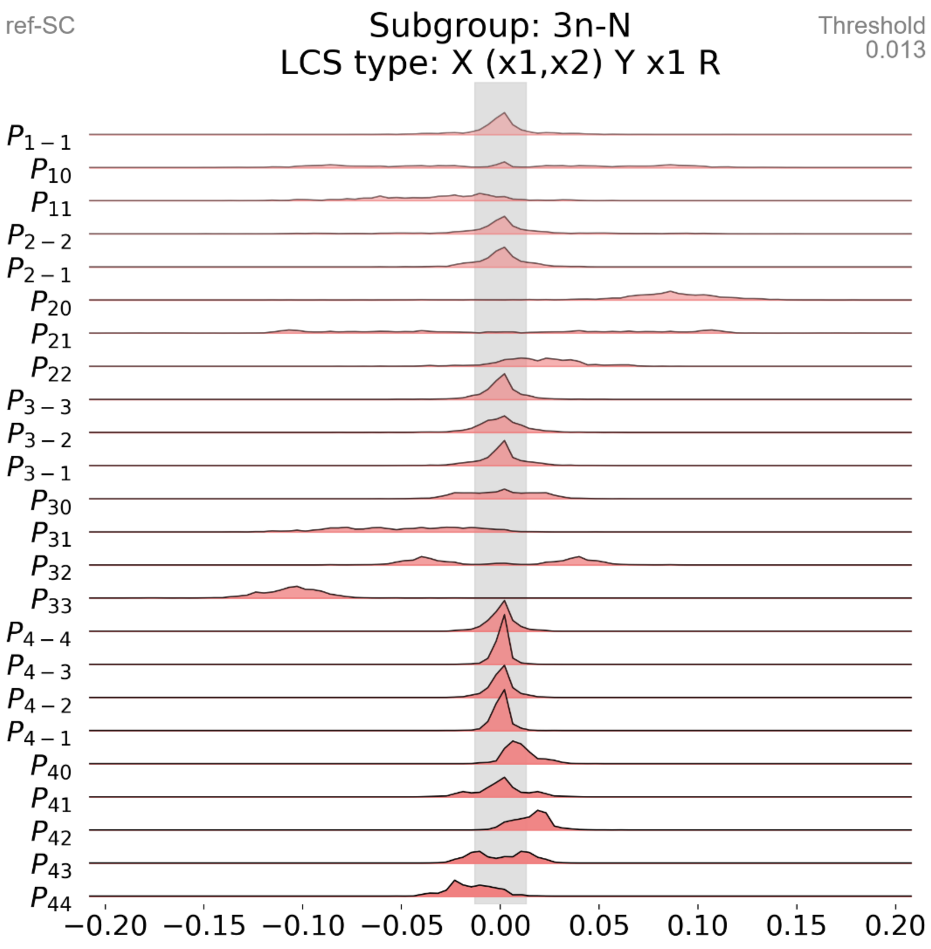

	min	max	median	mean	ssd
κ	0.9793	1.0149	0.9934	0.9931	0.0055
κ'	0.8899	1.1451	1.0307	1.0318	0.0368
P_{val}	4.764	5.265	5.031	5.033	0.078
P_{1-1}	-0.117	0.117	0.000	0.000	0.016
P_{10}	-0.149	0.149	0.000	0.000	0.068
P_{11}	-0.138	0.058	-0.028	-0.032	0.032
P_{2-2}	-0.207	0.207	0.000	0.000	0.031
P_{2-1}	-0.062	0.046	0.000	0.000	0.012
P_{20}	-0.114	0.177	0.086	0.082	0.033
P_{21}	-0.210	0.210	0.000	0.000	0.080
P_{22}	-0.083	0.092	0.018	0.019	0.021
P_{3-3}	-0.053	0.053	0.000	0.000	0.010
P_{3-2}	-0.083	0.061	0.000	0.000	0.013
P_{3-1}	-0.055	0.055	0.000	0.000	0.013
P_{30}	-0.066	0.066	0.000	0.000	0.018
P_{31}	-0.135	0.049	-0.046	-0.048	0.032
P_{32}	-0.090	0.090	0.000	0.000	0.040
P_{33}	-0.185	-0.018	-0.105	-0.106	0.017
P_{4-4}	-0.028	0.028	0.000	0.000	0.007
P_{4-3}	-0.024	0.026	0.000	0.000	0.004
P_{4-2}	-0.026	0.026	0.000	0.000	0.006
P_{4-1}	-0.028	0.028	0.000	0.000	0.004
P_{40}	-0.014	0.049	0.009	0.010	0.008
P_{41}	-0.045	0.045	0.000	0.000	0.012
P_{42}	-0.012	0.060	0.016	0.015	0.009
P_{43}	-0.037	0.037	0.000	0.000	0.014
P_{44}	-0.052	0.021	-0.015	-0.014	0.011

Figure S2.31 Ridgeplots for P_{lm} parameters (top) and statistical data for κ, κ', P_{val}, and P_{lm} parameters (bottom) for non-planar nitrogen atoms with three first neighbors (the 3n-N subgroup) for the refinement with symmetry constraints (ref-SC) in the X (x1,x2) Y x1 R LCS type. The grey rectangle shows parts of P_{lm} approximated as zero with threshold 0.013.

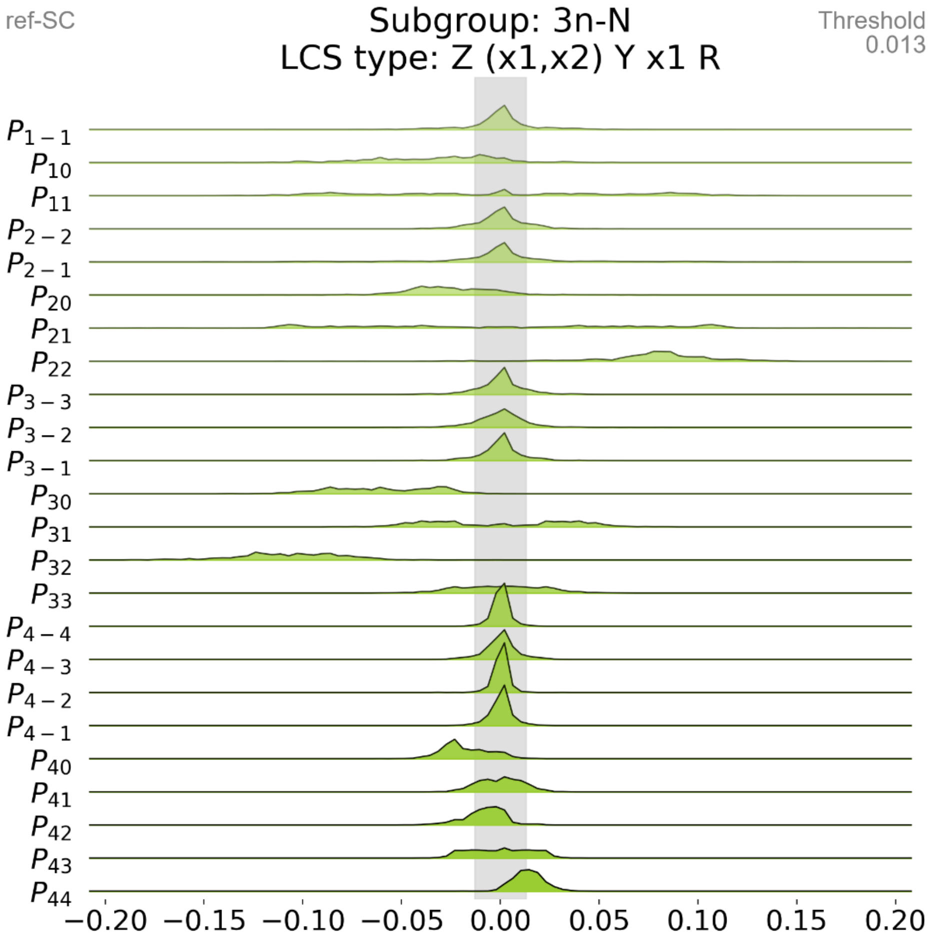

	min	max	median	mean	ssd
κ	0.9793	1.0149	0.9934	0.9931	0.0055
κ'	0.8899	1.1451	1.0307	1.0318	0.0368
P_{val}	4.764	5.265	5.031	5.033	0.078
P_{1-1}	-0.117	0.117	0.000	0.000	0.016
P_{10}	-0.138	0.058	-0.028	-0.032	0.032
P_{11}	-0.149	0.149	0.000	0.000	0.068
P_{2-2}	-0.046	0.062	0.000	-0.000	0.012
P_{2-1}	-0.207	0.207	0.000	0.000	0.031
P_{20}	-0.121	0.076	-0.027	-0.024	0.023
P_{21}	-0.210	0.210	0.000	0.000	0.080
P_{22}	-0.110	0.193	0.080	0.078	0.030
P_{3-3}	-0.047	0.047	0.000	0.000	0.011
P_{3-2}	-0.061	0.083	0.000	-0.000	0.013
P_{3-1}	-0.062	0.062	0.000	0.000	0.012
P_{30}	-0.142	0.007	-0.060	-0.060	0.027
P_{31}	-0.086	0.086	0.000	0.000	0.037
P_{32}	-0.188	-0.002	-0.105	-0.106	0.026
P_{33}	-0.067	0.067	0.000	0.000	0.022
P_{4-4}	-0.017	0.018	0.000	-0.000	0.004
P_{4-3}	-0.032	0.032	0.000	0.000	0.008
P_{4-2}	-0.033	0.033	0.000	-0.000	0.004
P_{4-1}	-0.025	0.025	0.000	0.000	0.006
P_{40}	-0.060	0.020	-0.020	-0.018	0.012
P_{41}	-0.037	0.037	0.000	0.000	0.011
P_{42}	-0.047	0.021	-0.007	-0.008	0.010
P_{43}	-0.039	0.039	0.000	0.000	0.015
P_{44}	-0.017	0.046	0.014	0.014	0.007

Figure S2.32 Ridgeplots for P_{lm} parameters (top) and statistical data for κ, κ', P_{val}, and P_{lm} parameters (bottom) for non-planar nitrogen atoms with three first neighbors (the 3n-N subgroup) for the refinement with symmetry constraints (ref-SC) in the Z (x1,x2) Y x1 R LCS type. The grey rectangle shows parts of P_{lm} approximated as zero with threshold 0.013.

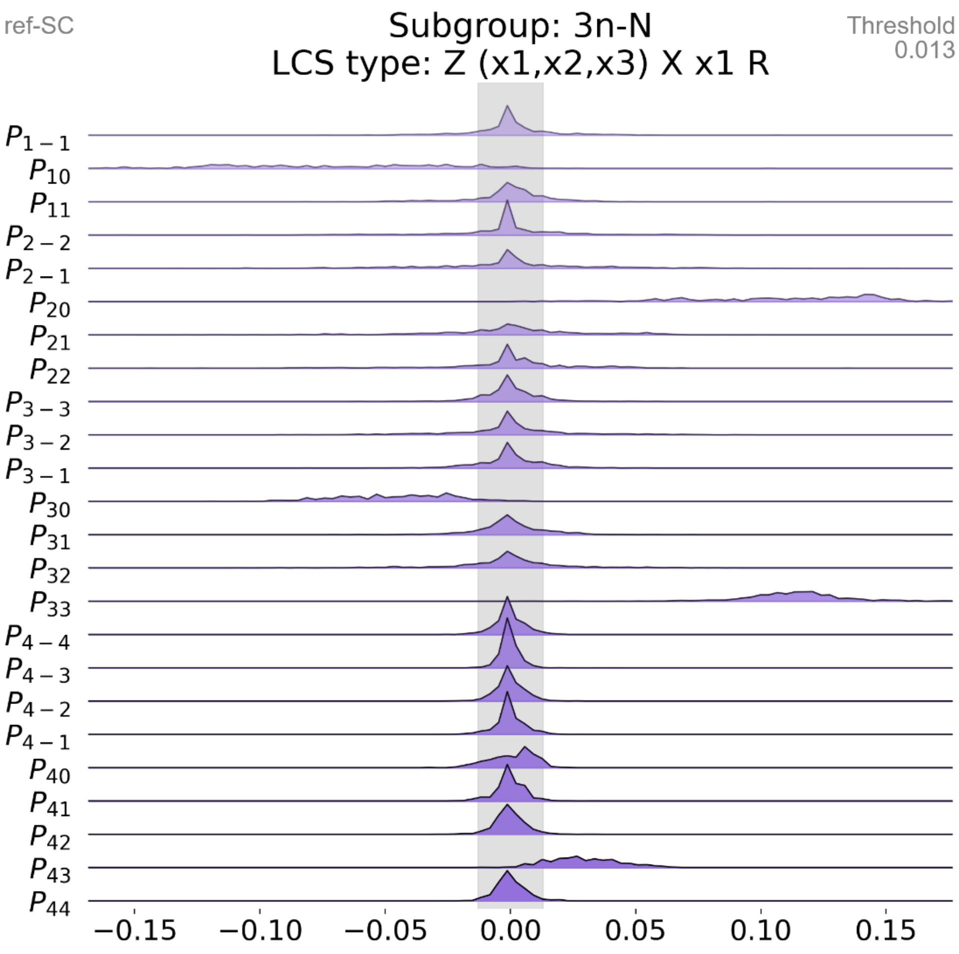

	min	max	median	mean	ssd
κ	0.9793	1.0149	0.9934	0.9931	0.0055
κ'	0.8899	1.1451	1.0307	1.0318	0.0368
P_{val}	4.764	5.265	5.031	5.033	0.078
P_{1-1}	-0.075	0.106	0.000	-0.000	0.015
P_{10}	-0.170	0.102	-0.064	-0.068	0.045
P_{11}	-0.080	0.104	0.000	-0.000	0.015
P_{2-2}	-0.155	0.102	0.000	0.000	0.024
P_{2-1}	-0.133	0.152	0.000	0.000	0.032
P_{20}	0.002	0.178	0.118	0.110	0.036
P_{21}	-0.155	0.100	0.001	-0.000	0.032
P_{22}	-0.143	0.135	0.000	0.000	0.025
P_{3-3}	-0.059	0.057	0.000	-0.000	0.011
P_{3-2}	-0.092	0.096	0.000	-0.000	0.022
P_{3-1}	-0.080	0.056	0.000	-0.000	0.013
P_{30}	-0.118	0.009	-0.043	-0.045	0.022
P_{31}	-0.070	0.073	0.000	0.000	0.013
P_{32}	-0.109	0.088	0.000	-0.001	0.023
P_{33}	0.006	0.178	0.114	0.114	0.019
P_{4-4}	-0.025	0.020	0.000	-0.000	0.006
P_{4-3}	-0.022	0.039	0.000	-0.000	0.004
P_{4-2}	-0.027	0.028	0.000	-0.000	0.006
P_{4-1}	-0.027	0.026	0.000	0.000	0.006
P_{40}	-0.033	0.023	0.002	0.001	0.009
P_{41}	-0.022	0.032	0.000	-0.000	0.006
P_{42}	-0.030	0.032	0.000	-0.000	0.006
P_{43}	-0.011	0.065	0.028	0.029	0.014
P_{44}	-0.026	0.024	0.000	0.000	0.006

Figure S2.33 Ridgeplots for P_{lm} parameters (top) and statistical data for κ, κ', P_{val}, and P_{lm} parameters (bottom) for non-planar nitrogen atoms with three first neighbors (the 3n-N subgroup) for the refinement with symmetry constraints (ref-SC) in the Z (x1,x2,x3) X x1 R LCS type. The grey rectangle shows parts of P_{lm} approximated as zero with threshold 0.013.

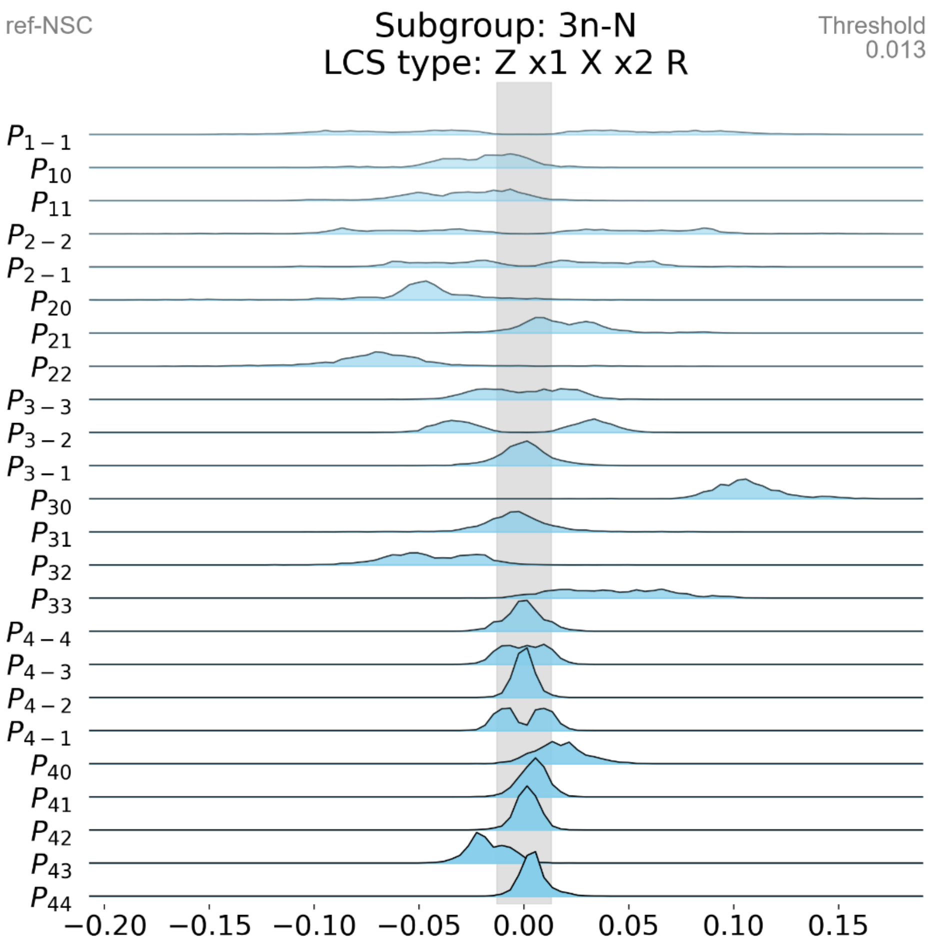

	min	max	median	mean	ssd
κ	0.9815	1.0149	0.9939	0.9935	0.0054
κ'	0.9170	1.1364	1.0393	1.0414	0.0370
P_{val}	4.741	5.198	5.025	5.020	0.072
P_{1-1}	-0.149	0.149	0.000	0.000	0.071
P_{10}	-0.113	0.043	-0.017	-0.020	0.022
P_{11}	-0.132	0.052	-0.026	-0.029	0.026
P_{2-2}	-0.185	0.173	-0.004	0.000	0.067
P_{2-1}	-0.115	0.122	0.000	-0.000	0.045
P_{20}	-0.209	0.057	-0.047	-0.049	0.031
P_{21}	-0.072	0.104	0.015	0.018	0.022
P_{22}	-0.172	0.091	-0.068	-0.067	0.029
P_{3-3}	-0.061	0.060	0.000	0.000	0.021
P_{3-2}	-0.069	0.071	-0.003	-0.000	0.034
P_{3-1}	-0.058	0.046	0.000	-0.000	0.012
P_{30}	0.021	0.192	0.105	0.107	0.017
P_{31}	-0.102	0.100	-0.004	-0.004	0.017
P_{32}	-0.119	0.049	-0.046	-0.044	0.021
P_{33}	-0.026	0.115	0.040	0.042	0.026
P_{4-4}	-0.032	0.031	0.000	0.000	0.009
P_{4-3}	-0.030	0.030	0.000	-0.000	0.010
P_{4-2}	-0.019	0.019	0.000	0.000	0.005
P_{4-1}	-0.028	0.028	0.000	-0.000	0.011
P_{40}	-0.021	0.056	0.016	0.017	0.012
P_{41}	-0.030	0.025	0.005	0.004	0.007
P_{42}	-0.024	0.025	0.002	0.002	0.006
P_{43}	-0.045	0.020	-0.018	-0.017	0.009
P_{44}	-0.019	0.039	0.004	0.004	0.007

Figure S2.34 Ridgeplots for P_{lm} parameters (top) and statistical data for κ, κ', P_{val}, and P_{lm} parameters (bottom) for non-planar nitrogen atoms with three first neighbors (the 3n-N subgroup) for the refinement without symmetry constraints (ref-NSC) in the Z x1 X x2 R LCS type. The grey rectangle shows parts of P_{lm} approximated as zero with threshold 0.013.

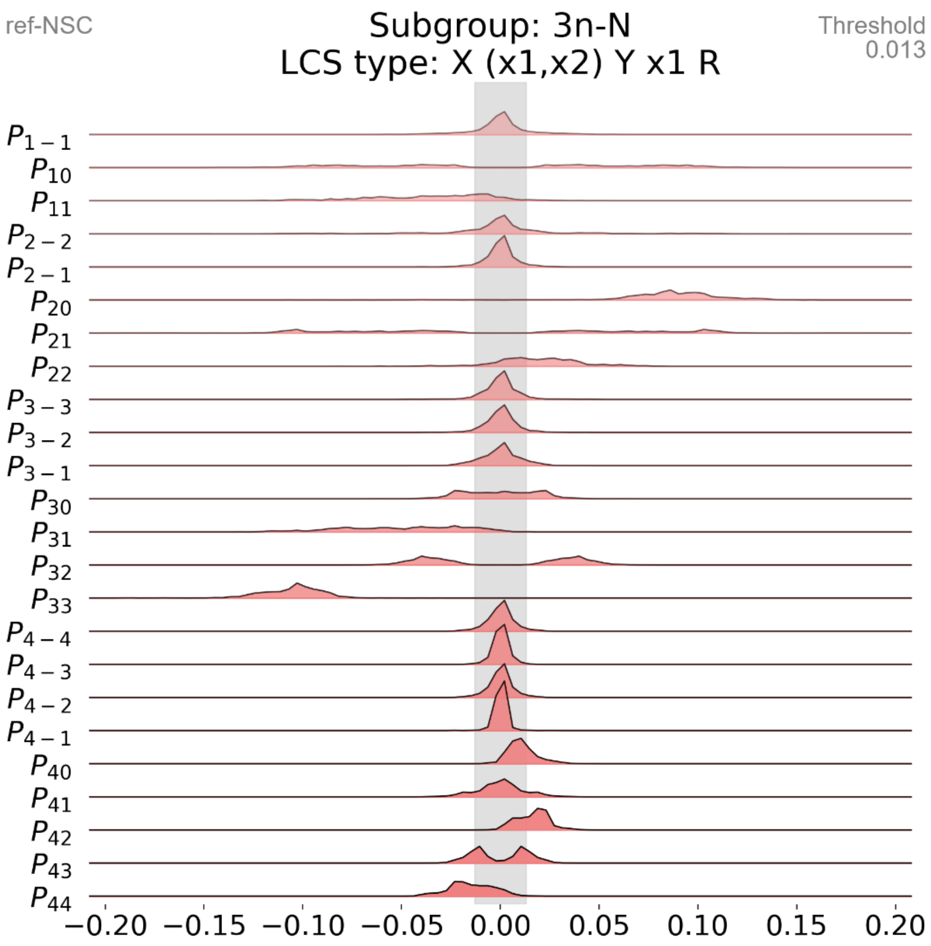

	min	max	median	mean	ssd
κ	0.9815	1.0149	0.9939	0.9935	0.0054
κ'	0.9170	1.1364	1.0393	1.0414	0.0370
P_{val}	4.741	5.198	5.025	5.020	0.072
P_{1-1}	-0.059	0.059	0.000	0.000	0.014
P_{10}	-0.149	0.149	0.000	0.000	0.071
P_{11}	-0.139	0.052	-0.030	-0.035	0.031
P_{2-2}	-0.151	0.151	0.000	0.000	0.030
P_{2-1}	-0.038	0.031	0.000	0.000	0.007
P_{20}	-0.108	0.159	0.087	0.085	0.030
P_{21}	-0.210	0.210	0.000	0.000	0.081
P_{22}	-0.083	0.082	0.018	0.019	0.021
P_{3-3}	-0.049	0.049	0.000	0.000	0.009
P_{3-2}	-0.038	0.043	0.000	0.000	0.008
P_{3-1}	-0.043	0.043	0.000	0.000	0.010
P_{30}	-0.066	0.066	0.000	0.000	0.018
P_{31}	-0.133	0.014	-0.045	-0.049	0.030
P_{32}	-0.076	0.076	0.000	0.000	0.038
P_{33}	-0.193	-0.030	-0.103	-0.105	0.016
P_{4-4}	-0.025	0.025	0.000	0.000	0.007
P_{4-3}	-0.017	0.017	0.000	-0.000	0.004
P_{4-2}	-0.023	0.023	0.000	0.000	0.006
P_{4-1}	-0.023	0.027	0.000	-0.000	0.003
P_{40}	-0.009	0.049	0.010	0.011	0.007
P_{41}	-0.042	0.042	0.000	0.000	0.012
P_{42}	-0.009	0.044	0.017	0.016	0.008
P_{43}	-0.035	0.035	0.000	0.000	0.014
P_{44}	-0.046	0.018	-0.015	-0.014	0.011

Figure S2.35 Ridgeplots for P_{lm} parameters (top) and statistical data for κ, κ', P_{val}, and P_{lm} parameters (bottom) for non-planar nitrogen atoms with three first neighbors (the 3n-N subgroup) for the refinement without symmetry constraints (ref-NSC) in the X (x1,x2) Y x1 R LCS type. The grey rectangle shows parts of P_{lm} approximated as zero with threshold 0.013.

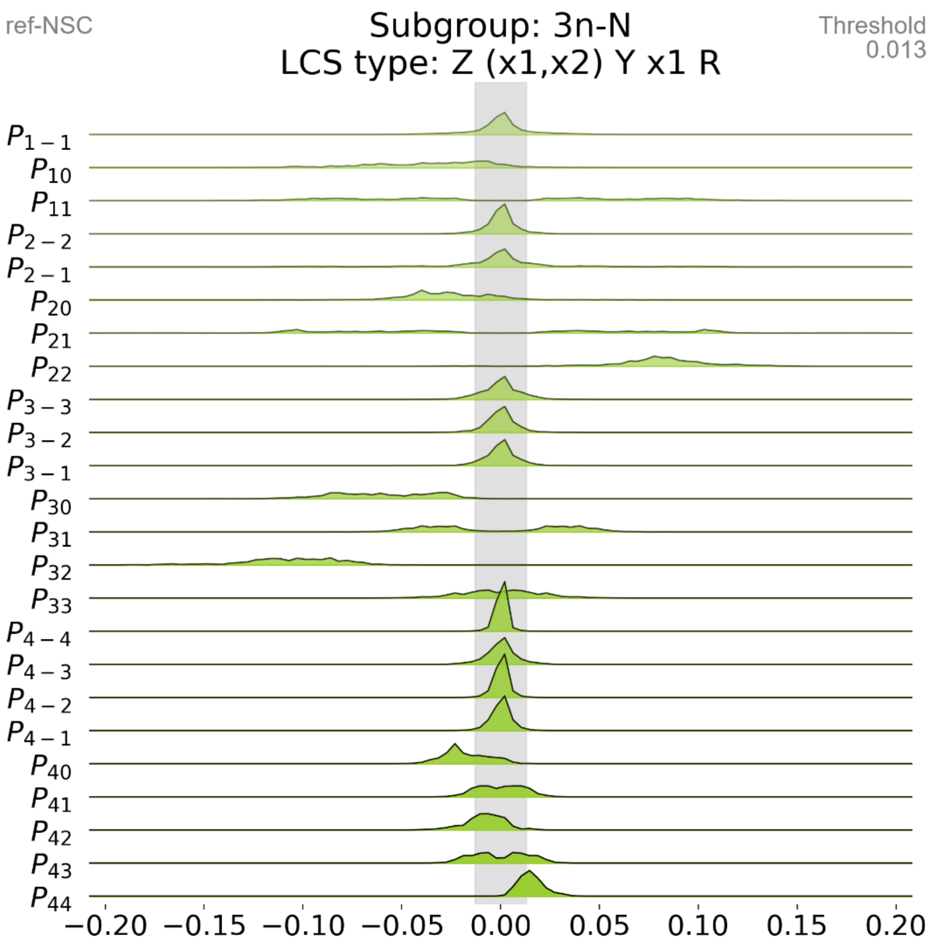

	min	max	median	mean	ssd
κ	0.9815	1.0149	0.9939	0.9935	0.0054
κ'	0.9170	1.1364	1.0393	1.0414	0.0370
P_{val}	4.741	5.198	5.025	5.020	0.072
P_{1-1}	-0.059	0.059	0.000	0.000	0.014
P_{10}	-0.139	0.052	-0.030	-0.035	0.031
P_{11}	-0.149	0.149	0.000	0.000	0.071
P_{2-2}	-0.031	0.038	0.000	-0.000	0.007
P_{2-1}	-0.151	0.151	0.000	0.000	0.030
P_{20}	-0.121	0.088	-0.027	-0.025	0.024
P_{21}	-0.210	0.210	0.000	0.000	0.081
P_{22}	-0.089	0.159	0.081	0.080	0.028
P_{3-3}	-0.044	0.044	0.000	0.000	0.009
P_{3-2}	-0.043	0.038	0.000	-0.000	0.008
P_{3-1}	-0.054	0.054	0.000	0.000	0.009
P_{30}	-0.151	0.011	-0.060	-0.059	0.026
P_{31}	-0.087	0.087	0.000	0.000	0.036
P_{32}	-0.187	-0.046	-0.104	-0.106	0.024
P_{33}	-0.061	0.061	0.000	0.000	0.020
P_{4-4}	-0.025	0.021	0.000	0.000	0.003
P_{4-3}	-0.028	0.028	0.000	0.000	0.007
P_{4-2}	-0.017	0.018	0.000	0.000	0.004
P_{4-1}	-0.024	0.024	0.000	0.000	0.005
P_{40}	-0.051	0.015	-0.020	-0.018	0.011
P_{41}	-0.036	0.036	0.000	0.000	0.011
P_{42}	-0.046	0.020	-0.008	-0.008	0.009
P_{43}	-0.034	0.034	0.000	0.000	0.014
P_{44}	-0.002	0.039	0.015	0.015	0.006

Figure S2.36 Ridgeplots for P_{lm} parameters (top) and statistical data for κ, κ', P_{val}, and P_{lm} parameters (bottom) for non-planar nitrogen atoms with three first neighbors (the 3n-N subgroup) for the refinement without symmetry constraints (ref-NSC) in the Z (x1,x2) Y x1 R LCS type. The grey rectangle shows parts of P_{lm} approximated as zero with threshold 0.013.

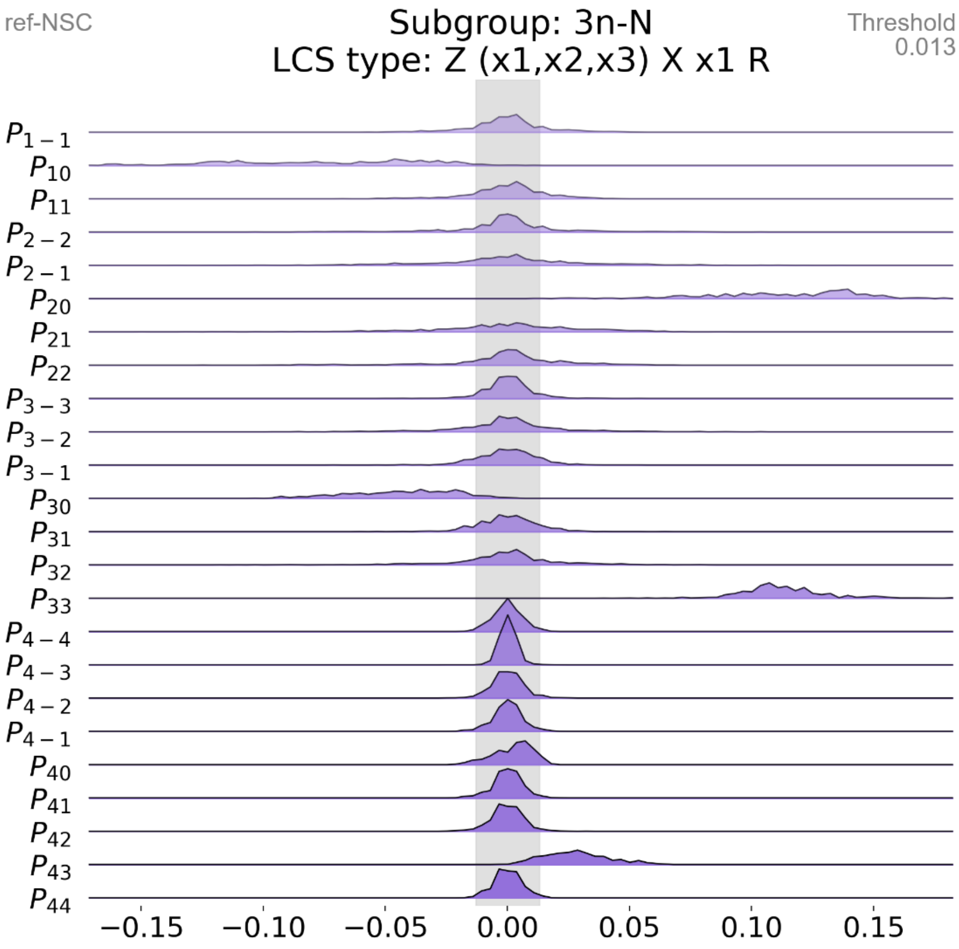

	min	max	median	mean	ssd
κ	0.9815	1.0149	0.9939	0.9935	0.0054
κ'	0.9170	1.1364	1.0393	1.0414	0.0370
P_{val}	4.741	5.198	5.025	5.020	0.072
P_{1-1}	-0.056	0.054	0.000	0.000	0.015
P_{10}	-0.173	0.012	-0.069	-0.073	0.040
P_{11}	-0.067	0.047	0.001	0.000	0.015
P_{2-2}	-0.105	0.104	0.000	0.000	0.023
P_{2-1}	-0.134	0.136	0.000	0.000	0.032
P_{20}	0.012	0.179	0.118	0.114	0.031
P_{21}	-0.150	0.089	0.001	-0.001	0.031
P_{22}	-0.124	0.068	0.001	0.000	0.023
P_{3-3}	-0.051	0.053	0.000	0.000	0.009
P_{3-2}	-0.098	0.104	0.000	0.000	0.022
P_{3-1}	-0.070	0.052	0.000	-0.000	0.012
P_{30}	-0.104	0.003	-0.040	-0.043	0.022
P_{31}	-0.060	0.064	0.000	0.000	0.012
P_{32}	-0.118	0.081	0.000	-0.001	0.023
P_{33}	0.041	0.184	0.111	0.113	0.017
P_{4-4}	-0.017	0.018	0.000	-0.000	0.006
P_{4-3}	-0.025	0.021	0.000	0.000	0.003
P_{4-2}	-0.025	0.025	0.000	0.000	0.006
P_{4-1}	-0.021	0.020	0.000	-0.000	0.006
P_{40}	-0.022	0.019	0.004	0.002	0.008
P_{41}	-0.022	0.023	0.000	-0.000	0.006
P_{42}	-0.026	0.031	0.000	-0.000	0.006
P_{43}	-0.003	0.065	0.027	0.028	0.013
P_{44}	-0.017	0.017	0.000	0.000	0.006

Figure S2.37 Ridgeplots for P_{lm} parameters (top) and statistical data for κ, κ', P_{val}, and P_{lm} parameters (bottom) for non-planar nitrogen atoms with three first neighbors (the 3n-N subgroup) for the refinement without symmetry constraints (ref-NSC) in the Z (x1,x2,x3) X x1 R LCS type. The grey rectangle shows parts of P_{lm} approximated as zero with threshold 0.013.

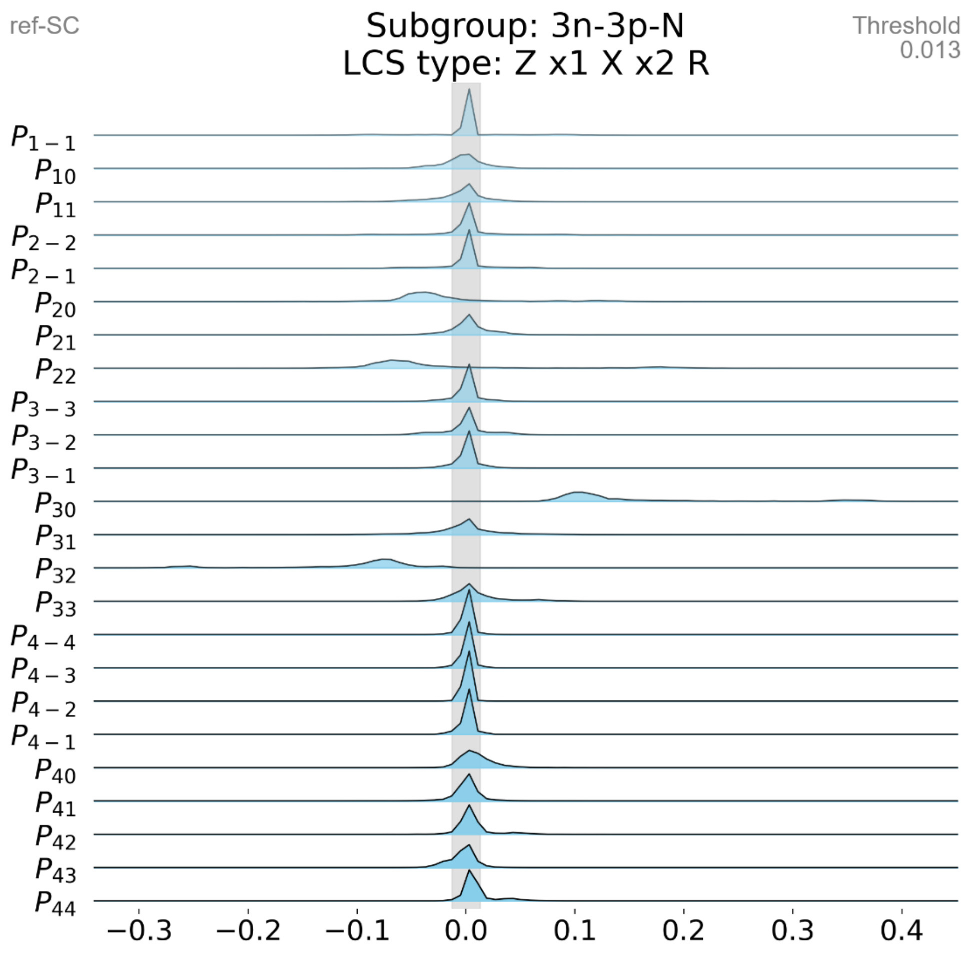

	min	max	median	mean	ssd
κ	0.9793	1.0193	0.9948	0.9946	0.0057
κ'	0.7100	1.2176	1.0213	0.9844	0.1102
P_{val}	4.667	5.320	5.016	5.013	0.080
P_{1-1}	-0.149	0.149	0.000	0.000	0.031
P_{10}	-0.113	0.126	-0.002	-0.003	0.022
P_{11}	-0.132	0.117	-0.001	-0.005	0.024
P_{2-2}	-0.176	0.174	0.000	0.000	0.031
P_{2-1}	-0.116	0.117	0.000	-0.000	0.022
P_{20}	-0.233	0.222	-0.033	-0.019	0.054
P_{21}	-0.178	0.178	0.001	0.004	0.021
P_{22}	-0.201	0.293	-0.057	-0.029	0.080
P_{3-3}	-0.090	0.064	0.000	-0.000	0.011
P_{3-2}	-0.086	0.097	0.000	0.000	0.019
P_{3-1}	-0.062	0.061	0.000	0.000	0.010
P_{30}	-0.006	0.455	0.114	0.147	0.081
P_{31}	-0.254	0.254	0.000	-0.001	0.030
P_{32}	-0.345	0.058	-0.080	-0.101	0.066
P_{33}	-0.077	0.116	0.002	0.009	0.024
P_{4-4}	-0.036	0.033	0.000	0.000	0.005
P_{4-3}	-0.033	0.031	0.000	0.000	0.005
P_{4-2}	-0.021	0.024	0.000	-0.000	0.002
P_{4-1}	-0.029	0.034	0.000	0.000	0.006
P_{40}	-0.049	0.121	0.008	0.010	0.015
P_{41}	-0.112	0.112	0.000	0.001	0.014
P_{42}	-0.066	0.121	0.004	0.007	0.015
P_{43}	-0.061	0.059	-0.001	-0.003	0.012
P_{44}	-0.025	0.103	0.006	0.010	0.014

Figure S2.38 Ridgeplots for P_{lm} parameters (top) and statistical data for κ, κ', P_{val}, and P_{lm} parameters (bottom) for non-planar and planar nitrogen atoms with three first neighbors (the 3n-3p-N subgroup) for the refinement with symmetry constraints (ref-SC) in the Z x1 X x2 R LCS type. The grey rectangle shows parts of P_{lm} approximated as zero with threshold 0.013.

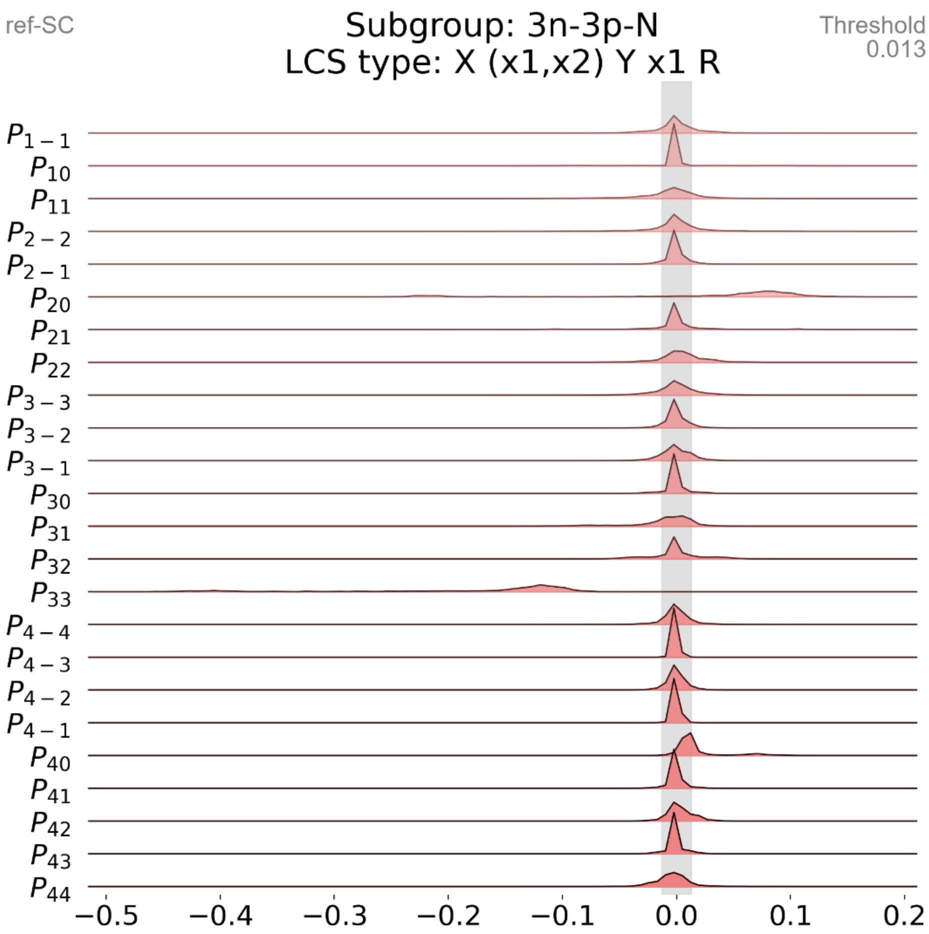

	min	max	median	mean	ssd
κ	0.9793	1.0193	0.9948	0.9946	0.0057
κ'	0.7100	1.2176	1.0213	0.9844	0.1102
P_{val}	4.667	5.320	5.016	5.013	0.080
P_{1-1}	-0.126	0.126	0.000	0.000	0.019
P_{10}	-0.149	0.149	0.000	0.000	0.031
P_{11}	-0.138	0.113	-0.003	-0.006	0.026
P_{2-2}	-0.214	0.214	0.000	0.000	0.023
P_{2-1}	-0.062	0.046	0.000	0.000	0.007
P_{20}	-0.261	0.199	0.071	0.036	0.097
P_{21}	-0.210	0.210	0.000	0.000	0.037
P_{22}	-0.221	0.099	0.003	0.004	0.021
P_{3-3}	-0.156	0.156	0.000	0.000	0.017
P_{3-2}	-0.083	0.061	0.000	-0.000	0.009
P_{3-1}	-0.073	0.073	0.000	0.000	0.013
P_{30}	-0.066	0.066	0.000	0.000	0.008
P_{31}	-0.135	0.058	-0.004	-0.010	0.027
P_{32}	-0.090	0.090	0.000	0.000	0.021
P_{33}	-0.519	-0.018	-0.128	-0.168	0.096
P_{4-4}	-0.125	0.125	0.000	0.000	0.013
P_{4-3}	-0.024	0.026	0.000	0.000	0.002
P_{4-2}	-0.046	0.046	0.000	0.000	0.009
P_{4-1}	-0.028	0.028	0.000	0.000	0.003
P_{40}	-0.014	0.116	0.010	0.016	0.020
P_{41}	-0.045	0.045	0.000	0.000	0.006
P_{42}	-0.043	0.060	0.002	0.003	0.011
P_{43}	-0.037	0.037	0.000	0.000	0.006
P_{44}	-0.127	0.104	-0.003	-0.004	0.015

Figure S2.39 Ridgeplots for P_{lm} parameters (top) and statistical data for κ, κ', P_{val}, and P_{lm} parameters (bottom) for non-planar and planar nitrogen atoms with three first neighbors (the 3n-3p-N subgroup) for the refinement with symmetry constraints (ref-SC) in the X (x1,x2) Y x1 R LCS type. The grey rectangle shows parts of P_{lm} approximated as zero with threshold 0.013.

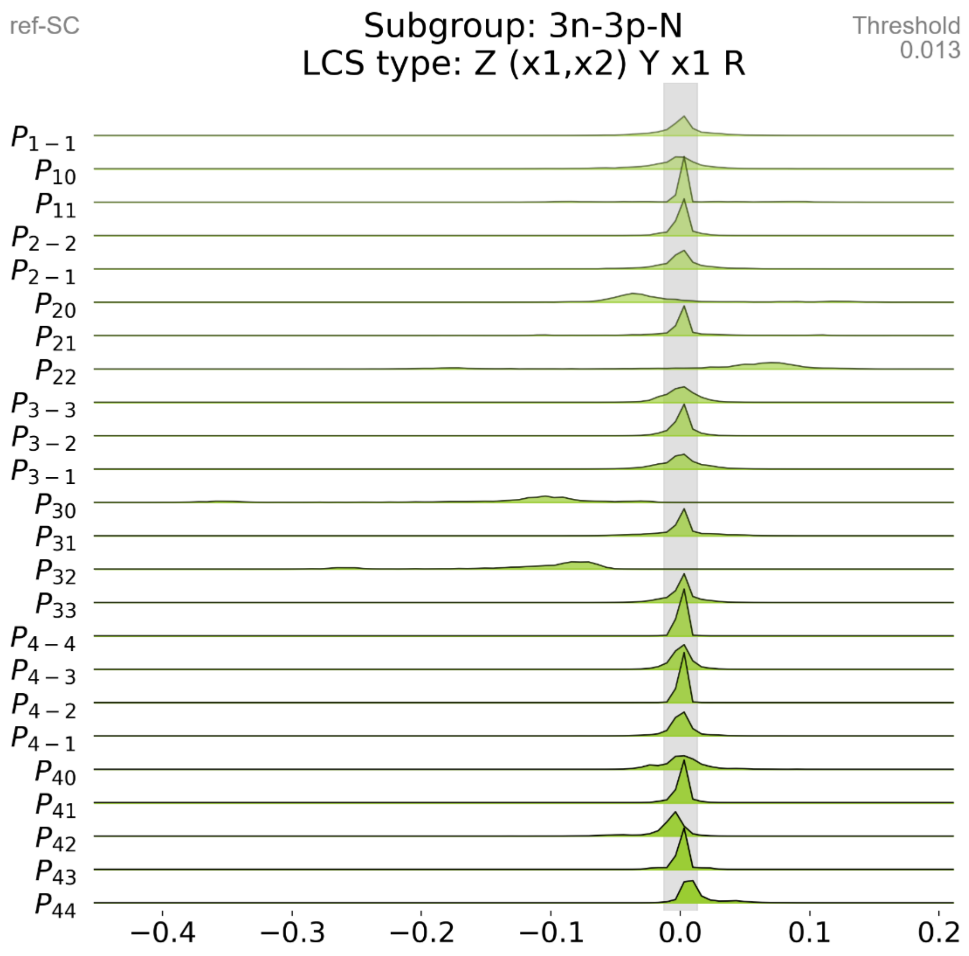

	min	max	median	mean	ssd
κ	0.9793	1.0193	0.9948	0.9946	0.0057
κ'	0.7100	1.2176	1.0213	0.9844	0.1102
P_{val}	4.667	5.320	5.016	5.013	0.080
P_{1-1}	-0.126	0.126	0.000	0.000	0.019
P_{10}	-0.138	0.113	-0.003	-0.006	0.026
P_{11}	-0.149	0.149	0.000	0.000	0.031
P_{2-2}	-0.046	0.062	0.000	-0.000	0.007
P_{2-1}	-0.214	0.214	0.000	0.000	0.023
P_{20}	-0.167	0.192	-0.030	-0.015	0.050
P_{21}	-0.210	0.210	0.000	0.000	0.037
P_{22}	-0.292	0.193	0.059	0.032	0.082
P_{3-3}	-0.080	0.080	0.000	0.000	0.013
P_{3-2}	-0.061	0.083	0.000	0.000	0.009
P_{3-1}	-0.156	0.156	0.000	0.000	0.017
P_{30}	-0.456	0.007	-0.110	-0.139	0.089
P_{31}	-0.086	0.086	0.000	0.000	0.019
P_{32}	-0.344	-0.002	-0.092	-0.115	0.059
P_{33}	-0.067	0.067	0.000	0.000	0.012
P_{4-4}	-0.017	0.018	0.000	-0.000	0.002
P_{4-3}	-0.051	0.051	0.000	0.000	0.009
P_{4-2}	-0.033	0.033	0.000	-0.000	0.002
P_{4-1}	-0.130	0.130	0.000	0.000	0.013
P_{40}	-0.068	0.103	0.001	0.001	0.017
P_{41}	-0.037	0.037	0.000	0.000	0.005
P_{42}	-0.132	0.054	-0.006	-0.009	0.015
P_{43}	-0.039	0.039	0.000	0.000	0.007
P_{44}	-0.025	0.103	0.008	0.012	0.014

Figure S2.40 Ridgeplots for P_{lm} parameters (top) and statistical data for κ, κ', P_{val}, and P_{lm} parameters (bottom) for non-planar and planar nitrogen atoms with three first neighbors (the 3n-3p-N subgroup) for the refinement with symmetry constraints (ref-SC) in the Z (x1,x2) Y x1 R LCS type. The grey rectangle shows parts of P_{lm} approximated as zero with threshold 0.013.

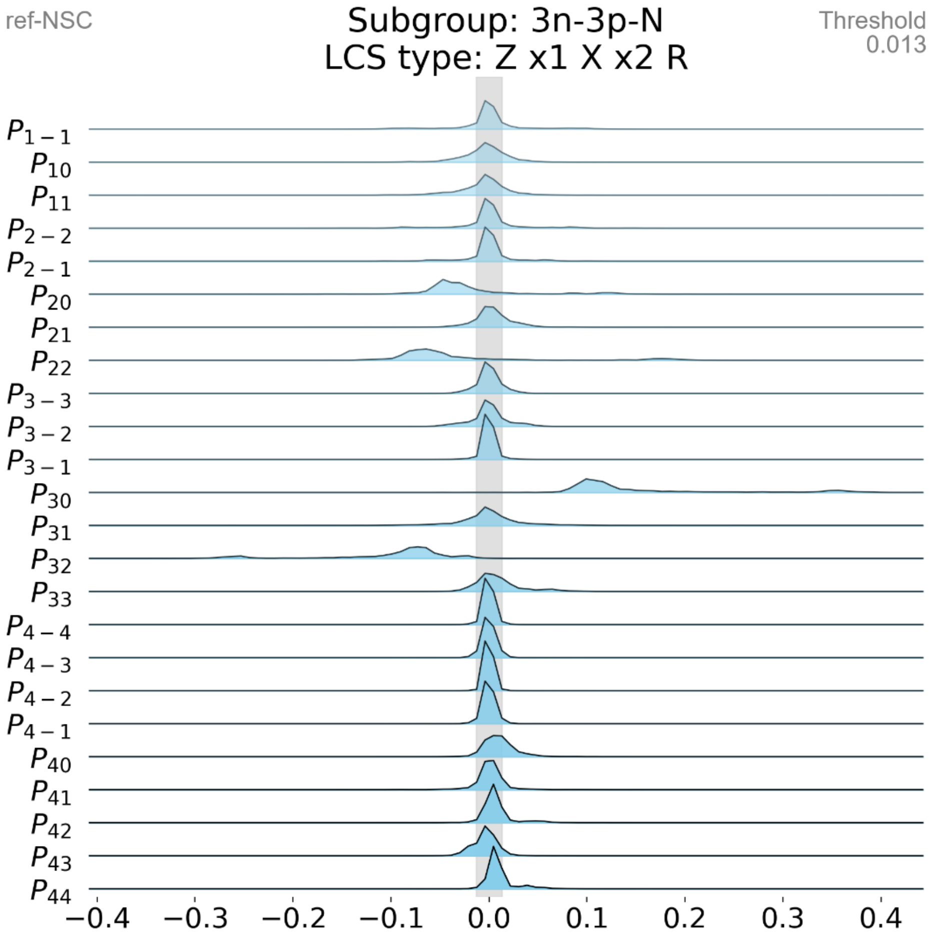

	min	max	median	mean	ssd
κ	0.9781	1.0217	0.9950	0.9947	0.0058
κ'	0.7088	1.8155	1.0250	0.9874	0.1138
P_{val}	4.663	5.372	5.010	5.005	0.079
P_{1-1}	-0.149	0.149	0.000	0.000	0.033
P_{10}	-0.268	0.436	-0.003	-0.004	0.023
P_{11}	-0.412	0.410	-0.003	-0.006	0.025
P_{2-2}	-0.185	0.173	0.000	0.000	0.031
P_{2-1}	-0.115	0.122	0.000	-0.000	0.021
P_{20}	-0.241	0.241	-0.035	-0.020	0.054
P_{21}	-0.179	0.179	0.002	0.004	0.021
P_{22}	-0.236	0.292	-0.058	-0.031	0.080
P_{3-3}	-0.061	0.060	0.000	0.000	0.012
P_{3-2}	-0.069	0.071	0.000	-0.000	0.018
P_{3-1}	-0.058	0.052	0.000	-0.000	0.006
P_{30}	-0.048	0.447	0.114	0.148	0.082
P_{31}	-0.258	0.258	-0.001	-0.001	0.030
P_{32}	-0.339	0.049	-0.080	-0.101	0.066
P_{33}	-0.086	0.115	0.004	0.009	0.024
P_{4-4}	-0.147	0.080	0.000	-0.000	0.005
P_{4-3}	-0.091	0.087	0.000	0.000	0.007
P_{4-2}	-0.046	0.097	0.000	0.000	0.004
P_{4-1}	-0.114	0.111	0.000	-0.000	0.006
P_{40}	-0.109	0.130	0.008	0.010	0.015
P_{41}	-0.149	0.152	0.001	0.001	0.014
P_{42}	-0.110	0.140	0.005	0.007	0.015
P_{43}	-0.084	0.079	-0.003	-0.004	0.012
P_{44}	-0.079	0.161	0.007	0.010	0.014

Figure S2.41 Ridgeplots for P_{lm} parameters (top) and statistical data for κ, κ', P_{val}, and P_{lm} parameters (bottom) for non-planar and planar nitrogen atoms with three first neighbors (the 3n-3p-N subgroup) for the refinement without symmetry constraints (ref-NSC) in the Z x1 X x2 R LCS type. The grey rectangle shows parts of P_{lm} approximated as zero with threshold 0.013.

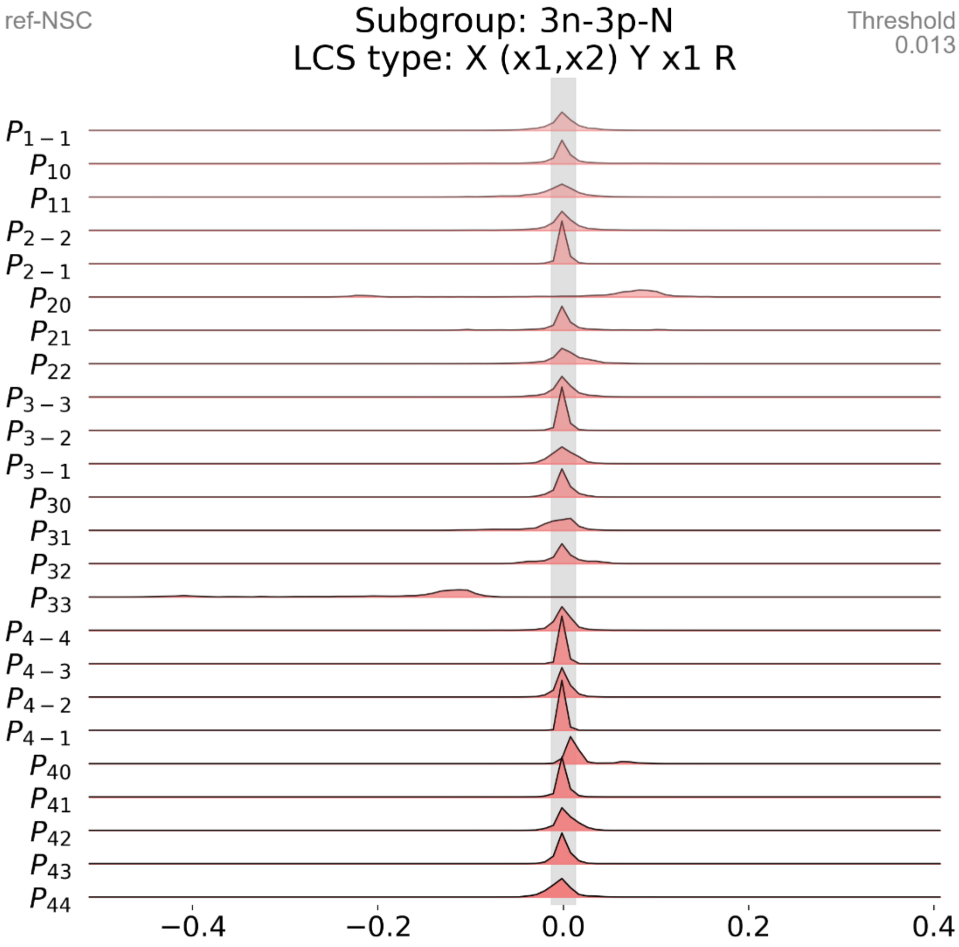

	min	max	median	mean	ssd
κ	0.9781	1.0217	0.9950	0.9947	0.0058
κ'	0.7088	1.8155	1.0250	0.9874	0.1138
P_{val}	4.663	5.372	5.010	5.005	0.079
P_{1-1}	-0.411	0.411	0.000	0.000	0.020
P_{10}	-0.149	0.149	0.000	0.000	0.033
P_{11}	-0.438	0.275	-0.004	-0.007	0.027
P_{2-2}	-0.214	0.214	0.000	0.000	0.023
P_{2-1}	-0.067	0.047	0.000	0.000	0.005
P_{20}	-0.266	0.200	0.073	0.038	0.097
P_{21}	-0.210	0.210	0.000	0.000	0.037
P_{22}	-0.222	0.146	0.003	0.004	0.022
P_{3-3}	-0.154	0.154	0.000	0.000	0.016
P_{3-2}	-0.048	0.049	0.000	0.000	0.005
P_{3-1}	-0.087	0.087	0.000	0.000	0.013
P_{30}	-0.066	0.066	0.000	0.000	0.010
P_{31}	-0.133	0.061	-0.005	-0.011	0.027
P_{32}	-0.076	0.076	0.000	0.000	0.020
P_{33}	-0.516	0.039	-0.128	-0.168	0.097
P_{4-4}	-0.157	0.157	0.000	0.000	0.013
P_{4-3}	-0.084	0.044	0.000	-0.000	0.004
P_{4-2}	-0.066	0.066	0.000	0.000	0.009
P_{4-1}	-0.113	0.088	0.000	-0.000	0.004
P_{40}	-0.064	0.135	0.011	0.017	0.019
P_{41}	-0.106	0.106	0.000	0.000	0.006
P_{42}	-0.092	0.085	0.002	0.004	0.011
P_{43}	-0.057	0.057	0.000	0.000	0.008
P_{44}	-0.131	0.157	-0.004	-0.004	0.015

Figure S2.42 Ridgeplots for P_{lm} parameters (top) and statistical data for κ, κ', P_{val}, and P_{lm} parameters (bottom) for non-planar and planar nitrogen atoms with three first neighbors (the 3n-3p-N subgroup) for the refinement without symmetry constraints (ref-NSC) in the X (x1,x2) Y x1 R LCS type. The grey rectangle shows parts of P_{lm} approximated as zero with threshold 0.013.

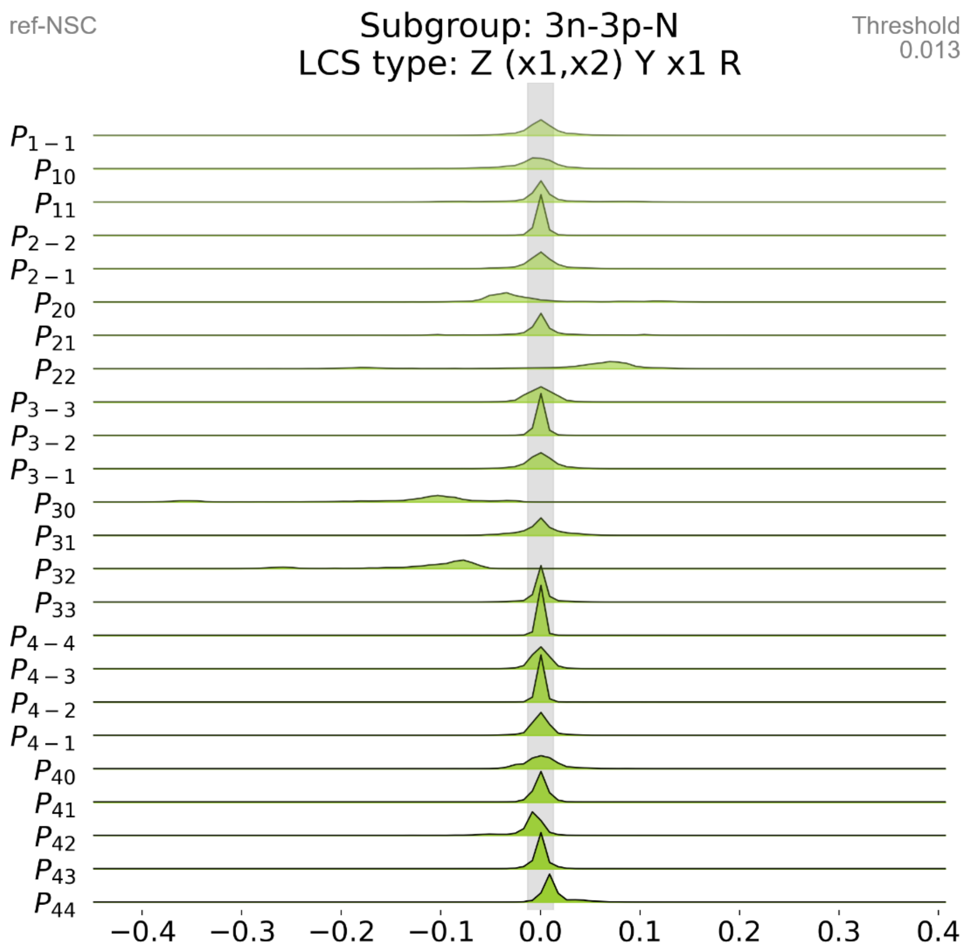

	min	max	median	mean	ssd
κ	0.9781	1.0217	0.9950	0.9947	0.0058
κ'	0.7088	1.8155	1.0250	0.9874	0.1138
P_{val}	4.663	5.372	5.010	5.005	0.079
P_{1-1}	-0.411	0.411	0.000	0.000	0.020
P_{10}	-0.438	0.275	-0.004	-0.007	0.027
P_{11}	-0.149	0.149	0.000	0.000	0.033
P_{2-2}	-0.047	0.067	0.000	-0.000	0.005
P_{2-1}	-0.214	0.214	0.000	0.000	0.023
P_{20}	-0.222	0.215	-0.031	-0.015	0.050
P_{21}	-0.210	0.210	0.000	0.000	0.037
P_{22}	-0.292	0.229	0.061	0.033	0.082
P_{3-3}	-0.089	0.089	0.000	0.000	0.014
P_{3-2}	-0.049	0.048	0.000	-0.000	0.005
P_{3-1}	-0.154	0.154	0.000	0.000	0.016
P_{30}	-0.453	0.057	-0.109	-0.139	0.090
P_{31}	-0.087	0.087	0.000	0.000	0.019
P_{32}	-0.341	0.036	-0.094	-0.116	0.060
P_{33}	-0.061	0.061	0.000	0.000	0.010
P_{4-4}	-0.076	0.119	0.000	0.000	0.003
P_{4-3}	-0.081	0.081	0.000	0.000	0.010
P_{4-2}	-0.037	0.110	0.000	0.000	0.004
P_{4-1}	-0.146	0.146	0.000	0.000	0.013
P_{40}	-0.129	0.136	0.000	0.001	0.018
P_{41}	-0.093	0.093	0.000	0.000	0.007
P_{42}	-0.132	0.116	-0.007	-0.010	0.015
P_{43}	-0.077	0.077	0.000	0.000	0.007
P_{44}	-0.066	0.130	0.009	0.013	0.014

Figure S2.43 Ridgeplots for P_{lm} parameters (top) and statistical data for κ, κ', P_{val}, and P_{lm} parameters (bottom) for non-planar and planar nitrogen atoms with three first neighbors (the 3n-3p-N subgroup) for the refinement without symmetry constraints (ref-NSC) in the Z (x1,x2) Y x1 R LCS type. The grey rectangle shows parts of P_{lm} approximated as zero with threshold 0.013.

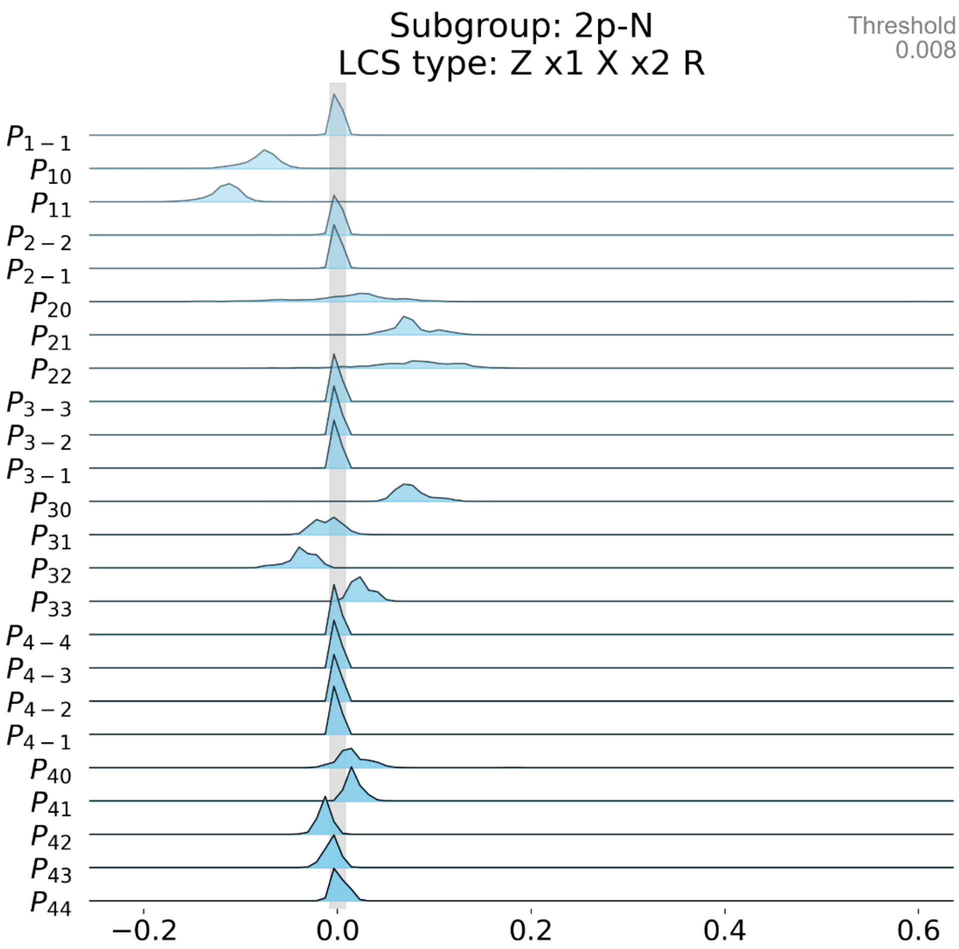

	min	max	median	mean	ssd
κ	0.9754	1.0053	0.9888	0.9891	0.0032
κ'	0.6486	1.1734	1.0476	1.0471	0.0313
P_{val}	4.726	5.555	5.029	5.025	0.065
P_{1-1}	-0.036	0.036	0.000	0.000	0.004
P_{10}	-0.260	0.259	-0.076	-0.078	0.021
P_{11}	-0.188	0.014	-0.114	-0.116	0.016
P_{2-2}	-0.072	0.047	0.000	0.000	0.005
P_{2-1}	-0.033	0.065	0.000	-0.000	0.004
P_{20}	-0.151	0.640	0.018	0.012	0.055
P_{21}	-0.091	0.154	0.073	0.077	0.022
P_{22}	-0.092	0.191	0.083	0.079	0.042
P_{3-3}	-0.009	0.007	0.000	0.000	0.002
P_{3-2}	-0.014	0.011	0.000	-0.000	0.002
P_{3-1}	-0.009	0.009	-0.000	-0.000	0.002
P_{30}	-0.067	0.158	0.074	0.077	0.017
P_{31}	-0.047	0.049	-0.010	-0.010	0.014
P_{32}	-0.093	0.050	-0.037	-0.037	0.015
P_{33}	-0.008	0.057	0.022	0.025	0.010
P_{4-4}	-0.006	0.006	-0.000	-0.000	0.002
P_{4-3}	-0.008	0.008	0.000	-0.000	0.002
P_{4-2}	-0.007	0.007	0.000	0.000	0.002
P_{4-1}	-0.012	0.006	0.000	-0.000	0.002
P_{40}	-0.025	0.187	0.013	0.016	0.017
P_{41}	-0.041	0.063	0.016	0.017	0.008
P_{42}	-0.041	0.011	-0.013	-0.013	0.006
P_{43}	-0.030	0.018	-0.006	-0.006	0.007
P_{44}	-0.015	0.025	0.001	0.002	0.007

Figure S2.44 Ridgeplots for P_{lm} parameters (top) and statistical data for κ, κ', P_{val}, and P_{lm} parameters (bottom) for planar nitrogen atoms with two first neighbors (the 2p-N subgroup) in the Z x1 X x2 R LCS type. The grey rectangle shows parts of P_{lm} approximated as zero with threshold 0.008.

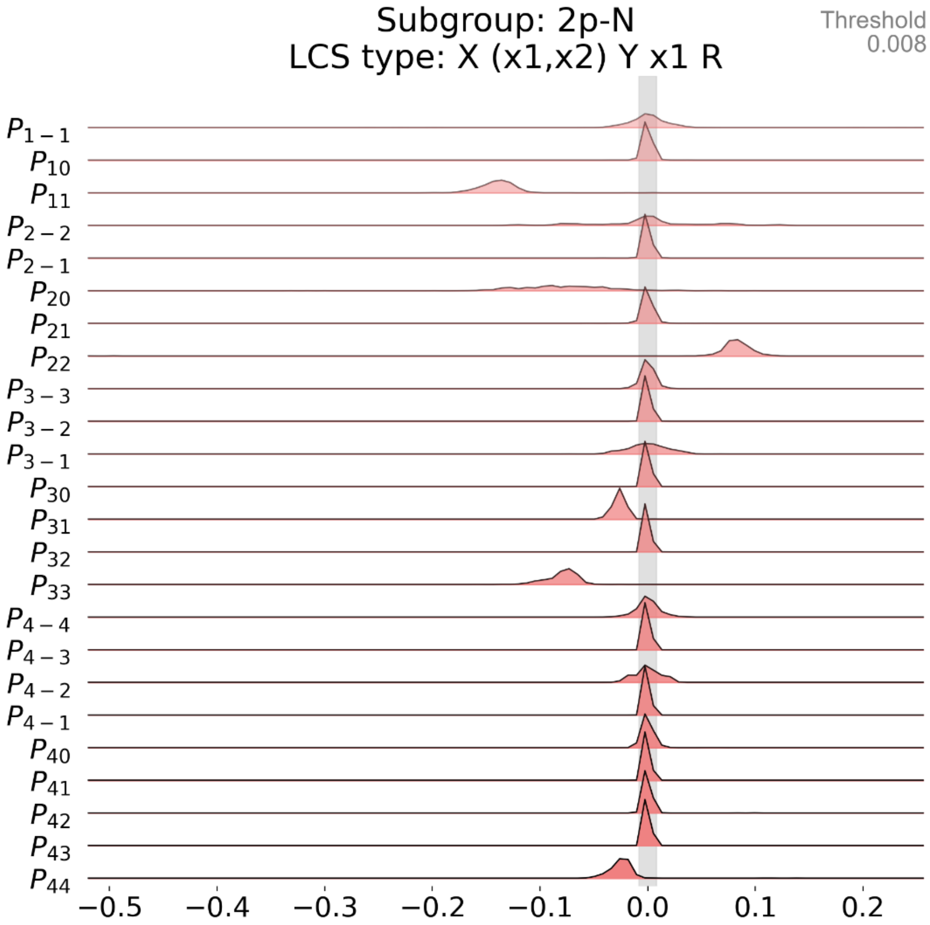

	min	max	median	mean	ssd
κ	0.9754	1.0053	0.9888	0.9891	0.0032
κ'	0.6486	1.1734	1.0476	1.0471	0.0313
P_{val}	4.726	5.555	5.029	5.025	0.065
P_{1-1}	-0.260	0.260	0.000	0.000	0.019
P_{10}	-0.036	0.036	0.000	0.000	0.004
P_{11}	-0.207	0.005	-0.139	-0.140	0.017
P_{2-2}	-0.134	0.134	0.000	0.000	0.050
P_{2-1}	-0.039	0.015	-0.000	0.000	0.003
P_{20}	-0.330	0.100	-0.078	-0.078	0.042
P_{21}	-0.060	0.060	0.000	0.000	0.005
P_{22}	-0.523	0.126	0.082	0.082	0.032
P_{3-3}	-0.063	0.063	0.000	0.000	0.006
P_{3-2}	-0.013	0.014	0.000	0.000	0.002
P_{3-1}	-0.070	0.070	0.000	0.000	0.018
P_{30}	-0.009	0.009	0.000	0.000	0.002
P_{31}	-0.045	0.007	-0.026	-0.026	0.006
P_{32}	-0.012	0.012	0.000	0.000	0.002
P_{33}	-0.159	-0.004	-0.077	-0.080	0.015
P_{4-4}	-0.040	0.040	0.000	0.000	0.010
P_{4-3}	-0.007	0.008	-0.000	-0.000	0.002
P_{4-2}	-0.032	0.032	0.000	0.000	0.012
P_{4-1}	-0.010	0.006	-0.000	-0.000	0.002
P_{40}	-0.014	0.067	-0.001	-0.000	0.006
P_{41}	-0.008	0.008	0.000	0.000	0.002
P_{42}	-0.012	0.098	-0.000	0.000	0.006
P_{43}	-0.009	0.009	0.000	0.000	0.002
P_{44}	-0.060	0.141	-0.024	-0.025	0.012

Figure S2.45 Ridgeplots for P_{lm} parameters (top) and statistical data for κ, κ', P_{val}, and P_{lm} parameters (bottom) for planar nitrogen atoms with two first neighbors (the 2p-N subgroup) in the X (x1,x2) Y x1 R LCS type. The grey rectangle shows parts of P_{lm} approximated as zero with threshold 0.008.

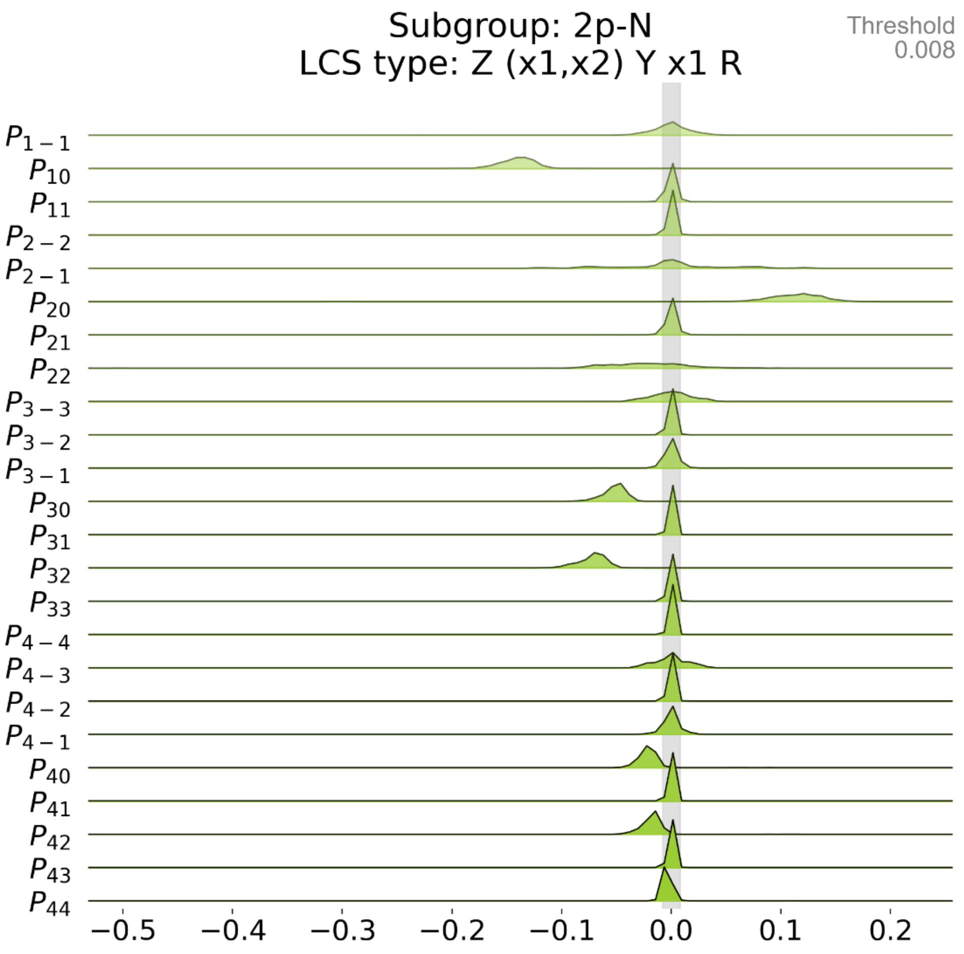

	min	max	median	mean	ssd
κ	0.9754	1.0053	0.9888	0.9891	0.0032
κ'	0.6486	1.1734	1.0476	1.0471	0.0313
P_{val}	4.726	5.555	5.029	5.025	0.065
P_{1-1}	-0.260	0.260	0.000	0.000	0.019
P_{10}	-0.207	0.005	-0.139	-0.140	0.017
P_{11}	-0.036	0.036	0.000	0.000	0.004
P_{2-2}	-0.015	0.039	0.000	-0.000	0.003
P_{2-1}	-0.134	0.134	-0.000	-0.000	0.050
P_{20}	-0.310	0.186	0.116	0.113	0.031
P_{21}	-0.060	0.060	0.000	0.000	0.005
P_{22}	-0.535	0.109	-0.023	-0.023	0.042
P_{3-3}	-0.068	0.068	-0.000	-0.000	0.017
P_{3-2}	-0.014	0.013	-0.000	-0.000	0.002
P_{3-1}	-0.058	0.058	0.000	0.000	0.006
P_{30}	-0.110	-0.008	-0.050	-0.052	0.010
P_{31}	-0.008	0.008	0.000	0.000	0.002
P_{32}	-0.135	0.003	-0.070	-0.072	0.013
P_{33}	-0.012	0.012	0.000	0.000	0.002
P_{4-4}	-0.006	0.011	0.000	0.000	0.001
P_{4-3}	-0.042	0.042	-0.000	-0.000	0.015
P_{4-2}	-0.008	0.008	0.000	0.000	0.002
P_{4-1}	-0.032	0.032	-0.000	-0.000	0.007
P_{40}	-0.049	0.087	-0.021	-0.022	0.009
P_{41}	-0.009	0.009	0.000	0.000	0.002
P_{42}	-0.049	0.116	-0.017	-0.018	0.010
P_{43}	-0.010	0.010	0.000	0.000	0.002
P_{44}	-0.015	0.117	-0.004	-0.003	0.007

Figure S2.46 Ridgeplots for P_{lm} parameters (top) and statistical data for κ, κ', P_{val}, and P_{lm} parameters (bottom) for planar nitrogen atoms with two first neighbors (the 2p-N subgroup) in the Z (x1,x2) Y x1 R LCS type. The grey rectangle shows parts of P_{lm} approximated as zero with threshold 0.008.

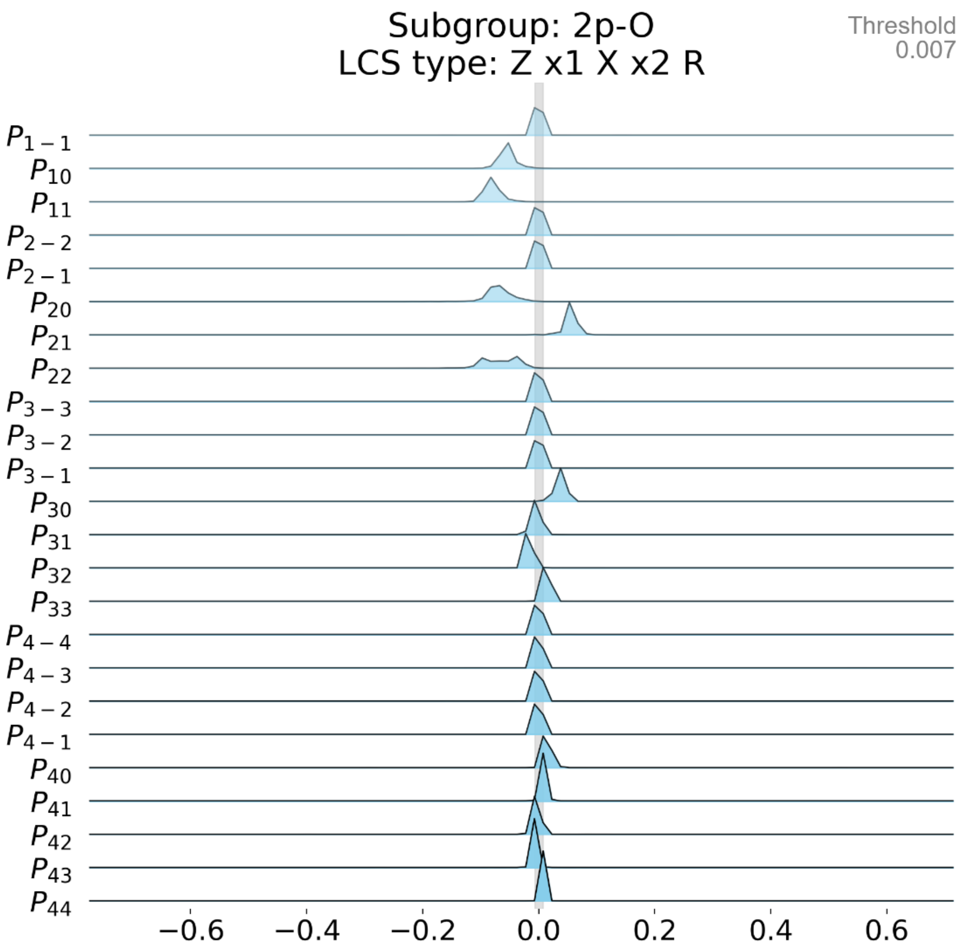

	min	max	median	mean	ssd
κ	0.9622	1.0052	0.9863	0.9863	0.0028
κ'	0.8887	1.7113	1.1723	1.1711	0.0430
P_{val}	6.008	7.284	6.216	6.216	0.054
P_{1-1}	-0.150	0.150	0.000	0.000	0.004
P_{10}	-0.237	0.251	-0.055	-0.054	0.015
P_{11}	-0.134	0.155	-0.081	-0.080	0.014
P_{2-2}	-0.210	0.255	0.000	-0.000	0.006
P_{2-1}	-0.174	0.097	0.000	0.000	0.005
P_{20}	-0.781	0.250	-0.070	-0.068	0.025
P_{21}	-0.317	0.327	0.057	0.056	0.014
P_{22}	-0.718	0.023	-0.062	-0.065	0.030
P_{3-3}	-0.093	0.100	0.000	-0.000	0.003
P_{3-2}	-0.056	0.056	0.000	0.000	0.002
P_{3-1}	-0.087	0.114	0.000	0.000	0.003
P_{30}	-0.138	0.064	0.038	0.037	0.009
P_{31}	-0.055	0.039	-0.005	-0.005	0.007
P_{32}	-0.089	0.096	-0.017	-0.017	0.006
P_{33}	-0.128	0.076	0.013	0.013	0.005
P_{4-4}	-0.408	0.055	0.000	-0.000	0.006
P_{4-3}	-0.105	0.554	-0.000	0.000	0.008
P_{4-2}	-0.156	0.108	0.000	0.000	0.003
P_{4-1}	-0.088	0.721	0.000	0.000	0.011
P_{40}	-0.292	0.523	0.014	0.014	0.010
P_{41}	-0.117	0.475	0.009	0.009	0.008
P_{42}	-0.391	0.110	-0.002	-0.002	0.007
P_{43}	-0.278	0.068	-0.008	-0.007	0.005
P_{44}	-0.053	0.072	0.006	0.006	0.003

Figure S2.47 Ridgeplots for P_{lm} parameters (top) and statistical data for κ, κ', P_{val}, and P_{lm} parameters (bottom) for planar oxygen atoms with two first neighbors (the 2p-O subgroup) in the Z x1 X x2 R LCS type. The grey rectangle shows parts of P_{lm} approximated as zero with threshold 0.007.

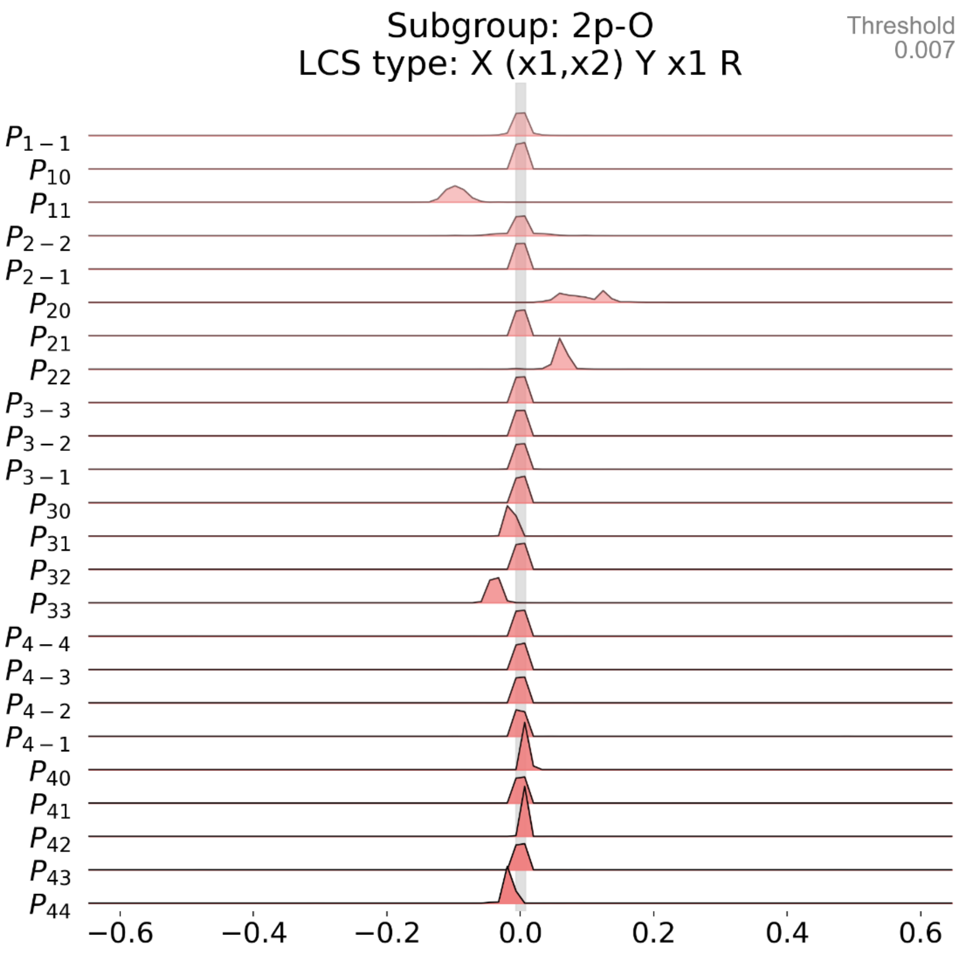

	min	max	median	mean	ssd
κ	0.9622	1.0052	0.9863	0.9863	0.0028
κ'	0.8887	1.7113	1.1723	1.1711	0.0430
P_{val}	6.008	7.284	6.216	6.216	0.054
P_{1-1}	-0.283	0.283	0.000	0.000	0.012
P_{10}	-0.150	0.150	0.000	0.000	0.004
P_{11}	-0.154	0.014	-0.097	-0.097	0.016
P_{2-2}	-0.653	0.653	0.000	0.000	0.024
P_{2-1}	-0.052	0.066	0.000	-0.000	0.003
P_{20}	0.020	0.526	0.090	0.093	0.032
P_{21}	-0.269	0.269	0.000	0.000	0.007
P_{22}	-0.062	0.176	0.062	0.061	0.011
P_{3-3}	-0.043	0.043	0.000	0.000	0.004
P_{3-2}	-0.128	0.040	0.000	-0.000	0.004
P_{3-1}	-0.134	0.134	0.000	0.000	0.006
P_{30}	-0.119	0.119	0.000	0.000	0.003
P_{31}	-0.084	0.065	-0.014	-0.014	0.005
P_{32}	-0.045	0.045	0.000	0.000	0.002
P_{33}	-0.062	0.075	-0.039	-0.038	0.008
P_{4-4}	-0.485	0.485	0.000	0.000	0.010
P_{4-3}	-0.113	0.038	0.000	-0.000	0.003
P_{4-2}	-0.109	0.109	0.000	0.000	0.004
P_{4-1}	-0.104	0.490	-0.000	0.000	0.010
P_{40}	-0.081	0.063	0.009	0.009	0.004
P_{41}	-0.244	0.244	0.000	0.000	0.005
P_{42}	-0.042	0.095	0.004	0.004	0.003
P_{43}	-0.473	0.473	0.000	0.000	0.010
P_{44}	-0.307	0.088	-0.015	-0.016	0.008

Figure S2.48 Ridgeplots for P_{lm} parameters (top) and statistical data for κ, κ', P_{val}, and P_{lm} parameters (bottom) for planar oxygen atoms with two first neighbors (the 2p-O subgroup) in the X (x1,x2) Y x1 R LCS type. The grey rectangle shows parts of P_{lm} approximated as zero with threshold 0.007.

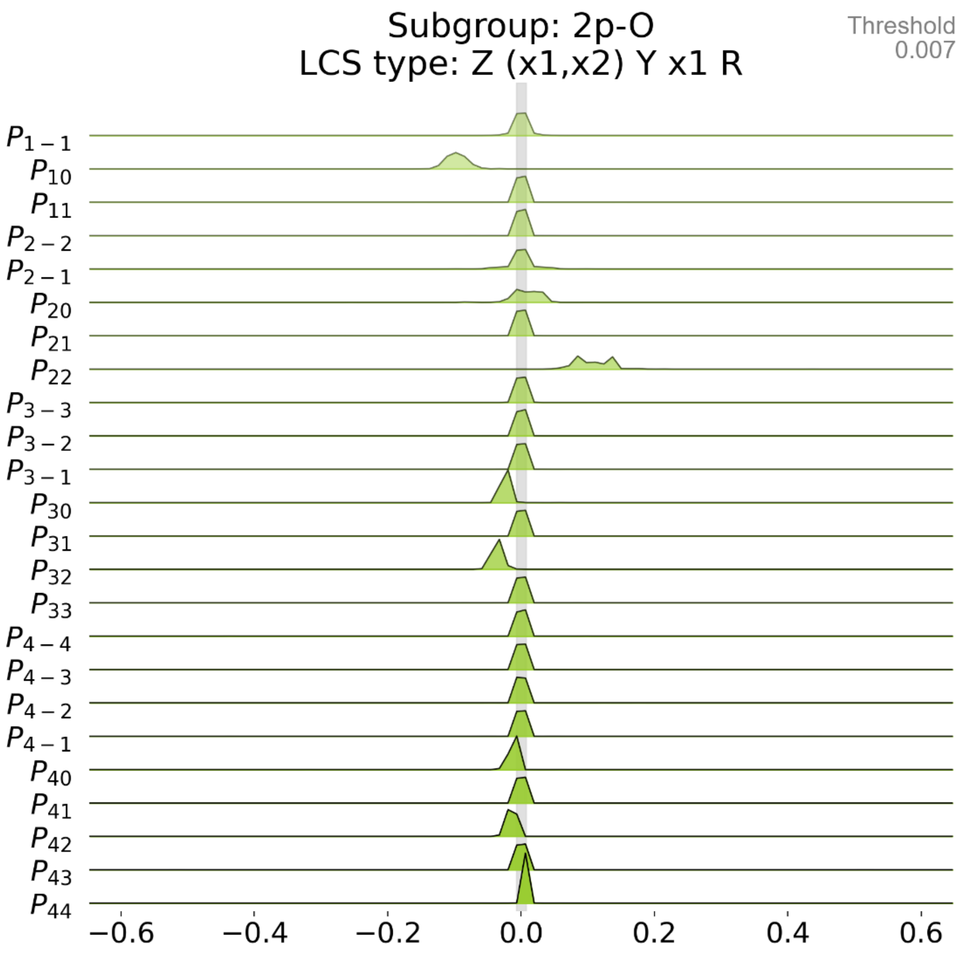

	min	max	median	mean	ssd
κ	0.9622	1.0052	0.9863	0.9863	0.0028
κ'	0.8887	1.7113	1.1723	1.1711	0.0430
P_{val}	6.008	7.284	6.216	6.216	0.054
P_{1-1}	-0.283	0.283	0.000	0.000	0.012
P_{10}	-0.154	0.014	-0.097	-0.097	0.016
P_{11}	-0.150	0.150	0.000	0.000	0.004
P_{2-2}	-0.066	0.052	0.000	0.000	0.003
P_{2-1}	-0.653	0.653	0.000	0.000	0.024
P_{20}	-0.258	0.115	0.011	0.008	0.020
P_{21}	-0.269	0.269	0.000	0.000	0.007
P_{22}	0.038	0.438	0.106	0.108	0.026
P_{3-3}	-0.115	0.115	0.000	0.000	0.006
P_{3-2}	-0.040	0.128	0.000	0.000	0.004
P_{3-1}	-0.076	0.076	0.000	0.000	0.004
P_{30}	-0.044	0.062	-0.024	-0.024	0.005
P_{31}	-0.068	0.068	0.000	0.000	0.002
P_{32}	-0.063	0.082	-0.036	-0.036	0.008
P_{33}	-0.086	0.086	0.000	0.000	0.003
P_{4-4}	-0.432	0.097	0.000	-0.000	0.009
P_{4-3}	-0.283	0.283	0.000	0.000	0.007
P_{4-2}	-0.041	0.178	-0.000	0.000	0.005
P_{4-1}	-0.441	0.441	0.000	0.000	0.009
P_{40}	-0.316	0.096	-0.012	-0.013	0.008
P_{41}	-0.129	0.129	0.000	0.000	0.003
P_{42}	-0.153	0.072	-0.014	-0.014	0.006
P_{43}	-0.517	0.517	0.000	0.000	0.011
P_{44}	-0.064	0.105	0.007	0.006	0.003

Figure S2.49 Ridgeplots for P_{lm} parameters (top) and statistical data for κ, κ', P_{val}, and P_{lm} parameters (bottom) for planar oxygen atoms with two first neighbors (the 2p-O subgroup) in the Z (x1,x2) Y x1 R LCS type. The grey rectangle shows parts of P_{lm} approximated as zero with threshold 0.007.

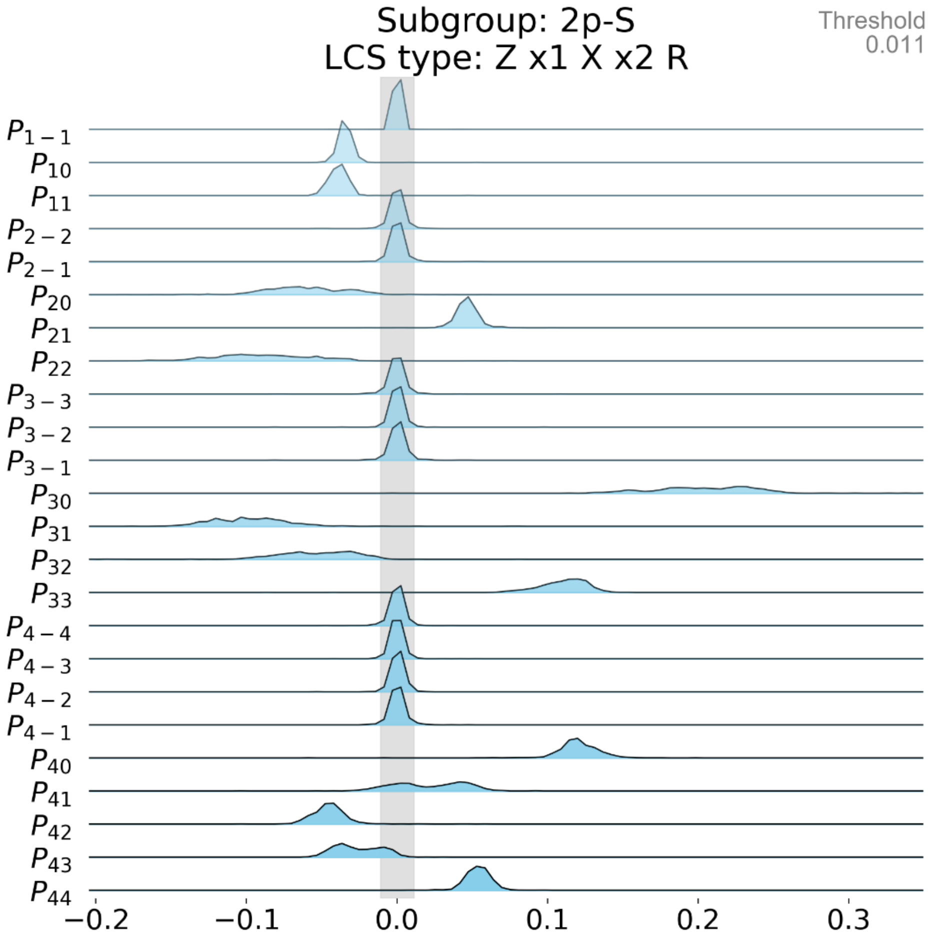

	min	max	median	mean	ssd
κ	0.9765	0.9982	0.9831	0.9833	0.0024
κ'	0.9526	1.1409	1.0675	1.0678	0.0298
P_{val}	5.970	6.457	6.311	6.303	0.068
P_{1-1}	-0.042	0.042	0.000	0.000	0.003
P_{10}	-0.051	0.051	-0.034	-0.034	0.005
P_{11}	-0.055	0.046	-0.039	-0.039	0.006
P_{2-2}	-0.054	0.026	-0.000	-0.000	0.005
P_{2-1}	-0.022	0.055	0.000	0.000	0.005
P_{20}	-0.179	0.060	-0.062	-0.060	0.027
P_{21}	-0.022	0.103	0.046	0.046	0.008
P_{22}	-0.189	0.078	-0.090	-0.088	0.034
P_{3-3}	-0.080	0.106	-0.000	-0.000	0.007
P_{3-2}	-0.083	0.096	0.000	0.000	0.006
P_{3-1}	-0.081	0.047	0.000	0.000	0.006
P_{30}	-0.001	0.352	0.205	0.203	0.033
P_{31}	-0.177	0.080	-0.100	-0.098	0.025
P_{32}	-0.207	0.084	-0.051	-0.053	0.026
P_{33}	-0.086	0.155	0.113	0.110	0.017
P_{4-4}	-0.045	0.038	0.000	-0.000	0.005
P_{4-3}	-0.044	0.054	-0.000	-0.000	0.005
P_{4-2}	-0.053	0.018	0.000	0.000	0.004
P_{4-1}	-0.020	0.046	0.000	0.000	0.005
P_{40}	-0.059	0.196	0.120	0.120	0.015
P_{41}	-0.105	0.091	0.024	0.022	0.024
P_{42}	-0.102	0.056	-0.045	-0.045	0.012
P_{43}	-0.060	0.030	-0.028	-0.026	0.015
P_{44}	0.007	0.096	0.054	0.054	0.008

Figure S2.50 Ridgeplots for P_{lm} parameters (top) and statistical data for κ, κ', P_{val}, and P_{lm} parameters (bottom) for planar sulfur atoms with two first neighbors (the 2p-S subgroup) in the Z x1 X x2 R LCS type. The grey rectangle shows parts of P_{lm} approximated as zero with threshold 0.011.

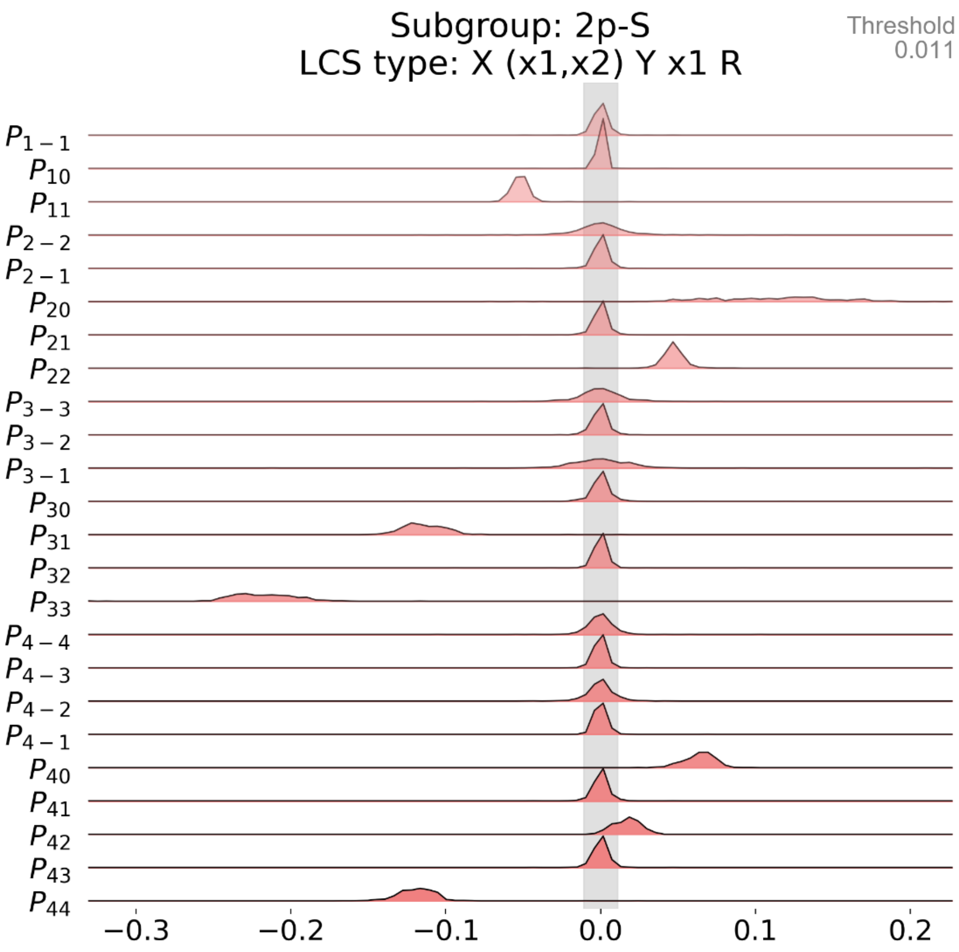

	min	max	median	mean	ssd
κ	0.9765	0.9982	0.9831	0.9833	0.0024
κ'	0.9526	1.1409	1.0675	1.0678	0.0298
P_{val}	5.970	6.457	6.311	6.303	0.068
P_{1-1}	-0.048	0.048	0.000	-0.000	0.005
P_{10}	-0.042	0.042	0.000	-0.000	0.003
P_{11}	-0.067	0.021	-0.052	-0.052	0.006
P_{2-2}	-0.119	0.119	0.000	-0.000	0.017
P_{2-1}	-0.050	0.022	0.000	-0.000	0.005
P_{20}	-0.072	0.230	0.114	0.110	0.041
P_{21}	-0.051	0.051	0.000	0.000	0.005
P_{22}	-0.011	0.087	0.047	0.047	0.007
P_{3-3}	-0.076	0.076	0.000	-0.000	0.013
P_{3-2}	-0.027	0.023	-0.000	-0.000	0.005
P_{3-1}	-0.210	0.210	0.000	-0.000	0.020
P_{30}	-0.123	0.123	0.000	-0.000	0.009
P_{31}	-0.149	0.012	-0.115	-0.114	0.014
P_{32}	-0.092	0.092	0.000	0.000	0.006
P_{33}	-0.333	-0.114	-0.218	-0.218	0.021
P_{4-4}	-0.103	0.103	0.000	0.000	0.009
P_{4-3}	-0.018	0.028	-0.000	-0.000	0.004
P_{4-2}	-0.048	0.048	0.000	0.000	0.009
P_{4-1}	-0.013	0.071	-0.000	0.000	0.005
P_{40}	0.026	0.099	0.065	0.064	0.010
P_{41}	-0.046	0.046	0.000	0.000	0.005
P_{42}	-0.003	0.037	0.018	0.017	0.008
P_{43}	-0.045	0.045	0.000	0.000	0.005
P_{44}	-0.170	0.025	-0.118	-0.119	0.014

Figure S2.51 Ridgeplots for P_{lm} parameters (top) and statistical data for κ, κ', P_{val}, and P_{lm} parameters (bottom) for planar sulfur atoms with two first neighbors (the 2p-S subgroup) in the X (x1,x2) Y x1 R LCS type. The grey rectangle shows parts of P_{lm} approximated as zero with threshold 0.011.

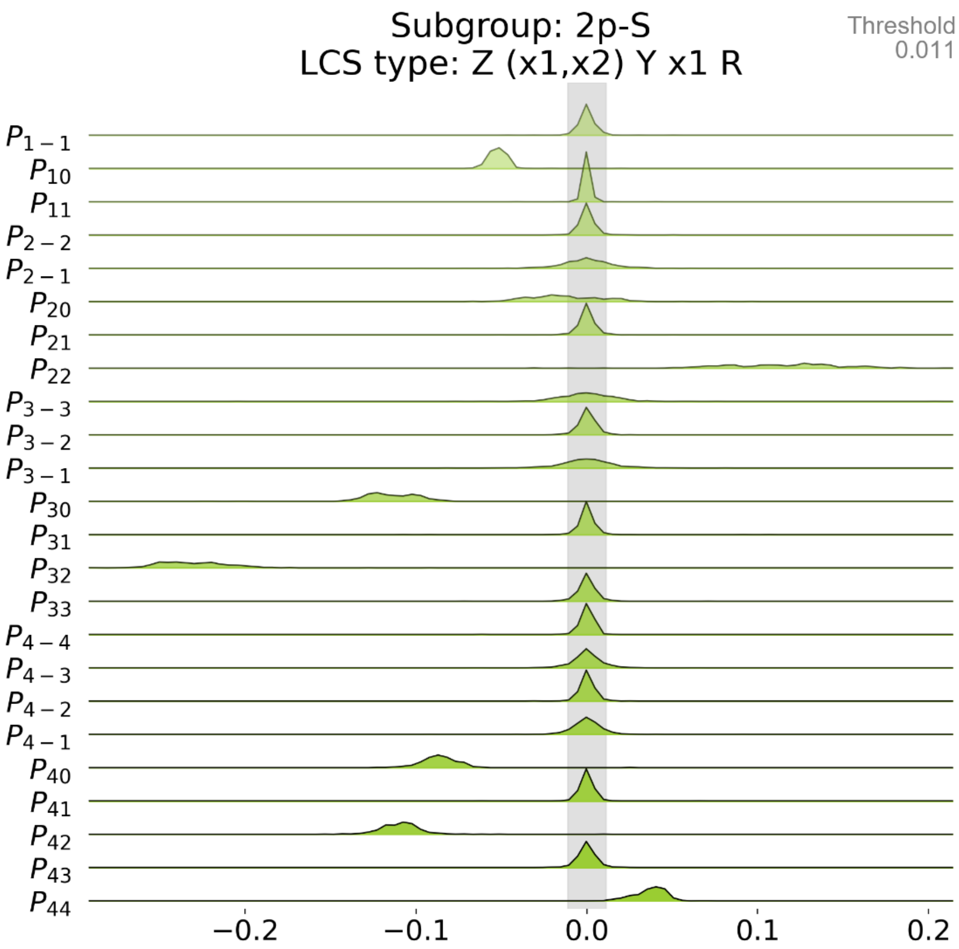

	min	max	median	mean	ssd
κ	0.9765	0.9982	0.9831	0.9833	0.0024
κ'	0.9526	1.1409	1.0675	1.0678	0.0298
P_{val}	5.970	6.457	6.311	6.303	0.068
P_{1-1}	-0.048	0.048	0.000	-0.000	0.005
P_{10}	-0.067	0.021	-0.052	-0.052	0.006
P_{11}	-0.042	0.042	0.000	0.000	0.003
P_{2-2}	-0.022	0.050	-0.000	0.000	0.005
P_{2-1}	-0.119	0.119	0.000	-0.000	0.017
P_{20}	-0.067	0.098	-0.015	-0.012	0.021
P_{21}	-0.051	0.051	0.000	-0.000	0.005
P_{22}	-0.033	0.216	0.118	0.115	0.034
P_{3-3}	-0.204	0.204	0.000	0.000	0.018
P_{3-2}	-0.023	0.027	0.000	0.000	0.005
P_{3-1}	-0.080	0.080	0.000	0.000	0.015
P_{30}	-0.227	-0.076	-0.115	-0.114	0.016
P_{31}	-0.107	0.107	0.000	0.000	0.008
P_{32}	-0.294	-0.091	-0.232	-0.230	0.021
P_{33}	-0.071	0.071	0.000	0.000	0.006
P_{4-4}	-0.062	0.013	0.000	0.000	0.005
P_{4-3}	-0.065	0.065	0.000	0.000	0.009
P_{4-2}	-0.029	0.026	0.000	0.000	0.004
P_{4-1}	-0.092	0.092	0.000	0.000	0.009
P_{40}	-0.125	0.024	-0.087	-0.086	0.012
P_{41}	-0.064	0.064	0.000	-0.000	0.005
P_{42}	-0.154	0.008	-0.108	-0.109	0.012
P_{43}	-0.026	0.026	0.000	-0.000	0.005
P_{44}	0.003	0.070	0.038	0.037	0.009

Figure S2.52 Ridgeplots for P_{lm} parameters (top) and statistical data for κ, κ', P_{val}, and P_{lm} parameters (bottom) for planar sulfur atoms with two first neighbors (the 2p-S subgroup) in the Z (x1,x2) Y x1 R LCS type. The grey rectangle shows parts of P_{lm} approximated as zero with threshold 0.011.

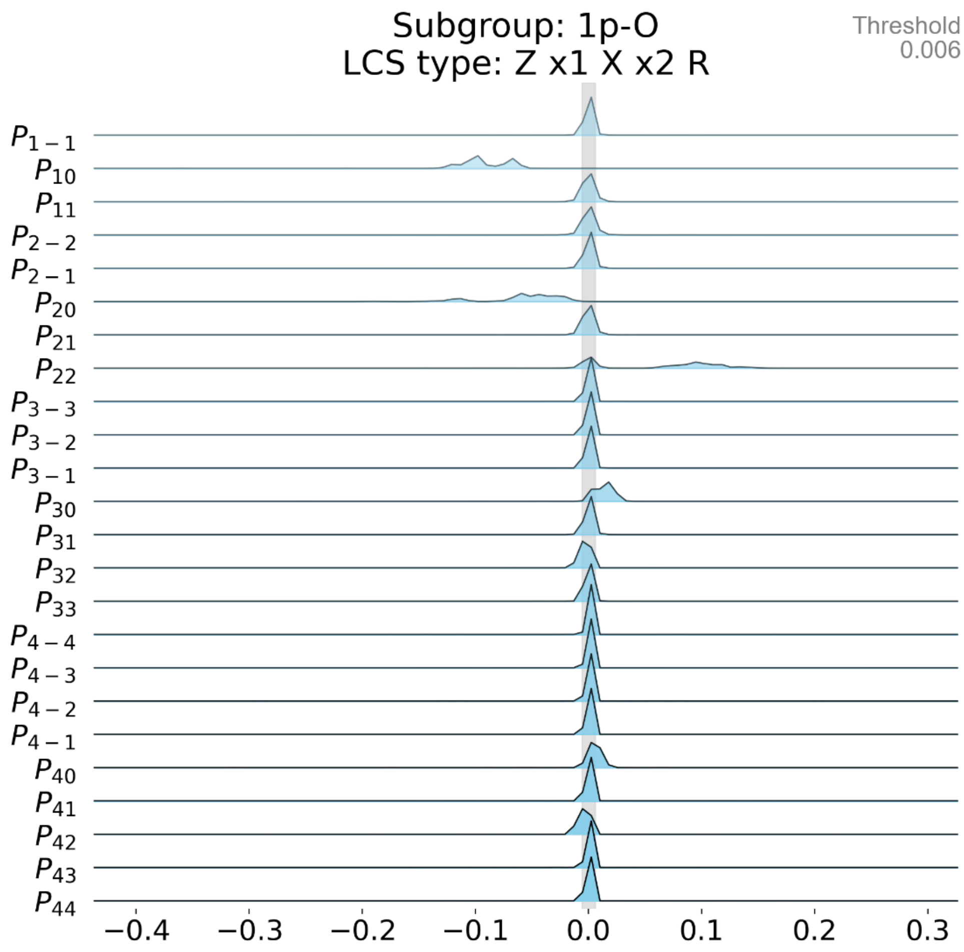

	min	max	median	mean	ssd
κ	0.9512	1.0063	0.9851	0.9845	0.0051
κ'	0.5161	1.7424	1.1657	1.1950	0.0767
P_{val}	5.782	6.581	6.181	6.197	0.102
P_{1-1}	-0.253	0.324	0.000	-0.000	0.006
P_{10}	-0.441	-0.047	-0.097	-0.091	0.021
P_{11}	-0.311	0.210	-0.000	-0.000	0.007
P_{2-2}	-0.167	0.145	0.000	0.000	0.008
P_{2-1}	-0.228	0.330	0.000	0.000	0.007
P_{20}	-0.268	0.026	-0.049	-0.057	0.034
P_{21}	-0.298	0.240	-0.000	-0.000	0.007
P_{22}	-0.145	0.176	0.081	0.061	0.051
P_{3-3}	-0.038	0.034	-0.000	-0.000	0.002
P_{3-2}	-0.206	0.156	-0.000	-0.000	0.004
P_{3-1}	-0.075	0.114	-0.000	-0.000	0.003
P_{30}	-0.249	0.039	0.015	0.014	0.010
P_{31}	-0.085	0.107	0.000	0.000	0.003
P_{32}	-0.203	0.134	-0.003	-0.003	0.005
P_{33}	-0.044	0.040	-0.000	-0.000	0.003
P_{4-4}	-0.176	0.167	-0.000	-0.000	0.004
P_{4-3}	-0.142	0.276	-0.000	-0.000	0.006
P_{4-2}	-0.293	0.199	0.000	-0.000	0.005
P_{4-1}	-0.161	0.161	-0.000	0.000	0.005
P_{40}	-0.310	0.065	0.006	0.005	0.009
P_{41}	-0.154	0.165	0.000	0.000	0.004
P_{42}	-0.284	0.217	-0.006	-0.005	0.007
P_{43}	-0.150	0.106	0.000	-0.000	0.004
P_{44}	-0.120	0.174	-0.000	-0.000	0.004

Figure S2.53 Ridgeplots for P_{lm} parameters (top) and statistical data for κ, κ', P_{val}, and P_{lm} parameters (bottom) for planar oxygen atoms with one first neighbor (the 1p-O subgroup) in the Z x1 X x2 R LCS type. The grey rectangle shows parts of P_{lm} approximated as zero with threshold 0.006.

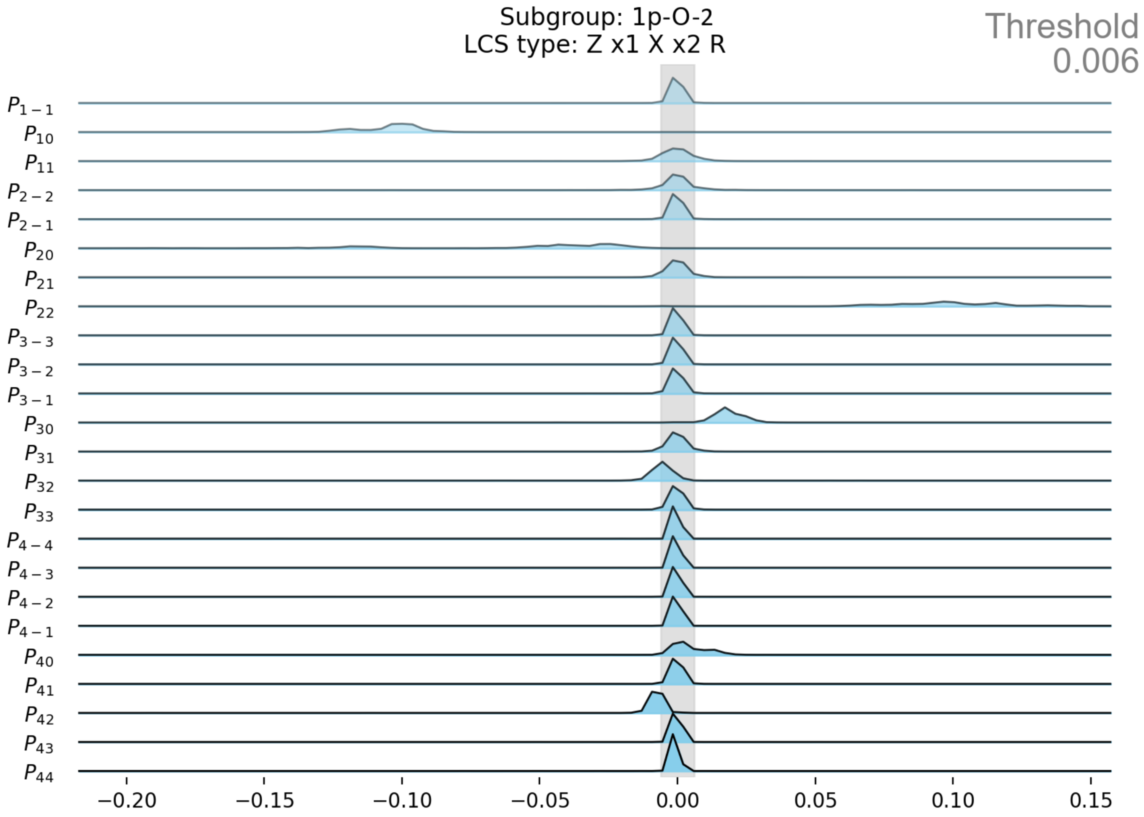

	min	max	median	mean	ssd
κ	0.9512	1.0007	0.9876	0.9867	0.0038
κ'	1.0566	1.7424	1.1499	1.1502	0.0281
P_{val}	5.959	6.487	6.140	6.153	0.071
P_{1-1}	-0.042	0.042	0.000	0.000	0.002
P_{10}	-0.138	-0.074	-0.102	-0.104	0.010
P_{11}	-0.049	0.049	0.000	-0.000	0.006
P_{2-2}	-0.125	0.145	0.000	0.000	0.007
P_{2-1}	-0.043	0.043	0.000	0.000	0.002
P_{20}	-0.219	0.002	-0.041	-0.055	0.041
P_{21}	-0.082	0.082	0.000	-0.000	0.004
P_{22}	-0.079	0.159	0.098	0.098	0.023
P_{3-3}	-0.023	0.022	0.000	0.000	0.002
P_{3-2}	-0.042	0.050	0.000	-0.000	0.002
P_{3-1}	-0.032	0.032	0.000	-0.000	0.002
P_{30}	-0.045	0.039	0.018	0.018	0.006
P_{31}	-0.054	0.054	0.000	0.000	0.004
P_{32}	-0.065	0.066	-0.006	-0.006	0.004
P_{33}	-0.040	0.040	0.000	0.000	0.003
P_{4-4}	-0.147	0.084	0.000	-0.000	0.003
P_{4-3}	-0.055	0.054	0.000	0.000	0.002
P_{4-2}	-0.129	0.061	0.000	-0.000	0.003
P_{4-1}	-0.115	0.114	0.000	0.000	0.003
P_{40}	-0.174	0.032	0.003	0.004	0.007
P_{41}	-0.043	0.043	0.000	0.000	0.002
P_{42}	-0.191	0.052	-0.008	-0.008	0.005
P_{43}	-0.051	0.052	0.000	-0.000	0.002
P_{44}	-0.077	0.011	-0.001	-0.001	0.003

Figure S2.54 Ridgeplots for P_{lm} parameters (top) and statistical data for κ, κ', P_{val}, and P_{lm} parameters (bottom) for planar oxygen atoms with one first neighbor and two second neighbors (the 1p-O-2 subgroup) in the Z x1 X x2 R LCS type. The grey rectangle shows parts of P_{lm} approximated as zero with threshold 0.006.

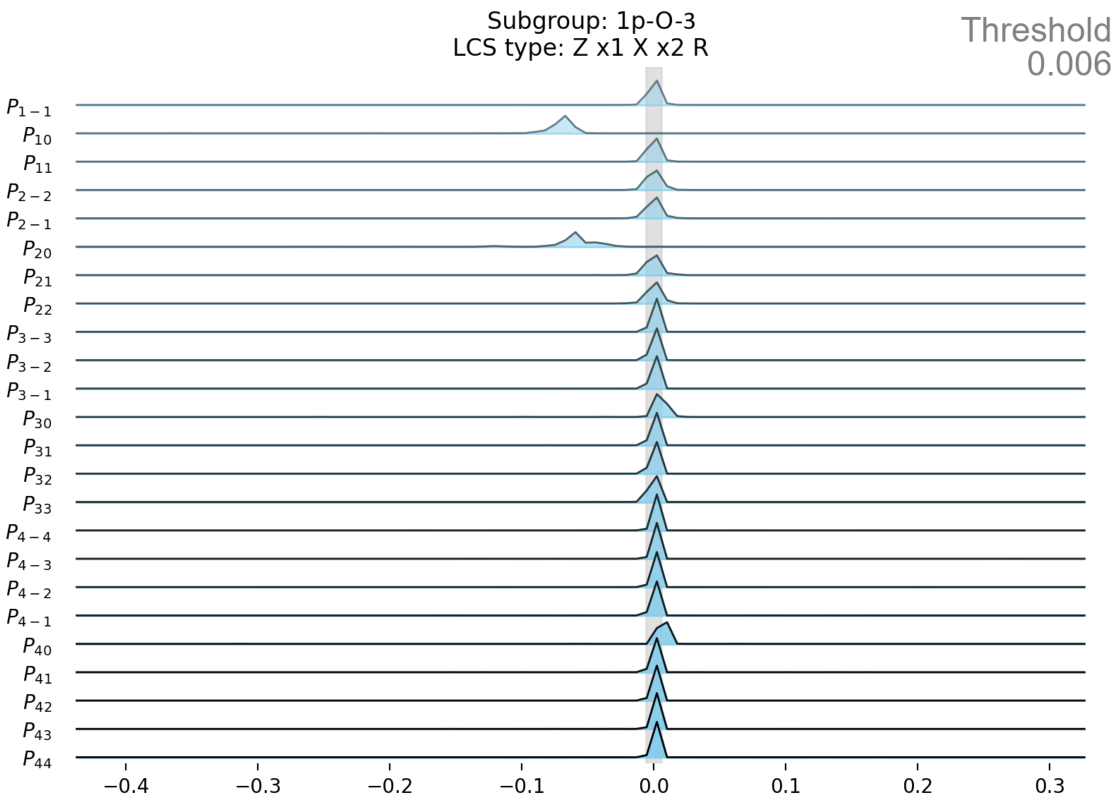

	min	max	median	mean	ssd
κ	0.9629	1.0006	0.9823	0.9810	0.0051
κ'	0.5161	1.4225	1.2926	1.2682	0.0748
P_{val}	5.930	6.581	6.256	6.271	0.102
P_{1-1}	-0.253	0.324	0.000	-0.000	0.009
P_{10}	-0.441	-0.047	-0.069	-0.070	0.017
P_{11}	-0.311	0.210	0.000	-0.000	0.008
P_{2-2}	-0.167	0.136	0.000	0.000	0.009
P_{2-1}	-0.228	0.330	0.000	-0.000	0.011
P_{20}	-0.268	0.025	-0.060	-0.060	0.020
P_{21}	-0.298	0.240	0.000	-0.000	0.010
P_{22}	-0.145	0.154	0.000	0.000	0.008
P_{3-3}	-0.038	0.034	0.000	-0.000	0.002
P_{3-2}	-0.206	0.156	0.000	0.000	0.005
P_{3-1}	-0.075	0.114	0.000	-0.000	0.003
P_{30}	-0.249	0.022	0.005	0.006	0.009
P_{31}	-0.085	0.107	0.000	0.000	0.003
P_{32}	-0.203	0.134	0.000	-0.000	0.005
P_{33}	-0.044	0.026	-0.001	-0.001	0.003
P_{4-4}	-0.176	0.167	0.000	0.000	0.005
P_{4-3}	-0.142	0.276	0.000	-0.000	0.010
P_{4-2}	-0.293	0.199	0.000	0.000	0.007
P_{4-1}	-0.161	0.161	0.000	0.000	0.007
P_{40}	-0.310	0.065	0.007	0.007	0.010
P_{41}	-0.154	0.165	0.000	0.000	0.007
P_{42}	-0.284	0.217	0.000	-0.000	0.007
P_{43}	-0.150	0.106	0.000	-0.000	0.006
P_{44}	-0.120	0.174	0.000	-0.000	0.005

Figure S2.55 Ridgeplots for P_{lm} parameters (top) and statistical data for κ, κ', P_{val}, and P_{lm} parameters (bottom) for planar oxygen atoms with one first neighbor and three second neighbors (the 1p-O-3 subgroup) in the Z x1 X x2 R LCS type. The grey rectangle shows parts of P_{lm} approximated as zero with threshold 0.006.

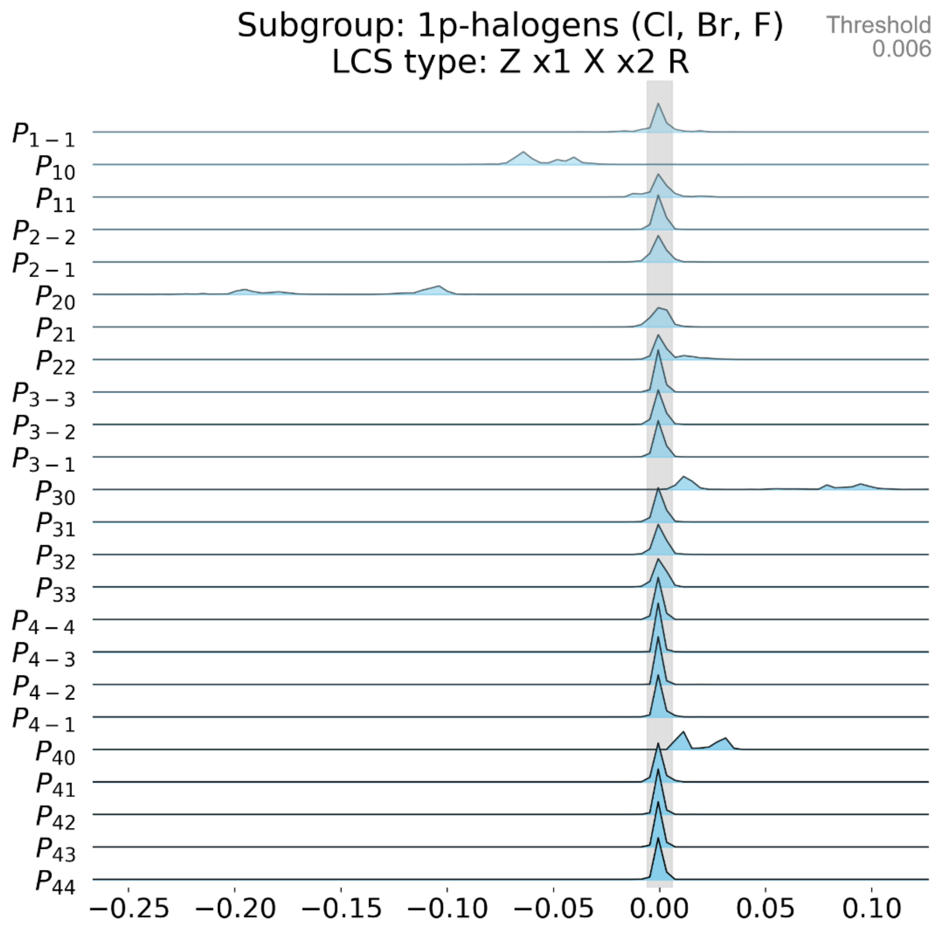

	min	max	median	mean	ssd
κ	0.9818	0.9970	0.9920	0.9918	0.0022
κ'	0.8691	1.2899	0.9583	1.0275	0.1275
P_{val}	6.964	7.571	7.210	7.209	0.050
P_{1-1}	-0.038	0.035	0.000	-0.000	0.006
P_{10}	-0.115	0.043	-0.061	-0.056	0.014
P_{11}	-0.030	0.042	0.000	-0.000	0.006
P_{2-2}	-0.011	0.011	0.000	0.000	0.002
P_{2-1}	-0.022	0.021	0.000	-0.000	0.004
P_{20}	-0.269	-0.088	-0.124	-0.148	0.044
P_{21}	-0.027	0.016	0.001	0.000	0.004
P_{22}	-0.012	0.034	0.001	0.003	0.007
P_{3-3}	-0.007	0.008	0.000	-0.000	0.001
P_{3-2}	-0.008	0.011	0.000	0.000	0.002
P_{3-1}	-0.012	0.011	0.000	-0.000	0.002
P_{30}	0.002	0.128	0.017	0.048	0.039
P_{31}	-0.013	0.013	0.000	-0.000	0.002
P_{32}	-0.009	0.018	0.000	0.000	0.003
P_{33}	-0.011	0.009	0.000	0.000	0.003
P_{4-4}	-0.005	0.005	0.000	0.000	0.001
P_{4-3}	-0.009	0.008	0.000	0.000	0.001
P_{4-2}	-0.005	0.020	0.000	0.000	0.001
P_{4-1}	-0.019	0.019	-0.000	-0.000	0.002
P_{40}	0.006	0.040	0.012	0.019	0.010
P_{41}	-0.016	0.016	-0.000	-0.000	0.002
P_{42}	-0.011	0.013	-0.000	-0.000	0.001
P_{43}	-0.006	0.006	0.000	0.000	0.001
P_{44}	-0.011	0.005	-0.000	-0.000	0.002

Figure S2.56 Ridgeplots for P_{lm} parameters (top) and statistical data for κ, κ', P_{val}, and P_{lm} parameters (bottom) for planar chlorine, bromine, and fluorine atoms with one first neighbor (the 1p-halogens subgroup) in the Z x1 X x2 R LCS type. The grey rectangle shows parts of P_{lm} approximated as zero with threshold 0.006.

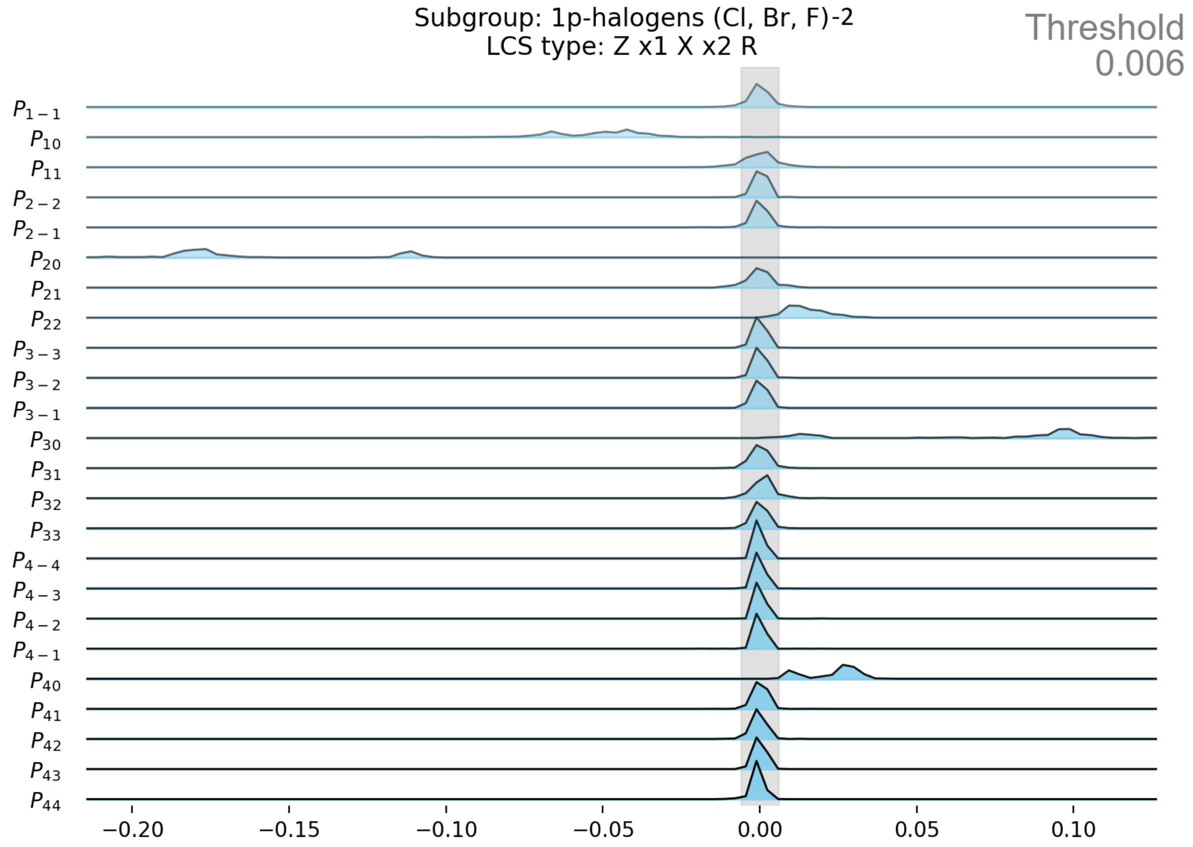

	min	max	median	mean	ssd
κ	0.9818	0.9970	0.9920	0.9918	0.0022
κ'	0.8691	1.2899	0.9583	1.0275	0.1275
P_{val}	6.964	7.571	7.210	7.209	0.050
P_{1-1}	-0.014	0.014	0.000	-0.000	0.004
P_{10}	-0.103	0.007	-0.049	-0.051	0.015
P_{11}	-0.023	0.023	0.000	0.000	0.006
P_{2-2}	-0.010	0.009	0.000	0.000	0.002
P_{2-1}	-0.021	0.021	0.000	0.000	0.003
P_{20}	-0.216	-0.103	-0.177	-0.163	0.031
P_{21}	-0.013	0.013	0.000	0.000	0.004
P_{22}	0.002	0.034	0.014	0.015	0.006
P_{3-3}	-0.007	0.008	0.000	-0.000	0.002
P_{3-2}	-0.007	0.011	0.000	0.000	0.002
P_{3-1}	-0.007	0.007	0.000	0.000	0.002
P_{30}	0.002	0.128	0.094	0.073	0.037
P_{31}	-0.013	0.013	0.000	-0.000	0.003
P_{32}	-0.009	0.018	0.001	0.001	0.004
P_{33}	-0.009	0.009	0.000	0.000	0.002
P_{4-4}	-0.003	0.003	0.000	0.000	0.001
P_{4-3}	-0.009	0.008	0.000	0.000	0.001
P_{4-2}	-0.003	0.020	0.000	0.000	0.002
P_{4-1}	-0.019	0.019	0.000	0.000	0.002
P_{40}	0.006	0.040	0.026	0.023	0.008
P_{41}	-0.016	0.016	0.000	0.000	0.002
P_{42}	-0.011	0.013	0.000	-0.000	0.002
P_{43}	-0.006	0.006	0.000	-0.000	0.002
P_{44}	-0.011	0.005	0.000	-0.001	0.002

Figure S2.57 Ridgeplots for P_{lm} parameters (top) and statistical data for κ, κ', P_{val}, and P_{lm} parameters (bottom) for planar chlorine, bromine, and fluorine atoms with one first neighbor (the 1p-halogens subgroup) in the Z x1 X x2 R LCS type. The grey rectangle shows parts of P_{lm} approximated as zero with threshold 0.006.

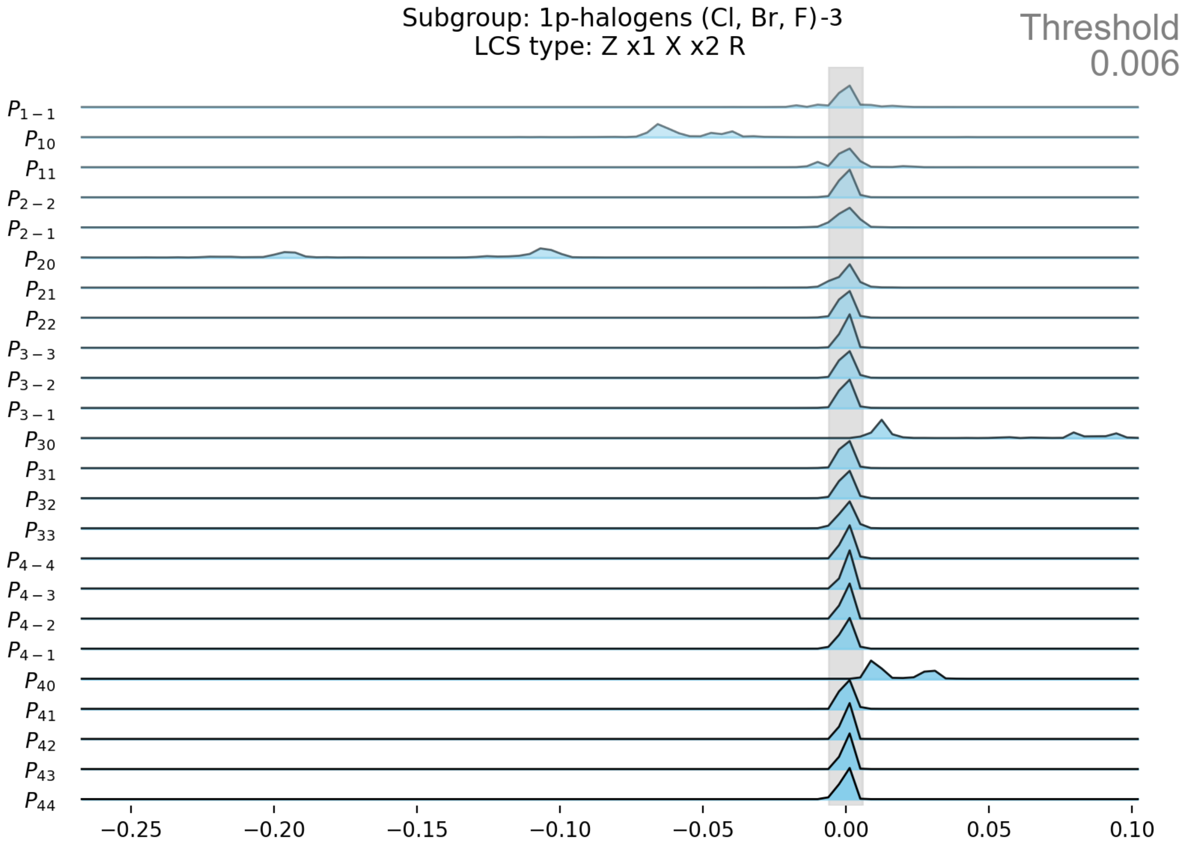

	min	max	median	mean	ssd
κ	0.9815	0.9968	0.9928	0.9922	0.0023
κ'	0.8523	1.2966	1.1999	1.1293	0.1399
P_{val}	6.935	7.517	7.168	7.181	0.052
P_{1-1}	-0.038	0.035	0.000	-0.000	0.006
P_{10}	-0.115	0.043	-0.062	-0.057	0.013
P_{11}	-0.030	0.042	0.000	-0.000	0.007
P_{2-2}	-0.011	0.011	0.000	-0.000	0.002
P_{2-1}	-0.022	0.021	0.000	0.000	0.004
P_{20}	-0.269	-0.088	-0.113	-0.143	0.046
P_{21}	-0.027	0.016	0.001	0.000	0.004
P_{22}	-0.012	0.009	0.000	0.000	0.002
P_{3-3}	-0.006	0.008	0.000	-0.000	0.001
P_{3-2}	-0.008	0.008	0.000	-0.000	0.002
P_{3-1}	-0.012	0.011	0.000	0.000	0.002
P_{30}	0.005	0.104	0.014	0.040	0.035
P_{31}	-0.011	0.011	0.000	0.000	0.002
P_{32}	-0.008	0.008	0.000	-0.000	0.002
P_{33}	-0.011	0.008	0.001	0.000	0.003
P_{4-4}	-0.005	0.005	0.000	0.000	0.002
P_{4-3}	-0.003	0.007	0.000	0.000	0.001
P_{4-2}	-0.005	0.005	0.000	-0.000	0.001
P_{4-1}	-0.010	0.007	0.000	0.000	0.002
P_{40}	0.006	0.037	0.011	0.017	0.009
P_{41}	-0.009	0.009	0.000	0.000	0.002
P_{42}	-0.005	0.004	0.000	0.000	0.001
P_{43}	-0.005	0.005	0.000	0.000	0.001
P_{44}	-0.006	0.005	0.000	0.000	0.002

Figure S2.58 Ridgeplots for P_{lm} parameters (top) and statistical data for κ, κ', P_{val}, and P_{lm} parameters (bottom) for planar chlorine, bromine, and fluorine atoms with one first neighbor (the 1p-halogens subgroup) in the Z x1 X x2 R LCS type. The grey rectangle shows parts of P_{lm} approximated as zero with threshold 0.006.
