# Supplementary material for: From atoms to a data bank: optimizing transferability of electron-density symmetry
Source: Acta Crystallogr A Found Adv. 2026 Jun 12;82(Pt 4):256–75. doi: 10.1107/S2053273326004651 (PMC13325189; doi:10.1107/S2053273326004651)
Supplement: Supplementary file 3 [file a-82-00256-sup3.pdf]

FOUNDATIONS
ADVANCES

Volume 82 (2026)

Supporting information for article:

From atoms to a data bank: optimizing transferability of electron-density symmetry

Paulina Maria Rybicka, Marta Kulik, Vladislav Ignat'ev and Paulina Maria Dominiak

From atoms to a data bank: optimizing transferability of electron density symmetry

Authors

Paulina Maria Rybicka[a], Marta Kulik[a], Vladislav Ignat'ev[a] and Paulina Maria Dominiak[a]*

[a]University of Warsaw, Faculty of Chemistry, Biological and Chemical Research Centre, Zwirki i Wigury 101, Warsaw, 02-089, Poland

Correspondence email: pdomin@uw.edu.pl

Funding information Narodowe Centrum Nauki (grant No. UMO-2020/39/I/ST4/02904).

Supporting information S3

Note on nomenclature: In the main text, the term “no symmetry” has been replaced with “1” for consistency with standard notation. However, in this Supporting Information, the original phrasing “no symmetry” has been retained for better visibility. Both notations refer to the same concept.

S1. 4n-C

Atoms in individual LCS orientations

Pseudosymmetry point groups were assigned to electron densities of 4n-C atoms expressed in various individual LCS orientations, and distributions of these pseudosymmetries were analyzed within each LCS type independently. For the 4n-C subgroup, the lowest possible pseudosymmetry no was assigned to ca. 45% to 65% of atoms in individual LCS, depending on the LCS type (Figure S3.1a left). The next common pseudosymmetry was m (m⊥x, m⊥y, m⊥z). Pseudosymmetries higher than m were observed for less than one-fourth of all individual LCS orientations, regardless the LCS type. It is worth to note that the $\bar{4}$3m($\bar{4}$‖z) and mm2(2‖x, 2‖z) symmetries cannot be directly assigned to the individual LCS orientations of the Z x1 X x2 R and Z (x1,x2,x3) X x1 R LCS types, the $\bar{4}$3m($\bar{4}$‖z) and 3m(m⊥y) symmetries to the individual LCS orientations of the X (x1,x2) Y x1 R LCS type and the 3m(m⊥y) symmetry to the individual LCS orientations of the Z (x1,x2) X x3 R LCS type.

IMPORTANT: this document contains embedded data - to preserve data integrity, please ensure where possible that the IUCr Word tools (available from http://journals.iucr.org/services/docxtemplate/) are installed when editing this document.

The overall distributions of pseudosymmetries assigned to individual LCS orientation for 4n-C atoms from the ref-SC and ref-NSC datasets exhibited slight differences, with an increase of no pseudosymmetries for ref-NSC (Figure S3.1a left). The assigned pseudosymmetry was different for 102,895 individual LCS orientations (21.78 %) (Table S1.16). The three most common changes of pseudosymmetry between the ref-SC and ref-NSC were: from m(m⊥y) to no (4.20%), no to m(m⊥y) (4.01%), and 3m(m⊥y) to no (2.12%) (Table S1.16). Moreover, for the ref-SC dataset, 45 individual LCS orientations, including only Z x1 X x2 R or Z (x1,x2) X x3 R LCS types, had the cyl symmetry assigned. The existence of cylindrical symmetry of the electron density is not feasible for a non-planar atom with four first neighbors. The cyl pseudosymmetry was no longer present for 4n-C atoms from the ref-NSC dataset.

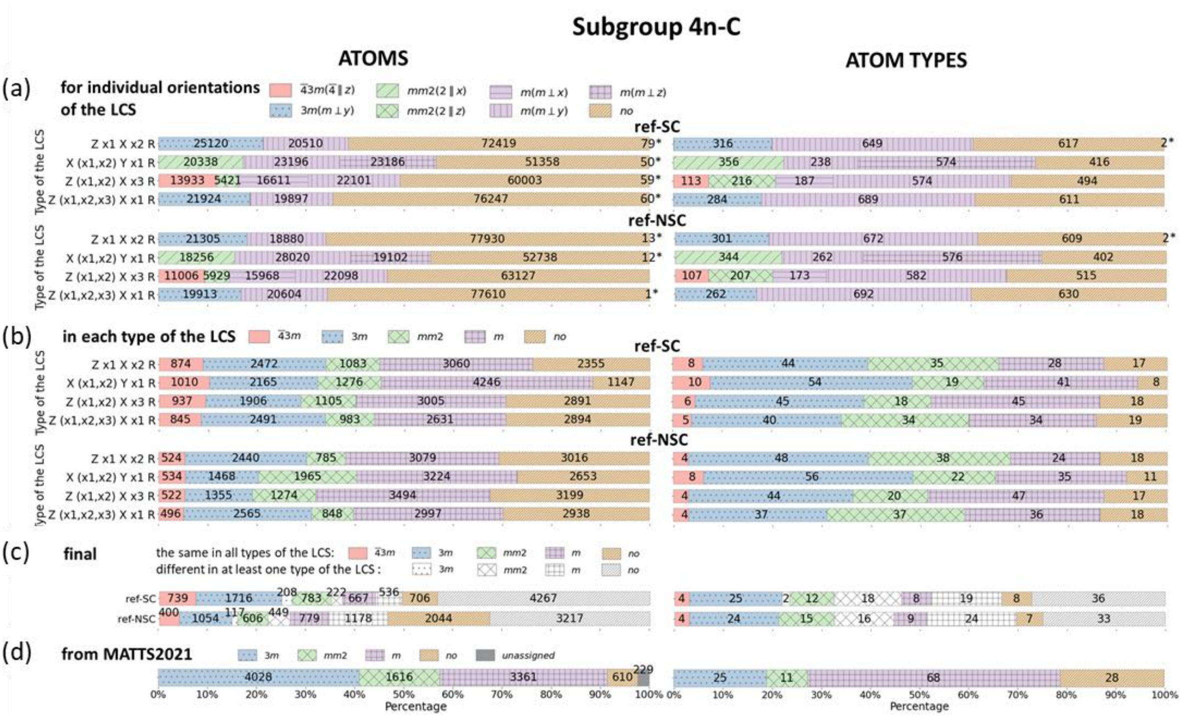

Figure S3.1 Distributions of pseudosymmetries of electron density assigned to individual LCS orientations LCS and grouped by four LCS types (a), to entire LCS type (b), and final pseudosymmetry (c) assigned to non-planar carbon atoms (left) and atom types (right) with four first neighbors (the 4n-C subgroup) on the basis of the P_{lm} values for ref-SC and ref-NSC datasets. Symmetry distributions for atoms and atom types in the MATTS2021 data bank is presented in the panel (d). Number of instances with each pseudosymmetry is shown on the bars. For clarity purposes, categories with a small count of instances were summed up and presented at the end of the bars with a mark *.

Atoms in LCS type

By considering together all individual LCS orientations from the given LCS type, the electron density pseudosymmetries of the 4n-C atoms were assigned from the perspective of the entire LCS type (Figure S3.1b left). Each of the four LCS types allows for the direct or indirect assignment of all pseudosymmetries expected within the 4n group. Comparing to electron densities analyzed independently for each individual LCS orientation, the % of cases with the no pseudosymmetry was considerably lower, since many atoms got assigned higher pseudosymmetry using indirect approach. The m pseudosymmetries dominated in all but one cases. The second most common pseudosymmetry was 3m or no, depending on the LCS type. The mm2 pseudosymmetry was usually second to last, and the $\bar{4}$3m pseudosymmetry was the least common in all LCS types.

In the ref-NSC dataset, the 3m pseudosymmetry was favorable in the Z x1 X x2 R and Z (x1,x2,x3) X x1 R LCS types, whereas the mm2 pseudosymmetry was favorable in the X (x1,x2) Y x1 R LCS type. For the Z (x1,x2) X x3 R LCS type, the distribution of all pseudosymmetries seemed to be the most balanced.

Some discrepancy in the distributions of assigned pseudosymmetries was observed comparing the ref-SC and ref-NSC datasets. For all four LCS types, the percentage of no pseudosymmetries increased and the percentage of $\bar{4}$3m pseudosymmetries decreased after releasing the symmetry constraints. The differences in the overall distribution of 3m and mm2 pseudosymmetries between different LCS types were more pronounced for the ref-NSC than for the ref-SC dataset.

Atoms

In order to assign the final pseudosymmetry to electron density of each atom, it was necessary to consider all four LCS types together and ascertain whether the assigned pseudosymmetry was consistent across all of them. Such a situation occurred for 4,611 out of 9,844 atoms from the 4n-C subgroup of the ref-SC dataset and for 4,883 out of 9,844 atoms for ref-NSC (Figure S3.1c left). If the assigned pseudosymmetry was inconsistent across the four LCS types, the lowest pseudosymmetry was chosen. The ratio between atoms with the pseudosymmetry consistent across all four LCS types to the ones where the pseudosymmetry was inconsistent was obviously rising while moving to atoms with lower pseudosymmetries. The ratio exhibited a slight difference depending on the fact if the symmetry constraints were applied or not during the refinement, especially for the no pseudosymmetry.

In general, approximately half of the atoms had the no pseudosymmetry assigned, for both the ref-SC and the ref-NSC datasets. The second most popular pseudosymmetry was 3m for the ref-SC (20%) but m for the ref-NSC (25%). The $\bar{4}$3m pseudosymmetry was the least often observed in both datasets (7% and 4% for ref-SC and ref-NSC, respectively).

Overall, a decrease in the number of atoms with a $\bar{4}$3m and 3m pseudosymmetries and an increase in the number of atoms with the mm2, m, and no pseudosymememetries could be observed for the ref-NSC in comparison to the ref-SC (Figure S3.1c left). When each atom was analyzed independently to find out how often final pseudosymmetry assigned to atom changed when symmetry constraints were removed from the refinement, it could be noted that 6,887 atoms (69.96%) had the same final pseudosymmetry assigned for the ref-SC and ref-NSC, while the remaining 2,997 atoms (30.44%) had a different final pseudosymmetry assigned. The most common changes of pseudosymmetry after releasing the symmetry constraints were from m to no, 3m to m, and from no to m.

Atom types in individual LCS and in LCS type

P_{lm} values for atom types were generated by *bankMaker* by averaging P_{lm} for all atoms belonging to that atom type. The averaging and subsequent pseudosymmetry assignment was done independently for each individual LCS orientation for atom types in the 4n-C subgroup from the ref-SC or from the ref-NSC datasets (Figure 3.1a right). Next, pseudosymmetries as seen from the perspective of the entire LCS type were assigned to atom types (Figure 3.1b right). Similar tendencies in pseudosymmetries distributions per the LCS type could be observed comparing to atoms, with one exception for the no pseudosymmetry. There was a major decrease in the percentage of the no pseudosymmetry and increase of the m, and in some cases of the mm2, pseudosymmetry comparing to atoms. This, however, might be simply the result of differences in in the number of atoms associated with particular atom type, see analysis of atom types vs atom pseudosymmetries.

The tendency to favor the 3m pseudosymmetry by the Z x1 X x2 R and Z (x1,x2,x3) X x1 R LCS types, and the mm2 pseudosymmetry by the X (x1,x2) Y x1 R LCS type was reversed for atom types (Figure S3.1b right). Distributions of pseudosymmetries in the Z x1 X x2 R and Z (x1,x2,x3) X x1 R LCS types were no longer so similar to each other as it was for atoms.

As the symmetry constraints were removed, shifts by 1 to 2% in the overall distribution of assigned pseudosymmetries were observed. The percentage of the $\bar{4}$3m pseudosymmetry decreased and with the mm2 pseudosymmetry increased in all LCS types (Figure 3.1b right). For other pseudosymmetries, the direction of the shift dependent on the LCS type.

Atom types

The overall distributions of the final electron density pseudosymmetries for atom types, taking into a consideration if the assigned pseudosymmetry was consistent across all LCS types or not, had changed comparing to pseudosymmetries of atom types in each LCS type independently (Figure S3.1c left, Table S4.1). Generally, the percentages of types with the 3m and m were reduced to ca. 20% and ca. 25 %, respectively, and the percentages of no symmetries increased to ca. 30%.

The overall tendency of the percentage share of each final pseudosymmetry showed small differences between the ref-SC and ref-NSC datasets. The number of atom types with the $\bar{4}$3m remained constant, while there was a slight decrease for 3m and no, and an increase for mm2 and m pseudosymmetries. When changes for individual atom types were compared, it could be noted that 100 atom types had the same final pseudosymmetry for both refinements, 32 atom types had different symmetries, and the proportion of the change of the pseudosymmetry to higher or lower was almost 50/50. These were mostly changes from no to m and from m to no. For atom types belonging to 3- or 4-member rings, the pseudosymmetry usually increased. Only one atom type among those for which the largest changes in P_{val} values were observed due to the release of symmetry constraints also changed pseudosymmetry – this was C419b belonging to the 4-member ring (from no to m).

Atom type vs atoms

Ultimately, the final pseudosymmetry assigned to each atom type was compared with the most common final pseudosymmetries assigned to individual atoms that belong to said atom type (Table S4.1). Finding the one most dominant pseudosymmetry among individual atoms was not always clearly evident. For example, for atom type C404a the no pseudosymmetry was assigned to 38.4 % of individual atoms and the mm2 pseudosymmetry to 38.0 % of individual atoms (Table S4.1). Overall, there were 90 atom types in the ref-NCS dataset, for which the most common pseudosymmetry among atoms belonging to that atom type was observed for 100% to 75% of individual atoms, 31 atom types for which the most common pseudosymmetry was observed for 74.99% to 50% of individual atoms, and 11 atom types for which the most common pseudosymmetry was observed for 49.99% to 25% (Table S4.1). Overall, the pseudosymmetry assigned to atom types from the ref-NCS dataset was the same for 58 atom types and higher for 74 atom types comparing to the most common pseudosymmetry among atoms belonging to that atom type (Table S4.1). Among the latest, 23 atom types had the pseudosymmetry the same as the second most common pseudosymmetry among the atoms belonging to that atom type (Table S4.1). Pseudosymmetry for 51 atom types (38%) was higher than the first and the second most common pseudosymmetry of atoms.

Atom types vs MATTS2021

Compared to the symmetry from the MATTS2021 data bank, the pseudosymmetry of the electron density from the ref-NSC was the same for 71, higher for 38, and lower for 23 atom types (Figure S3.2, Table S4.1).

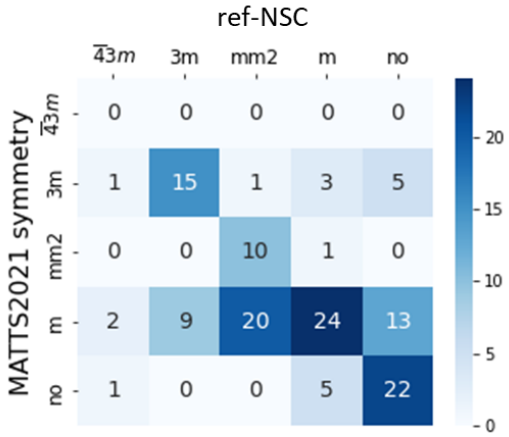

Figure S3.2 Heatmap comparing the pseudosymmetry from the ref-NSC assigned for 4n-C atom types with their original symmetry from the MATTS2021 data bank.

The changes occur mostly for atom types with the original m symmetry, either increasing to mm2 or decreasing to no. The effect was also visible when analyzing the overall distribution of atom types symmetries from the MATTS2021 data bank (Figure S3.1d right). The majority of atom types, which were assigned the higher pseudosymmetry than in the MATTS2021 data bank, were the ones where directions to the neighbors of different element type appeared to symmetrically the same. In addition, the higher pseudosymmetry for the eight atom types implied similarities between first neighboring carbon atoms of different hybridizations (having different number of their first neighbors), and the remaining three atom types having higher pseudosymmetry belonged to the 4-member rings. The decrease of the pseudosymmetry compared to the MATTS2021 was observed for 14 atom types that have at least three carbon atoms among the first neighbors. Nine of them were the atom types which also were assigned lower pseudosymmetry upon the release of symmetry constraints during the refinement. Apparently, bulky substituents, even if of the same type, may introduce asymmetric distribution of electron density around the central atom of the atom type.

The influence of 3- and 4-membered rings on the assigned pseudosymmetry appears to be ambiguous. From 26 atom types which cover all 4n-C types belonging to 3-member and/or 4-member rings, seven were assigned pseudosymmetry higher than in the MATTS2021 databank, and three – lower (Table S4.1).

S2. 4n-N, 4n-P, 4n-S

Atoms in individual LCS

The distributions of pseudosymmetries assigned to electron densities expressed in individual LCS orientations and grouped by the LCS type were different in details for each chemical element (Figures S3.3a, S3.4a, S3.5a left), although some general trends could be observed. Overall, the no pseudosymmetry was the most frequent and pseudosymmetries higher than m were usually assigned least frequently, with the exception for the X (x1,x2) Y x1 R LCS type, were percentage of the m pseudosymmetries was slightly larger than for the no. In general, the trends were same as were for the 4n-C subgroup. Sulfur atoms had the highest percentage of no pseudosymmetry assigned to individual LCS orientations, and phosphorus the lowest percentage. The $\bar{4}3m(\bar{4}\|z)$ pseudosymmetry, directly seen only in the Z (x1,x2) X x3 R LCS type, was the least frequent for individual LCS of phosphorus atoms. The observed trends concerning the influence of the LCS type on the pseudosymmetries that are present and dominant for individual LCS were analogous to those identified for carbon. The lowest percentage of orientations with no pseudosymmetry was observed in the X (x1,x2) Y x1 R LCS type for all chemical elements. The Z x1 X x2 R and the Z (x1,x2,x3) X x1 R LCS types led to very similar distributions of pseudosymmetries.

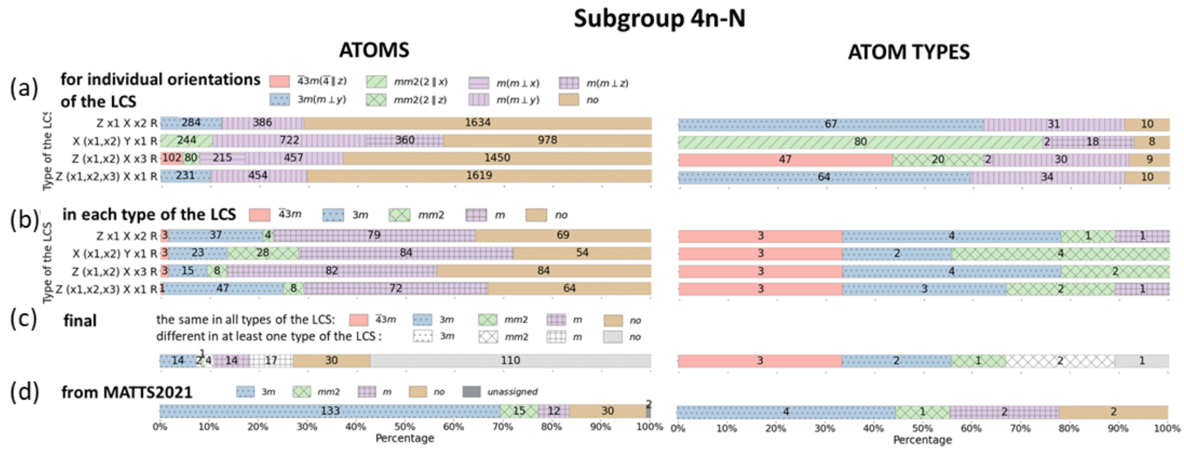

Figure S3.3A comparison of local pseudosymmetry of electron density (for individual LCS orientations in all four LCS types (a), in each LCS type (b), final pseudosymmetry (c)) assigned for non-planar nitrogen atoms (left) and atom types (right) with four first neighbors on the basis of the P_{lm} values. Symmetry for atoms and atom types in the MATTS2021 data bank is presented in the panel (d). Number of atoms with each pseudosymmetry is shown on the bars.

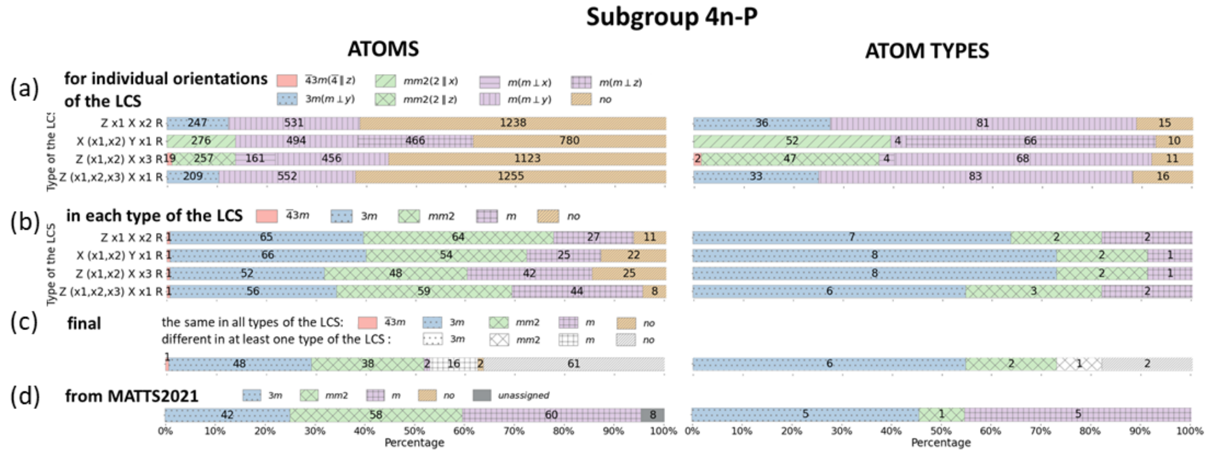

Figure S3.4A comparison of local pseudosymmetry of electron density (for individual LCS orientations in all four LCS types (a), in each LCS type (b), final pseudosymmetry (c)) assigned for non-planar phosphorus atoms (left) and atom types (right) with four first neighbors on the basis of the P_{lm} values. Symmetry for atoms and atom types in the MATTS2021 data bank is presented in the panel (d). Number of atoms with each pseudosymmetry is shown on the bars.

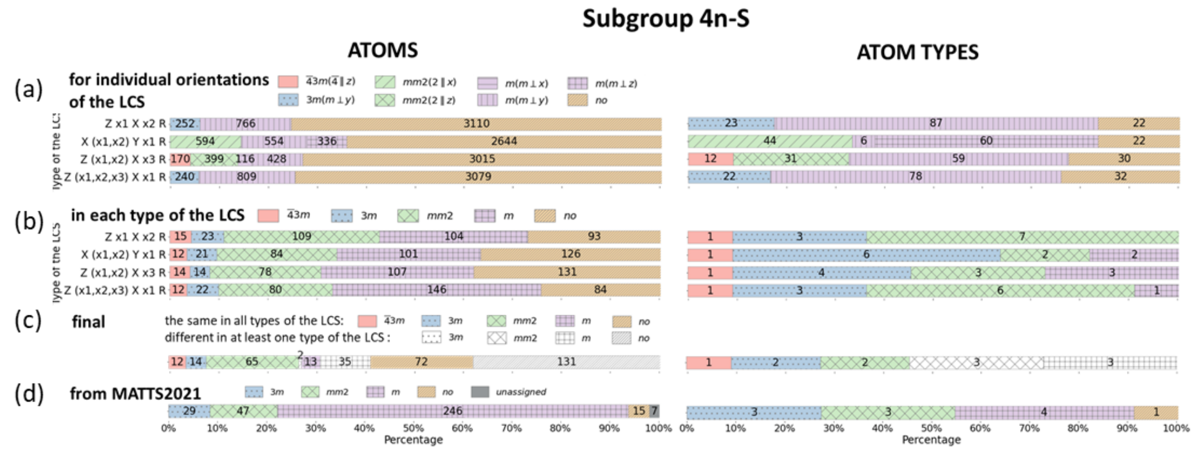

Figure S3.5A comparison of pseudosymmetry of electron density (for individual LCS orientations in all four LCS types (a), in each LCS type (b), final pseudosymmetry (c)) assigned for non-planar sulfur atoms (left) and atom types (right) with four first neighbors on the basis of the P_{lm} values. Symmetry for atoms and atom types in the MATTS2021 data bank is presented in the panel (d). Number of atoms with each pseudosymmetry is shown on the bars.

Atoms in LCS types

When information about pseudosymmetries from individual LCS orientations was combined to assign to atoms pseudosymmetries per each LCS type, the frequency of the no symmetry considerably dropped in each LCS type (Figures S3.3b, S3.4b, S3.5b left), similarly as it was for carbon. The distribution of pseudosymmetries specific for each chemical element from the 4n subgroup was found to differ, with exception for the $\bar{4}$3m pseudosymmetry. The $\bar{4}$3m pseudosymmetry was the least frequent for all chemical elements in each LCS type. For nitrogen, the distributions were dominated by m and no pseudosymmetries, regardless the LCS type and somewhat similar to carbon. For phosphorus, there was a significant percentage of atoms with 3m, mm2, and very little atoms with the no pseudosymmetries in all LCS types. For sulfur, the distribution was dominated by mm2, m, and no pseudosymmetries in all LCS types.

Individual differences in the ratio between pseudosymmetries in each LCS type could be observed, and these differences were usually similar as for carbon. The highest percentage of atoms with no pseudosymmetry was present in the Z (x1,x2) X x3 R LCS types. The 3m pseudosymmetry was observed most frequently in the Z x1 X x2 R and Z (x1,x2,x3) X x1 R types, with exception for phosphorus. The mm2 pseudosymmetry was the most prevalent in the X (x1,x2) Y x1 R type only for nitrogen. With respect to the individual LCS orientations, the Z x1 X x2 R and the Z (x1,x2,x3) X x1 R LCS types started to show slightly different distributions of pseudosymmetries, as it could be noted for carbon as well.

Atoms

The final pseudosymmetry assigned to nitrogen, phosphorus, and sulfur atoms from the 4n subgroup revealed (Figures S3.3c, S3.4c, S3.5c left) that the phosphorus atoms had the highest percentage of pseudosymmetries higher than no (62.5%), followed by sulfur (41.0%) and nitrogen (27.08%) atoms. Apart from the no pseudosymmetry, the second most occurring one was 3m for phosphorus (28.6%), mm2 for sulfur (19.5%), and m for nitrogen atoms (16.1%). All groups had a high percentage of atoms with m and no pseudosymmetries due to the inconsistency among pseudosymmetries from different LCS types. The $\bar{4}$3m pseudosymmetry was the least frequent for atoms of all chemical elements (less than 3.5%). Nitrogen atoms behaved somewhat the most similar to the carbon atoms in the 4n subgroup.

Atom types in individual LCS and in LCS types

The distributions of pseudosymmetries for atom types as revealed for individual LCS orientations and LCS types, were much different than for atoms. First of all, substantial reduction of atoms in individual LCS orientations having no pseudosymmetry was observed (Figures S3.3a, S3.4a, S3.5a right) and no single atom type in none of the LCS types was assigned the no pseudosymmetry after individual LCS orientations were analyzed together (Figures S3.3b, S3.4b, S3.5b right). In some of the LCS types for nitrogen and sulfur, the m pseudosymmetry disappeared as well. The percentage of the $\bar{4}$3m pseudosymmetries increased for nitrogen, but the $\bar{4}$3m pseudosymmetry was not observed for phosphorus atom types in none of the LCS types. Overall, the 3m and mm2 pseudosymmetries dominated among atom types of nitrogen, phosphorous and sulfur in all LCS types.

Atom types

Similarly to atoms, to assign final symmetry for atom types it was necessary to check whether the pseudosymmetry assigned in all LCS types was consistent or not. The existence of atom types with the $\bar{4}$3m pseudosymmetry was confirmed for three nitrogen and one sulfur type (Figure S3.3c, S3.4c, S3.5c right). Finally, there were one nitrogen and two phosphorous atom types with no pseudosymmetry. The most frequent final pseudosymmetry for phosphorous was 3m and for sulfur was mm2. Overall, the nitrogen, phosphorous and sulfur atom types in the 4n subgroup tended to have higher final pseudosymmetry than carbon.

Atom type vs atoms

The final pseudosymmetries of atom types were compared with the most frequently occurring pseudosymmetries among individual atoms within the same atom type (Tables S4.2-S4.4). For eight out of nine nitrogen, three out of 11 phosphorus, and six out of 11 sulfur atom types, the most common pseudosymmetry among individual atoms was lower than the pseudosymmetry of the given atom type. The remaining atom types had the same pseudosymmetry as the one most common among

its atoms, except for the P409c atom type, where the most common pseudosymmetry for atoms was higher (3m) compared to the pseudosymmetry for the atom type (no). The topological pseudosymmetry of P409c was 3m. It is worth mentioning that the most common pseudosymmetry assigned to individual atoms was no for all nine nitrogen atom types and all six sulfur atom types. When the second most common pseudosymmetry among atoms was considered, it was the same as their atom types only for one atom type (N401b). In that respect, nitrogen atom types behave similarly to carbon atom types. Sulfur and phosphorous atom types were different, when second most common pseudosymmetries for atoms were included, pseudosymmetries for almost all atom types agreed with them.

Atom types vs MATTS2021

Compared to the original symmetry of atom types from the MATTS2021 data bank, the most frequently observed changes were from m to mm2 in the case of phosphorus and sulfur, and from no to $\bar{4}3$m in the case of nitrogen (Figure S3.6, Tables S4.2-S4.4). This observation suggested that in some cases, the electron density fragments directed towards atoms of different element might be on average symmetrically equivalent. Here these could be electron densities directed to carbon and nitrogen (e.g. S405) or carbon and oxygen (e.g. P402) as the first neighbors, all atoms connected with the central atom by formally single bonds, as opposite to the remaining oxygen atoms as the first neighbors connected with formally double bonds. There were three nitrogen, five sulfur, and five phosphorus atom types with the same assigned pseudosymmetry as the symmetry in the MATTS2021 data bank.

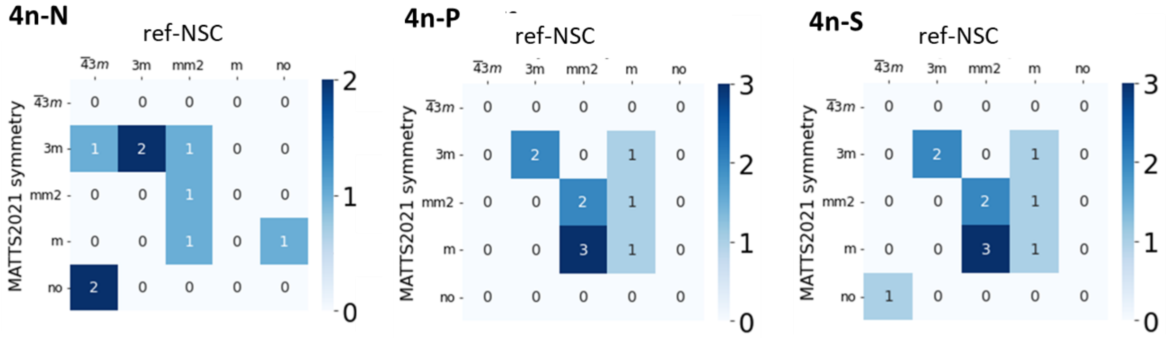

Figure S3.6 Heatmaps comparing the pseudosymmetry from the ref-NSC assigned for 4n-N, 4n-P, and 4n-S atom types with their original symmetry from the MATTS2021 data bank.

S3. 3n-N

It is necessary to keep in mind that for the 3n group, only 3m, m(non-planar), and no symmetries can be present in theory. The first two cannot be directly observed in individual LCS orientations of the Z x1 X x2 R LCS type, and the 3m symmetry cannot be directly observed in the X

(x1,x2) Y x1 R and Z (x1,x2) Y x1 R LCS types. In addition, the $\bar{6}$m2, mm2 and m(planar) symmetries should never be observed for the 3n group. It is important to note, that for the 3n group it is still, even indirectly, impossible to observe the symmetries higher than no in the Z x1 X x2 R LCS type. All the remaining LCS types, when their individual LCS orientations are considered together, allow to observe all symmetries expected for the 3n group.

Atoms in individual LCS

For 3n-N in the ref-SC dataset, more than 95% of atoms in individual LCS orientations belonging to the Z x1 X x2 R type had the no pseudosymmetry assigned (Figure 3.7a left). The remaining atoms were assigned with the m(m⊥y) and m(m⊥x) pseudosymmetries. The first one passes through the x1 and x2 neighbors, and implies that direction towards the third neighbor (not used to define x1 and x2) and towards the expected position of the lone electron pair are symmetry equivalent. The second one passes between atoms, and might only results from wrong symmetry constrains applied in the refinement. Ca. 60% of atoms in individual LCS orientations from the remaining three LCS types were assigned the no pseudosymmetries. Almost all other atoms were assigned the m(m⊥y) pseudosymmetries in the X (x1,x2) Y x1 R and Z (x1,x2) Y x1 R LCS types. Tiny fraction of unexpected mm2(2∥x), mm2(2∥z), and and $\bar{6}$m2(m⊥y) pseudosymmetries was observed as well in these two LCS types. In the Z (x1,x2,x3) X x1 R LCS type, all other atoms were assigned either m(m⊥y) or 3m(m⊥y), in almost 50/50 ratio, and no unexpected symmetries were assigned.

The relaxing of symmetry constrains during the refinement (the ref-NSC dataset) resulted with disappearance of all pseudosymmetries unexpected for the 3n group (Figure 3.7a left). This confirms, that especially for the 3n-N subgroups it was important to remove wrongly assigned constraints. Also, the percentage of no pseudosymmetries decreased by ca. 10 % in the X (x1,x2) Y x1 R and Z (x1,x2) Y x1 R LCS types, and increased by ca. 10 % in the Z (x1,x2,x3) X x1 R LCS type upon removal of symmetry constraints.

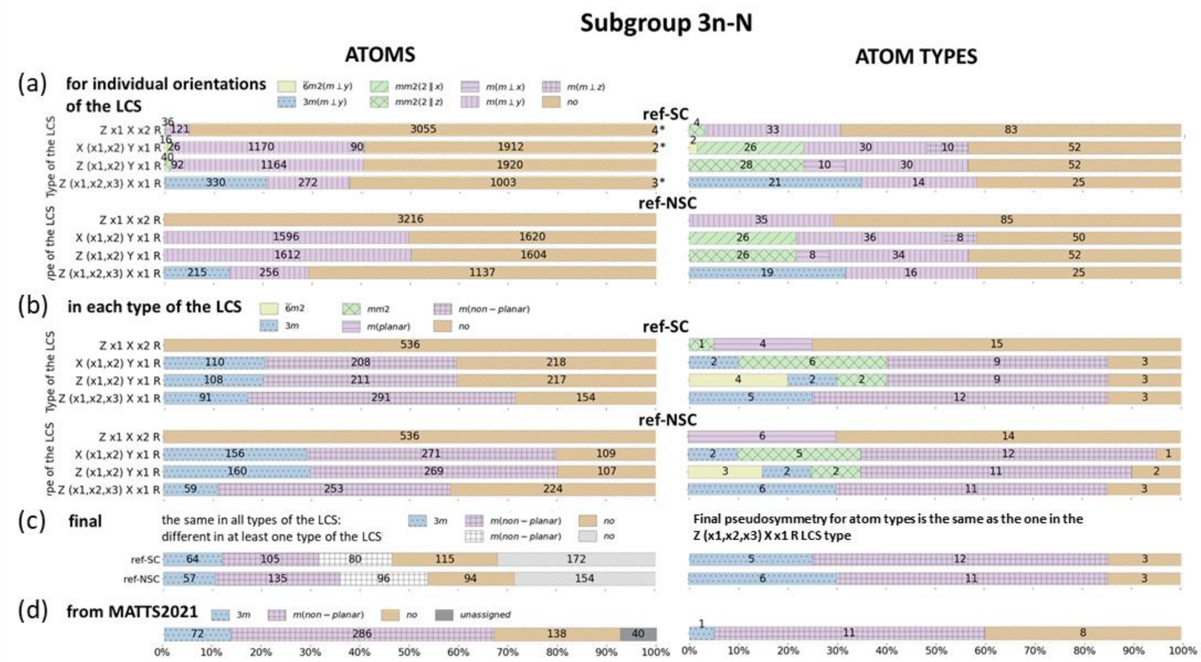

Figure S3.7A comparison of pseudosymmetry of electron density (for individual LCS orientations in all four LCS types (a), in each LCS type (b), final pseudosymmetry (c)) assigned for non-planar nitrogen atoms (left) and atom types (right) with three first neighbors on the basis of the P_{lm} values for ref-SC and ref-NSC. Symmetry for atoms and atom types in the MATTS2021 data bank is presented in the panel (d). Number of atoms with each pseudosymmetry is shown on the bars. For clarity purposes, categories with a small count of instances were summed up and presented at the end of the bars with a mark *.

Atoms in LCS types

By considering all individual LCS orientations together, the 3m pseudosymmetries were also possible to be observed in the X (x1,x2) Y x1 R and Z (x1,x2) Y x1 R LCS types and thus, the percentage for the no pseudosymmetries was reduced in these LCS types (Figure S3.7b left). In the Z (x1,x2,x3) X x1 R LCS type, the percentage of the m(non-planar) pseudosymmetry increased several times.

With symmetry constraints removed, the differences in distributions of pseudosymmetries in the X (x1,x2) Y x1 R and Z (x1,x2) Y x1 R LCS type as compared to the Z (x1,x2,x3) X x1 R LCS type increased (Figure S3.7b left). The later showed less 3m pseudosymmetries in benefit for the no pseudosymmetries.

Atoms

To assign the final pseudosymmetry for nitrogen atoms belonging to the 3n-N subgroup - only the X (x1,x2) Y x1 R, Z (x1,x2) Y x1 R, and Z (x1,x,2,x3) X x1 R LCS types were taken into account, because in the Z x1 X x2 R none of the higher than no pseudosymmetries can be observed.

Nitrogen atoms belonging to the 3n-N subgroup had finally only non-planar pseudosymmetries assigned (Figure S3.7c left). 284 (53.0%) atoms in ref-SC and 286 (53.4%) atoms in ref-NSC had the same pseudosymmetries assigned consistently across all LCS types. The no pseudosymmetry dominated, followed by slightly smaller percentage of m(non-planar) pseudosymmetry. The 3m pseudosymmetry was finally assigned to only 11.9 % and 10.6 % of atoms for ref-SC and ref-NSC, respectively.

After releasing symmetry constraints, mostly a decrease in the number of atoms with no pseudosymmetry was observed, similarly as previously for planar atoms. Simultaneously, the number of atoms with the m(non-planar) pseudosymmetry increased.

Focusing on individual pairs of atoms showed that for 346 (65 %) out of 536 atoms from the 3n-N subgroup the pseudosymmetry was not changed by the release of the symmetry constraints. For the remaining atoms, the pseudosymmetries switched often to lower ones (an opposite trend to 3p-N), most frequently from m(non-planar) to no or from 3m to m(non-planar).

Atom types in individual LCS and LCS types

For nitrogen atom types belonging to the 3n-N subgroup, the distributions of pseudosymmetries for individual LCS orientations and across LCS types significantly differed (Figure S3.7a-b right) compared to atoms. The unexpected planar pseudosymmetries appeared in much larger percentage in individual LCS orientations and entire sets of the Z x1 X x2 R, X (x1,x2) Y x1 R, and Z (x1,x2) Y x1 R LCS types for the ref-SC dataset. Only the mm2(2‖z) in Z x1 X x2 R LCS type disappeared after symmetry release in the ref-NCS dataset.

This situation happens due to the fact that in these three LCS types there is no control over how the second axis of the LCS will be oriented in respect to the lone electron pair of the central nitrogen atom and the third first neighbor. During the averaging of nitrogen atoms belonging to the 3n-N subgroup expressed in these LCS types, the orientation of the lone electron pair was not preserved. Atoms with lone pair oriented in the opposite directions were averaged leading to appearance of artificial planar pseudosymmetries mixing the electron density of the lone pair with the electron density directed to one of the neighboring atoms. This had its further consequences in observing significant percentage of planar $\bar{6}$m2, mm2, and m(planar) pseudosymmetries when individual LCS orientations of atom types were analyzed together in the three above mentioned LCS types (Figure S3.7b right). Thus why, the Z (x1,x2,x3) X x1 R LCS type involving all three neighbors and orienting the Z-axis along the lone electron pair of nitrogen, is necessary to properly average multipole model parameters for the 3n group of atoms.

In individual LCS orientations of the Z (x1,x2,x3) X x1 R LCS type in ref-SC, only pseudosymmetries expected for the 3n group appeared, 3m(m⊥y) in 35%, m(m⊥y) in 20% and no in 45% of cases. After symmetry constraints removal, percentages of 3m(m⊥y) very slightly decreased

and of m(m⊥y) increased (Figure S3.7a right). Distribution of pseudosymmetries assigned in the Z (x1,x2,x3) X x1 R type changed considerably for the m(non-planar) pseudosymmetries, which increased to 60% and thus the no pseudosymmetries decreased to 15% (Figure S3.7b right).

Atom types

Due the reasons discussed above, it is not appropriate to assign final pseudosymmetry of 3n-N atom types taking into account all analyzed LCS types. The proper final pseudosymmetry of 3n-N atom types can only be found in the Z (x1,x2,x3) X x1 R LCS type, which was the approach applied here. The Z x1 X x2 R LCS type is also suitable, but only in cases when x1, x2, and x3 are topologically distinguishable in the definition of the atom type and that the chiral option is applied. However, it was not possible to use this option for all atom types in the present work.

The final pseudosymmetries of the 3n-N atom types for ref-SC were distributed as follows: 30% in 3m, 55% in m, and 15% in no (Table S4.5, Figure S3.7c right). After the removal of symmetry constraints only one atom type (N304s) changed their symmetry, from m(non-planar) to 3m (Table S4.5).

Atom types vs atoms

As for previous subgroups, the next step of the analysis is a comparison of the pseudosymmetry assigned to the atom type and the pseudosymmetry most commonly assigned to individual atoms within the atom type (Table S4.5). For 12 out of 20 atom types the pseudosymmetry most commonly assigned to individual atoms was lower than for the corresponding atom type, and for the remaining eight – the same.

Atom types vs MATTS2021

Compared to the symmetry from the MATTS2021 data bank, nine 3n-N atom types in ref-NSC changed their symmetry from lower to a higher one (Figure S3.8, Table S4.5). There were no changes to a lower symmetry than the one defined in the MATTS2021 databank.

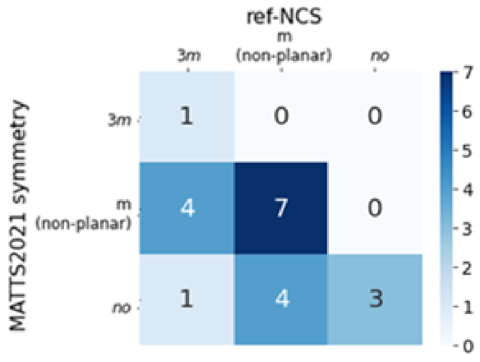

Figure S3.8 Heatmap comparing the pseudosymmetry from the ref-NSC assigned for 3n-N atom types with their original symmetry from the MATTS2021 data bank.

S4. 3p-C

Atoms in individual LCS

For 3p-C electron densities expressed in individual LCS orientations, the m pseudosymmetries where the mirror plane is lying in the plane of the atom (m⊥y in Z x1 X x2 R, m⊥z in X (x1,x2) Y x1 R, m⊥x in Z (x1,x2) Y x1 R) dominated (ca. 60%), followed by no (ca. 35%) (Figure S3.9a left). Less than 7% of atoms was assigned the mm2(2∥x or 2∥z) pseudosymmetry. The 6̅m2(m⊥y) pseudosymmetry, which is possible to be directly observed only in the X (x1,x2) Y x1 R LCS type, was assigned to only 0.07% of individual LCS orientations of atoms. Interestingly, the m pseudosymmetries where the mirror plane is perpendicular to the plane of the atom (m⊥x in Z x1 X x2 R, m⊥y in X (x1,x2) Y x1 R, m⊥y in Z (x1,x2) Y x1 R) were observed for tiny fractions of atoms. The percentage distributions of pseudosymmetries were almost identical in each LCS type.

The existence of 3m(m⊥y), m where the mirror plane is perpendicular to the plane of the atom, or no pseudosymmetries for atoms in individual LCS orientations from the 3p group can only be explained by the situation, where, although the atom and its first neighbors (their nuclei) are genuine planar, the electron density of that atom is different on one side of the plane than on the another. Alternative explanation is that the group planarity threshold was too high, and the atom is not planar enough. And of course, it should also be kept in mind that the threshold value used to decide which P_{lm} is zero is certain approximation.

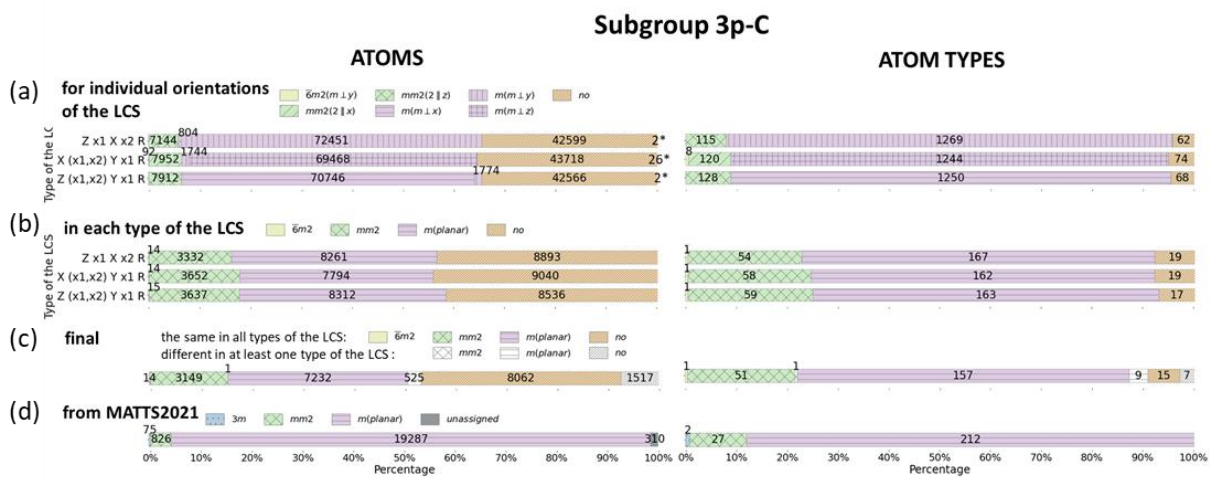

Figure S3.9A comparison of local pseudosymmetry of electron density (for individual LCS orientations in all three LCS types (a), in each LCS type (b), final pseudosymmetry (c)) assigned for planar carbon atoms (left) and atom types (right) with three first neighbors on the basis of the P_{lm} values. Symmetry for atoms and atom types in the MATTS2021 data bank is presented in the panel (d). Number of atoms with each pseudosymmetry is shown on the bars. For clarity purposes, categories with a small count of instances were summed up and presented at the end of the bars with a mark *.

Atoms in LCS types

The distribution of pseudosymmetries assigned per each LCS type, by considering all the individual orientations of the given LCS type together (Figure S3.9b left) showed slight increase (up to 17.5%) in percentage of the mm2 pseudosymmetries comparing to individual LCS orientations. The m(planar) and no pseudosymmetries dominated distributions in each LCS type. The $\bar{6}$m2 pseudosymmetry was assigned to 0.07% of atoms. The m(non-planar) pseudosymmetry was not confirmed and apparently was not genuine within the used approximations. The distributions of $\bar{6}$m2, mm2, m(planar), and no pseudosymmetries were very comparable for each LCS type, similarly as it was for individual LCS orientations.

Atoms

The final pseudosymmetry, determined through considering the pseudosymmetries assigned in all LCS types, was consistent across all the types for 90% carbon atoms belonging to the 3p-C subgroup (Figure S3.9c left). Majority of the atoms had either m(planar) or no pseudosymmetry assigned, 15% had the mm2 pseudosymmetry, whereas the $\bar{6}$m2 very rarely occurred (0.07%). Atoms with the no pseudosymmetry had the ”planarity esd” values ranging from 0 to 0.097788, with the median of 0.009331, whereas for the remaining atoms these were from 0 to 0.017084, with median of 0.000285. This suggest, that for some of the 3p-C atoms, the no pseudosymmetry resulted from not ideal planar arrangement of atoms and for others the arrangement was ideally planar, but the electron density was polarized differently from one side of the plane than from the other.

Atom types in individual LCS and in LCS types

Comparing to atoms, percentages contributions from the no pseudosymmetries assigned to individual LCS orientations of atom types belonging to the 3p-C subgroup were lowered considerably, to less than 5% (Figure S3.9a right). The m pseudosymmetries where the mirror plane is lying in the plane of the atom dominated (>85 %). The m pseudosymmetries the mirror plane is perpendicular to the plane of the atom were not observed. The percentages of the mm2(2∥x or 2∥z) and the $\bar{6}$m2(m⊥y) were very similar compared to atoms, 8.3% and 0.5%, respectively.

When all individual LCS orientations were analyzed together within the given LCS type (Figure S3.9b right), the percentage of the mm2 pseudosymmetries increased to ca. 24% on the expenses of the m(planar) pseudosymmetries. One atom type in each LCS type had the $\bar{6}$m2 pseudosymmetry. Distributions were very similar for each LCS type.

Atom types

The analysis final pseudosymmetries assigned by checking the consistency of the pseudosymmetry assignments across all the three LCS types (Figure S3.9c right), confirmed that there was only one atom type with the $\bar{6}$m2 pseudosymmetry (C332a, representing atoms belonging to three

different planar six-member rings), 22% of atom types had the mm2, and 70% had the m(planar) pseudosymmetries. 9% of atom types (22 types) still remained with the no pseudosymmetry.

Atom types vs atoms

The final pseudosymmetries of 3p-C atom types were compared with the most common pseudosymmetries among individual atoms that belong to given atom type. The pseudosymmetry assigned to atom types was the same for 68 atom types, higher for 170 atom types and lower for three atom types (C352b, C387, C332a) comparing to the most common pseudosymmetry among atoms (Table S4.6). Within the latest two, 112 atom types had the pseudosymmetry the same as the second most common pseudosymmetry among the atoms. Pseudosymmetry for 60 atom types (25%) was higher than the first and the second most common pseudosymmetry of atoms.

The $\bar{6}$m2 pseudosymmetry of atom type C332a was also the most common among atoms belonging to that atom type. Only four atom types with the mm2 pseudosymmetry were composed from atoms with the same pseudosymmetry being the first or second most common (C330, C569T, C706b), majority of them (77%, 40 types) were dominated with atoms having the m(planar) or no pseudosymmetries, and 8 types with atoms of only no pseudosymmetry. For atom types with the m pseudosymmetry, 92% of them (153 types) had atoms with the same pseudosymmetry being among the first or second most common pseudosymmetries, one atom type (C332c) had atoms with the mm2 pseudosymmetry as the most common, and 12 atom types had atoms only with the no pseudosymmetry. Half of the atom types with the no pseudosymmetry contained many atoms with the m(planar) symmetry, second half was composed solely from atoms with no symmetry. Atom types with the no pseudosymmetry assigned had the tendency to be composed from atoms having planarity esd threshold slightly larger than the other atoms (Table S4.6).

The vagueness of the mm2 pseudosymmetry assignment to atom types from the 3p-C subgroup most probably is connected with the chemistry of such carbon atoms. In the most common scenarios, they are either part of aromatic ring or delocalized system. Then often the neighbors have the same topology (implying mm2 or higher symmetry) but some alternation in bonds might still be present (non-equivalent electron densities pointing to neighbors of the same topology).

Atom types vs MATTS2021

Compared to the symmetry from the MATTS2021 data bank, 170 atom types had the same pseudosymmetry assigned as in the MATTS2021, whereas for 38 atom types the assigned pseudosymmetry was higher and for 33 atom types it was lower (Figure S3.10). The most common change was from m in MATTS2021 to mm2, and then from m to no. Additionally, two atom types in the MATTS2021 databank (C522 and C310) had an incorrect 3m symmetry specified, in this analysis we found out the pseudosymmetries mm2 and m to be more accurate for these atom types.

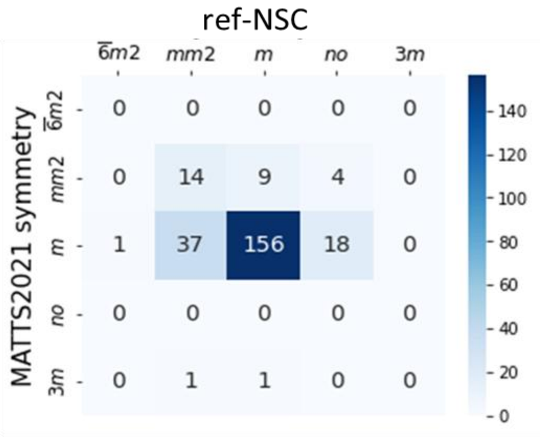

Figure S3.10 Heatmap comparing the pseudosymmetry from the ref-NSC assigned for 3p-C atom types with their original symmetry from the MATTS2021 data bank.

S5. 3p-N

Atoms in individual LCS

For 3p-N, the distributions of pseudosymmetries assigned to individual LCS orientations were somewhat similar to carbon (Figure S3.11a left). The m pseudosymmetries the mirror plane is lying in the plane of the atom were assigned to ca. 60% of atoms, but the percentage of the no pseudosymmetries was around twice smaller, the percentage of the mm2(2∥z) was higher in the Z x1 X x2 R and Z (x1,x2) Y x1 R LCS types than mm2(2∥x) in the X (x1,x2) Y x1 R LCS type. Larger percentage of atoms (10%) in the X (x1,x2) Y x1 R LCS type was assigned 6̅m2(m⊥y) pseudosymmetry. Also here, tiny fraction of atoms were assigned with m pseudosymmetries where the mirror plane is perpendicular to the plane of the atom.

After releasing the symmetry constraints, up to couple of percentage increase or decrease in pseudosymmetries was observed, with visible increase of m pseudosymmetries where the mirror plane is perpendicular to the plane of the atom and decrease in m pseudosymmetries where the mirror plane is lying in the plane of the atom, for individual LCS orientations of X (x1,x2) Y x1 R and Z (x1,x2) Y x1 R types, and increase in m where the mirror plane is lying in the plane of the atom and decrease in mm2(2∥z) pseudosymmetries for Z x1 X x2 R LCS type.

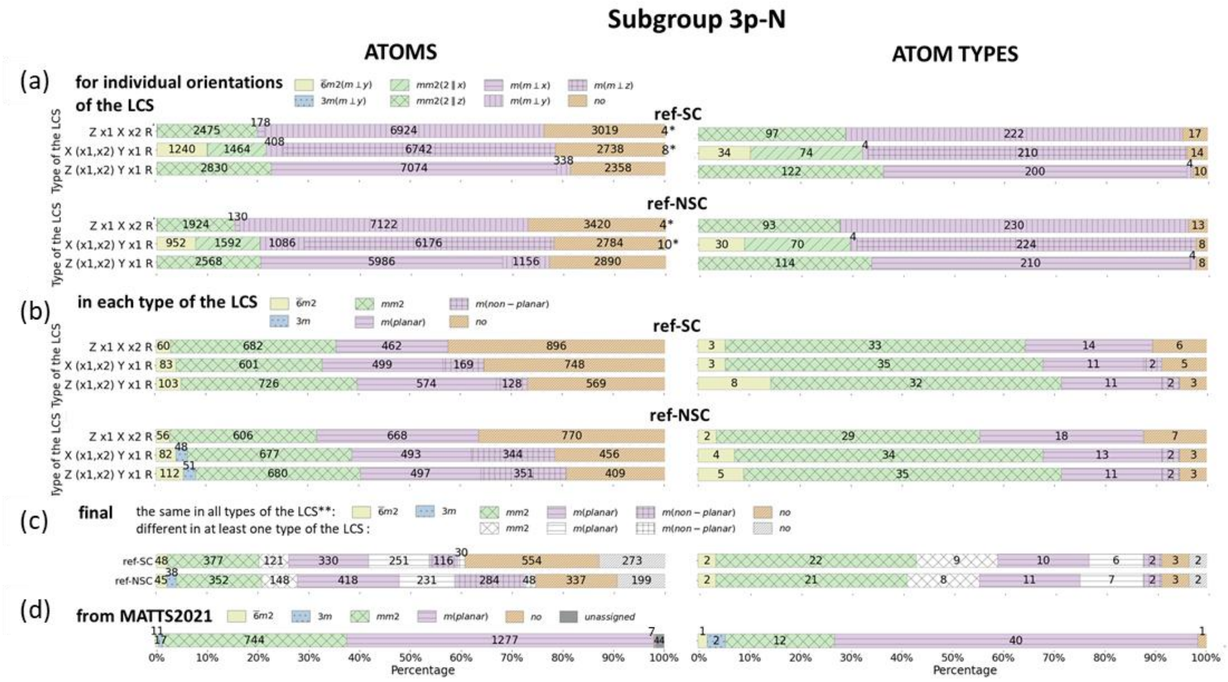

Figure S3.11 A comparison of pseudosymmetry of electron density (for individual LCS orientations in all three LCS types (a), in each LCS type (b), final pseudosymmetry (c)) assigned for planar nitrogen atoms (left) and atom types (right) with three first neighbors on the basis of the P_{lm} values for ref-SC and ref-NSC. Symmetry for atoms and atom types in the MATTS2021 data bank is presented in the panel (d). Number of atoms with each pseudosymmetry is shown on the bars. For clarity purposes, categories with a small count of instances (potentially errors) were summed up and presented at the end of the bars with a mark *. (double column)

Atoms in LCS types

Distributions of pseudosymmetries resulting from combined analysis of all individual LCS orientations from the same LCS type together changed considerably comparing to individual LCS alone (Figure S3.11b left). The largest increases were observed for the mm2 and no pseudosymmetries and decrease for m(planar). The m(non-planar) pseudosymmetries disappeared for the Z x1 X x2 R LCS type, they cannot be observed in this LCS type, but remained in the other two LCS types.

Upon symmetry release, more m(planar) pseudosymmetries appeared at the expenses of the mm2 and no pseudosymmetries for the Z x1 X x2 R LCS type. Distributions for the X (x1,x2) Y x1 R and Z (x1,x2) Y x1 R types became very similar, with couple of percentage of 3m pseudosymmetries appearing, a little bit more percentages of $\bar{6}$m2 pseudosymmetries, and very similar contributions from m(planar), m(non-planar), and no pseudosymmetries, meaning that the percentage of the m(non-planar) pseudosymmetries increased upon symmetry constraint release.

Atoms

Out of the 2,100 planar atoms belonging to the 3p-N subgroup, the final pseudosymmetry was consistently the same in all available LCS types for 1,425 (68%) atoms from the ref-SC dataset and 1,474 (70%) atoms from ref-NSC (Figure S3.11c left). The final pseudosymmetries were somewhat similar to pseudosymmetries observed for X (x1,x2) Y x1 R and Z (x1,x2) Y x1 R LCS types. For the ref-NSC, the distribution was dominated by the no, m(planar), mm2, and m(non-planar) pseudosymmetries (between 15 and 30%) and the $\bar{6}$m2 and 3m were rarely assigned (3% each). Before release of the symmetry constraints, more atoms had no pseudosymmetry instead of m(non-planar) and 3m was not observed, what is confirmed by direct analysis of individual atom pairs: one atom from ref-SC vs the same atom from ref-NCS.

Comparing the assignment of the pseudosymmetry between ref-SC and ref-NSC by focusing on individual atoms (not just on overall distributions of pseudosymmetries) showed that the pseudosymmetry was the same for 1,306 (62%) out of 2,100 atoms from the 3p-N subgroup. Following the release of symmetry constraints, a transition from no to m(non-planar) was most commonly observed. Some atoms switched between planar and non-planar pseudosymmetries, and vice versa.

The appearance of m(non-planar) and 3m pseudosymmetries for 3p-N atoms could be the results of a situation in which electron density from one side of the atom plane is difference than from the other side, despite the existence of symmetry equivalence in directions of three or two neighboring atoms. Another explanation is, that the atom is in fact non-planar and the planarity threshold was set to too high value.

Atom types in individual LCS and in LCS types

When atom types were generated from 3p-N atoms and distributions of their pseudosymmetries for individual LCS orientations were analyzed independently (Figure S3.11a right) and together within the given LCS type (Figure S3.11b right), it was clear that no and m(non-planar) pseudosymmetries almost disappeared among atom types. For individual LCS orientations, the percentage of m pseudosymmetries where the mirror plane is lying in the plane of the atom increased the most (up to 70%), compared to atoms. For LCS types, the percentage of mm2 pseudosymmetries increased the most, compared to atoms.

Atom types

The final pseudosymmetries of 3p-N atom types for ref-NSC were covered by 55.36% of mm2, 28.57% of m(planar), 8.93% of no (N334, N3593, N325a, N4511, N454e), 3.57% of $\bar{6}$m2 (N333a, N339) and 3.57% of m(non-planar) (atom types N333b and N338). Upon the release of symmetry constraints, five atom types (N334, N3593, N325a, N458, N319) changed their symmetry

from higher to lower and six (N3592, N447, N318, N3162) from lower to higher. 45 atom types remained unchanged (Table S4.7).

Atom types vs atoms

Comparing final pseudosymmetry of each 3p-N atom type with the most frequently observed pseudosymmetry among its constituent atoms, 24 atom types retained the same pseudosymmetry, 30 exhibited a higher pseudosymmetry, one (N3593) had lower and one (N319) had m(planar) whereas dominating pseudosymmetry for atoms was m(non-planar) (Table S4.7). Half of atom types with the higher pseudosymmetry than the first most common pseudosymmetry among atoms, had the same pseudosymmetry as the second most common among atoms. 14 atom types were constituted from atoms with lower pseudosymmetries dominating. These were exclusively atom types with the mm2 pseudosymmetry. For 10 atom types, the most common pseudosymmetry of their atoms was m(non-planar), and for another eight atom types, the m(non-planar) pseudosymmetry was the second most common among atoms.

The atom types with the m(non-planar) pseudosymmetries were N333b and N338, were built from atoms among which the m(non-planar) pseudosymmetry dominated. Atom type N333b with three bulky sp^3 carbon neighbors, from chemical point of view should be non-planar but was classified planar within the applied planarity threshold which suggest the value of the threshold was not appropriate. Atom type N338 with two bulky sp^3 carbon neighbors and one nitrogen of any kind, again should be chemically non-planar.

Similarly as for 3p-C, the mm2 pseudosymmetry was the most elusive one among 3p-N atom types. What is novel, comparing to the 3p-C, is existence of so many atom types with large percentage of atoms having m(non-planar) pseudosymmetry.

Compared to symmetries associated with atom types in the MATTS2021 data bank, 31 atom types from the ref-NSC had the same pseudosymmetry, 19 had higher, and five had lower than in the MATTS2021 (Figure S3.12). One atom type (N338) had m(non-planar) instead of m(planar). The majority of atom types with higher than MATTS2021 pseudosymmetry had the mm2 pseudosymmetry instead of m (18 types). The types that had the incorrect for 3p group 3m symmetry specified in the MATTS2021 databank, their symmetry changed to $\bar{6}$m2 for N333a and m(non-planar) for N333b. The most noticeable drop in symmetry was observed for one atom type – N334, where the symmetry changed from mm2 to no.

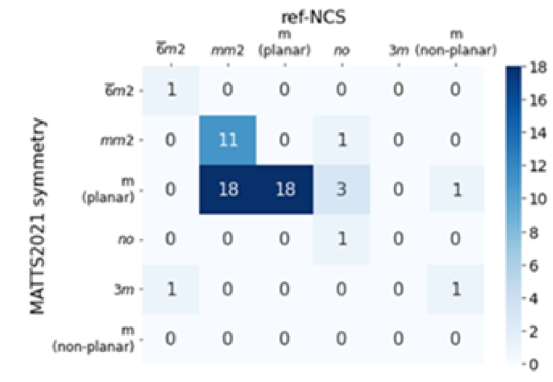

Figure S3.12 Heatmap comparing the pseudosymmetry from the ref-NSC assigned for 3p-N atom types with their original symmetry from the MATTS2021 data bank.

S6. 2p-N, 2p-O, 2p-S

Atoms in individual LCS and in LCS types

Distributions of pseudosymmetries for individual LCS orientations were dominated by the m pseudosymmetries, their total percentage share ranged from 60 % to 90 %. The ratio between pseudosymmetries mm2 and m was different for each chemical element, with a percentage of the mm2 pseudosymmetry increasing from 11% to 28% in the order: nitrogen, sulfur, oxygen (Figures S3.13a, S3.14a, S3.15a left). It should be noted that the planar mm2 symmetry cannot be observed in the Z x1 X x2 R LCS type. The percentage of the no pseudosymmetry almost did not depend on the LCS type, and was 12% for nitrogen and ca. 18% for oxygen and sulfur. Tiny fraction of atoms were assigned the m pseudosymmetry with the mirror plane perpendicular to the atom plane and passing in-between the two neighboring atoms, implying that the electron density fragments directed to the two neighboring atoms are equivalent but electron densities below and above the atom plane are not.

Pseudosymmetries assigned to atoms by combinations of all individual LCS orientations within the particular LCS type showed almost the same distributions as for individual LCS orientations (Figures S3.13b, S3.14b, S3.15b left). The common trend that can be observed for oxygen, nitrogen, and sulfur atoms is the minimal difference in the distribution of assigned pseudosymmetries between the X (x1,x2) Y x1 R and Z (x1,x2) Y x1 R LCS types.

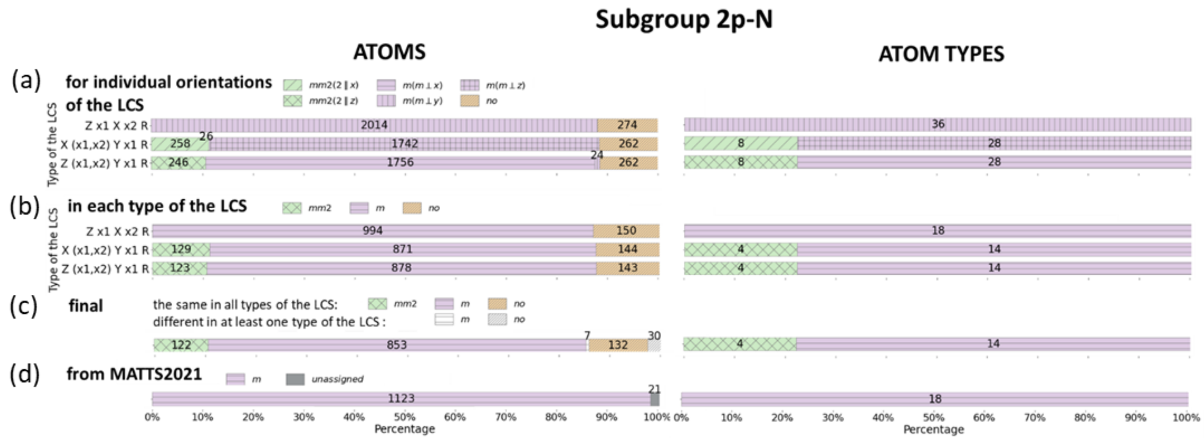

Figure S3.13 A comparison of pseudosymmetry of electron density (for individual LCS orientations in all three LCS types (a), in each LCS type (b), final pseudosymmetry (c)) assigned for planar nitrogen atoms (left) and atom types (right) with two first neighbors on the basis of the P_{lm} values. Symmetry for atoms and atom types in the MATTS2021 data bank is presented in the panel (d). Number of atoms with each pseudosymmetry is shown on the bars.

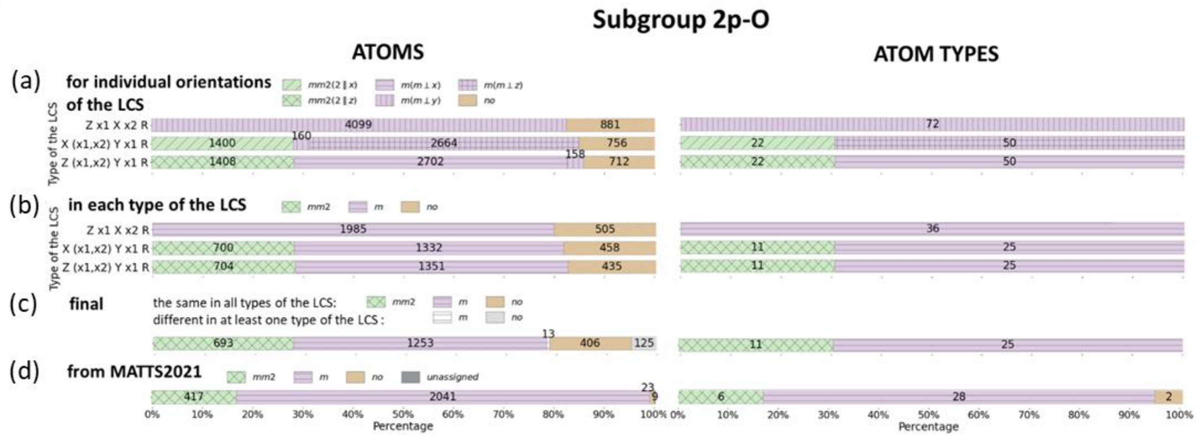

Figure S3.14 A comparison of pseudosymmetry of electron density (for individual LCS orientations in all three LCS types (a), in each LCS type (b), final pseudosymmetry (c)) assigned for planar oxygen atoms (left) and atom types (right) with two first neighbors on the basis of the P_{lm} values. Symmetry for atoms and atom types in the MATTS2021 data bank is presented in the panel (d). Number of atoms with each pseudosymmetry is shown on the bars.

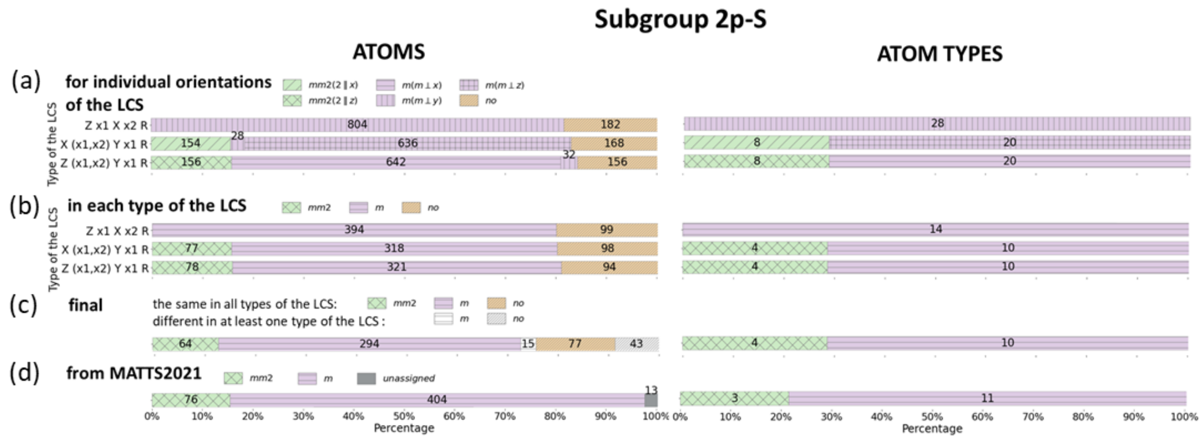

Figure S3.15 A comparison of pseudosymmetry of electron density (for individual LCS orientations in all three LCS types (a), in each LCS type (b), final pseudosymmetry (c)) assigned for planar sulfur atoms (left) and atom types (right) with two first neighbors on the basis of the P_{lm} values. Symmetry for atoms and atom types in the MATTS2021 data bank is presented in the panel (d). Number of atoms with each pseudosymmetry is shown on the bars.

Atoms

The distributions of final pseudosymmetries for 2p atoms (Figures S3.13c, S3.14c, S3.15c left) closely followed the trends observed for the X (x1,x2) Y x1 R and Z (x1,x2) Y x1 R LCS types. Notably, even though the lowest possible pseudosymmetry for a planar group should be m, a significant percentage of atoms had the no pseudosymmetry assigned, implying that their electron densities below and above the atom plane are not equivalent.

Atom types

Regardless if pseudosymmetries for individual LCS orientations of atom types are analyzed or for entire LCS type together, their distributions are the same (Figures S3.13a-b, S3.14a-b, S3.15a-b right). None of the nitrogen, oxygen, or sulfur atom types has no pseudosymmetry assigned. The ratio between mm2 and m pseudosymmetry of the electron density assigned to atom types is comparable between oxygen and sulfur (30/70), but for nitrogen percentage share of the mm2 pseudosymmetry is lower (22%).

The final pseudosymmetries for nitrogen, oxygen, and sulfur had the same distributions as for the X (x1,x2) Y x1 R and Z (x1,x2) Y x1 R LCS types (Figures S3.13c, S3.14c, S3.15c right).

Atom types vs atoms

Evaluation of the final pseudosymmetries of the 2p atom types against the first and the second most frequently observed pseudosymmetries of their individual atoms (Tables S4.8-S4.10) revealed

that all but two (N204, N207b) nitrogen, all oxygen, and all but two (S203, S208) sulfur atom types showed the same pseudosymmetry.

Atom types vs MATTS2021

A majority of 2p nitrogen, oxygen, and sulfur atom types had the m pseudosymmetry in the MATTS2021 data bank (Figure S3.15d right). Compared to that, the pseudosymmetry of the electron density changed to a higher one (mm2) for four nitrogen (N204, N207b, N210, N211), five oxygen (O202d, O206, O216, O230, O231), and two sulfur (S205, S209) atom types (Tables S4.8-S4.10). In each case, both first neighbors were of the same chemical element (hydrogen, carbon, or nitrogen).

S7. 1p-O, 1p-halogens

Atoms

The only LCS type considered in this group was Z x1 X x2 R with two or three possible orientations, depending on the number of the second neighbors, towards which the X axis was oriented to. The distribution of pseudosymmetries assigned to individual LCS orientations varied between the atoms with two and three second neighbors. The m and no pseudosymmetries were assigned to approximately half of the atoms, both with two or three second neighbors (Figure S3.16a, S3.17a left). The assignment of cyl pseudosymmetry was by far more frequent for atoms with three second neighbors, with occasional occurrences of 3m(m⊥y) and mm2(2∥z) pseudosymmetries. Instead, the mm2(2∥z) pseudosymmetry was more frequently observed for atoms with two second neighbors.

It is important to keep in mind the presence of two lone electron pairs on oxygen atoms and three lone electron pairs on halogen atoms when interpreting the assigned pseudosymmetry. The mm2 symmetry is unexpected in the case of three second neighbors. Likewise, one of the m symmetries is also unexpected. In addition, the no symmetry for two second neighbors is not expected. However, such a situation may arise in several cases, for example, (a) when two electron pairs do not lie in the plane of the first neighbor and are not perpendicular to it, (b) when the second neighbor is not planar, (c) when there are three electron pairs, but none of them lies in the plane of the first neighbor or perpendicular to it, (d) when electron pairs are aligned proportionally with the neighbors but are not equivalent. Similarly, when m symmetry appears instead of mm2 for the case of two second neighbors, and under the assumption that electron pairs lie in the plane of the first neighbor, this indicates that the electron pairs are not equivalent.

There were no big differences in pseudosymmetries distributions between oxygen and halogen atoms, the later had a bit larger percentage of the m symmetries over the no symmetries.

Due to difficulties in determining the pseudosymmetry for atoms with a high certainty by taking into account all individual LCS orientations together, no analysis of pseudosymmetry for individual atoms was done.

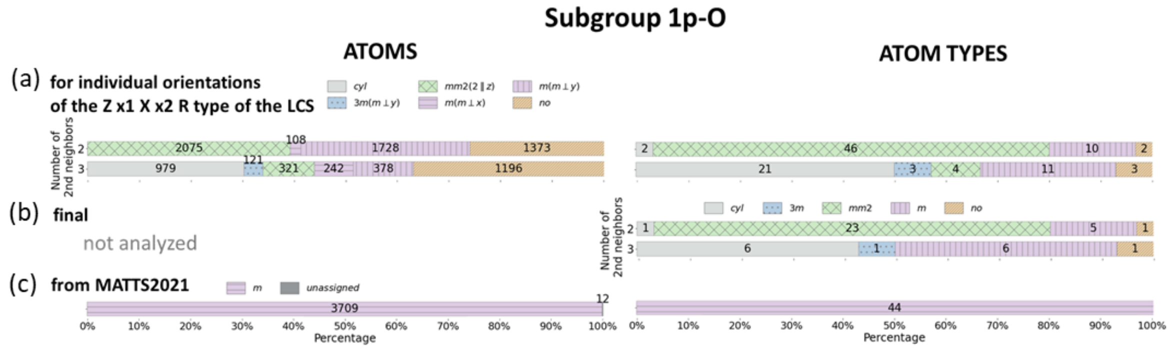

Figure S3.16 A comparison of pseudosymmetry of electron density (for individual orientations of the Z x1 X x2 R LCS type (a), final pseudosymmetry (b)) assigned for planar oxygen atom types (right) with one first neighbor and two or three second neighbors on the basis of the P_{lm} values. Symmetry for atoms and atom types in the MATTS2021 data bank is presented in the panel (c). Number of atoms with each pseudosymmetry is shown on the bars.

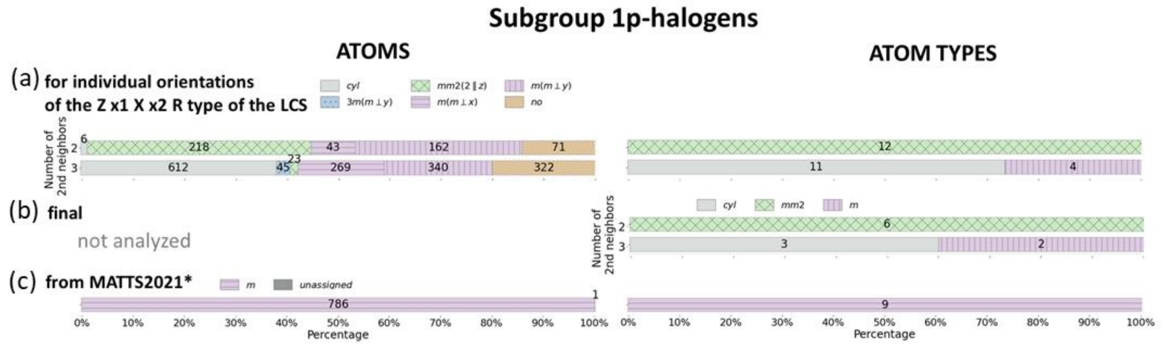

Figure S3.17 A comparison of pseudosymmetry of electron density (for individual orientations of the Z x1 X x2 R LCS type (a), final pseudosymmetry (b)) assigned for planar halogen (chlorine, bromine, fluorine) atom types (right) with one first neighbor and two or three second neighbors on the basis of the P_{lm} values. Symmetry for atoms and atom types in the MATTS2021 data bank is presented in the panel (c). Number of atoms with each pseudosymmetry is shown on the bars.

*Comment to Figure S3.17c: There were six types where the number of the second neighbors was strictly defined as either two (Cl02, Cl03, Br001, and F002) or three (Cl01a and Cl01b). However, there were three atom types (Cl01x, Br002, and F001) that had the number of second neighbors defined in a more broad way including "any number of any atoms" (Table S4.12). For the purpose of this work, we split these atom types into two separate modified definitions – one with two and another one with three second neighbors (Cl01x-2, Cl01x-3, Br002-2, Br002-3, F001-2, F001-3). It turned out

that the Br002-2 atom type did not actually include any atoms from the model molecules, and it was excluded from the further analysis. Thus why, the number of 1p-halogens atom types in MATTS2021 is nine, but the analysis includes 11 atom types.

Atom types

For oxygen atom types with two second neighbors (1p-O-2) the distributions of pseudosymmetries assigned to individual LCS orientations (Figure S3.16a right) and the final pseudosymmetries (Figure S3.16b right) were almost the same. Comparing to individual LCS orientations for individual atoms, the percentage of the mm2 pseudosymmetries increase more than two times, m decreased twice, and no almost disappeared. The final pseudosymmetry mm2 was assigned to 23 atom types, m to five atom types, no for one atom type (O372), and cyl for one atom type (O122f).

For oxygen atom types with three second neighbors (1p-O-3) the pseudosymmetry assigned to individual LCS orientations was cyl in 50 % and m in 25 % of cases (Figure S3.16a right). Comparing to atoms, these percentages increased, whereas the percentage of no pseudosymmetries visibly decreased. The final pseudosymmetries of atom types were: m for six atom types, cyl for six atom types (less in percentage than for individual LCS orientations), 3m for one atom type (O189 in ClO_4^-), and no for one atom type (O121). Interestingly, the symmetries usually follow the number of formally double, single and resonant bonds to the first neighbor (Table S4.11).

All six halogen atom types with two second neighbors (including Cl01x-2 and F001-2) had the mm2 pseudosymmetry assigned, for both individual LCS orientations (mm2(2‖z), Figure S3.17a right) and for final assignment (mm2, Figure S3.17b right). Comparing to individual atoms, averaging by atom type increased the pseudosymmetry for half of them (Table S4.12).

In the case of halogen atom types with three second neighbors, two of them (Cl01a in chloroform molecule and Cl01b in dichloromethane molecule) had the m pseudosymmetry assigned, whereas the remaining three of them (including Cl01x-3, Br002-3, and F001-3) had the cyl pseudosymmetry assigned. The distributions of final pseudosymmetries and of individual LCS orientations for atom types were similar, but they differed from distribution for individual LCS orientations of atoms (Figure S3.17a-b right). As for chlorine atoms with two first neighbors, averaging by atom type led to increased pseudosymmetries for almost half of atoms (Table S4.12). Comparing 1p-O and 1p-halogens subgroups, halogen atom types tend to be more symmetric.

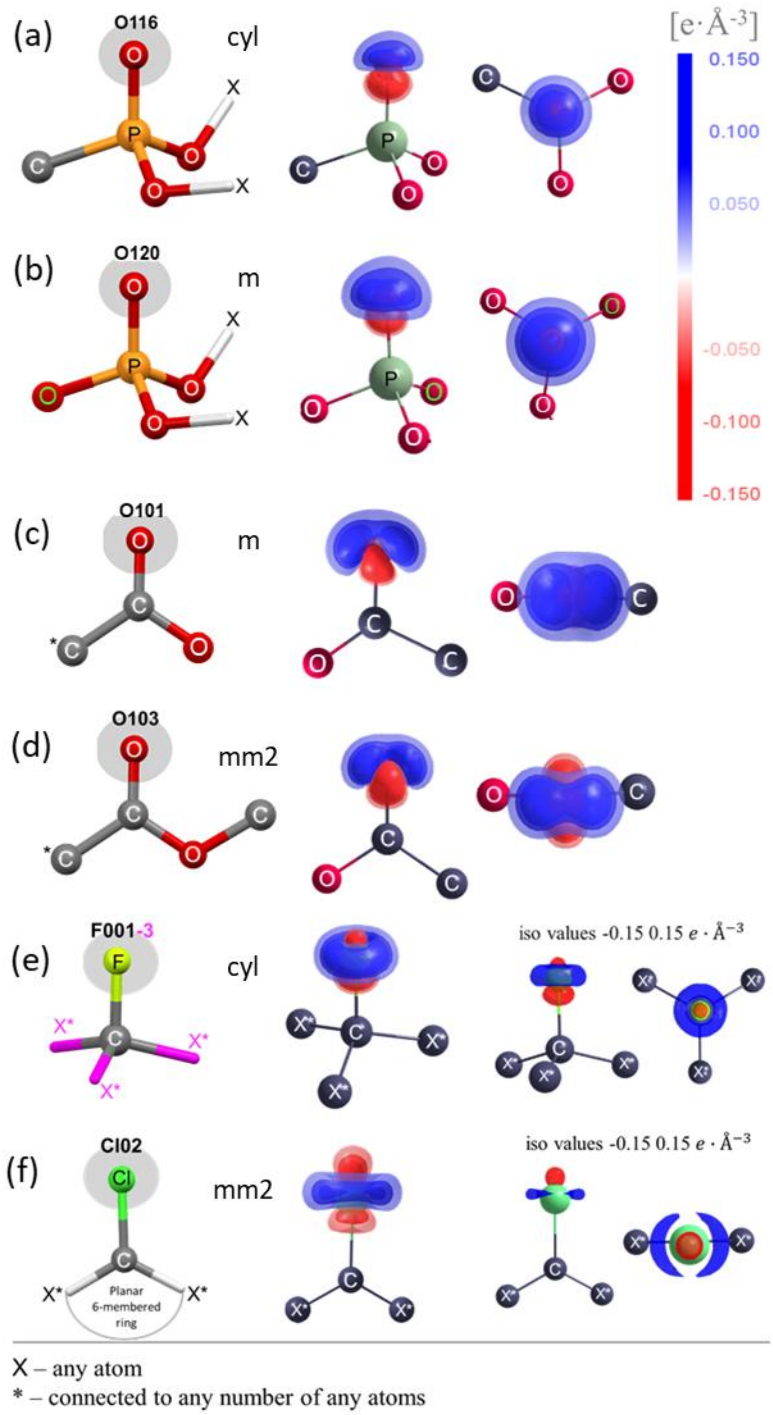

Figure S3.18 Deformation electron density maps for atom types O116 (a), O120 (b), O101 (c), O103 (d), F001-3 (e), and Cl02 (f) with the graphical visualization of the definition of the atom type in the MATTS2021 data bank and specification of the final assigned pseudosymmetry from the analysis. Atom type F001-3 does not actually exist in the MATTS2021 data bank but is a modification of existing atom type F001 made to explicitly define the number of second neighbors as three. The added parts of the definition are shown in magenta.
